# Chemosensing of honeybee parasite, *Varroa destructor*: Transcriptomic analysis

**DOI:** 10.1038/s41598-017-13167-9

**Published:** 2017-10-12

**Authors:** Nurit Eliash, Nitin K. Singh, Starlin Thangarajan, Noa Sela, Dena Leshkowitz, Yosi Kamer, Ilia Zaidman, Ada Rafaeli, Victoria Soroker

**Affiliations:** 1 Institute of Plant Protection, Agricultural Research Organization, The Volcani Center, Rishon LeZion, Israel; 2 Institute of Agroecology and Plant Health, Robert H. Smith Faculty of Agriculture, Food and Environment, Hebrew University of Jerusalem, Rehovot, Israel; 3 Department of Biological Services, Weizmann Institute of Science, Rehovot, Israel; 4 Department of Food Quality and Safety, Institute of Postharvest and Food Sciences, Agricultural Research Organization, Volcani Center, Rishon LeZion, Israel

## Abstract

Chemosensing is a primary sense in nature, however little is known about its mechanism in Chelicerata. As a model organism we used the mite *Varroa destructor*, a key parasite of honeybees. Here we describe a transcriptomic analysis of two physiological stages for the Varroa foreleg, the site of primary olfactory organ. The transcriptomic analysis revealed transcripts of chemosensory related genes belonging to several groups. These include Niemann-Pick disease protein, type C2 (NPC2), gustatory receptors (GRs), ionotropic receptors (IRs), sensory neuron membrane proteins (SNMPs) and odorant binding proteins (OBP). However, no insect odorant receptors (ORs) and odorant co-receptors (ORcos) were found. In addition, we identified a homolog of the most ancient IR co-receptor, IR25a, in Varroa as well as in other members of Acari. High expression of this transcript in the mite’s forelegs, while not detectable in the other pairs of legs, suggests a function for this IR25a-like in Varroa chemosensing.

## Introduction

Chemosensing is the primary and apparently the oldest mode of sensing in nature. It is essential for animal survival, allowing behavioral modulation according to environmental input thus optimizing detection of food, mates, and enemy-avoidance *via* volatile and contact chemicals. As the most successful group on earth, Arthropods possess remarkable chemosensory abilities. Their olfactory neurons are encased in sensory hairs, mostly located on an appendage such as the antenna in insects^1^ or the forelegs in Acarids^2^. The sensory hairs are hollow perforated structures, into which the dendrite(s) of the olfactory neurons project within the sensillar lymph. Translation of chemical cues into an electrical signal that activates the nervous system is presumably stereotypic throughout the animal kingdom, generally requiring four stages: odorant solubilization/transport through the aqueous lymph; recognition by olfactory receptors on the dendritic membrane; activation of ion channels; and signal removal/degradation. Although the genomes of various taxa have been partially sequenced, our current knowledge of arthropod olfaction is based heavily on data acquired from insects. These studies have led to the identification of some major components of the olfactory machinery. Lipid carrier proteins: odorant-binding proteins (OBPs), chemosensory proteins (CSPs) and Niemann-Pick disease protein, type C2 (NPC2). Membrane-bound receptors: odorant receptors (ORs), ionotropic receptors (IRs) and their corresponding co-receptors (ORco and IRco) as well as gustatory receptors (GRs). In addition, associated proteins such as sensory neuron membrane proteins (SNMPs) were found to play a role in some organisms, and odorant-degrading enzymes (ODEs) are important for the termination of the signal^1,3–6^.

Most of the knowledge regarding Chelicerata chemosensing is limited to morphological and electrophysiological studies^7–11^. It is assumed that Chelicera branched from Hexapoda and Myriapoda just after arthropods invaded land (during the late Cambrian period)^12,13^. This long-term split apparently resulted in profound differences in the sequences of molecular components between the groups which remain the main obstacles in the annotation of the relevant protein components. Fundamental research on non-insect arthropods is needed in order to form a more general picture of the molecular components of the chemosensory systems.

Recently, genomic, transcriptomic and proteomic studies revealed that some of the known insect’s chemosensory protein families have homologs in a few Chelicerata species, such as NPC2, CSPs, IRs, SNMPs and GRs^14–17^. In addition, a new soluble protein family, in which its predicted tertiary structures resemble OBPs but have low sequence similarities, was found in the lone star tick *Amblyomma americanum*, therefore named “OBP-like”^14^. Subsequently, members of this new group were also found in the deer tick, *Ixodes scapularis*, in spiders (order Aranaea): *Dysdera silvatica* and *Stegodyphus mimosarum*, and in the genomes of the centipede *Strigamia maritime* (Mandibulata)^17^. As animals often alter their chemosensory input by modulating expression levels of chemosensory related genes in response to environmental and physiological changes^18^, transcriptomic studies are expected to disclose chemosensory components. This strategy was adopted in the present study.

We used the mite *Varroa destructor* Anderson & Trueman (Acari: Varroidae) as a model arthropod. Varroa is an obligatory ectoparasitic mite of honey bees (*Apis spp*.), considered to be one of the major causes of honeybee colony losses almost worldwide^19^. The significance of honeybees for pollination and for sustainable agriculture is well recognized. The Varroa life cycle is synchronized with that of the honeybee’s and can be generally divided into two main phases: a phoretic phase, in which the Varroa parasitizes and is carried by an adult bee (nurse or forager), and a reproductive phase, in which the Varroa reproduces within the sealed brood cell^20^. The entrance of a Varroa female into the brood cell is synchronized with the developmental stage of the bee larvae^21^. The strong association between the Varroa’s and the bee’s life cycles underscores the importance of Varroa chemical perception for its host finding, selection and reproduction^20,22,23^. Recently we found that phoretic mites are significantly more successful in reaching an adult bee host^24^, and identified a gene transcript for pheromone receptor transcription factor-like (PRTF-like), which is involved in Varroa chemosensing and host finding behavior. Silencing this gene using RNA interference (RNAi), effectively converted a phoretic mite into a reproductive mite. This change was associated with a reduction of both Varroa host sensing and ability to reach a host, while vitellogenin gene (Vg) levels were upregulated^24^. Higher expression of Vg in reproductive and PRTF-silenced mites, corresponds with a former report regarding the role of Vg in Varroa reproductive readiness^25^. Despite the significance of PRTF-like in phase transition of the Varroa mite, the essential elements of the chemosensory machinery remained to be identified.

As the Varroa genome is poorly annotated with merely short sequences^26,27^, and genes related to chemosensing in Chelicerata are still mostly unknown, we conducted a transcriptomic analysis of the Varroa foreleg, the site of the mite main olfactory organ. Focusing on chemosensory related genes, we compared transcriptomes between phoretic and reproductive stages, in which differential host orientation occurs.

## Results and Discussion

### *De novo* assembly and differential expression

**Figure 1:**
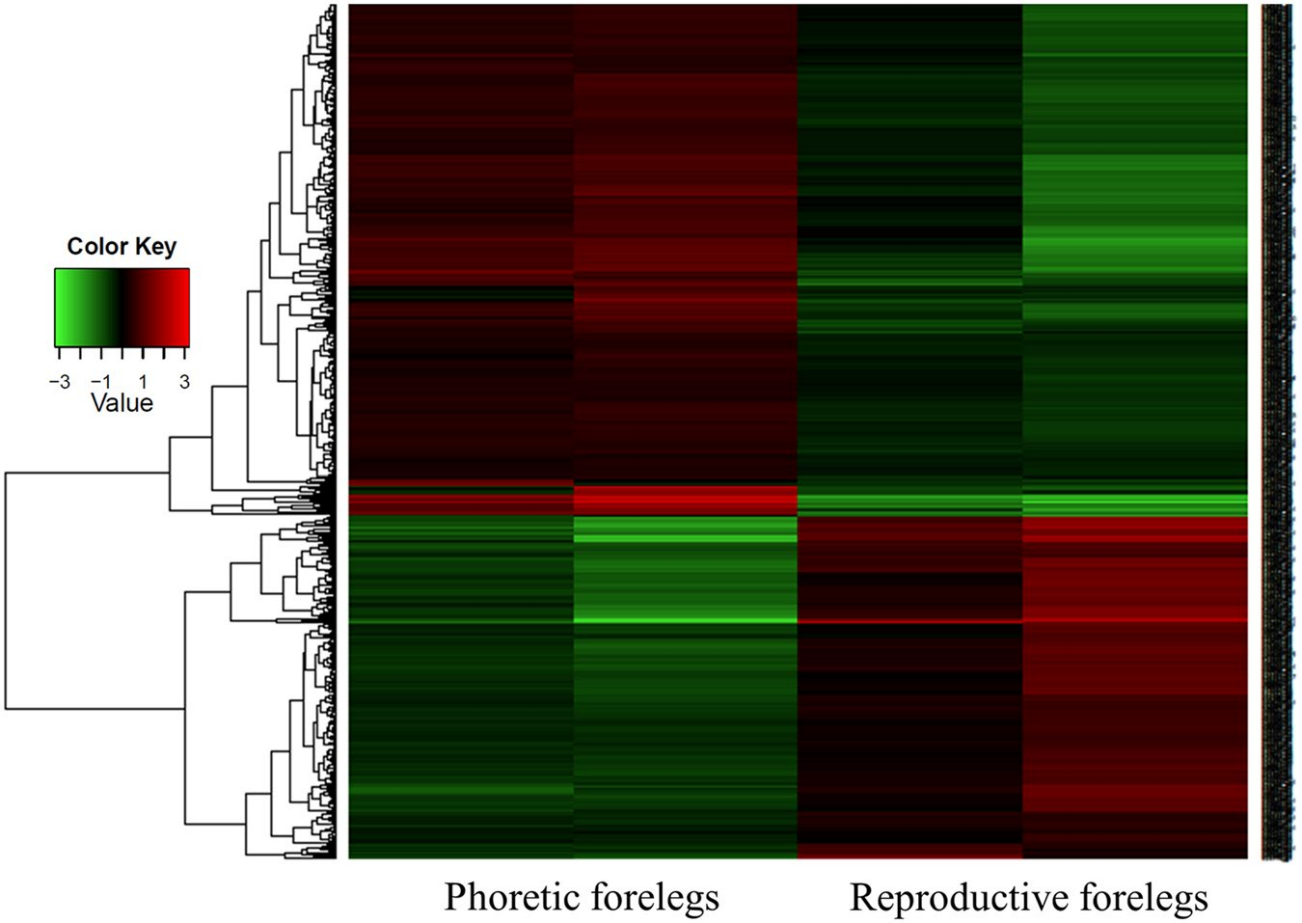
Heat maps of the expression profiles of the mite forelegs from two physiological stages: Phoretic and reproductive mites. The normalized expression values log2-transformed, median centered are displayed with the color scale in reference to forelegs of the reproductive stage mites: with down-regulated genes in green and up-regulated genes in red.

Illumina TruSeq platform generated a total of 559,287,718 reads, 276,824,996 for the phoretic stage and 282,462,722 for the reproductive stage, two replicates for each stage. To date, only a few Varroa genes were reported in the data bases (such as housekeeping genes^28^, as well as vitellogenin^25^, and some detoxification enzymes such as glutathione S-transferase)^29^. Therefore, the transcripts’ sequences were subjected to *de novo* assembly using Trinity software^30^, with default parameters. The quantification of alignments was done using RSEM package^31^. The Bioconductor DEseq. 2 package^32^ was used to identify differentially expressed transcript genes in phoretic and reproductive stages. A total of 154,037 contigs (GC% = 42.83; average contig = 1143.14 bp; N50 = 3022; Total assembled bases: 176085124) were found. These summed to 125,047 total trinity ‘genes’ that were subjected to functional annotations. All data were uploaded to SRA under BioProject accession PJNA383492. The assembled 154,037 transcripts were used as queries in BLASTx against the non-redundant (nr) NCBI database^33^. A Blast2GO analysis of the transcripts of Varroa identified 30,697 transcripts (20%) with blast hits and 123,340 (80%) without blast hit, 19,840 transcripts had annotations. From the transcripts with blast hits, 1,164 transcripts were mapped to *Apis mellifera* with E value less than 1e-3 and therefore are probably contamination from the host, the honeybee. The rest of the transcripts mapped primarily to Chelicerata: *Metaseiulus occidentalis* (24,423 transcripts), *Ixodes scapularis* (710 transcripts) and *Limulus polyphemus* (569 transcripts). Only 61 transcripts mapped to known *V*. *destructor* proteins emphasizing the relatively scant information currently available on Varroa in the public databases. This assembly is not expected to represent all Varroa transcripts since it is a transcriptome assembly from only one tissue of the mite. DEseq. 2 analysis revealed 799 differentially expressed gene transcripts, 479 down-regulated in the phoretic stage while 320 were up-regulated (Fig. 1). We used as a threshold for differentially expressed genes that were with FDR (false discovery rate) smaller than 0.05 and log fold change greater than 1 or lower than −1^34^.

In general, gene ontology (GO) term enrichment analysis revealed that reproductive mites’ forelegs were enriched with transcripts related to cell replication and interspecies interaction while those from phoretic mites were enriched in transcripts related to chitin, amino sugar and aminoglycan metabolic processes and cuticle constituent (Fisher’s exact test and Blast2GO)^35^. Specifically, transcriptomic analysis confirmed our recent findings regarding increased PRTF and decreased vitellogenin 1 and 2 expressions in forelegs of phoretic relative to reproductive stages^24^.

### Functional annotation of chemosensory related transcripts

Relying on the fact that tertiary and secondary structures are more conserved than the basic sequence^36,37^, we searched for known conserved domains related to chemosensory functions. Using this method, we identified several members of chemosensory protein families, including soluble proteins (OBPs and NPC2), chemosensory receptors (GRs and IGRs) and SNMPs (results summarized in Table 1). The presence of these transcripts in Varroa was confirmed by PCR (data not shown), as described in the methods section, and the presence of the conserved domain was confirmed by manual search for domains using both InterProScan^38^, and hmmscan (HmmerWeb version 2.7.2 (http://hmmer.org/))^38^. A list of the chemosensory transcripts, by conserved domains is available, as well as the complete transcripts sequences (see Supplementary Data S1 and S2 respectively).

**Table 1:** Families of chemosensory genes, their characteristic domain/s and their InterPro and Pfam signature; in each group the number of transcripts that contained the domains or at least one of them is noted, according to Blast2GO analysis.

Group	Domain description IPR	Domain (IPR)	Domain (Pfam)	Number of transcripts
OBPs	PBP_GOBP	IPR006170	PF01395	5
NPC2	MD-2-related lipid-recognition domain	IPR003172	PF02221	8
CSP	Insect odorant-binding protein A10/Ejaculatory bulb-specific protein 3/OS-D	IPR005055	PF03392	None
ORs	7tm Odorant receptor 7tm_6	IPR004117	PF02949	None
GRs	7m_7	IPR013604	PF08395	3*
	Trehalose receptor	IPR009318	PF08395	
IGRs	Receptor, ligand binding region (ANF)/amino terminal domain (ATD)	IPR001828	PF01094	46**
	Ligand binding domain (LBD)	IPR019594	PF10613	
	Ionotropic glutamate receptor/ion channel domain (ICD)	IPR001320	PF00060	
SNMP	CD36-Snmp family, CD36 antigen	IPR002159	PF01130	8

*Contain at least one of the two GR domains.

**Contain at least one of the three IGR domains.

For the first time, to our knowledge, we identified transcripts containing the conserved domain of PBP/GOBP (IPR006170) in a non-insect Arthropod (five transcripts). In addition, we identified eight transcripts that contained the conserved domain of NPC2 family (IPR003172). It is worthwhile to note that the search of NPC2 members posed a challenge as the transcripts had no chemosensory-associated go-term, therefore they were identified by searching for the presence of the conserved domains; while OBPs’ transcripts had also an “Odorant binding” go-term. We did not identify transcripts that contained the OS-D domain belonging to the CSP family (IPR005055) though members of this group were reported in the related Acari (*I*. *scapularis*)^15^, as well as in Crustaceans and Chilopods (*D*. *pulex* and *S*. *maritima*)^13,39,40^. On the other hand, CSPs were not found in the Acari *I*. *ricinus* ^16^ and their presence in *A*. *americanum* is controversial^14,41^.

Interestingly, Varroa transcripts containing the conserved domain of PBP/GOBP, show low similarity with insect OBPs (see Supplementary Fig. S3). However, they also do not bear similarity to the recently reported OBP-like transcripts in *A*. *americanum* ^14^ (MAFFT in “Auto” strategy, see Supplementary Fig. S4). In fact, the OBP-like transcripts that were reported in *A*. *americanum* do not contain the PBP/GOBP domain (domain search using both InterProScan^38^, and hmmscan (HmmerWeb version 2.7.2 (http://hmmer.org/))^38^. These findings would suggest that the Varroa OBPs found in the present study belong to a new arthropod OBP family.

In accordance with earlier publications of non-insect arthropods, homologs to the classical seven-transmembrane odorant receptors (Olfactory receptor, insect (IPR004117)) were not found among Varroa foreleg transcripts. On the other hand, three transcripts of the GR family, two containing the two conserved domains (IPR013604 and IPR009318), while the third containing only the first domain were detected. From the IGR family, we identified 46 transcripts which encode for the conserved IGRs domains: 27 transcripts contained only one of the three domains, another five transcripts encode for the two conserved domains (IPR019594 and IPR001320), and additional 14 transcripts contained all three domains.

In addition, we also found eight transcripts with the conserved domain of the SNMP family (IPR002159). SNMPs, orthologs of vertebrates’ CD36 (a fatty acid receptor)^42^, are known as membrane proteins associated with pheromone sensitive neurons in Lepidoptera and Diptera, and participate in the processes of pheromone signal transduction^43^.

**Figure 2:**
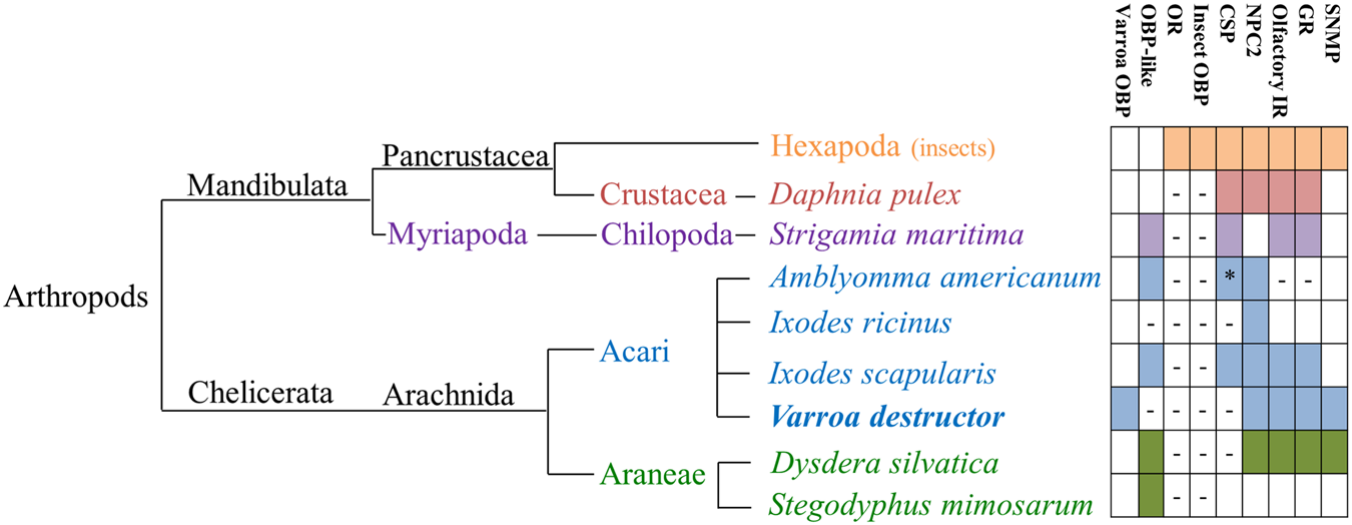
Scheme of annotated sequences of chemosensory components identified by genome, ESTs, transcriptome, or protein datasets of Hexapods and the indicated non-insects Arthropods species: *Daphnia pulex* ^40,50^, *Strigamia maritimia* ^13,17^, *Amblyomma americanum* ^14,41^, *Ixodes ricinus* ^16^, *Ixodes scapularis* ^15,17,41,50^, *Dysdera silvatica* ^17^, *Stegodyphus mimosarum* ^17^, and *Varroa destructor* (as was found in the current study). The organisms are ordered according to the tree on the left (the tree was built based on NCBI Taxonomy Browser). Colors distinguish insect orders or other groups. (-) indicates that the authors did not found members of this group in the datasets; (?) indicates that the authors did not deny/approve the presence of this group; (*) indicates inconsistent reports: No CSP genes were detected by Renthal *et al*.^14^, but one copy was found by Vieira and Rozas^41^.

The absence of insect OR and ORcos in the Varroa transcriptome (Fig. 2), supports the hypothesis that insect ORs originated after the split of the Hexapoda and Crustacea (B470Mya). However, the presence of transcripts with insect OBP domains within the Varroa transcriptome contradicts the hypothesis that insect OBPs originated at the same era as ORs, as previously suggested^15,40,41^.

### Varroa IGR repertoire

We further focused on the examination of the transcriptome for the IGRs, the superfamily from which both the iGluRs and the IRs, the most ancient chemoreceptor family in protostomes, originated^44^. IGRs are believed to contain three domains: The ligand binding domain (LBD, InterPro domain: IPR019594); the ion channel domain (ICD, InterPro domain: IPR001320); and a more variant amino terminal domain (ATD, InterPro domain: IPR001828). According to former genomic surveys of the evolution of IGRs across protostomes^44,45^, transcripts that contained all three domains, were predicted to belong to the iGluR family; while those that lack, or have a shorter ATD, were predicted to belong to the IR family. Previous molecular evolutionary analysis suggested that the IR family in *D*. *melanogaster* has two subfamilies: “antennal IRs” (also called “olfactory IRs”) and “divergent IRs”^44^. As opposed to the antennal IRs, which are expressed in antennae only, the divergent IRs are expressed in both chemosensory related organs (olfactory and gustatory), as well as in other tissues, such as the brain^44^.

In order to classify the Varroa IGRs found in this study into the above-described sub-groups, we implemented both domain-based and phylogenetic analysis classification. Initially, using Blast2GO, we checked for the presence of any of the three characteristic IGR domains. As previously mentioned, this initial scan method revealed 46 transcripts that contain at least one of the three domains (Table 1 and Supplementary Data S5). Out of these 46 transcripts, only 19 were found to contain the two essential conserved domains (IPR019594 and IPR001320, Table 1). As the function of IGR proteins presumably requires the presence of these two domains^44–46^, we excluded all the other transcripts from subsequent analysis. The presence and length of the domains for each of the remaining 19 transcripts was verified manually using InterProScan^47^, and hmmscan (HmmerWeb version 2.7.2 (http://hmmer.org/))^38^. To avoid misinterpretation of the IGR sub-groups due to technical limitation of the sequencing or assembly process, which is based on the presence of the ATD conserved domain, we adopted a conservative approach. Transcripts that contained the two conserved domains but ORF positioned at the N-terminal of the sequence, without a methionine at the N-terminal were excluded from further analysis. According to the literature, all three domains usually appear in a single ORF. We thus excluded one transcript that contained the three domains, but in two separate ORFs (the first containing the variant domain, and the second containing the two conserved domains (Vd20855)). Final transcript evaluation revealed 17 valid transcripts, 14 of which contain all of the three domains, and another three that contain only the two conserved domains.

**Figure 3:**
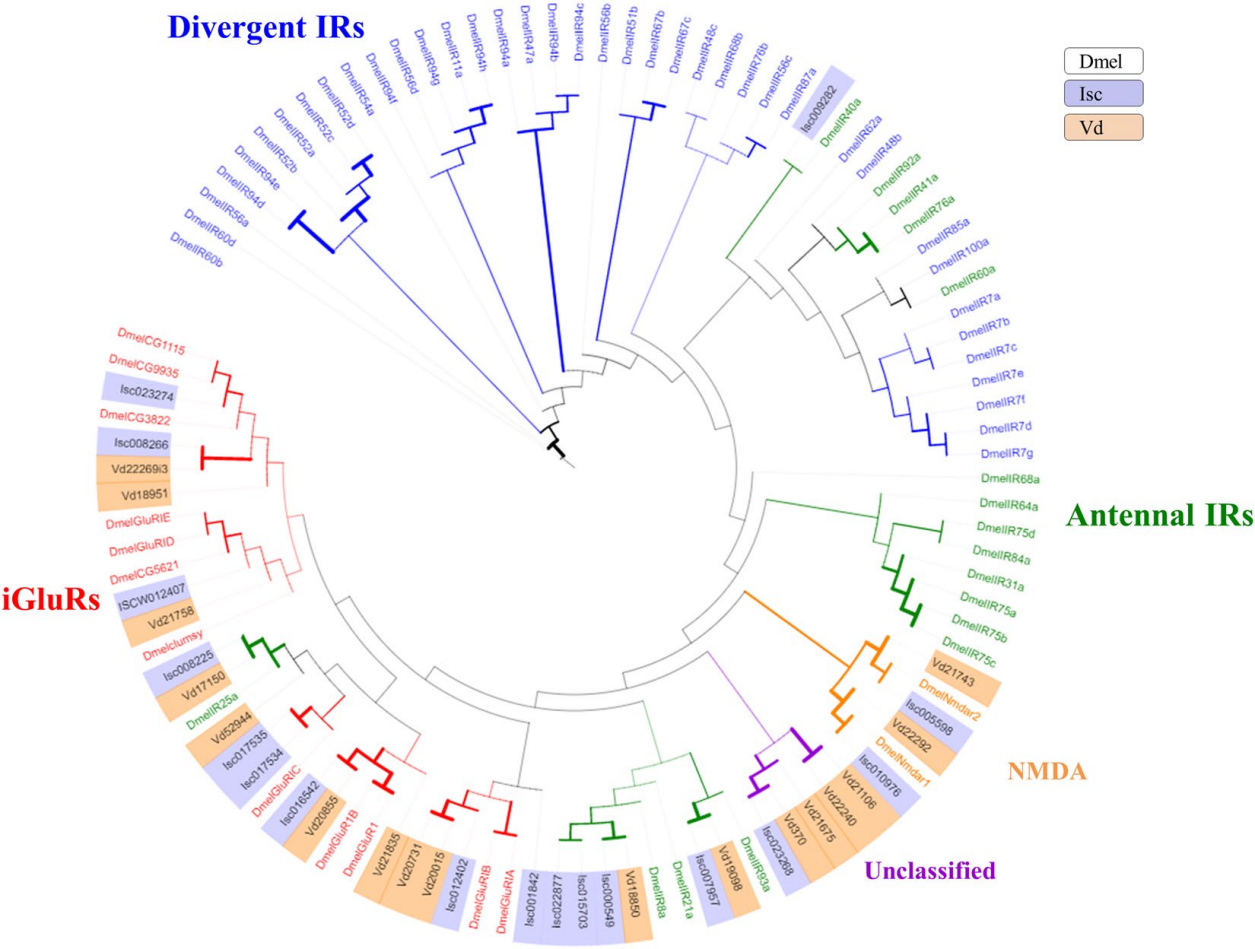
Phylogenetic tree of IGR repertoires of *D. melanogaster* (Dmel), *Varroa destructor* (Vd) and *I. scapularis* (Isc) amino acid sequences (total of 105 sequences). The tree was constructed using PhyML^53^ under the LG model of substitution^55^, and is based on the conserved columns of the open reading frame of the amino acid alignment using MAFFT^51^. Bootstrap values were estimated using an approximate likelihood ratio test and are displayed proportionate to the width branch (0-500).

To extend the Varroa IGR investigation, we applied a phylogenetic analysis including the IGR repertoire of the most studied arthropod, *D*. *melanogaster* ^44^, and Varroa’s close relative, *I*. *scapularis* ^26^. Since *I*. *scapularis* IGRs are not annotated, we first scanned the tick translated genome (VectorBase, http://www.vectorbase.org, *I*. *scapularis scapularis* PEST, IscaW1.4), using Blast2GO for the presence of the three characteristic IGR domains, as was performed for the Varroa transcriptome. The initial scan revealed 73 translated transcripts that contained at least one of the three IGR conserved domains (only 44 of them had also a GO-term of “Glutamate receptor”, the others were “uncharacterized”). The second filtration, using InterProScan^38^, and hmmscan (HmmerWeb version 2.7.2 (http://hmmer.org/))^38^, revealed 17 sequences, nine with all three domains present, and the other eight with only two conserved domains (see Supplementary Data S6). Predicted amino acid sequences of *I*. *scapularis* (17) were aligned with those of Varroa (17) and *D*. *melanogaster* (71). In total, 105 sequences (Supplementary Data S7) were used for the construction of the phylogenetic tree as described in the methods section (Fig. 3).

The phylogenetic analysis revealed that out of the 17 Varroa IGR transcripts, seven are close to the iGluR group, whilst two are close to the NMDA sub-group, and another three bear a similarity to the *D*. *melanogaster* “Antennal IRs”. Another four Varroa transcripts formed a distinctive sub-group along with one sequence of *I*. *scapularis* (ISCW023268). This group could represent a new Acarian IGR sub-group, as no *D. melanogaster* representatives are present in this branch. However, this assumption cannot be assessed to date, mostly due to the scarcity in Acari genomic and transcriptomic data in the available databases. Therefore, these transcripts, (along with another inconclusive transcript, Vd52944) are referred to as an “unclassified group”.

**Figure 4:**
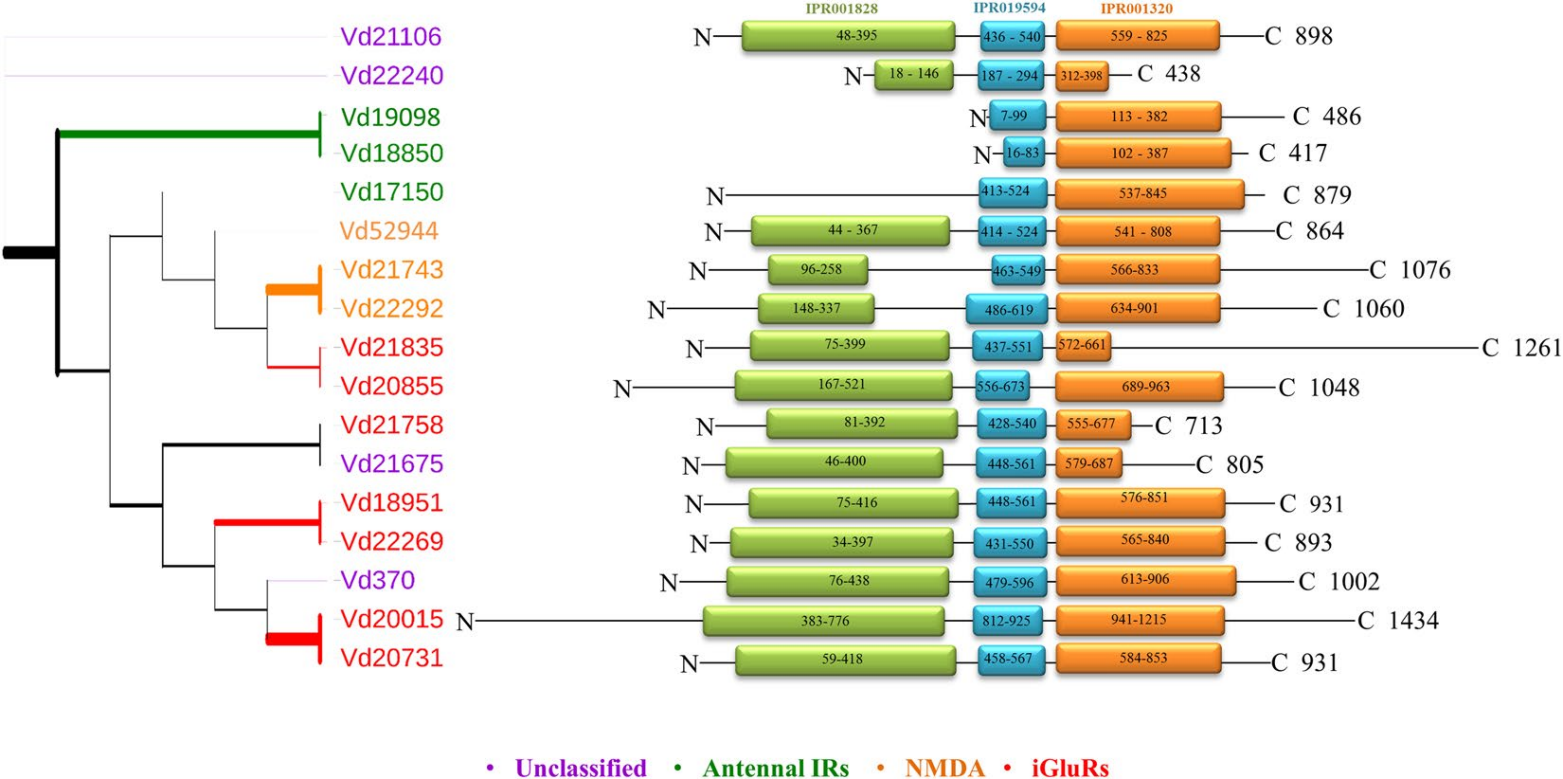
Phylogenetic tree of IGR repertoires of Varroa (Vd) amino acid sequences (17)^17^, and its corresponding sequences with their protein domains: Receptor family ligand binding region (IPR001828), Ligated ion channel L-glutamate; glycine-binding site (IPR019594) and Ligand-gated ion channel (IPR001320). The tree was constructed using PhyML^55^ under the LG model of substitution^57^, and is based on the conserved columns of the open reading frame of the amino acid alignment using MAFFT^53^. Bootstrap values were estimated using an approximate likelihood ratio test and are displayed proportionate to the width branch (0-500). The presence, length and position of the protein domains were predicted using hmmscan, HmmerWeb version 2.7.2 (http://hmmer.org/)^38^, and InterProScan^47^. The sequences were distributed into the 4 different groups (“Antennal IRs”, “NMDA”, “iGluRs” and “unclassified”), according to their proximity to *D. melanogaster* sequences in the combined phylogenetic tree in Figure 3.

The phylogenetic distribution corresponds to the domain structure of the sequences (Fig. 4): All Varroa iGluR members contain all the three IGR domains, while the three “Antennal IR” members have the characteristic “Antennal IR” structure and lack the third domain (ATD, IPR001828). Our analysis has not revealed any “divergent IR” members among Varroa as well as in *I*. *scapularis* transcripts. This might suggest that this sub-group has emerged after the branching of the insects and Chelicerates.

**Figure 5:**
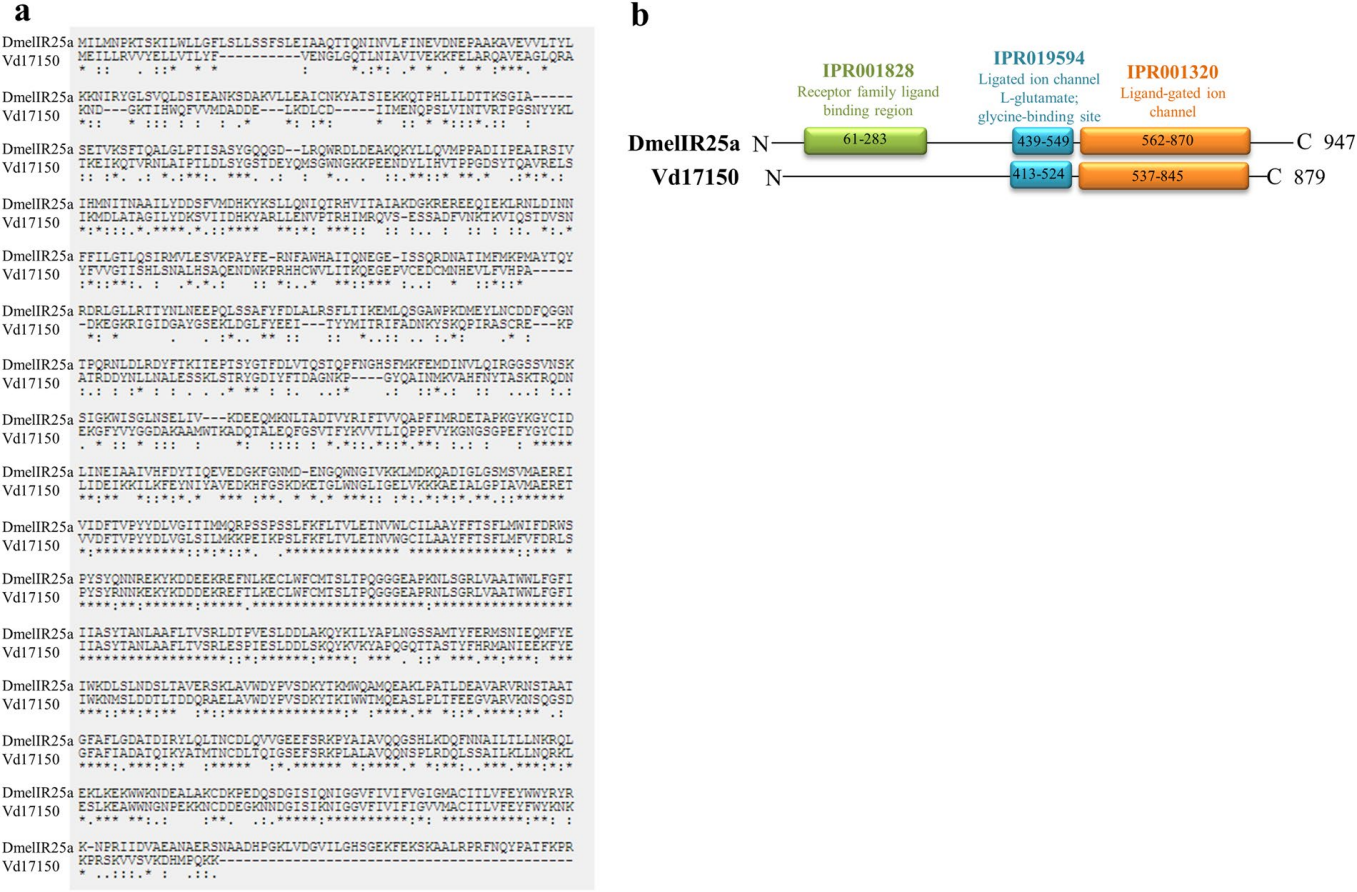
**(a)** Pairwise alignment of amino acid sequences of *D. melanogaster* IR25a (DmelIR25a) and its Varroa homolog transcript (Vd17150), with 41.3% identity. Alignment was done using Pairwise alignment EMBOSS Needle. **(b)** Conserved domains found according to InterPro scan, of amino acid translated sequences of *D. melanogaster* IR25a (DmelIR25a) and its homolog in Varroa mite (Vd17150).

Among the Varroa “Antennal IR” transcripts, it can be assumed that Vd17150 codes for a protein homolog to *D*. *melanogaster* co-receptor DmelIR25a (Fig. 5a), since the two sequences bear 45% identity, 95% cover (Pairwise alignment EMBOSS Needle), and share the same phylogenetic branch (Fig. 3). We therefore named this gene “Varroa IR25a-like homolog” (Accession MF069507). However, the Varroa IR25a-like has the structural pattern of a typical “antennal IR” with only the two conserved domains present (IPR019594 and IPR001320) (Fig. 4), while *D*. *melanogaster* IR25a has all the three domains^45^. As can be seen in Fig. 5b, the transcript structure of the Varroa IR25a-like shows a complete ORF, with both C and N termini. The closeness in the phylogenetic tree of ISCW008225 in *I*. *scapularis*, *D*. *melanogaster* IR25a and the Varroa IR25a-like, suggests that ISCW008225 is also a homolog of IR25a. However, a caution needs to be taken when a phylogenetic based classification is implemented, and the function of IR25a-like homologs as co-receptors in mites awaits experimental confirmation.

**Figure 6:**
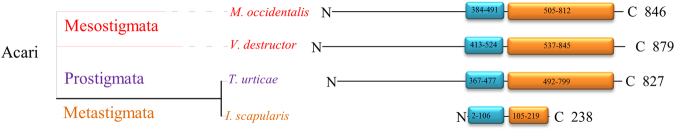
Phylogenetic tree of IR25a homologs of the Acarians: *M. occidentalis* (Acari, Parasitiformes, Mesostigmata), *V. destructor* (Acari, Parasitiformes, Mesostigmata), *Tetranychus urticae* (Acari, Acariformes, Prostigmata) and *I. scapularis* (Acari, Parasitiformes, Metastigmata). For each sequence the protein’s domains are presented: Ligated ion channel L-glutamate; glycine-binding site (IPR019594) and Ligand-gated ion channel (IPR001320). The tree was constructed using PhyML^55^ under the LG model of substitution^57^, and is based on the conserved columns of the open reading frame of the amino acid alignment using MAFFT^53^. Bootstrap values were estimated using an approximate likelihood ratio test and are displayed proportionate to the width branch (0-500). The presence, length and position of each proteins’ domains were predicted using hmmscan, HmmerWeb version 2.7.2 (http://hmmer.org/)^38^.

In order to have a better understanding of the origin of IR25a within Acari, we searched for sequences similar to the Varroa IR25a-like (Blastp against the non-redundant protein database, NCBI). We then chose sequences of representative Acari groups: *M*. *occidentalis* (Acari, Parasitiformes, Mesostigmata), *I*. *scapularis* (Acari, Parasitiformes, Metastigmata), and *Tetranychus urticae* (Acari, Acariformes, Prostigmata), and searched within each sequence for conserved domains (HmmerWeb version 2.7.2 (http://hmmer.org/))^38^. The sequences were then aligned along with Varroa’s IR25a-like sequence, and a phylogenetic tree was constructed, as described in the method section. The latter phylogenetic analysis revealed that all IR25a-like homolog transcripts of the four Acari representatives have the structural pattern of a typical “antennal IR” with only the two conserved domains present (IPR019594 and IPR001320) (Fig. 6). A recent analysis of another parasitic mite of honeybees, *Tropilaelaps mercedesae*, has also revealed the presence of transcripts similar to DmIR25a and DmIR93a, and both genes were highly expressed in the forelegs of the mite^48^.

It is important to note that although genome assemblies among a few members of other Acarian orders such as *Hypochthonius rufulus* and *Platynothrus peltifer* (Acari, Acariformes, Oribatida), are available in the NCBI database, a BLAST search against those genomes yielded only partial sequences thus not suitable for reliable alignment. It seems that despite the accelerated investigation of non-insect Arthropods in the last few years, not enough data have been acquired, and a more wide genomic survey across Acari requires more trustworthy and complete assemblies.

It should be taken into consideration, however, that the strict approach that we implemented in this study could have filtered out some members of the IGR group, and thus may affect this phylogenetic analysis. Needless to say, the function of all IGRs found in this study requires further investigation.

### Differential expression of chemosensory transcripts

From the families of IGR, GR, NPC2, SNMP, and OBP, one member of each family was highly expressed in all four samples of both stages (Vd370 in IGR, Vd7144 in GRs, Vd69937 in NPC2, Vd11771 in SNMP and Vd14320 in the OBP family, see Supplementary Figs S8–S12). The similarity in expression levels of these chemosensory transcripts in the two stages could be explained by the fact that in both stages Varroa requires chemosensing. In the phoretic stage, Varroa needs to differentiates between different bee hosts^11^, while in the reproductive stage, the mite needs to synchronize its reproduction in accordance with the stage of the developing bee pupa^49^. The high expression of these specific transcripts may indicate their importance in chemosensation in both stages, and therefore should be further investigated. In addition, one should also keep in mind the possibility that due to our stringent analysis we may have overlooked some relevant transcripts, and thus could have missed certain stage specific differences.

### Quantitative-PCR analysis for relative gene expression of Varroa IR-like genes

**Figure 7:**
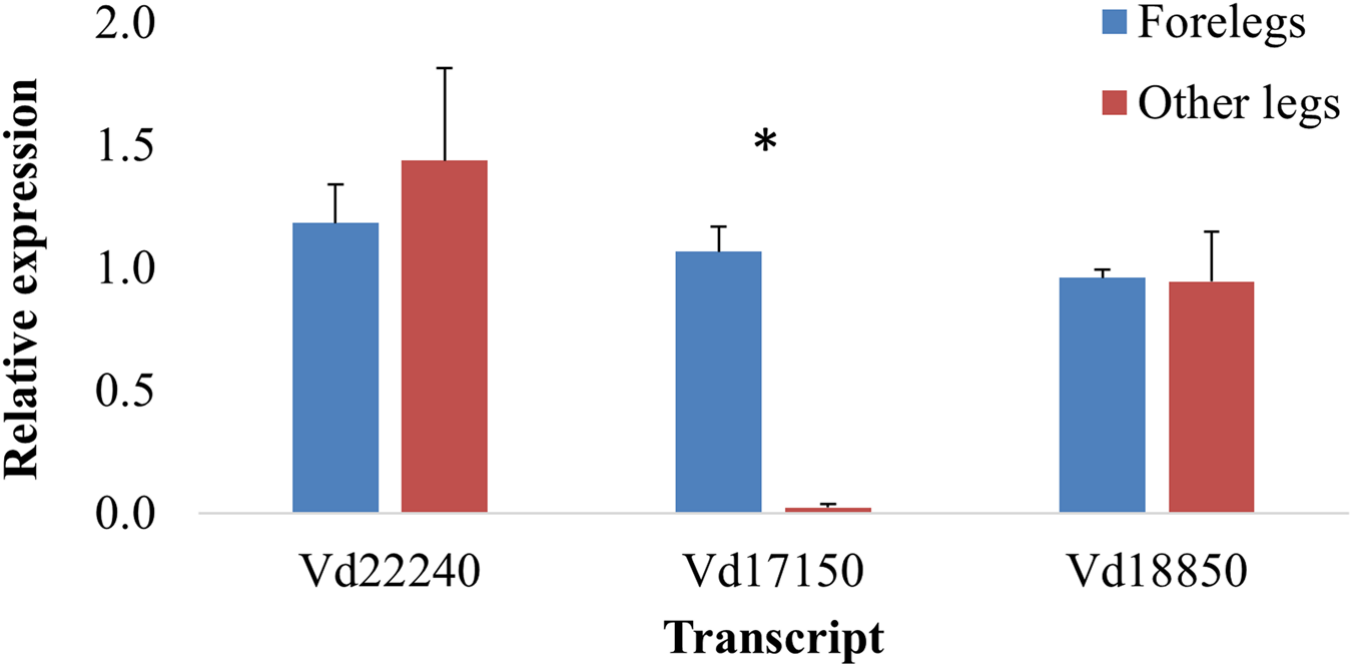
Relative expression of IR-like gene transcript in the female Varroa mite foreleg and other legs. Results were normalized to the host 18S rRNA gene transcript. Each experiment was performed in triplicates and statistically analyzed using T-test, different letters indicate significant difference, p < 0.05. Error bars indicate the standard deviation.

As the transcriptomic analysis revealed no differential expression in the chemosensory related transcripts between forelegs of phoretic and reproductive stages, we examined the relative expression using qPCR between the mite foreleg and other legs, assuming that the forelegs contain the main olfactory organ, while the other three pairs of legs possess no chemosensory function. We focused on Varroa IR-like transcripts: Vd22240 (from the “unclassified group”), Vd18850 (from the IR group) and Vd17150 (the IR25a-like in the IR group), according to the phylogenetic and domain-based analysis (Fig. 4). We found that the relative expression of Varroa IR25a-like was significantly higher in the forelegs compared to the other pairs of legs, while the expression levels of Vd22240, Vd18850 were not significantly different between both tissues (Fig. 7). This strongly suggests a function for IR25a-like in chemosensation in Varroa. However, this function will require future physiological and behavioral confirmation.

In summary, comparative transcriptomic analysis revealed a differential expression profile of the mite forelegs, between the two physiological stages, phoretic and reproductive. Since most of the differentially expressed genes have not yet been annotated, the significance of these differences cannot yet be speculated. The major finding in this study was the detection of chemosensory related transcripts based on domain search, some of them for the first time in Acari; including NPC2, SNMPs, GRs, OBPs, and the most ancient chemoreceptor family of IR. To our knowledge, this is the first report of OBP-domain sequences in a non-insect Arthropod. We found that Varroa possesses a few members of the olfactory-IR family among its IGR transcripts, including the most ancient IR, the co-receptor IR25a. As opposed to its homolog in *D*. *melanogaster*, in Varroa and other Acari IR25a-like exhibits two of the characteristic IR domains (IPR019594 and IPR001320), while missing the third domain (ATD, IPR001828). The significantly higher expression of Varroa IR25a-like in the forelegs relative to its undetectable expression in the other pairs of legs, along with the phylogenetic results, suggests that this transcript may play a role in Varroa chemosensing. Our phylogenetic analysis of all IGR-like transcripts reveals that both Varroa and *I*. *scapularis*, have no “divergent IR” members among their IGR transcripts, but possess another IGR sub-group, which is probably unique to Acari. No insect OR and ORcos were found. In addition, contrary to former reports^15,41,50^ members of the CSP family in related Acari (*A*. *americanum* and *I*. *scapularis*) were not found in the Varroa transcriptome.

## Materials and Methods

### Biological material

Honey bee colonies (*A*. *mellifera liguistica*) were maintained at an experimental apiary at Rishon LeZion, Agricultural Research Organization, Volcani Center, Israel. The experimental hives were maintained without any treatment against Varroa, but they received seasonal sugar and 70% pollen cake feeding when needed.

### Collection of mites

Mites were collected separately for transcriptomic and qPCR analysis. For the transcriptomic analysis, two types of adult female mites were collected for the different experiments: “Phoretic mites” and “reproductive mites”. Collection of phoretic mites was performed by taking a brood frame out of the hive and brushing off the adult bees. Emerging bees were allowed to naturally emerge at 26–30 °C, and phoretic adult female mites were collected from emerging bees, using fine tweezers and a fine paintbrush. Mites were maintained on moist filter paper and dissected up to 1 hour after emerging. Reproductive mites were collected from purple-eyed pupae, and used within 1 hour of collection. For qPCR analysis, mites were collected from bottom board trays, maintained on moist filter paper, and dissected up to 1 hour from collection.

### Total RNA extraction

Total RNA of Varroa forelegs and bodies devoid of forelegs was extracted using GeneAll Kit (Seoul, South Korea) as described before^24^. In short, forelegs were excised from the mite using a fine surgical blade (size no. 11) under a stereo microscope (Olympus SZX12, Shinjuku-ku, Tokyo, Japan). For each replicate, 50 forelegs (for transcriptome study) and 80–90 forelegs (for qPCR study) were homogenized and RNA extraction was carried out according to manufacturer’s instructions. The quality and quantity of the RNA were measured using a NanoDrop 2000 spectrophotometer (Thermo Scientific, Wilmington, DE, USA).

### Transcriptomic analysis

RNA samples of two replicates for each stage were processed by Technion Genome Center (Israel) according to the Ilumina TruSeq RNA Library Preparation Kit v2. The constructed libraries were sequenced in one lane on program 100 PE using SBS V reagent, in an Illumina HiSeq. 2500 System. The complete paired-end sequences were obtained as individual FASTQ files.

### *De novo* transcriptome assembly

*De novo* assembly was performed by Trinity V2.2.0 with default parameters^51^. Before assembly, raw reads were trimmed by removing adaptor sequences and ambiguous nucleotides. Reads with quality score less than 20 and length below 50 bp were also filtered out. Gene and transcript quantification was done using RSEM^31^. The Bioconductor DEseq package^52^ in the R environment was used to identify differentially expressed genes in phoretic versus reproductive stages.

### Phylogenetic analysis

Sequences were aligned using MAFFT in “Auto” strategy^53^. Due to the high divergence among sequences and many gaps, we applied curation approach to remove any ambiguous regions (i.e. containing gaps and/or poorly aligned) with Gblocks (v0.91b) under a “less stringent selection”, and trimAl (parameters for columns removal: all columns with gaps in more than 40% of the sequences or with a similarity score lower than 0.001)^54^. The realigned conserved blocks were subjected to a maximum-likelihood analysis using Phyml, 500 bootstraps^55^. The visualization of the phylogenetic tree analysis was done using iTOL server^56^.

### Functional annotation of chemosensory related transcripts

In order to search for chemosensory related genes, we integrated the traditional strategy of a sequenced-based BLAST search, along with a domain-based search for the presence of specific domains characteristic for each family using Blast2GO with default parameters^35^. We used InterproScan with the following domain search: BlastProDom, FPrintScan, HMMPIR, HMMPfam, HMMSmart, HMMTigr, ProfileScan, HAMAP, PatternScan, SuperFamily, SignalPHMM, TMHMM, HMMPanther, Gene3D, Phobius, Coils, CDD and SFLD.

### Confirmation of the presence of chemosensory related transcripts

To confirm the presence of the chemosensory transcripts’ in the Varroa RNA, we amplified and sequenced the transcripts using PCR and specific primers. Sets of primers were designed (NCBI primer design tool), based on the open reading frame (ORF) which contained the conserved domain according to hmmscan (HmmerWeb version 2.7.2 (http://hmmer.org/))^38^ (see Supplementary Data S13). PCR amplification was carried out using Dream Taq Green Master mix (Thermo Scientific, Waltham, MA, USA) with cDNA and primers. Purified RNA (500 ng), was used for cDNA synthesis using a RevertAid First Strand cDNA Synthesis Kit (Thermo Scientific, Pittsburgh, PA, USA) following the manufacturer’s recommendations. As a control gene, 18 S ribosomal RNA (18 S) gene of Varroa was used^28^. The PCR reactions were performed using a thermo-cycler machine (Sensquest Lab-Cycler, Gottingen, Germany) in 0.2 ml micro-tubes, each containing 20 μl of total reaction volume, using the following thermal cycling profile: 94 °C for 1 min followed by 35 steps of: 94 °C for 30 s, 56 °C for 30 s and 72 °C for 1 min. After 35 cycles, samples were brought to final extension at 72 °C for 5 min. 10 μl of amplified PCR products were loaded to each well of a 1.4% agarose gel containing 10 μl ethidium bromide. The agarose gel was run until the bromophenol blue front reached the end of the gel (approximately 30 min at 250 volts). The agarose gels were visualized under UV light in a Geldoc (ENDUROTM GDS Gel Documentation System, Labnet, Edison, NJ, USA) and the size of the products was verified to match to the expected length.

### Relative gene expression of Varroa IR-like genes

Specific primers were designed based on the ORF containing the conserved domains in the sequenced PCR products (using NCBI primer design tool (see Supplementary Data S14)). A relative expression study between the mite’s forelegs and other legs was performed using 18 S rRNA gene of Varroa as a normalizing gene^28^. Amplifications were performed using Absolute blue qPCR SYBR Green Rox mix (Thermo Scientific, Waltham, MA, USA). The expression of genes was tested in triplicate of three independent biological experiments. The cycling conditions were as follows: 15 min activation at 95 °C, 40 cycles of 15 s at 95 °C, 30 s at 60 °C and 30 s at 72 °C. A melting ramp from 72 to 95 °C was used with a 1 °C rise at each step and a 5-s interval between steps. A Rotor Gene RG-6 (Corbett Robotics Pty, Brisbane, Australia) with analysis software was used for qPCR data normalization and quantification. Standard curves for each set of primers were done using a mix of all cDNAs. For all qPCR assays a no-template control was included (data not shown).

### Statistical analysis

Real-time PCR data were analyzed using T- test, with significance level set at P < 0.05.

## Electronic supplementary material

Supplementary Information
