# Supplementary material for: Chemosensing of honeybee parasite, Varroa destructor: Transcriptomic analysis
Source: Sci Rep. 2017 Oct 12;7:13091. doi: 10.1038/s41598-017-13167-9 (PMC5638865; doi:10.1038/s41598-017-13167-9)
Supplement: Supplementary file 1 — Supplementary Information [file 41598_2017_13167_MOESM1_ESM.pdf]

Chemosensing of honeybee parasite, *Varroa destructor*: Transcriptomic analysis

Nurit Eliash, Nitin K. Singh, Starlin Thangarajan, Noa Sela, Dena Leshkowitz, Yosi Kamer, Ilia Zaidman, Ada Rafaeli & Victoria Soroker

Data S1. List of Varroa chemosensory transcripts, by conserved domains

OBPs * Conserved domains presence

Pfam		PF01395
InterPro		IPR006170
**Transcript**	**Length (bp)**	
Vd22474	4072	*
Vd14320	2868	*
Vd15084	12397	*
Vd22214	2522	*
Vd18044	4389	*

IGRs

Pfam		PF01094	PF10613	PF00060
InterPro		IPR001828	IPR019594	IPR001320
**Transcript**	**Length (bp)**			
Vd22269	3992	*	*	*
Vd52944	4189	*	*	*
Vd21675	4956	*	*	*
Vd22292	7715	*	*	*
Vd21835	8381	*	*	*
Vd20015	7051	*	*	*
Vd22240	1315		*	*
Vd21758	3164	*	*	*
Vd20855	5670	*	*	*
Vd17150	4310		*	*
Vd18850	2417		*	*
Vd19098	4303		*	*
Vd21743	8234	*	*	*
Vd18951	6474	*	*	*
Vd21106	4056	*	*	*
Vd20731	4898	*	*	*
Vd370	4530	*	*	*

GRs

Pfam		PF08395	PF08395
InterPro		IPR013604	IPR009318
**Transcript**	**Length (bp)**		
Vd7144	1203	*	*
Vd69666	1088	*	*
Vd105872	2631	*	

NPC2

Pfam		PF02221
InterPro		IPR003172
**Transcript**	**Length (bp)**	
Vd21392	1965	*
Vd42649	640	*
Vd69937	2140	*
Vd111865	620	*
Vd25130	2267	*
Vd69836	619	*
Vd74517	938	*
Vd40090	792	*

SNMP

Pfam		PF01130
InterPro		IPR002159
**Transcript**	**Length (bp)**	
Vd11771	4072	*
Vd15725	5002	*
Vd19004	11392	*
Vd19882	3914	*
Vd20660	4082	*
Vd21127	5041	*
Vd21844	3819	*
Vd22104	4577	*

Data S2. Varroa chemosensory transcripts sequences

>Vd22474 len=1895 path=[1719:0-1894]
CACACATCCGAGTGCGAGCTCTCCCTTCTACTATACGATATGCATCAATTTCAGTAGATG
TAATCACAACCAGTTCTCTCTATAGCGAAAATGGTATCTATCTACCGCCCGTCTCTGTGT
TGGCTCGCATTCAACCCGTGTTTTTGGGGTTGTTGTCGTTGTCGTTTCTGGTACCTCTAT
CATCGGCTGACTCTACTGTTGAACACAGCTTAGCACGTTGACGGCCCCACAGCGTACGCA
CGCTTTCCTCAAGCGGTGGAAGCAGCGGCAGCAGTAGTCATAGAACTGACAGTGCAATGA
CGTACGGATTGGGGGCAATGGGCATCGTCTCCCCTTTCCCCTTTTAAGACTACTGCGGCG
GCGGGAGCGTATAACGCGTCACTACCACCGCCATCGCCACCGCCTATGTATCTGATATGT
GTGAGTGTTTGTGGGTGTGCGTGTATCAGGCTGGCTGGCGACGCCATATAAGCCCGCGAT
CATCGCGGCTCTCCTCAACAGAAACAACTGCAGCAACTTGTTGTAGCTTGGAATTTTTAT
TGCGTTTCAGAGTAGCTCTAGCGGATCGTAGACGTTCGAAAGGTCGGGCACCTGTGATTC
TCAGTAGATTCTTTGTGCATTAGCGGCATACACAGCAAAAACATGAAGACGTTTATCGGT
TTGGTGCTCGTGGGCATGGCCATGGCAGCCCCCCCAGCATCTGGCGGCCCCGAGGCCGGA
AAGCCACCTACGATCGAGTGGGGTAAATGCAGCCAGCTAAAGCCTTCCGACAGTGAGCGC
ATGACGAAAGCTGCCGTTGTTGAGAAGTGTGTGCAGTCTCTACCACTTCCCGAGGCTGGT
AAAGCTTCGCAGGCCGAGATTGAGAAGCATCGTGAAGATGTGACCACCTGTGCCCTTAAG
GCTGAAGGGTGGTTCGATGACAAGGGTGTTTACAAATTCGATCGCGCTCGTGATGAGATT
AAGAACAAGAAGCTTGCCAGCGATATTGAGACTCCCGTTTTGGAGAAGCACGCCGAGTGC
CAAAAGGAGGCTGGTGAGAAGCACACGAGTGACTATATCAAACAGGTTCAGCTGTACCAG
GCATGCATGGACTTCAACATCTCGCAAATCTGCGGTATTAAGGTTGTATCTTAATTCATA
TTGGCAGGTTTCTACTAAGTCACTACAAGGCTGACAACTATGTCGACCAAAGAAGTATGA
TTGACTGAGAATATGGACGAATGAATTTCAAAAAGATGAATAGACGAAGGCATGGAGAGC
CAATGACTTTTGCTTGTTAGTCCAACTACAACGACATTTGACATTTTCTAGTAAAGACGA
TAATAATTTTGCACGTCACCGAAAGAATAACTGAATATGACAGTTCCAATAACGACCACA
ACTATAATTACTTCTACATTATTGTAGCAATAAAAATTATAGCAATATTATATGCAAAAG
GTATTTGATCACGAGTCTCGCCCTTGTCTGTGACGCGCCCTATTATATTGATTCCGTTCT
GACCTATGCTATGACTCGGCTCTTAGACTACATCCACCTATTAAAATTCACCATATCGGA
CGCTAGGTGTGTAAAGTCACCTCGGCTGTTACGAAGCGCGAGTGTTTTAGTACTACTCAA
AGCGCTAATATATTACCAGAACTACAGGCCAATTCTCGTGATGCTGATTTCACAGACAGA
TTTACTTTCACACGCGAAATGACCAGAACATGTAAAAACAGAAATTTACAATCTGCCGCA
ATATCTGCTTGCCTGCCTGAATCGGACAATGATTCGCAAAAGCACAACAACAACAATAGC
ACAAGTGAAACCGATCTAGCTCGGTCTAGCAACTTGAAACTATTGAGAAGAGTATGATGA
ATAATTTTGTGAGCAACAATAAAGTAAAATGCTCA

>Vd14320 len=2868 path=[2914:0-673 3587:674-683 70:684-2867]
TTTTTTTAAATATTAATGTAATTTTATTTAGAAGTCGATTATCTACGAAGATGTGCTACC
ATAGGGGTTTGTCGTCTGTCAGTTGCTTCTGTTATTGAAAATAACCGTCCCACATTTAAC
ATTCCCTCGGGAGATTTTGAATAGGGGCTTTGGCTGGCAGTCAGTTCTGTGTTAATATTG
CTAATGCAAGGAGTAAAAATGCAATATTGTTCTTCTGTAGCTTCGTAACATTGAGAGGAC
CTTATTCTTGGTGGATAATCACCATATTCCCACCCGTCAACACTTGCACCAAATTTAAGC
CACTAGGACCACGCTGATGAGCATTCGATTCAGTACCATGAGATCTCCATTCCAGCTAAG
AAAATATCCTCCGCGATTTCCGGTAGATCACATAATTGAGTAGACATTTATCGTTTGTTC
TAACATTTAATATCCACGAATTTATTGACTTTTGTTAAATGTTTTATGGCCTGACGTGTA
AACCCAAAACGTCCGATCTCACAGAATATAGCTCAACTGAAGCACCGGTACATGAGTTGT
TTTATATTCTTATCGACCAAACATTGTCGCTGGAACCACCCTTATTCCAGCAGATAATTT
TGCAGCATAATTTCAATTGCTTCTGCTTACTTTGACGCCAACTTACAATCGACTTCGAGG
TGATCGATGAGACATTTTATACTGCGATCCTCGGAGGTATTCTTATCATCCTTGGGACAA
TTCTCGTAAGCTTTTTTAAGTTCGGGCGAGCCTGCTGAAGCAGCGGTCATTGACATCTTC
TTCAAATCGACCTTGTGATTTTTCAGTAGTCCGTCACTGGTTAACACACAATTAAGTCCT
CGGCGAAATTTGTTCATATCTGTTCCGCCTGGCTTAATAAAACATTCATCAAACACTTCC
CTTTTTTTTGCGGGCAATTTATCTGCTAGACATGTCCTCCATTTTCCGAACTGAGAAGTG
TCGGACGCGTGGTCTCCGGAGGCGGTATCGGCTGAGCAACAGCACGCCAGCATGGCCGTT
ACGATATACACAGCCGCATTCATTATGATTCATGTGCTGGTCTCTATTGCTCGTCCGTTA
GGTAGCGCCTAAGTTGCCTAGGAAGTGCGAAAAATTGCTTTTTATAAATGTGAGGGCGCT
GGCGTCGGTCCGAGGCGCTGCAGAGCAAAGTAAGTCCCTGATCACTGGCTCCGTTTCATA
TGGGCCTCTTCTTTGTTGCGATTGTTAACGACAGCTGTTTTAGATTGACGTTGATTTATC
GACAAGGTCCATTGTGTGTCAGACAGGCAAAAGGATTGAACAGTTACTGCTTTGATATAG
TTGCAGATCTGTGTACGACGATAGTTTATGGCTGTTGTCGTTGTTGCCGTCAAGAGTGAT
ATTTGTCCGGACGACTGAATCATTGTCTAGTGGATGGAGAAAATTTTTATTTGGAACCCC
GATGATCATCCGTTGCATAGCTGATGGTCTAATATGACTCTATTCCTTCTGCCCTTACTA
CTGGAAAGTAAGTGACTATCGCTGGTTTGGCAAAACGGAGTCAGCAGCTGAGATTTGCTT
CAAGAATGTCCTGAGTCGTCGTTGATAACCAATGCTCTGCGGCACGGGTGCATAAGTACG
ATTGGATCAAGCTGAGAAGAGACTGTTTTTGTTCTGCTTTTTCTACGTCACGGCTTCTCG
TAGTCGCTGTTCATCACGAACAGATTCGCCGGACGGTAGAAGAAACGAAGGAATGCGCTG
GTTGCCAGCCTGATAGCTTTGCATCCTCTTCGCCGATGAGTGATGGCGTGACGTCACCAA
ACAAACAACGCCGTATGTCTCAGGAAAAACTCGCTTCGTAGAATGTGTTCACACGTTCCG
AACTCGCTTTTATTATAGCCTAGCGCACCGTCCCTGCAGCTATCATCAGTGATATTAGTA
AGCGCAAGGACACACCGATTAGGAGTGGTTCCGTCCTTGAGCATACCCAAAAAGTAACAA
ACGGCTACTCAATTAGTATGTATCTAATAACAGCAAGGATGTTATGGATGCTATATATTG
TAGTTAGCAAGTCCCAGCACGCATTTTAGCTGATTTTAATCTTTGTCCCAGGTTGATTTC
AACTCTCCTCTTATAAAGTTTTTGAACGACCTTTTCTTGGCAAGATTTACGTCTTAGGTG
TTGATTTCCTTGATTATTAAGGGTCATATTTACGATTCTATTAGGATGCGACACTATACC

AAATCGAATGTTTGCTTAGGTGACAAAAATTACCTCTAGGAGCTTGAACATTTTTCATCG
TCCAGAAAGAACGAGAAATCACTTGAAGGGGACATCGACAAGGCAACTCTTTTTATGCCA
TGTACAGTCGTTAGCATCAACGTCGTAGACCTTTGCAAGATTGGGACACATTATAGATTT
CAGATAATTTCATTATAGATTGAAAATTGAATCAGTTTTCGCCAAATAAAAACGACAGCA
CTTAAGATTGAAGGTAAAGTCGTTTACTATTAGTCAGCTGCTCATCGATGACGAATCCCA
TGATATGCCCTTCACATTGCGTAGAATTCGAGTAGCGTAATCACATGGCCTAGCTCGAAT
TGTCTCTAAAGCAGTACCTTTCATTTTCGCTTAACACCATTTCAATCGAGAGGCTACCCC
CATTAAATATACCGACATAGGAAATAGACTTGTAACATGACTGATAGCATGTTTTGAGAT
GAACGGACCAAAAGTGAAGCATAACAATACGGATGGTTCAAGAGCCACCTAGCAGAATCA
CGCGTTTAACTCAATAACGTTACATGCAATTTACTACTATAAATTACTAATTGAACAGTT
AATAAATGTCACCCTGTAATAATTTGTTTTTTTCTTGCAGCAATGCGG

>Vd15084 len=12397 path=[12508:0-574 104:575-12396]
CAACACAGCAGTACCAAAAGCACTGCCTGTTTATGGATTGGAGGCAACAGCATGCCCTCT
CCCCTTCCGCCGCAATCGACCCACCACTGTATGCTTCTGCCGTAATATAAACGCACGACC
AAAGCGACACTCTTCAGCAGAAGCGCCAGCGCCTCCTTGATTGTGGCTTTCATTACTTTC
TGGAGTAAGAAGTTTAGGACATAAAATAGTCATTTCGGTGTATTAGGGCTTGCAATATCA
AAATTACCAGCATGAAGGTTTTCGTGATCATCGCATTGGTAGGCATGGCTAAGGCCGCCC
CTCAGGCACCTGCTTCCGCCACACCAGCCAAGGTCCCCGTGATTGAATGGGGCAAGTGCG
AGCAGCTGAAACCCTCAGAGAGCGAACGGACTAGCAAAGCTGCAGTAGTGGATAAGTGTT
TGCAATCGCTTCCTCTTCCGGATCCCGAAAAGGCGACACAACAGGAGATTGACAAGCACC
GTGAATCGGTTACAACTTGTGCACTTAAGGCTGAAGGATGGTTCGACGACGAGGGTGTGT
ACAAGTTCGATCGTGCTCGGAACGAAATCAAGAACAAAAAACTCGATAGTGAAGTTGAGG
AGGCTGTTCTGCTGAAGCACGACGCATGTCAGAAGGAGGCAACTGAAAAACACGACGACT
ATATTAACCAAGTTCAGCTATACCAAGCTTGTATGGATTACAACATCTCGCAGATCTGTG
GTATCAAAGTCATGGTTTAGGTCGTTTTTTACTTCCTGACCTACTGCCAGGATCTGCGAA
CCGAGAGACTTCTGCTCAAAGTTTGTGGTATTGCAACCCTCTTGACCAAAGGAATATAAT
TATGCTTGGATGATGATTTTGATTAAGCGCAGGTACACGAAAAGCCAACTGACCTTGCCG
ACTGTTACAAGTACTCAGCAATATCCGACGTCCTGTTAACAGTATCTAAAGTATCTAAAA
GTATTCTTGTTACACTTATTTGTTCATAGAAAATGTAATACTACGAGCAAGCGTAATTGC
CTGTCACTTAAGTTACGTATCAGTCACTTCAGGCGAAGTCCATGTTCACGGCTTTCAAAA
ACCCGCTATCTCGAGCGATTCAATGTTTGCCTTACGACTACCAACGACAATAGCACAATG
TAGTGGAAATTGAAACAGTATTTGATCTCTTAGGTTAGAATTGACTGCGCTGATTACCTT
CGTTCTTCCTATTACATGCATTTAGACTCAAATTGAGCATTCGATTTTATGGACGCACCC
GTTATCAATTCTCTCTAGCGGAAATTTTACATTACACAGTTTGCCTTAAAAAAAAAAACTC
GCTTTCTAATGTACAGAAGACGTTAATTTTTACCAATGGTTAAAGAATTGTCAGTCTGGC
CATTATGCGTAATACCTGATAAATCAAAAGTATATTCGTTAAAGCAGCTACGTGTAACCC
CACAGCGATAGAAATATATGTTCCGGTGGATAAAACTGCTACCGGAAGTAGCAAAAGTAC
ACAAATCATAACTATGAAAATGATGTGAATAAGTCTGAAGGTAATCATCAAGAAAGGTGA
TTATTACGTCAAATCTTACTGTTCGGCTGGCTTATGATTAAGGAATTTCGGCTGGAATCC
TTATATAGGACTTATTTTCCCTTCTTAAACTAAAGAAAGTACAAGTACGAGAAGATTACT
AAACCTGGCGTTAAAAATAACTGACTAAAGTGCTTGTGCAGTTAATAGTGGTATAGGTCT
AATCTATCCTACACTACTGCGGACGAAAAAAATTTCTGCTGCCCTCTAAGGTAACGTAGA
CATGATCTAAAAAATTGCTTTATGGATGCTGCGAATGATCCGATACTGTCTTGTTGATTT
GATCCAATATATTGTACGTTCACATTTCACAGTTATTCAACTAAGTCATATTCATTGAAC
TTACAATACAATTGGCAATTGAATATCTGTATACTACTGTATCGCTACCTAATTAAAGAT
GACTTTTTACAATGCTTTAATTGATCGTGAATTGTGAAAATTCTTTTTATTATTAAAAAA
TGGTTTATACACAGCGTGGCGCGTTCAAGTTTGAGCGCATTTTGGCGGGCGGTAGTAGGC
ATGCGTTTATCTGCTACTAAACGAAAACCCGATCTTGCTAGAACACAATATCAAGCTCGT
GCGTCGTAATCAAAATCCGTGCGAAAATCAGCTATTATCTAATATTTTCATCCTGAATAA
GCCCCTATCAAGACCTCAAAGTTTATTTGATATCAACAGCCTACATAGTTGCCAGTTTCC
ATGCGGTTTTTGAAGAATTAGATTTTTTTTTTTTTCGAAGATTAAAAACTAAATAGTTT
GGAAGTTCTGCCATTTTGTTGATATTGCAAAATCGCTGTATTGCCGAAGTAAACTTACCT
ATCCATATGACTAACTTGACTTAAAAATAAAGTGTATTTTCATTATGCTTAACAAAGTTT
TTGGGTATAGTTAGTGAATTTCGTCGTCAGCTTGAGAAATAGCTAAACGTCAACTATCTG
ACTATCAGTTCTCATTTACTTCTATCCCAGAAGGTTCGTGTCTCCATTTAAATGAAACTA
CATTTTGGGTCTCGAATATTTGTGGTGTATGTGGCTCTTCAATAGCCCTGCATGGAATAT
TATCGAATATTTGCGAGCTTTCAATTTTTAGTAACTTACGACGGTAGTGACATAGAGACT
TGAATTATAATGGTACATATTGTAGTGCAAAGTAATGCTATATAATGATCTGTGTTAATT
TTCCGTCCGCTATTCTTGGTGCTAAAAGAAAAGCAGTTCTTTTCAACAACAAACCTCAAA
TTGATCACATCGGCTGGGGGTTCATCTTTGGTATATACAAGGAAGTCATCTATTGTCAAA
AACCTTTAAGCATAGCGAATAGTACTGAAATTAAACACTTTACATATGTTTTTTTACATA
CCAAGTTTTATGCGGCTTCTAAAAAAAAAAAATTGTCTTCTTCTTCTTCTTCAAAGCGTAT
CAGAAAATTCTCAATACAGAACTCGAATAGCCCAGTTTTTATGATCCCAAACTTAATGCG
AAGATCAGCGGCCGTTTTAGAGATGAGAATATTTGAAAAATTCGAAGAAAATCGAAGTGA
ACGGATGTTAGTCGAATAAAGATAACCGTAAGTATTGTCAGTTCGCACATGTAAAAAAAT
GTGCTTGTTTAGATGTAATTTGGTAACAAATCAATACAAATTCGAACTCCAACAAAGCTC
CCTGTAGTGTTATAGTATGAGTTATTAATAATTAGAAAATTGCAAAAACCAATCGCAATT
ATAACGCATGGCAATGAAATAAACAGTTTGTGCAATTGTAATTAAATACTGCCCGCCTTT
GTATCCACAAAGTTGTCAGCTGTAACACTGTACAACATTAATAAGCATATTATGCATTTT
GCATGACGTTTCAACCTACTGAACGAAAAACATTTTTGAGGCCTTGCTTGCATAAGAATG
GATGTAACTGGATGTAACTAAGATGGACGACTATGGCGATTCTGTTCGAACTACCTAAAA
TTTTGCGTCATGCTTGAGCTCAGCAAAATTAGAGATACTCTATATATATATGATAACGGG
CGATTATTATACAAAAATGCTTTCATTTAGCATTGCATGTCTATGTTGTAGGTAGTTATT

CTGGATAGAAAGAGGAAATCAAGACATATGAACAGGACCCACAGATCCGATCTATCTTTA
TATATAAATATATTTTTCTAAAAAAGAAACATAAGTACGTGTGGGGAACGTAGAAGGGCC
AGGCCGGTGTGTATACATTTTTAGATATAACATGAATCATCTTTGGTACTCTCCAAGCAT
ACTGATTAGTTATATATTATATAAAAATTGCGGTGTAATAGTATATGATCCCTAAGATTT
CTATCTGACTTCAAGAAAAAATATTGGCTTTGGTTTATAACCAACGGTATTACAAAGGGT
AGACAAGTAATATCCGTCTCAGTATAGTATCTCATCCGTAAACGTAACTTTTTCGGAATC
ACTCAATAGAATACGTTTAAAAACTGCCTGTTGGGTCTCAGCTACGTTCTTATGTGGATA
TTTATAACCAATATTGCTTCATCGACAGAAGGGACTTCATTGTACATTTTTTTTTCTTTA
AAAAATCTTTTCTATAAAATTTCAGGCTCAAGTTTTCCGTGATCATACATATACGGTGTT
GAACTGAAAAAGATTTGTTGGTGTTGTGAAGTCTGATAGCTGCATTTTAATCGACACACT
GAAGCAGCGGAAAACACAAGATCCGAATAAAGAATACCATATTAAAAAAGAATGTTTTTG
CTCCTATCTCAGGCCTTATTTCATGCCAGTAATGATTCAAAATGCTTTAATCTGTGTATG
TGGATTATTTACATACCTCTTATCTATGAAAATAGCTAGATGAATTGGTACGTTTTGTCG
ACACGTGTTAGGCCATCCCGCTAATAAAACGTTTTACGATATGAAGGAAACGTGGAGCAA
CTAGTACTACGGCTAAGACAGGGTTCAGTTTTTTTTGTTTATTATCAAAACAATCTGTTT
TTATGTGGTTGAAATTTATCTGTAATGATTATAGTATCGGTAATGATTATAGTAATGATT
AATTAGATTTTATAATAGCTTGGTGGAGTGTGTATATGACAGAATTATTTTACCAATTAT
AGTAGCACTAAACTACGCACTACGAGCTAAACACGAAACAAGTATCTAGTGCTTTCATGA
CGACCACGAAGAGAAATAGCAATACGAGAGTTTACTAAGAAAAAAACATCGGTAAAACCT
TAAATGGACCAAGAGTATGCATACGTTTTCGTAGTTTTCCTTATTTTCTTTCTATTTGTT
GTTTACTGATCACCAAATTTTGCGTATTTCGTTAGAAAGCTGTTTGCTGTACACGATTGA
TGTCATTGCTTTCATTGAATTATAAATCACTATATTCGGTAATTATGCCCTTGTAACATA
ATCGTCCTATCCTCGCTATCAAATCCCAAGCTTTGATACTAAAAATATAGGATAGTAAAT
TAAAGGCGACTAAAATTTGGTATCGGAATAGCGAATTTATCAAAAGTAAGCCGTAAAAAG
CTATAACCTAAAAATAAAAATTGGAAATCTAAACAACATTTGTTTTATTTCAGTCATTTC
TAAAAATGTATAAGCATTACATTTTTCATATATCTATCAGTTTTCCAAAAAGCTAAAGTA
TCAGAGGTATTTAAAAAATAATAATGTAATATGAAGAAACAAGTTTGCTATTCATTGTTT
TCATTACTTTTAAAAATGGCAGACTTGCACTATTGCAGACTGCAGGCCCGATTTAGGGTC
AAATATTTAGTTTCTTTAACCCCTTCGGAGTAAATAGCTTTACTGCTTTTGGGATAAAAC
GCGTATAGGTGTACAAAGCTCTCGACAATCGTGGTGAAGTCACACAGCAAGCCCAATTCT
CACAACGTAGTTCAGGCCAGATTCAGGTTTTGATAATTAGTGTGTTGATTGCACAAACGC
CATCTGCAGTATTCGTAAATGTATTAAATAAATTTAGGTCTTTATCGTTTATACGTCGTA
ATATGTTAGATATTGACATATATCACGCATCAATTATGATACTACGTTTTGAAGGCAGCA
TGAACGAGTCCTTTTTGTAGATCCCAGGACGACATTTTCTGAAAACAAACTTGAGCTATA
CATGGTTTAAACTTCTTACGCTTTGTTTCTAAACGTGTAATTTCGGAACAAGCAAATTCT
TCAACGAAACTGTTTGTGCATACTTCTAACATTGATGTTATTAAATAAAAGCACCATTCA
ATGCGATTACAGCTTTGATTCGAAAACTAAATCACTACTTTAAAATATTTTTTTTTAGTC
TGTGAGTAACATTTCAATAGTAACCGTATGTTAAACGATATCGATAGCTATAGTCTCTCA
GCAAAATACGCTACATACAAATAAAATTATATTTTGCACTGATAGCAAACCGAAATTCTC
AAAAATGCGGCCATAACTTTCTGCATCGAAAACCTTTGCATATCAAAGTTTCGATTAATT
TTCGAGTCTTTTAAATCTAAATTCTATTAGGGACCCGCCTCCAAGGTAAACTTTTATGAA
GAAACCTTGAGCATGAAAATTGGCGATAACTTAGACTGTCGATTTCGAAGTTTTTAATTT
TGATGAGTACTCGTCCAGCGCAGAAGAATCGGATAATCGCCAATATTTGCTCAACTTTTC
AAAAGGATGCACCGTTCGCCGTGTTTTAATCTAAAAATAACTGCTTTTGTCTAATACCTG
GAGCATCGAATGACCAGCTCGCCAAATATTTTCAACAAATATTTTTCCCTGTTTTAACGC
ACCAGCTTGTATTTCACTTACTGCTTCGCCAATGTGCACCATCCTGTTTCGTCATCGCCT
CCATCTTGGGGTGACCGACTTGAATAAATTTTATATATGTAGAGTCCTGGAACACTTGAG
AGTACGCCACAAGCCTTTGATTTTAAGCACTTTCTTGTTTTGACTTTCTCTTGAGACTCA
GCTTAAGGCGACTGTGGATAAGAAGAGTAACTCAATCAATAAGCTCAATTTGGTTCCACC
CAGTAGTATTTGTATACCAGGATAATTGACCTTGACTAAAATTCAATTACTTTTTTTAAT
TCTGAGAAAGCTATACAGTTTGTCAGTTTTTAATAATATTTTGGTTACTTTACGCAATAA
ATACAAATTCACCATATTGCTAACAAGTTTGTAAAAGGCATGGTTTTTGAGTGCCTGTTC
TCCAGGGTCCTCTAAAGAATTCTTCGATTATGGATAAGTACAGAGACAACATAAACCGAA
TTAATTCGTTGTATTAAGTGGTAGCTCGATGGCGTGGAGGCCGAGGCTTTCGTTCGCTTG
ATGGTCTTGAAAGGTGTGTTTTGGTGTCCCCCAGATTCATCATTGTTCCGCTGAGCATTG
GTCCGGAGCCCATTAGAGGGAAATATGAGTGACTTCCTGCTATGGTGTGTCTACGTTCGA
GGCGAGATTTCTTACGTCGAGCGCCGCCGTTTTCTGTTCCACTTCGATTACCTTGATCGT
CTAGCTGAACAATTGATTTGAGTATTTTGGACGAATTTGGGCTATAAACAACACCATTCC
GATTACTTCGATTAGCGTGTTGCTGCTGTCCAAGATTTCGACCACTTCTAGCTCGAAGCA
CTACCATTTCTCCGTCACCTTTACTACTAATGCCAGTGGCTAGTAACGATTTTCCTATTA
TCAATCTCTCTTCAGCATCGTTATTGCCATCGACTGGCACGGTTTCTTCTTCAGCTGAGT
CGCACCTTTCTCCGTTCGGCACGTGACCGCCAGTAGAACTAACGGAAATTGGAACTGCAC
CACTCGGCGTTACCGGGTAGAATCCAGAGACAGAATCGACCATATTCTGGTTCTCCTTGT
CTATGTTATCATCGCTGTTATCCCTCGTAAGCAAAGTAGCCTGGCGTTTGACCTCCTGCC
ATATTCTTTCAGTTTGTTCCCGGCTCATGCGTTGCTCCTGAATCCAGTTTCCAGTCCCTG
AAACTATAACCTGGCTCCTGAAGGCCTTTTTCTCTAGAACAGCCCGTGTTTCTTCTTCAA
AAATCTTAGCTTTCTCAGCCGCCAGCACACTTCTCTCATGGTAAGACAAACACATGGGAT
TATTATATGCCTCGCATGTTCTTGGAGATTCGATAGGATCAGTTTGGCCCAGTTCATGGC
CTACTCCGGATCGCTGCCGGTGTCTATCCTTATGCACCTGTCGCTTGTATTGCGTAGCAA
GTTTACTTGGGATTGCTGTTTCAATAGTAACTGATGATGATGGTGATGTATAGGCTGATT
CCTCAATCTTTGATAACATGCAGGATTTCGTTAAAGTTGTACTAAGCGCAGTCTCTTCGA
GCACTGGAGGCTTGCCGCATTCGAACGAATGAATTTTTTGCTTAACGGAGAGTATTGGCG
CTGGTGTACCTGGTAGTAACGAGAGTTTTGTTGACAATGGTGATCCCGACTTGTATAGAC
TTGTACCATTGTCCATAATGGAAATCGAATTCGCTGGCGATGATGAAAGCAATGGTCTCG

TATCAACAAGTGCAGGCAGAGTAGGAGTCGATGTAGGATCCTCTGTAAAGAGATAGTCCC
CGTAGTTGACAAATGTCTCAGTAAGGTGACACTGATGACGCATATCAGATATCATCGATA
ACATTGAACTCTGGGCCACCCCTATTCCTGACCCAGATCTTACAAGTGTTGGTCCTAACA
CGATGCTGAGATTACGCGAGTCCATCTTATTGGTTTCTGCACGAGCAGCAACACGTTGTA
GATGAGAACAGAAAAATCTCAAAGTTCCCATATGGATTGGCGGTAACTTGTGTACTAAAG
AATGAAGAACGGCAAGGCGATGTCCACAGTCTGGAATCTTGTCGGCTGCTATAAAATGGT
GATATAATGTTCCAGTTAGCAGTGGCTCCGGTAATAGCCGGAAAAATGATTTCAGAAGAG
AGCTAACCACATTTACGTCCGCCCATCGCGGATCGTCTAATTTTACATTAATTGGCACAC
TAGTGCCACCTTTCTCCTTCGTCAAACTCTGGTTGTTAACAGTTGCCATAAGATGCTGTA
CGGCGGCTGAATTACCCGGTACTCGGTAAATGCCAAGCACATCTAAACCGCGTTTTTCTA
TAATCGAACAACAATGAACCACAAGCCAGGGCACCAGCGGATTATCGACAGAAGGAGGGC
AATGATCAAGCGGTACACCAATCGACGATCCTTTGTGATCACCTTTGTGCATGACAGCTC
CTTCTACTTCTCGGCCATCCAATAGAATATTTGGCTCATCGCCCATCGATGCTCGTCGAG
ACTTCGACCTTTGCGTTGCGGTGACTTCCGGTGGTTTCCTTAACATTCCAGTAGTTTGCT
GTTGCGTTGAGCTACCTGAGTGTGCCCACGAACTCGTTGCGCACGATGGTTGATGATTTT
TAAGAAAACGTCGGTCGAAGGCGGCTGCACGCTCAATTACAGAAGGCCCGACATGCAGTA
CTCCACTAGCATCATTGTTCTCCGCGTGTATCACACTGCAAATACTGTTCATAGTCGCAG
ATGTATCACATTGCACTAAAAACTCATACTCAGCATCACTTGAAGCTCTTGATGCGAGAA
GCTTCCCGTCTTGTGAACAGAATGAACATTCTTTTAGTGGATATTCGCTCTTTGTAGAAG
GGAACACCGTTCTTCCAACAATGAAGTGCAGCACGTTCTTGCGTCGTTTCGCCAAAGTAG
CTGACGCAAGGGATACGCGCACTGAGCCAGAGGCCAAATCGCACAATAGCTTATTTACAA
CTACGTTTATAGACTCCTTATCCGTGACAATCCTCAAAATGGAATTTCCATTGCCCCTTT
TCTCGACATGTACCCCAGCTGGACGCCAGCTGTGGTCGAATCTCTTGCCATTTTCGGGGA
AACGTAATATCTTGAGAGCGCAGCTAGTGCCACCATGGCTCATGGAACTTTTGTCTGCAA
AAAGTGTCTTCAGTTTCTTAAGAGACACTCCAGGTGGGGGAGAAGTCGTACCACTTTTCT
TATCCCCGCCAGTGCCAATTTTTTCATCCTTAACAGTGGAACTCTGCCAATTTGCTCTGG
TATCTTCATGAACAGAAACCTTTTTATTGATTCTTCGGCTACAGGTGTGATGTATGGTTT
CATCAGCGCCGCGAGCACTTACTATGACACTCTTTTCAAATTGCGCTGTGCGAGTCTGCA
CCGTATTCGGTGGTGGAGAAGTCATTCTTGTTAACTCACTGCGGTACAATTTAAGTTTCT
CCATTGACCGAGCAAGAAAACCCTGACCTCCACCTTCAAATAACTGCAATTTGGCAGCTA
CGGACACTTGTAGACCAGGTGACGGTGAATCAAGGGAGCCAAGTGATGAGGAACGGCTTC
CTTCTCCTCGACGAGCTGCCGCCATTTCAGTTGACAACCGTTTGTCCTTTTTCGCCTTTA
TTTTTTTATTTTTCTGTCCCCTACGGTTAGGTAGTGGTCCTCTTTCAGGGAGTATTCTCC
AATTATTTTCATCTTTCCCAGTAGCTAAGTGACTAGGTACAGAAACAGACGGTGGCATAG
GACCAGCACAGCTAGATGTTTTGGAAGAGCTTGACGAAGGCAAAGGTGTTGAGGTAGTCA
AAGGTATTGCAGGGAGGCTGAGGGACAGCTCACTTTCCGCACCGGAAATGCTGCCACTCG
TTGTTACACTTTCGTTTAATGATACGGTACTTTGGTTGATAGGTGAACTATGTACAAGCT
GCAACGACTTTAACTCCCGCTGCTGCTCTTCCCTATAGAAATATGAACCTATGCTGGCCA
AAGTTTTTCCTCGAGGGTACGAGCTGAGGTAGGGTCGAGGTGCTAGGGAGCTGCTTAGCC
AGTGGGATCGCTGCACAGGAATGACGTTGAATCCAGCGCCAATCCCAGCAGAGGCCAGCC
CGGTGCCTCCAGTAGTGTCCATCGAACCCAATGAAGGCGCAGGGCAGCTAATCACCGAAC
GAGACAGGCCCCCGGAAGGTACAGCACAGATTCCACCCCCGGCTCCCGCCGCCAGCAGGC
CGCTTCGAAGCAGAGGGTCATCCAGTACCGACACCACGACTAACTCCAGTACCGATGAGC
ACTGGTGTATGTGGGCGACGAATTTAGCGTAACTCTCGATGGGCGACCCCCGGTGAGCAA
CGATCCGATCGTGGGGTCGAAGACCAGCTGCCTGAGCAGCCCCACACTCTCGAACTTCAC
GAACAACCACCGCATCTAACGGCTTTCCTTCCACCCACCAGGGCTCGATATCTTTTGGCC
GTTTGCCCAAGGAGGTGGCAATCTTGTGAATGTGAGAGGGATAGGCCACAACGTTACGCA
ATGTAAAACCAAAGCCCTCTGGACGTCTTTCGATCGAAATCGGTCGCGAATGGGGCCAAC
GGCGCTGTTCTTCACACGGACCAAGCACTAGTACCCCATCGGCATCGTCATCAGGGCCAA
CGCAGTAATTTAGTCCACTCTTTAGCCGGGTCTCAACTCGTATTTCCCCTATTAACTTCA
AATCTTTATCATTGCAAATGTCACTTTGTTCAGCTCCTACTTTTTTTTGTTCTCCTTGTT
TTGCAATCAGTGATTTCTTCAGAGAGTCAGCGTCCTCATTGATGTCGCCTGTTTTTGCTT
CTGCGTTGCATGCATCCTGTTCCACACTTGTAGGTTCTTCAAATCCAGTGACTTCCTCGC
TTACGCTATCATAAGAGTAATTACCTTGCACGACACCGACACCACCACGCTTACTAATCA
AATCACAAGTTAATCCATCCGTTCGGTGTGTAATTTCAAGAACTGCTGATGCGTTCTCAG
TAATTGCATTTGTTGCAAAACTGCTCTCATGTGAAGAACTCTGTTCTGCTTCGGCACAGC
CTAATATTTGCGGAGGGCAATGATACTTTTGAGATTTCATGGTTTCGTCAGCTGGCTTGT
CGTCATCTGATGATGTAATCCTTAGATAATCTTTCGTATTGAAAGGGATCTCAGTAGAGA
GCTTTGCATGCACCTGAATCTTGCAGACGTCGTCACAGCGAACAGCGGAACTGGCCCCAA
ATAACTGATGACTTTTATATTCGGGATATGACAATGGATTGCTTTGGCGGTTCGACAATA
ATGGCTCCACAAAACTGGATGTTGATTGTAGATCTCCAGCGTCCTTAACGGCATAGTGGT
CCATCTTCCACCACCTCACGAAATCCGAAGACGTCTCCTGCTCTCTAGTTTCATCTTAAC
AGCTTTGGAATCTTGACATAGAACATTCTTCTTGATACCTGAAGATTCTTAACAAAGCGC
GAGATAAATTAGTCCTTAGATATTTGCAACCCACATATCATACTTATGCTCTGTTTTCAC
TACATATGAAAATGTGGAGCACTTATTTTTCAAAGAATTTTCATATTTCTTGCGCGGCAA
AACTGCAAAGTGGCTCATAAAACCGTCGGTAATACAATATCACTCACATTGACTTTTTCA
TTAAACTGTAATACACAGTATCATTCAACATAACCAATGCTCGACTGCAATAAGGCCAGC
CGCACGACTGATGGTTTTAAAATAAATTCAGTTCTTTGAATCATACTCGCAATTTTTTAC
ATATGTAACACAGATTTCCCTTCCACCAATTTTTAAGTTCGTGACTTTGCTGCAACACTG
AAGCAAGCACAACATCGCACATATGGGCTCCTTACTACTCCGAACACCCATAAATAAATA
ATCATCAATAAAATGAGAAAAGTCACTTAATCCCTCCCTGGAACGGCGGGCACTAAATTC
CATAAGATTACCACCTATTCTAATCATAACAATGTCG

>Vd22214 len=2522 path=[13559:0-1210 16002:1211-1549 19101:1550-1551 16343:1552-2182 102:2183-2521]

AAAGACATCGTTACGTTTTCAACAGAAAGTAGTTTTTAAATGGAATGGTACCTCGATGTT
ATTGTTCACACATCGCAGTTTATTTCTTAAACGCTTTTAGACTGATACTGTTATTATTAT
CACACTCCCTGTGTCAACCCTTATCTGCTTCAGGTGAACATACAAAATGACGTCGACCTT
CAAAGGTCCGGAAGTTTCAGCTAGCCAAGACATTTACGTGTGTTTAATTGTACCAGACGA
GCAACGAGATGAGCCAATCGTTAACTCTAATTGCACGCTATACTCGTGAATCAAAACACA
CAATACCGTACGCAAAACCGGCTGAGACCACTAGCAGAATCAATCTTACACGCTTAAATA
TCAATCCGTCGACTCAGAATAACCTCAAACTTCCTACGTAATATCTAAATGCCCGTATCT
AAGTGAACCATCAAAATTTATACTATCAGATTCACTTATTCGTGGAAAGGCAGCCGCTTG
AAAACAAACACTTAGACAAAGTGGTCAAGACACTTCATCAGACCTTAGTATCGGACGCGA
AGTTCTTCGAGCTGCGCTGAAAGTCGACTATACACGATCGCCTTTACAAAGAAGCTGAAA
CATTCAACGGATTTGATTACCAAGCACTCTCGTTAGACATCGTCATGATGATTCCTCCCT
GGTTTCAACGCAATTCTGGCCTGCCATACATAATTGTTATTCTTCTCGGAATAACCTTAG
AGGCTCAATTTGGATTCTCCGAAACCGTCAACATGGACCAATACAAGTTCCACATGTCGA
AGGTTCTTTCCTTGTACATGAATGGCTTCAATGTTCACCAACTTGGCACCATTCTCGTTT
CGATGGCGTACTCGTTTTCACCCGCTGAGGATCGCCCTGTCGTCGAGCAGTGTCTGGGTC
AGCATGACAATGAAACTGTAACGGAGCTGATCAAATGTTATATGGAGAAGACGAACATGA
TGGATTCTGATAAGAAATGCAATCTGACGAAACTGAAGGAGTTCATAACGGTAAAGATGG
CAGCGGCTCCGCCGATAGCCGTTGCAGTACTACAAAAGTACGCTGAATGCGTATGTCAGA
TATCAGGAGCTGAGGGAATGATTTGCGCTCAACAAAAAACAATGGATTATGTCAGGACGC
TCTGCAGCGCCATAGTCGGCGACGAATGAACGGCGAGCTTCGCTACCTCTGAAATCTACT
TTATTTTGACCGTATGTGTTGCCAACGTACGCGTTCTTGACGAATTCAATCTAACTAAAC
CGCTTGTACAACGGTGAAAAAATTTCGTTTGGTATTCTCTTTGGAATAGCAAAACGAGGT
TCTCCTGCACGCTTTGTAACCTGATGTCGTTAGTAATTTTATCAAGTTCAACAGTGAACG
CTTCAAGAGCGAGGAGTCTCAATGTTGGCCAGTGCAATCGACTAGTCTGAGCAATTCTAC
AATAGACTTCCTGTTTGCGCCTGAGACTGTCGCATTTCTTTCTGAACGAGCTATGCAAAT
GGATTTAGAATCTAATTAGCTAGCCGTTGCTTCAACTGAAACTCACCAATAGGTCGGTAT
GCTACATTATTCCCAGCTAAGAAAATGCAAACTGGAAAGCTAAATAGGAATACAGATCGA
ACAAGCTATTCATATTGTCGTTGGAAAGGCTTTTCACAGCTGTGTTCGCAGGTGGCCGAT
TTTTTTCCAAAAGCAAAGCAAAGCAGCTTTACTCAAAGCTAGCAGTAGAAAATGTACATC
TATGAATTTTAGATTCTTTCAAAATACAGTTGTCAGATGGATCCGCTACCGTAAACATAC
AACGCTACCAGATAAAATACAAGAAATTAAGATGTATCTCTTTCGAACGGCAACAAGTTA
TTTTCTTGACGATATCTTTTACTTAACTACACTCGAAGCTGAACGTTACTTTCTTTTTTT
TTATGAAATTTATTCACCAATCAACGTTTACAAAGTCCAAAATGACAGCATGTATGTATA
CAGCCAGGATGATAATAACAGCATGTTTGAATGTGCTGAAACCTGTAGTTAAGCATGATT
CTAGTACAAATAGTTATTCATGGCAACTTCTTATCGATGCTCATTATCGGTGTCTGTGTC
GATGATGTATTGTTTTCCAAACGATGTGTTATGTCAAAACAATCGTCAACGTTATTTTCC
GCAACGGCTATAATAATATACTGCCTTAGTTTAAGTTTACATATATTTGCGTTCCTCATA
TTAAGCTGAGCTGTGCTCTTATCATACTCAGTTCTTCTAATTTCTCTTAAGATTAGCATC
GATGGCATTCGGCTGGAATAACATAACCAAAATTGTATACAGCTTACCGTTTTATGGAAA
ATAGAAATTTGAAATAAGCGAAACTGCGACTGTTGTTTTTCTCCTTCTTCTTCTTTACCA
TAACTTCAACAAAGAAGCAGTTGTCTTCTCATACAGCTCCACCTTTCCATTATTTAATAA
CTTTCGCGTAGTCTTCACACATTGTGTAGCACGTTCGAAATATAGTATATATATATATAT
AT

>Vd18044 len=4389 path=[5777:0-1921 12961:1922-2175 @5776@!:2176-2706 7699:2707-4388]
CCTTGGATTCTACTGTCCTTTTGCCGCAAATTCTATACTCATCTATTTGTTAATAACATG
ACAGTTCGTCCGCTGTAATAACTACTAAGTGTGTGCAAAAACTATCGGGTGCAAAACAAA
ATCAAAGTATAGATAGAAGGAATAATTTCGCGGATATTAGCAAATGATTACCGTCCATTG
TTTGTATTGGCACTGCACAAGCAGAAACGATATCGGCCTTATTAATTCGCTCTACGCACA
TACTACTCAGAGATACCTACGTGTTGTAGATCTATAGCAGTCGCACGCAATCGATTCATC
AAAATGTTTAAGACAGTCCAAACATTGAGATACCTATACGGACTAGAGACGTTCCTGCTG
CCGTCACAATACCAATCTAATATCGCATATAACATAGTATTATCGATATCCGTCGACGCT
GTAGCGTTCGTTATCTCCTTTCGCGTACGAAAATGACGTCAATGATTTTGTGCGATCATG
ACGTATCGTTGCGGTCGAGATGATGCTACCAATCTAGGAAATCCACTACTTCGACAGCAG
CATTACAAACCTGTAAAACAAACCGCCTCTGTTATGGTTATTTTGTTGATGAGGATTATT
ACTCTAACGATATCGATTCGACATATTCGATGTATCCTTTTATAAATTTGGTCGTCTGAC
TATGGCGGCACTGGCTGCAATCGCAGGCCAGAGTGGTACGGAAGGCCTGTTCTGGCGTAC
GCTGAGATGTTACCCGTCTCCTATTCACTCTCAGTCCATTCGATGGATCAGGGCGGCCCA
TCAACACTGCGGACTGGTGGCTGTTACCGCTACGACGAACGCCGACCAGCCCTATATACA
TACATATATTACGAAGCCGCCAATCGATGGCGGCGACTAGCACAACGACGACGATGGAGA
AGACGCGGGCCGTCTGTGGTATCAGCTTTTTCGTCACCAGCAGAAGTGGCAATAATAGGA
TCGCTGCTGGCACAACGTCTGCAGCCCTGGGTGCCAGCTCTTGACCCCAACAAATGCCCC
CTCGTAGCGGCCGTCAGTCGCGTCAGTTTCGTTCGACAGGTCGACTGCTGCTGCCCAAAC
TGCCCATTGCTGCACTCGTCGCAGGCGACTATGAAACAACGACGAACAAGATGCTGGCCG
CCCTTACGCGATTTTCGCAGTGCGCGCCGCCTCTGTGGGACGTCGTCTAGCATTGCTTTG
GATTATTGCAATCGCAGCTACTGGGCTTCCATCAGTTCCCAGTCATGCCGAAGCTGTCTG
GATGGATGCCTGGCTGACTCCTGTCACTCAGTCAATCGTTGCTTGTTCGCCTGGGTCAGC
TATCGCGTACAGCAAACAAGCTGCAAGTTGCTATCGAGCTGCAGTGGCGTTCGGCTCTAC
TCCCACTCCGCTCGACTGCAGAAGACCCTGCAGACCCAGACGTCGACGGTAACCGGCAGC
AGCTGTTGCTATTGGCTCGTATCTGGTTGATCGTTTGCTTCGCGCAGCAGCGCCCTCCTC
CTTTTGCACGGTGCGAGTGGTTTGCCTACAAAAGCCCTGCATAGCTCGGCACCTGTCGAA
CGAACAGCCAGCTGTCCGCCGTCTAAGGGGGCAGCCCAAAGCGACCCAGAGCAGTTACAC
TCACTATTACTGCTCGGTGCAGTTGAGTTGTTAAGAATTCGGTCTATTCAAACAAATCCG
AAATCGCCTCAAGTCTCGAAATTGTTGTTGGGTGCTAGACGGAACACAGCTTATCAGTGC

CTGCTTGATCAGTTCATGGTTGTTTTCTTTTGCAAGTAGTGATTATTACTAGTAAAGTTC
AAGTTGTAAGGATGCATCGCAGCTTTGTACTTCAGTGCTGTGGCTTGTTGTTCATCGTCG
GACTCGCCCTCGCAGGACCTCTGGCCAAGGACAAGTCCGAGAGGATCACTCCCAAACAAA
CCGCAACGGACAACAACAATTTTGAGAAATCCATTGGAACGTTTCGATCACTGCTCGAAT
AGCTAAGCTTATTACGGGTTATTGACTAACTATAATTACGGTAACACAGGCGAACACGAT
TGGATCATTTAGATAAATTGAAAAATTTGTTAGAACCCAAAACGATACGATCGTTTGAAC
TATAACAATGACACTAATAAACTTGGGTAACATAATTATAGTATTAAGAATCTAACTTTC
AACTTATCTCTTTCAGCTACTTGATGATTTCTACAACACATCAACATTGGATAGCAATTT
AGAATAGGAATTCAGGCCGAGCTTGAACGTCCATGCCGACAGAATATTTAAACTCTTAAA
ACGTTATTCTAAGTTTGGTATTAATAACACGCTTCCACATCATAACTAATTGCTATTTTT
GCTACCACTCCACATTGGCTTTCTGCGTAGGCAGCAGACAAATCCGATTAGACGCAAAAA
TATTTGCTTGGCAAAATCATACCTGAAATAATTTTCGTCATATTTTTTGAAAACAGGTTC
CAAGTGAATAGAGAAAAACAAACAACAGGCGTGCTAAATTTTGTACGTTGGTGTCAGCTA
TAGTTTTTGCATAAACACGATACTCACTATTGCCATAAAATCTGTATAATATAACAGACA
TAATATGAGAATTCCTTTAAGGATTTTAAGGCTTCTTTTCCAAGTATATTGCCTGTAGTG
AAAATGATTGAGCATCGACGCACCGATGATATCCTGAACGAAACATCGCGATTGCATCGA
CGATGCTGTCGAGGAGTTCTTTACATCAAACTGGGGCGAATGCGAGCCGCTGAAGAACCC
TTTGGTCCTTCCTGAAATCAGTGACGCTGCCACATGGGAACGCTGCCGTGTTCATATCCC
ACTTTCGCCAGATCAAGTCGAGGAAGCTTTGCCTAACCTTAGCAGATGTATGTTGAAGGT
CATCGGTTGGGTTCGTGCCAATGGAAACATGAACTTGACGAAGTATATGGATTACCTTTC
GCAGGCTGGACTTGACGACAAAACCCTGGAGCTGACGAAGACTGCTCACGAGAAATGTTC
GCAGCGTGCTCTGTCGGACGGTTCTGGCGATTACAAGGAGCAGGATAAAATATACGTGGA
GTGCATCTTCAGGCACTTTGATAAAACGTGCCCGCCCAACTTCCAGGCCGCTGTAAAGCA
GGGTTACATTGTTGGCATTACCCAAATCTCTTTTGTGCCGCGTTACGACGAAGTTGAAGT
AGAGAGTGATACGGACGCTGAAACTGAGGGTCAGGCTGGACCTAGTGTTGCTGACGCAAC
CGGTGAGGCGGCCGCCAACGACAACGCCAATGGCAGCGGCGGCCCGCAGTAATTCTAAGT
CACAAATGAGCAAGCAGAGCGGGTCGATAGTATCAATAGATAGGGCTGCACCACCCCCTT
ACGAAACACTCCTGATAAAAATGCATGAGTGGATAGAAATTCTTGGAGTTTACTTTGAAT
GGTCAAATACACATCTGAGTATTGGCTGATAGATAGGAACAAAGCCTAGATCTGTTGAAT
TTGTTTTACTGGGGGGCGTTCAACCCCGGGGGTTAACCCCTCTAACACCGACGTAACTGT
ACAAAGTACAACCAATGCTGAGGTGAGACCGCAGGGTAGCGAGACTGACAAGCAGTTACC
ATTGTCGTTTATCAACGCGAAACACTGAATCTAGCAAATATTCATCTAGCTAGTATCATC
TAGTAGATACCGAGACGTACTTTTGGCTGTATTTTAATACGAGCAAACAGTAATCACCCA
AGCTCATTCAGTCAATCTAGCCCATCCAGACGTTCTGACTTGCTATAGGGATCTCATATC
CATATTTTTCACATTTATTCTTCTGTGCTCCGATAACGGACTCTGTTCAAAAGGCAGCAC
CTAGTTAACAAGATTTCAGAAACAAATGTACTTGAAAAAATAGAGCTACATAATGTGTGT
AATGCAAATTTACGCGCGCAGGGATCGTACATTAGTAATACGGTAATAACTCACCGACGT
TAAAGCCTTAAATGTTCAAAAGGCTTTAAAAAAGATAACCCGAGGGATACTCTGCGGTGT
GGAGCGTGTACGTGCGCTGCACGCCTTTCCGTCGGTGAATTAGAACTCATCGTTTTAAAA
GGTGGTAAAATTATTGTGTTTGCTGTATTCGGTTTGCCAAGCCTGCACTATAGCCAGGTA
CCAACCCATCATTACAAAGAGATCTGTGATACGAACGAAAACATCCTACTCCACACTCTG
CATGAAGTATATACACAAAGATAAACCGATTATCGCTCACTTCGAAAAAAAACTACGTAT
AAACTATATATAACTGAAAAGTGCATCTGTGAAATTTACATAGACGACATATTAAGCGAT
CGTTATCAGGCGAAACAGTTAATTCATTAAATCAGCAAGATTTTTCCATTAGACCCGATA
AAAAAGATC

>Vd69937 len=2140 path=[1:0-2139]
ATATTTATTAGTCAAAACTCAGCATGTTATGATAGATTCGTCGTTTAATGACAGTTCACG
CTCTTTAAATTGCTAACCTGCTATGATAAACTGCGTTGAATGAGATGAGCGTTGCTACTT
TTTATAAATCGATCATCAATAATATATAAAGTTTACCGACCTGAGTCCGATTACATAAAA
TAATCAAAATCTTATTGGAATCAGAAGGTGTTATAAGCTTTGTTCAATCATTTTGAATTT
GCTTAACTGTTGACAGGAAAAATTCATTAAAAAAATACATTAGATTTATAATAATCTCTT
TACCGCTTACGAGGGGTTGCGGTAAAGAGATTATTATAAATTGTGCTGAGATAGTTAACA
ATTAAATAACGTATTCTATATAATTAAAGAAATATATTTCTATAAAGTTAGATACAAATA
TAGTTATGAAGTTAGTAGTTGTTAACACTTCATTTCCACTGCAGGAATATTTAATATGCA
TATATTAGAAGTATTATTTTTGCTACCAGCAGTTGATACTTTTTATTCGGAAATGATGAA
AAGAAGCATTGCCACGTTATCATTCGTACAGATTCATGCATGCTTTTGTATAGTTAATGC
ATATTACAGTAACAGATTTTTGTCCTGTACGTTGCTCTTCTATACTCACGGCCTGCGTCA
GGCCGAGATAAGTACATAAAATATATCCGAAAGATTTAATCAAAGAATTTTTCTTAGAAA
ATTAAATTTGTCGACCCAATCGCCTATTCGTCATCCTCTTGATTGTCTACGTTGGCTGGT
TCCGTTGAGGCGTTAGGGTCATCGATAAGTCTAATCTTCATCGAGAAGCAACCTAGCTTT
TCTTCGGCCTCGGACTTCGCGTCATCGTCGATGAAGCGGACGAAGGCCGTAGTCTCAATA
TTTGGGAACACGCTGAGAATTGGTAGAACCATTGTGGTGGTGTACTCTTGGCCGGCTGTC
TGCGGACAGAAGTTTTCATTGCCAGGGGTTTTGCACACATAACGCTTAAATCCTGGGAAG
GGTAACTCTGCTTCGCCAACGAATCCAGTGATTAGCACCTTAACCACTTGGGCGCTTCGT
GGTGGAACATAAGTGACTCTTAGGAGATGATTGTGTCCCCGGATCAACTCACAGAGGTCC
TCTTTTGTACAGTTTTCTACTTCAACAGACTTGATTGTCGATAAACCATCGCACTGCTGT
ACGTTCTGGACCTTGTAGAGTTCTCCGGCATACGCCGCAGAGGCTATTGCAGCCAGCATG
ACAAATCTAAGCATTTTGTTAATTAATGATTTGTTCGTCAAAAGAACACTAACTCAATTA
ATGGCAACAGAGATCCTCTCTCAAGTCAAATTGAATGAAGGCGTTTAACAGGTAACTTCA
GTAACGACGATAATCATAGCAGCACCAGCAGCAATCTACAAATGGCCGAGCTGTCGACGA
TGGACGTCACTTTCACACGCGAAGAGAACGCCTGGCTCACCAGTAACCGTGTACTATATA
AGCCGCTGCGGCAATACCCTTCGCTGTTCGCTGCAGACGCACGACGTCGTGTTTTGACAC
TTTTGACCGCGATGGTAGCAGCGTCATCCCTCGATTAGACACGCGGCCATGTCCACTTTA

CGGGATTCATTGATGGCAAAAAACAGAAAGGCGAAAAAGAAACGGTTATCGATTCTCTAT
CGTTGCTTCTGCTACGAGCAGACGGATATTCAGCGAACTGCGAAAGTGAATTAGTAGCGT
CGAGCAAGCAAATCGATAGCTTGAGCATTGGTGATACGCCAAACGGAGAGGGGGGCAGCT
TGAAGCAGTCGAGAGAAAGGAGTCTATGTGACAATATTAAGGGGAGTCGAGTCCCTTGAT
CGAAGCCGGCGTCGTTGGAGGCGAGGGCGCGTACATACATTCACGATGAACCAACAGTAA
TAGCGATCGGTCGAAGACAACGAGATCACTCGATCATCTAAAAAGAATTAGATGAACAGA
GTCTTAATACAGAGTTACAATTCGGTCAGAAAGTGATGGGCGACAAACGTACAGATAGAT
TCTTGCCTATTAAGGGCCTCGAGAACCGAGCAAGAAACGGAACGATTTAAAGAAGATTAT
ACTGCTAACACAACGTTGAGGGCGCTTAGGAACGGTTAGG

>Vd69836 len=619 path=[597:0-618]
TTTTTTTATCTCTAGATAATAAAATCTATCGCATCTTTATTGCTCACAAACATCACAATC
AGAGCAGTGAAATAAAATGGTGGCCGTTCTTTTTGACCACATGTCGAGCAAATGATTAAC
GACGTCTTATTAATATCTACTGAATTACGACGGGAAACCTGGCGCAGATGATTCTATTTT
TTCTCTCGTCGAAGAGAGTCCATATGACATCGAGCGCAATAGAAGGAAAGCCGTTCCGGA
CCGGGAACGAGTGTTTGTAGGTAGCGGTTTCACCATTTTTCAGCGGGCAAATGAGTCCTC
TAGCGCAAGCGTTGCTGTTTGGCAGCGGATAAGGGATGCCAAATCCGGAGATGAGGCCGT
AAACTTCGACGGTGACCCGGGAAGCATCGCTGGCCGGTACGAACGTTATGCTAAGGTTGA
CATTAGTACCCTTATACAAGACGCAATATGGGTCGGTATCCTTGCAGCCGCTGATTTCTA
CCGTGCGGATAGTGCCCTGACTATGATTACAAGAAGTAAACGGACTAGCAATAGCGATGG
CCGCTAAAGCGAAAATTACTAGGAACGTTTTCATGGCTAGAAAGCAACACTGAAATCAAC
TTGAAACGACTAATGGTCG

>Vd21392 len=1965 path=[1607:0-405 22781:406-407 2015:408-512 2120:513-629 14129:630-999 2237:1000-1163 2401:1164-1270 2508:1271-1348 2586:1349-1501 2739:1502-1964]
CTCGTATGGTAGTTGTTTATTTAAAAATTAAAATAAATATTTCTATATTCTACAATTTAT
TCGGGTATCTAATTTATTACATGAGAGCAGCGCGGATACTATTCCGTTAGATATATCCTT
CCTTTAGAGCAATAGAAACTTCGTGCTAACCCTAAAGTTAGCTTCAATCCATTTAATAAA
AAAAATACTTGTCGCACGCGTATCTGGCAGCATAATTCTGAGTTCTAAAAATTCCAGGTG
TATAAAACATTATATTTAATGAACACGATTGCAACCTAATGAAAATAGTAACAGAAATAT
GCCGTTTTAAACCCTACTGCAAATGCTGATTGTCATTTCGGCTTTCAAATTATGGGACTT
CTCCCCTCAGCAGTTACTTAATCCACCACTGAACACCACTTTTCAGGTAACTAACGGAGC
TTTTGGTCGAGATTTAACGAAGTTTCTTAGAAAGGAAGTTCAGGAGGCGATCGCGGTCAT
CCGGGCCCTCCACAGACAATTTGAACCTAACGCAAAAAAGTGGTCGGCCGTTGGTCGAGG
CTTTCAAGATGACTTGATAATCGCCGGAAGTTAGCCATGACGGAAGGACGTCGGGAACAT
CGTACGCCTTATTATCTGTTCGATAGATACCCTGTAAGCGGACAACAGAGAACAACTACT
TAGAAGCCGTTTGAGAATATGTGAATCTAAGGCTCTAAATCCTGATAGGCTGTATTGTGG
GCCTACTGGTCATCAAAACGTGCCTTTTTTAAGCTGACCAACTAGTATCTAGTAATGAAG
TCACTGAAATTTGTATAACATAAAGGTGTTAAAGAGTGTTGCTTATCTGATGGGACGGCT
TAAAACATGGCCTATCTCATCTAAGCCGAAAATTGCCGAGATTGCCCAGAATGACATAGC
AAGAACGTATGTTCTAATGTAATATCATTACGAAATCTAAATAGATTAGGGGCAACAGGG
GGAACGTTAAAGATGTTAGGTCATTAGATAGTCGATGTAACCTTTTTAATCGGGCACCCG
CAAGGCAGGCCGAGTAGCTCGTAGAATACAGGACACTTGATGGTTGAGTCGCAGATGTTC
TCGTACACACATGAGCCGTATTGAGCGACGCAGGGGAGGTCCATCCAAAAATAGACCATA
CGACGTCGCATGCGGACGTGGACCGTTATAGGCTCTGTAATATCCTCGAGAACGTTAATG
TCCGAAGTGATACGTATTCTTCCGGGGAAAATGAGGGGATCAGGCTGAACTCGTAGGTTT
GAGTACTTCACAATGGCGTTCCGTTCATAGGGGCAGCTGGTCACGTGATAACTACGAATG
TAGTCGTCGTCCTCTTCGTCAAAGACCCAATTTGGATTGTCTTCTTCATTGTTTATATAT
CGACTGGGAACTTGCGGCATCGAGATGAGATCCATGTACCTGTCAAGGGTAACGTTTGCT
AGAGTACGATAGAATTCTTGGTATTCCGGCACCGCTGGACTGGCCTTTTGTAAAACATTG
CCTTTTTTTGGGCCGGCATAAATGCGTGTCGCCACGAGAGCCAAAATAACGGCAACAACA
CAATATGCACGACTCCCAGACATATCTCCAGGAGTGACCTACAGTTGGCCAATGTGCGCG
ATCTTTAGTCCATAAGGACTTTCTTAAACACAGCTCTATACACAGCAGCGTCGTGTGGCC
AACGGAAGGCCAATAAGAGACACTTAGGCACCTATACGGAGATTGTGTTTCGTTTTTTTT
TTTCTCTGTATTTGCTATTTTAGGACAACTGGCCGTTGACTGTCCAGCTTCCCCAATTGT
AAACACTGTCTTAACGCGCTATTGAAGTGCTATACGTTCCAATTTGCTTTGCTTTGCTTT
GGTAATAGCTAGCGTATTCGCGCCGTTGCATAGCACTGCGTCCAGCAAAAAAAAGATGTC
AGGTGCTACGACTGCGGCCAGCGGCCAGTCAACCATTAGACCGCA

>Vd42649 len=640 path=[618:0-639]
AAAATCCGTTCGTATCCCAGGATTAAGTACACCTAATATTTTTTATGTTTTTCTATTCTG
TATGCTTCAATCAGCTACAGGCAACAACAAAGTCCTGCTGCTGTGCCTTATTGAGTCTCG
ACAAGCAAATTGTTCTTATCGTTGCTGTTGTGCTTGGTAGAAATGTGCATCATTAAACTG
ATGTTTCGTTTCGCCGTATTTTCCATTGTCTTGATGGCCGCAATCACCACAGCACAAAAT
GAAGTACGAACAACTCCCTGCGCCAACCCTGGAAAGGGCAAGCTAAGGATGTTGACTGCG
ACGCCATGTCATTCAGACCCCTGCGAGATTAAGAAGGGCGGAGTGACGACATTCGATTTC
GTATTTGTTCCCGACATGACCAGTACCACGTTCAGGGTCGACGCTTACGTGAGCATAATG
GGTTTCACTATCCGTATTCCTGGAATTGAACCAGATCTCTGCAAAGCAGCAGTACGATGC
CCGATCAACAAAGACGAACCCGTGATAGGCGGTTTCAATATGTCCGTACCGAAAGTGCCA
ATTCGACGAACCGTCGTCGAAGTGAAGATCACGGGAGATCATGGAATGATGTCCTGTTTT
ACCCATAGTATTCTGCTCGCGTAGATCTAGCCTGAACGAG

>Vd111865 len=620 path=[1:0-619]
GCAAATAGACGGCATGTCGATGAACTGTTTATAATTTTGGCGGTGCGTACATATCCTAAC

TGCTTTTGCTTTGCTACTTTTGCTTAATGATTACAGCCATCAATTCATCGTCTGCTGAGT
GGGAGATCCGAGCTTTCGTAAACGGTCGTCCATATGGTCATCCTGAGCCTGTATCCGATG
CCTGCCTATTCGTTAATTCAACATCGCCTAATGCGTTCTCTAGTAGCCATCGAACGCCAT
TGAATTCAGATCAGTTTAAAAGCGGACCCTCGACGGCTTACCGACAGAAATCGTTTCTTC
CAATGTGCCCACTATTTGCGGGAAAGATCTACCGTTTTTCAAACGTTTTCTACGTCCACG
ATCAGTTTCCCAAGGTGGCGACAACGATACAGTATCTTCTGAGGGGCGATAATGACGAAA
TGATAGCTTGCGCCAGATTGCATATTCTAATGTCCTCGTAAGCGCTAAGCGTTCCATGCT
GGCTACCTTCTACAAGTGTAAGCTTGTACAGGTCGAAATCTCTGGCAGCAGATAGGTAAA
GGTTCCGCTTCGATTCCCGTTTCCAAATTCACTACCGATTCTTGATAAAAGAAAAAAAGA
AACACAGGAGTTGCTCGTCT

>Vd40090 len=792 path=[1:0-791]
CGGAACTGAAAGTAAAAAACAATTTATTGTAGCAGAATGTACGTTCCGCGTCTCTTTCAA
CATATTTATATTATATGAACACTATTATTCGCGTATACTAAGCGTGCTAAATGAACGATA
CACAATATCAACTTATGCAATATATTTGCGTTTAATACACTTGTGATTCAGCCAGGATGC
AGTCCGAAGTAGATCACTTTTTATCACTAATAATGATTTTTGTCCTCACACAGAATTGTA
GTACGTTCTCGGAAAATATCATAAAAACCACTTCCGTTTTTATGTTTGGGTATACGTTGA
AAATAGGAATCTCTATCTCGGCATCACGAAGTTCATTGGCCTTAATCGGACACTGCACAT
GTTTCCCGCAGAGCTCCTGATCAACGCCAGTCAGCGTCACGTTCATTTTATCCACAATAA
CAGAAGGTTGTACGCGAACGCTATTGGCATCGTGGCTAAAGGATCCGATGAAATGAACAT
TGACTTTGCTGCCTTTGGCAAATTCGCAGGGTTCCGACTCACACGGCACGACCGTGATAT
TCTCGATATGTCCCGTTCCTCCGCAATTAACGTACGAGTCGAGCTGCTTCGCATTATTTT
CATTCATCATTAGCATCACAGCGAATACGACCGTAAACAGATCCAGCAACATCATACTTT
CTAACTCTAATCCAAACTTGAAGAACTTCACAAAAGCTGAAATTTATTCTTGTAACAACG
CTGATCGTTTATTCCTATTCCGTAATCTTTCTCAAATAGCTAACTACAGGTTCGTGTAAT
CTACTGCCGAAG

>Vd25130 len=2267 path=[2245:0-2266]
TGCCTGTTATCATTCTACCCTTAGCACACATGTTTCGTTCTGTCTCTCAAGTCGACGGAA
TACAAGAGACTAACAATATGGTCTAGGGCAAGAACGCATACGATACCAATAAACCAGACA
GAACTATATAACATTAAAAGCGTTACTCTGTTGCAAAATGATAAAGCCTGATAGTGAGCA
ACATTCATTTCTTTTACTTTTTCTTTAAAAAAAAAAATACATGCAGTTAGGTATGATTTGG
TTAAGTAGATGTGGCTGGTAGCCTTATATGAGGCACACAAAACGAATGAGCAAAACCCTT
TTAAATAATTTGCTTTTGTGAAATAAGTAGTTCTAAGCTTACCGTTGTATTCTCTTCAAA
GCCAATAACTTTTGTAGTCAGATTACTATAGCAGATTTTGAAGCATTGATTATCTAAATC
GATCGATCGATCAAACGTGGGCCGTACATATGTTAAAATTGCTACCGGATAACTATATTA
TCATTAGTATATAGATGAAAATTATAATACGTCATTATTATATACTATTGTTATATGAAA
CTAAATAAGAGTACCCGACTTGTTTTGGTCTCCTGAAAAATTAACTGAGGGTCAATCGAT
TTGGCAACAAATAACGCTGAGTTTTTATTATTATTAGAATCGCCGCAGAAAATGGTGAGC
GCCCTTTTTGATGTATAAAACACTAATTGCTAGTAGCAGTGGCGTTTTGACTTTTTGTTT
TAAAATTAGGCCGAGTACACAAAATAGGAACTAAAACATATTCATACACCCGTGTGATCC
GCAATCCTGTATTTTAACGTCGACCATGCCGTCCAAACTCAAAAGTTATAATCTCTTGCA
AGCTTCATATGGTCAAACGGTAAATCATATGCAAATGACTGCGTGACAATTTCGAAGTGT
GCTGCCCTATTTCGAAAACTCCCTGGCACTTGCGCTTTTTATAACAACTGTTATACAATT
ATGATATTAGTGTTGAAAAAGCAATTACACAAACAATATAGTAATTTTTGCCAAAACAAA
AGCTGGACGATACAAAGCCACAGTCCTTTGCTTTTTTCAGGTGTTGGACAGTACCGAAAT
ACGGACACCACGCATGCTAGCTGTTCACATCCGCCTCGAAATCTGCATCACCATTATTGA
AGTCTGGTAGGGATCAAAGCACAAATGAGTTCGTTGTTGAAGCTGTCTACGAAGGTCCAT
TTGATAATGAGAAAAATTGAAGGAAAGACCTTCGGCACTGGGAAGGTTTGTGTGTATGTA
GCCGGAACGCCCTTCTCAAGCGGACATTCTAAGCCGGATTTTATACAAGCGTTGCTGTGG
GGTATCGGATACGGCACAGGAATGTTTTCTATAACTCCGAACACTTTAACAGTAATGTTG
TTCATCTCTACCTCGGGTGTGAACGTTAAGGCAATTTTAGTATCCACCCCTCTTTTGAGG
ACGCAAACATTTTCAGTATTGGGACAGCCTTCAATATCAAGTGTCAATATCGTTCCTTTG
GTTTCTATCTTCTCGCACGATTGGAATATACTGCCATTTCCAACAGTTGCCAAAGCAGCT
ACTGTCACCCATACGTAGAGCCACATGTTGGACTACTTTCACTTGAGATTTTGCAGAGAT
TTGATTTTGTCTCCGTCGAATTTCTAACTGCTTGCAGATCCTACAATGGCGAGATGTTAA
ATGGGGCGGCTATCCATTATTTAGCCAATGCTACATAGGCACGCCTCGGATGCACGTCAC
GTCAAGCCAACAGCGCACAGACGGGGACGTTTCGTTAATCCGCAACACCTATCGTGACAG
AGAATCGTACACTGCGTGTGAATGGTTCATAAACCATCATTTGAAAAAGAAAGATCCGGA
GAGCTGGCTAGCACGCACGCACGCTACAGCTACTTGAGATGATATTAACGAAAGTCAAGA
GGCTCTTACGGGAGAATTTGTAGTTGTGAACGACACTACGCATGCACTCGCCACTTTGTT
TATCATGAACCGCAACCTCACCACTTAGAAAAGAAAATATGGTTCCTTTTTGGTTACACG
TCATTGATCGACACTTGACATTGATTACGTGCCCGGATTCAATAGCAGTGCGGTGTATAC
CGATTAATCCGCTGACGACGTGGGACAGGAGCTCGGATCTGATGTGTAAAGTGGTGCTAG
AAAAGTTTCAGACCCGACTGCAATTAAATTGTCTCCCGTGCCGCTTCTATGTCTGGCCGT
GGCCGCCCGCAGAACACCGGCGAGCGATAAGCGTCAGACCACATGGA

>Vd74517 len=938 path=[916:0-937]
TGCAAACCCGACATTGTTATCATGATGAGTATTTGAAGTTCAAGGTCTTACTAATCCTCG
GCCGGACAACTCTAGCAGCGATGTAGGACGAGATTTTTGATTGAAGCCAGATTAGATGAA
TACTGCGTTGACCCCCTTACTAGCTAGGAACAAACAGCGAAATCGTCCGTAAAATTGTGA
TCGAGGATACTACGACGAGTGAAATGCCTCGGAGAAATTACAACTATATAAGGGATGTGA
CCGAACTTAATGATAGTCTTGCCTTCGTGTGCAGTTGATGGAAACCATGAAAGTGCTCGT
CGTGTTCAGTATCCTGGCTATCGGCGTCTACGCGCAGAGAGTTACCTTTAACGAATGTGG

CTCGAACGAGTATTTTACGCTGAAACAAGTGGACCTCAAGGATAAAATTACTTTTCCTTC
AATTGTACGCGGCGATGTACATGCGGTGGTCAACAAAGACATCCCAGCCGACGCTACGAT
TTCATTTCAGGTGTACAAAATCATAAACATCTTTGGTGAGCGTAGAATTAAAATTCCATG
CGTCGGAATATACGGTTCATGTGACTATAATCTGTGCAAATTCATCGCGAAAAAAACGGA
CCCGATCGAGCCACGCTTCGACATTTGCGTGCTATGGCCAGAGGGTATGCAATGCGGGTG
TCCAGTCTCCGAAGGAATGGTTTTCAAAAAGCAGAATGCTAAACTGCTCTTCCCTCAACT
TTCAACAATCGTCAAGCTTCTGAACAAGGGCAAATACGTACTCAACATTCAGTTAAGGGA
CGCTCAGCGACAGAGCTTGTACTGCGTCGACTTCAACGTCCATATTGGCTGAAGGGACAA
TAGGTAGCAGGAAGTGATTACAGGTCAATATTATACATCTATGAAAGAAAATGTATTCGT
GATAGCTAGCAAATAAACAGAGTGAAAATGCTTGAAAA

>Vd7144 len=1203 path=[1181:0-469 1651:470-1202]
GTCTGTTTGGCGTTTGGATTTACGTAATTTATCGCATTCTCCTTGATACGCTCGACTTCC
ACGTGGAGCGTCGTCTCCAGCTGTACGTTGAGAGCGAACTAATGATAGCGTTTTCTCAAA
TACCAAAGGTACTTTACCCCGTAATTTCTTTTGTGGCTCAATGCGACATAATTATCAATC
GGATTCTTGTTAGCGGTGGAATGTCCTTCGTAATTGTCGTCTACGTATTCGTGAACCTGT
TAATGAATGTGAAGTTCGAAAAGTGGGATACCGATATGGCGGAGTGCGTCGAACGCTCAA
GATTGGATAAAGCTTTTCCGCTGGCGCTTCGGGTAGGCGAGCTCAGAACGCGACACGCAA
AGCTCTGTGAAGTCGTTCAACGTCTCGATGGCATATTCTGCTCATTGGCGTTTGTCTGGC
ACACATCGATCGTCGTTAGTGCATGCATGGAAGCCACAACCATGATTCAGATGACTAAGC
GTCGATTTGAACTATTTCGTTTCCTTGGAAGTATGGTTAATACGTGTTACGTGTGTTTCT
CCCTCTTTTTGATAACCGGTTCCGCAGCACTCCTCTGTGAAGCGCATAGTAGTAGTCGCC
ATGTTGTCAATGCCATTCATATCGCTGACCAAGGATATACGAGCAGGGCTCTCGTGCATG
AGGTCCGATTTATCCTAAGCCAGATGTCGCAGCGCGTGGAAATGACGGCCTGGCGTTTCT
ACAAGTTAGATCGAAGCGCGATCCTACAGACGTTTGGTGCCTGCGTTACTTATGTGAGCC
TCGTTATACAGAGCGCGGAAGATATACTTGATTAGTAGTAAAGCACAGAACGAGGCAAAC
TTTGTGAGACGATAGCCTCAGAGCCGCAAAGCGTGCGCAGTAATCAATCTTATCATGGTA
GCGATTCAATCGTCTGCCCGATCCGATAGTAACAAGACAGCCAACACTGGCAGTAATGGT
AGAGACTCGTCTCAGCGTCTGACGGTTTACGATCCGGCATTTCGAGTCAGACCATTTCTT
AGAGCCGTGTCAAACATTAAACTATCTTTCCTTGCTAAAGGGTGACGAGTGGTTTTTTTG
TTAGGCCAGTGGCAAAGAAATTTTGCCAGCTTATTCGTCATTGCGATCATTCGCAATGAC
AAATAAGCTCTGTTGATACGAAATAAATACAGATATATATACATCTGCAGGTATAAACAT
GAG

>Vd69666 len=1088 path=[1:0-1087]
GACAATTTTTGATTTTTAGTTTTCAATTTGATACCAAAATTAAGGTTTCTTTCTTACCAA
GTCCATATCGCTGAGTTAATAAAGCTACGATAATATTAAATAGCTAGTATTAAACTGAGA
TATATGACTGGTATCGTGGATCTATTATTATATTTGCTTAACTTCCAGCGTCTCCTGGTC
AACAAAAACAACATATTCACCTTTTGTTGCACTAACTGGTCCTCAACAAGGAAAAGGGCT
TTTTTCATAAATTATATGTAATTAATACGCAACGATTGACCAATCTGTATATGCTCTTCA
AAGTATCTTTTCCGGCTTTACCAGGTACCACTCCGTGTCGTTATATTATTGAGCACGGTA
CTCGCCGTAGCATTATATAAGCTTGCCATTATTTTGATATCTGAGGCCACTGTAACGTCT
TTAACTTTCGACGGTGCCATCTGAATAATGAGGAGAATATAAGAGATCAGAGCTCCGCCG
ATTGTTAGAATGAAGTTGCGCGAAAACGTGAAGAAGTTCCAGCCGCTCATGGCAATTTCC
GGCTTATTCATCTGCGCGACTAGTAGATGGGCCTGCGAAAAGAGCTCGAGATTTTCCTCC
GTTGGCGCGTGGTTGATCATCCGATGTACAAGCATCGTGCATGCATGTGCAGTTTCAGTT
AGTTTGGCGCCAAAAATCGATAAAGATATGAAGAGCATAAAGAAATTCATTAGGTTTACG
AAGAGCTGCAGCAGCTCATTTGGATCGTCAAAATCTGGATCGGACGCCTTTCCATTGTAG
TACCTGGTAACCTCAAGGCAGACACCAATCACGAAGAGGCCAGTCCAGAAGAAGGCCATT
TTTTGAAAAAGATCGTCCGATTTTAAGACTGTTTTGCTCAAGTTGGATTGTAATCGGCGC
ACAGCCTGAAGCTCGACGAGACTCATCATACAGACACGGTACTGCCTGTCACAGTTAACA
GCCATGGAGGTGTTAGGTGGTTGTAGTTAGTTATCGTAGTAACAAATCGTAGTACAAAAA
AAAAAGTAAAAAATGAGCCAGTATTATACAACAGAATAATTTCGAATGACTAATTACATG
AAATATTT

>Vd105872 len=2631 path=[2609:0-2630]
GTTAAATTCAAGACTAACCACTGGTCTATTGTTTTACCTCACCTCAAAGGATATTGCAAT
GAGGACACTTAATATGAGAAGCGAAAGTTGGCTGCGGCTGTACTCCGGATGGTAGGAAAG
CCTCCACAAGTGCTTCTAGTCTCGTTTCGCCTTGGTCTGCTATTCATAACGAGTTCACGG
TCAACTTACACTAATTGTGCATTTCAGAGGTCTCGCCTTGACGAACCGCGCAAGCTACAG
GTTCGTTTCATCTCATGGATAATTTTAAAGTGTGTAGGGACTCGGTAACGATATTTCGCG
CGAGAAAGTATTCAGACGTCTACCGATACCTTGTATTCGGGGGCCGTTATGTGGGCCTCT
TTTGGAAAAATAAAATGATAGCTTGGACAATCAGAATTATTCACCTGGTCTTGCAGATTG
GCCTTATCGCTACTTCCGGATTCCACTATTTTCAGATTGAGGCCCCAGAGGGTCTGGACC
TAAAGTCCAAGAATAACCCGTTGCATGCAGAAAAGTTCTTCATGATGCAGATAGCACTTC
GAGTGTCTTTCGCCCTCATTACGTACGTCACGGGCATCGTTAAGGAGCGGAGACTTGAAG
AACTACGTCAGATTTTAACGTGTCTGTACGTGGACGATAAACTACTTCGTATCAATCGCT
TGTTGGTAATGTTCGTTATAGTGTTTCTTGCATCGTCGCTATCGATAGATATCTATCTTC
TCTGTGACCGCGGCGACGTCTTGCCCCTTCTCGGATCCGTTCATAACTTTCCGATTCGGA
TGACAAGCAAGTTGATCTACATCGCGTCAACATCGGCAGTACTTTGTATTCTACCAACGT
TCTTCGTAGCATCTTGCGTCTGTGTTCGGCGTGCGATCGGCGCCATCAATGACGATGTCA
CTCGAGCTCTCTACGCGCAGGTGGACGAAAAGCGTAAAGATGAGCTATTTTTGCGACTTC
GTGTTCGTTATGTTCAATTGCTGGACACACTTGACGAGCTATCTGATGTCTTTGGCCTTC
AACTTTTTGCCTGGTTATCGCTGATGTTCTTCCACACTTGTGTCAAGGTCTTCCTTTACG

CTAAATACAAGGACAATGTTCATTCAAATCAAACGGATCACATTGAATACATAATCGTTT
CGTCGGTGTTCGATATACTGCTCTTTTCAATGTTCTGTTACGCCGGTCATGGAGTGTTCA
AAGAGCGCGACAATACCCAGACATTGACCAGACGCTCCGAGTTGACGCCGTGCACACAAC
TTTTGCTCTTTCAGATGGACCTGCTAAACTCGGATGTAGATTTTGGGGTACGAACGGCAT
CTGGTCACAACATGAATTTGCAGTTAGCTTTTACGTTAGTCGGAACGGTCACTACATTTG
CTATCGTTATAATCCAAATTTACATGTCTTCTTCTTCCGTGCCGTTTGCAAACAACATGA
CCTTGACCACGATAGCCAACGCGGTCGGGATTTCCTCGCCCCCAGCTACTTCATAATTTA
CAATGGCGTCTTTGTATCAATAAGATCATTTTTATACATACGGTCTGGACTTCTGGAGTC
GAAGCTGGTCTGACACGGTCTCGAGTTCGTCGTCGTGGAAGTAGAGTGTTGTTCAGATGG
GGGGCCGAATGTCGCAGAAAATCTGTAGACATGTATATCTGAGCTTGGCAACAATCATTT
CCCTCAAACATAGCCAGCTGAAATCATCCTAACATACATAATAGATGATTGGCAAGGACT
GAGTCGGTCACTCCGAAATGGGCAAACATATTTAGGGGGGCAAGTGGAATCATTAATTCG
TCTGCAAATCAGTAAACGAAATAAATAATACTAGCAACTGCAAAAGTATAGATAGCTGCT
TTCTGTAAAACGAGAACGACTACGGATTGCCAATGTAAGAATGAGCAGCTCGAAAGAACT
GCAAGCTCCACGCTCTCGTAGATAGCAAATAACTGTAGAAGGGCAGCAGTGACACGTGAA
GGTCAGGCAACTGGGTAAAACACTATCTAGTAGTTATTGAATGTTGTATTTTTCGCAGTC
TGTTCCGTATTCAACAAGAGCTAGGTGTGCCTTGCGCTTTTTGTTAATGCCTCATGGGCT
TCTCGGCGTCATTCCATTTAAGATTCTGTATTGGATCTATCATACAGGATATAAGCTCCA
CATTGTATATTCGCTAAATACGATGCGCTAAACAAAAGGTATCTTCGCAAAGAACTTTGT
TGTTCATTGTCATTACGGCAGTAAAAGAACTTGGATGACTGACAGTTATTTTGAATGTAA
ATTACAGATACTATCTATGCCGCATCGGAAGCAACTTCTTTAAATCCCAAGTCAATTAAA
TTGATTCTCACATAATAATTTTTTAAGTTTTGTTTCGGCATGCTACAGGTGAAACAATAG
CTTGCAAACCAATACGTGGGGCAGTTGGGGTGTCTGAACTCTACGATACGCAGGGCAAAT
ATTTGTTGATCATTTGACAACGATGTACATTTTACATCTATCGAAGTAGCAAAGTATACG
AATATTATTTTAATTATTTTTACACTGATTCGCTAAACTAGGTATATAAATGAAATCAAA
ATTGCGACAAACGAGCTACGTTGTGAAAATAAATTAAAATATATAGCAAAA

>Vd17150 len=4310 path=[921:0-1315 @870@!:1316-3740 4661:3741-3784 4704:3785-3925 895:3926-4309]
TTTAGACAAAGTTATAGGAACTTGGTGTAATTCTGATGTTTAGACTAGTCATTTGTTTTT
TTTGGCGCAATTCATTTGCTGTGTCTTGACCCAGTTGAAGATCATCGGATTTCGTACTTT
AGATTTGCCTCTACGATCGTATTAGCTGATATATTATCAATTTAAAACTGGACAAAAATC
TTCTCGAATATAGTGTAGTGTAAAGGTACATGTTTTGCTTTATTTAGCGTGTAAAACGCA
TCAGGCCTTAAAATGTGGGCCTTTTGTATACTTTGAATCACTATGTATGTTTTCTAACGA
CCCCATCGAAGTTGAGGCGCGTACGTTTAGCGTTGTCAAGTTAGCCACTAACTTACAACA
GCTGAATAAAACATTGTGTATTCACAGACCATTGTGAACAATTACAGCGCTACTAGGGGC
TATCTATTATGAAATTTATTTTTATTATTGGATGATGGGCTTTATTTCTAAAACAAAAAA
AAAAGCAAAACAATGGAAGCGTAATAACATCAAAAGATGTCGAATTTTGATCAAAATTGT
GCCGATCAGGATATCCAATGCACCCACCGCGTACTAACCAAAAAGTTGACGAATTTTAAA
AATAATGTGGATTGTTCTTATATAAACGTCACTAGCTAAAGGAGATTGCACACTGATCCT
TTTAAAACTGTATAGTAGCATTTTATATTACAGGACACATTTAGTACTTAGCCAGGTCAG
TGACAATCAGTAAGCATCATTTATATTGAAGTTTGCGCTTAATATCAATTGGATCCGATT
CAGACTGAAACCCTCTCACCCCTAGTTAATTAATCCCCTGTTTAATTGACCAAGTCAGAT
TGAAATTTATAAATTTAAATAAATAAGTGTGTTTGAGACGTGCCATTTACGTTAATACTC
GATGCCATATGATAGATATTTTAGAAACGCGCACACAGTCACCTAGCTTATGCGGTCTCA
AAAACTTCAAGTAAGCACACCGGTTATACGCCCAATCCGGTTCGACCCGCTGTCAGGGAG
CTTGCCCCTAAAAAAAACTGCTATTCGATAATTCTAAAATTCCTATGTCTTAGGTAGGTG
ACCAAGGTTGACTGCGCTTTCCTCCGGAATAAGCGTACATCACGGTCGGTCTCGGGCCAC
AGATCTTTCATCACTGAGCCACTTAACGGCTATTCTTGTAGTAAGTTGAACGTCCGTGTA
ATAAGTTCTAGTTGCACATTAGTAGCAATGCAGGTAAGCCCCATTGACTTTAGACAGCTA
CCACCGTATCGTTTTAGCGACAATCTTCGTCCATCAATCGAAAGTTCACATCGAACCCTC
AATATCTGTCCATCAAATCTTGGTCCTTTCGGTGGTGATCAGTCGATGGCAATTCATACT
CCTGAAACGGTTAAGTGATCATCATTCTCCGAAATAGCGAGCAAGAACTTGACTGCTAGT
GAAGAAAAAAGGCCTCTTTCTCGTCCGAAATGTGCTGAATCTATTGATGACGGCAAGGAC
CTTCTACTTCTTTTGCGGCATATGGTCCTTTACTGAAACGACCTTTGAGCGGGGCTTCTT
ATTCTTATACCAGAAGTACTCAAACACCAATGTGATGCAAGCCATCACCACGCCAATGAA
GATCACGATGAACACGCCACCGATATTTTTGATAGAGATACCATCGTTGTTTTTTCCTTC
ATCATCACAGTTTTTCTTTTCGGGGTTTCCATTCCACCACGCTTCCTTGAGGCTTTCTAA
TTTACGCTGGTTCAAAAGCTTCAGAATGGCGCTTGAAAGTTGATCACGAAGTGGAGAATT
CTGCTGTACAGCAAGCGCCAGCGGTTTTCGCGAGAACTCTGAGCCTATCTGGGTAAGGTC
GCAGTTGGTCATTGTTGCGTACTTGATCTGTGTCGCATCAGCAATGAATGCGAAACCGTC
GCTTCCCTGACTATTTTTGACGCGGGCTACGCCTTCCTCGAAAGTCAGGGGCAGACTAGC
TTCCTGCATAGTCCACCAAATTTTAGTATATTTGTCACTGACTGGATAGTCCCATACGGC
CAACTCAGCCCTCTGATCGTCAGTGAGCGTGTCATCGAGAGACATGTTTTTCCAAATCTC
ATAGAATTTCTCTTCGATGTTAGCCATTCGGTGGAAATAGGTAGAGGCTGTCGTTTGACC
TTGGGGAGCGTACTTGACCTTATACTGTTTAGAAAGGTCATCCAAAGATTCGATTGGCGA
CTCCAGACGGGACACAGTGAGGAAAGCGGCCAAATTGGCGGTGTACGAAGCAATTATGAT
GAAACCAAATAACCACCAGGTCGCTGCAACCAGTCGACCAGATAAATTTCGTGGAGCTTC
ACCACCACCCTGAGGGGTCAGCGATGTCATGCAGAACCACAGGCACTCCTTCAGGGTAAA
CTCACGTTTCTCGTCATCGTCTTTGTATTTTTCCTTGTTGTTTCTATAGCTGTACGGCGA
TAGTCTATCGAAAACGAACATCAGAAACGATGTAAAGAAGTAGGCAGCCAAGATGCAGCC
CCACACATTTGTCTCGAGTACAGTAAGAAACTTGAATAGCGAGGGTTTAATCTCGGGTTT
TTTCATCAGAATTGACAGACCGACAAGGTCATAATAAGGCACCGTGAAGTCAACCACTGT
CTCGCGTTCCGCCATGACCGCGATCGGTCCAAGGGCGATTTCCGCTTTCTTTTTTACGAG
CTCGCCAATAAGTCCATTCCACAGGCCGGTCTCCTTGTCTTTCGATCCAAAGTGCTTGTC

CTCAACAGCGTAAATATTATACTCAAATTTGAGAATCTTCTTGATTTCGTCAATCAGGTC
GATACAATAACCGTAAAACTCTGGGCCTGATCCGTTGCCTTTATAAACGAACGGAGGTTG
GATTAGAGTGACCACTTTGTAGAAAGTCACCGATCCGAATTGTTCCAGTGCGGTCTGATC
AGCTTTGGTCCACATCGCCGCTTTCGCATCACCCCCATACACGTAAAAGCCTTTTTCGTT
GTCTTGTCGAGTTTTAGACGCCGTGTAGTTAAAGTGCGCCACCTTCATATTAATGGCTTG
ATAGCCGGGTTTGTTTCCCGCATCAGTGAAGTAAATGTCACCGTAGCGGGTCGAAAGCTT
CGATGACTCCAATGCGTTTAATAGGTTATAATCATCTCGTGTGGCGGGTTTCTCACGACA
CGATGCCCTAATAGGCTGCTTCGAATACTTGTTATCCGCGAAAATACGGGTTATCATGTA
GTAGGTAATTTCCTCATAGAACAATCCGTCCAGCTTCTCACTGCCGTACGCGCCATCGAT
TCCAATACGCTTTCCTTCCTTATCAGCAGGGTGGACAAAGAGTACTTCATGGTTCATACA
ATCCTCACAGACCGGTTCGCCTTCCTGTTTTGTAATTAGCACCCAGCAATGATGACGCGG
TTTCCAGTCGTTCTCCTGGGCGGAATGGAGGGCATTACTCAGGTGGCTAATAGTTCCCAC
AACGAAATAGTTGGACACATCAGTTGACTGAATAACCTTCGTCTTGTTAACAAAATCCGC
CGACGATTCGGACACTTGGCGCATGATGTGTCGCGTGGGAACGTTCTCCAAAAGGCGAGC
GTATTTGTGGTCGATAATGACACTCTTATCATAAAGAATTCCAGCTGTCGCTAGGTCCAT
CTTAATGCTCAGTTCCCGAACGGCCTGTGTGTACGAATCGCCTGGCGGCGTCACGTGTAT
CAAGTAGTCATTCTCCTCTGGTTTTTTACCATTCCACCCACTCATCTGATATTCGTCGGT
GCTTCCGTATGAGAGATCCAGTGTTGGTATAGCGAGATTTCTCACTGTTTGCTTGATTTC
TTTTGTCAGTTTATAATAGTTGCTTCCGGGTGTACGAACCGTATTTATAACGAGACTCGG
CTGATTCTCCATAATGATGTCGCATAAATCTTTTAATTCGTCGTCTGCATCCATAACGAC
GAACTGCCAATGGATAGTTTTTCCGTCGTTTTTTGCACGCTGTAGGCCAGCTTCAACGGC
TTGTCTTGCCAGCTCGAACTTCTTCTCGACGATGACGGCAATATTAAGCGTCTGTCCAAG
ACCATTTTCGACGAAATATAGAGTCACCAGTAATTCGTATACTACTCGTAAAAGGATCTC
CATAGCGGCTTGGAATATTCACGCTTTACGCCATCTGAAGACGTGGTCAGCTTCATGACT
TCAAAGACCTTGACAAAGACCGCTCCTCGATCGTTTATTGGCCGGCCGCCCTCTCCCATC
ACGGGACCGCCCACACCAATCAGCGTGTTCACGGATACTCATGGGGGTAG

>Vd18743 len=6368 path=[9503:0-1711 2676:1712-1994 17192:1995-1995 11496:1996-6367]
CATCATCATCATCATCATCATCAAGCACACAGCAGCAACAGCACGGGGAACATCTATGAG
GACTACGAAGGATACATCATGGACCTGCTGAAGATCTTGGCCGAGCAACTCGGCTTCCAC
TACGACCTCTATTTAGTGCCTGATAACAAGTTCGGGGTCATGAACACCAGCACACAAGAG
TGGAACGGCATGGTCAGAGAGATCATAGAAAAGAATGCAGATCTTGCCGTCGCTCCGATG
ACGATCAACTTCGCTCGTGAAAGTGTGATCGACTTCACGAAGCCATTTATGAACCTTGGA
ATTCAAATCTTGTTCAAGCTGCCTAGCGGGATGCCGGTGAAGCTGTTTTCCTTTATGAGC
CCTCTAGATACGTACATTTGGATGTACGTCCTCTGCGCGTATGTCCTCGTCTCGTGCACC
ATGTTCCTCGTGGCGCGTTTCTCGCCCTACGAGTGGAAGGACCCTCACCCGTGTGTCAAA
GAATCCGGACTCATGGAGAACCAATTCAGCCTGGGCAATAGTTTTTGGTTCACGATCGTG
ACTCTGATGCATCAGGGTTGCGATTTGAACCCGAAGGCAACATCGACACGAATGGTAGGC
ACCATCTGGTGGTTCTTTACGCTCATTTTGATCTCGTCGTATACAGCCAATTTGGCGGCC
TTCCTCACCGTCGAGCGCATGATCACTCCGATCGAGTCCGTTGAAGATCTTGCCGATCAA
TCCAAAATTGCTTATGGAACACTCGACTCCGGATCAACGATGACTTTCTTTCGCGACTCG
AAACTTGAACTCTACCAGAAGATGTGGAGATATATGAAAAACCGTCCTACCGTATTTGTG
AGCACGTACGAAGAGGGCGTCGAGAAGGTTCTCGAAGGCAACTATGCATTCCTCATGGAG
TCCACGATGTTGGACTATAAGGTACAGCGTGATTGTAACCTAACGGCCGTCGGTGGCCTA
CTCGACAATAAAGGCTATGGCATTGGCACCCCTATCGGGTCGCCATGGAGAGACAAACTT
TCGTTAGCCATACTCGACCTCCAGGAGAAAGGTGTAACTCAACAGCTATATAACAAATGG
TGGAAGTCGCCGGGGCTGACCTGTAGCCGAGATGAGAAAAACAAAGAGGGCAAAGCAAAT
GCCCTTGGCATCGGCAACATTGGCGGGGTTTTTGTCGTACTACTCATCGGCCTCGCCTTC
GCCATTTTAACAGCAATTTTCGAATTCATCTGGAACGCTCGGAATAATTCTCAGGTCGAT
AGGCGATCATTGTGCACCGAAATGGCCAATGAACTTCGGTTTGCCGTCCGATGTGGCGCC
TCGAGACAAAGACCCGCCTTCGATAGTGGACATAATCATCCCGCCAAGCGGAGATGCTCA
AAGTGCCTACAGGGCAGCACATTTGTGACCTCAGCGTTGGATTTGCCATTCCCACCGCCG
CCTCCGTTTCCGGGCCACGCCGGCCACGGGATTCGTGAAAACGGGCCTATTTGTGTGGGC
ATCGGAGGCGGTTCGTGTAGTGGTCCAGGGCTGGCGCGACCTCCTTCGCCATACGATTAC
GATGACACCTAGCATCAGGCCCTGGAGGTCGACAGCAGCGTCGCTTCCACCTCCAACTAT
GAGATCAACCCACAACAACAAGCAGCCCACGCTCATCATGTGGCCTCGCAAGGAACGACG
ACTTGCAGTCAGCAAGACGGGCGCTTTGCTACGATACCTCGTCGCGGCTCGATGACCTCC
TTTGGGCCTTACTGTGTGAACACCGATGCCATCCTTTCACAGCCTAGTGTTGGCGCTGTT
ACTGCTGGAGGCGCCACGCGTTGTCTTTGTGCCCATCCTGCGCCAGCATCCCAACCGCCC
ATAACAGCCTACGCGTCACGCTACTTTCTGCAGAGCGACCGGGACCGAAGTATTTATCAC
AACCATAAGCACGGCCATCAGTGTCTCTGTTACCATTCCGTCTACAACCGGAACAGACGA
CAGGGCGCCATACACGAAAACTGCTGTGAGTCAGGGGAAGTTCGTGCGCGAGCGATTCGC
TTCAGAGGGACAACGGACACCAGTGGAGGTAGTCCTGTTCATGACCGAACCAACTCCAGA
GATCCGCGCAGCTGTCCTTTGCACCATCGGGAACGTTCCGCTTCTCGAGAGAGAGCCATG
CCCGCAGACAATGACAGTTAGCCCCACCAATCGGCCGTTCCGGGCAACTACTACCTAAAT
GATGCAGCGAACAGGAACCAACAGGACGATAACAGCATCAGTGACCACCTCTACGCTGAC
GATGACGACGATAGCAACAACAAAAACGGAAACATCAGCAGTAACAACAATGGAGAAATC
CACATCTCCCAGTTCCTGGACGGACAAAAAAATTCAAGCTTTTGCCAGCACATACAAACTG
AATGCTACTGGCCAAATCGAACTTGAATTCGGCGAAAGTACACACCTAATACTAATTCTA
CCACCGTTTTCACGAGAAACATCACACCGATTTGCCACAGCGCTCGTTATCGCCGTAGCG
TAATATCCTCAGTATCTGAAAAGGACGATCCTCAAGGACAGCACGAAAACCAGATCATGG
CCACAAATAAGTTGCTTGATAATGAAAACAATAATAATAATAATGATAATAGCAGTAATA
AATTGAAGTTGCTTGATGTGATGATGACAGTCACAATAATAAAAAGAGAATTTTGACGAA
AAAGCCGACCAAACTAAGACCGGCTTATGTGGTCGACACCATTTGCTCAAGACGATCCTG

ACCACGCGAGCGTAGATACGTTACCACGGCGACAAAAGTATTCATGTGCGCTCAGCTTAC
GAGGAAGTTGATCCTTGCCTGTAAGATAGCGTTAAGTTGACATTTACCTATTAGGTCTAC
ATGGCTGTGATCGAACGATAACGGAGAAAAGCAAGAAAATCGAGTAAAGGAAACCTAGCT
AATTTAACCGTTACAACTGTTGTTTCTGCACAGAACACAGACAGAGGCGCCATAGAGCAA
AAAGCGTTGGTGGCGCTGGCATTGATTTTTAGGGGGCGTTCATGGCCGCCATGTGTGTGA
CACGCTCCTCAGCACTATGCATTATGTAAGCTAATGGTAAAAATAATAATAGCAGGATGT
ACGTCGTCCACCCAATGATGTACAAAATGGAAAATATGACAGAATCAATATGAAATTAAG
AAAAAAAAATAGAACACTATTGATACTCACGGCAAAGCTAACAATAACTGATGATAACAG
TGATTTTAAGGTGAGACCAAAATTAATACTACAGATCTTAACGGCTGCAACCACACGAAA
ACGTGTCTGACATATTGATGATCATTGAACAGTAATAGTATGATCTATGATTTTCGACCA
AATCTATATACAAACAAATGCAGACTGTGTTGCTGAACAAAATCAACAGAATGACATCTT
GCGACAAAGGAAAAGGTGAGGAAAAGATTAAAATGCGGTGCGATCGTTTACTTTGCAAAA
GATACTACGTTTTTCATGACGAAAGAAATTTAGCTATCGTTGCCAGTTGCTGCAAATGTG
CTGGTCCGACACTATAGGTGTCCCTGCGGCTAACATTTTCATCGATCCGTCTCCCTGGCT
TCTCTTCTACGGGTAGAAGTTGGTCGCATTTCGTATGTATTAGATAAACATCTATGGCAA
AGTAAACTGCATACATGATATAACAGTGCATGCGGAAAGCTAGTGGCTTGCTGACAAGCG
GCAACAATCGAAAGGAAAATGTCCACGAGTGGAGAACGAAGGCGGGAGAAAACGTATATG
TAACGATATAGGTAATACATCCTATAGAGTGATGAAGAAAGAAAACAACTCATGTAAATG
CCTTGCCGAAAATGCGTCATAACGGTAACCTAGCGTCGTTACAAACAGAGAACGTCTGCG
CTAATACAAACATTAAAACTAACAATAAAAACAATTATTATAGAATAAGATGGGAAACTA
ATTTACTTTATGTAAACAACTAATAAATGTCACTAATGGTAATGGTAACGAAGGCATAAA
CATAGAAGGGTAAATATTACAACACGAAAGCTGGCGAAACAATACCGGACGGCAGGTAAG
CTAAGCCAAGTATGAATTGAAACAAGAAAGTACGACACCGGACAGATATCGATAACAGCG
CTGATAAAAAGGTACGGTGTTATTAAATACGAACCTCGGAGTCAGTGAAGGATAGGATTG
TAGAGATATTGGCTTAACGCTGCATTTCCCTATTACTAGACTGATTGACCGCGGAGCTTC
AAACAACTACAATAGTTTCGTATGTGTATAGGGTGTGCCTTAGTGACGCATGTGAACGCA
TACAGGCTTCGCAATCTAAACACTAACCCGTTACGTTGGCGGCGATGCTTCTTCCTCTCA
GTTGGTGGAATATCGTAACTCGCCGGAAAGACAGCCGAGGATACGGTTTTATCTTGAAGA
AAAGCGCAGACCGTCACGATCTGATTTCTTTTCCGTTTTAAGTCACTTTGCGATTAGCGG
TTGTATTAACCGATACCAATTGGCAAAAGTGGTTTTATCCCTGAACGGCAGGCAATCCAT
GCAAAAATGCAAGATGATTAGGCGCAGCCGTACGGAGAGACCTTTTAGGTATTAAGAGCT
GGTCTTCTGTTCCAAATTCATATCTTGACTCTGCCTAGAGACACGTGCACCTAATTGAAA
CGCGACAAGATAATGATCGAACGTAGCTACTAGTAAACTAGTTAATTTTAGTCCGACAAG
CCTATACAGAAATTAACTGCCACTTCTAAATATTCGTACACGTTTTTAAAGTTCTAGTAT
TTAATTAAACCAAATGTCCCTATTAATAGACGCAAAAAAAGCCTTGACCTCTGCAGAAATT
ATAAAAACATCATGATCGCTTGTAATGACGATGACTCACAGCTCTGGATAATATCGTTTC
GCACTAATGAAGAAGCTTCGACGAATGACAAATTCTTTGTTCCTTATTGCTAGTATTACC
ACTACACTCTCTAATTAGGATCAGCAGTAACATGTTACTCACTAAATTTCGATGAAGAGT
AAAAGTTTGTCGTGAAAAGATAAGCCCTTGAACGCGAAACACAAACATTTAAAGAGCCTT
TCTTAGTTCGTCCAGGCCTGGCCCGCGTCGCGCCCCTTTTCTCTCTATGCATGTATGTAC
AACACTATACTATCGACTATTAGGAGGAGCCAGAAACGAAGGCGTTTTTGTAAAAAGCTC
GAGACGAGCAAACAGTCGATCACGATAACAATGAACATACGATGTATAACGAAGCCTCAA
CTGTTTTTTTCTAAATGTGAGCAAGTCGGGGGAAAAGCGGAGCAAGCGACACGAAAACCA
AGTAGGAAATCGCTCGAAAATGACGATCCGATGACATTAGATGAATGCACAACACTCATC
CGACAATCGGCATAGCCTCTCAAATGAAGACGCTGACGATCGTAGCAAATATACTGATCA
TAGCGGAACTGTTAACAGATTGTAACTAGGCTAGTTCCAGAACTAGAACTGTACAGCGGA
AGTTGGACAAACATAGCCTGCGACAAATACGGACAAAACGGGCAGACCGAATGAATCCGA
GAGATTCCTTGATGACAGAAATCGACTAACTTAAAACGTAGATCAAACTTAAATGTAATA
TATAGCGTATAATAGTAACTATGAATGAGGCATAAAGCTAGCCAAATCACTGACAAAGTT
CCTAGTAACACTTCATCGACCTAAGTATAATCGGAAGTTGTTTTTGTGTACTCGCAATGA
TGCTGTAATTCAACAATGAATTCTAGCTGATTTATCGAGGTGCGGTAAATTCGTATCGGA
TGCATTCTTGTATGGAAAAAAATCATGGGTTTTTATTGCGGATTACCTGGTTTCGTTCAA
CTAAACTCTAAAACCTATTTAGACATTCTCTAATTTTACCATTTTATTCAGTGATAATAA
TCAATGTCCACCAGAACTAGTCCACCGCAGCTGTGAGAATCTCATTTATCGTAAATCAAA
TCAGAGACATTTCCTGAAAGTAGGTTAAATTTTGATGCCCATCGTTTTACCAGAAGGGGA
GCCTGGAACGCTACACATAAGCCTTATACCGAGTCGGAAGTGACCTGACTTTTAGAGGAG
CACCTGATACTCGAGTACTTTGAATTGAACATCGTGTACAAATTGTACAAAAACACTGCG
AGAGGAGACACAAGTAAAAAATGTTTCTTGCCTACCAATATTATATGAAAATTTTAACCA
CCCCCATTATCGTTCATTGCTCAATGGCGGGAGAGGATAGAGCAAAAATGATCTAAAAGG
TATTATGTGAAAAACAAGAGCAACGAAGGGTGTGCGAGAACGGAAGAGCGAGAGCGAGAG
AGAGAGAG

>Vd18850 len=2417 path=[4442:0-992 650:993-1153 5595:1154-1433 @866@!:1434-1808 492:1809-1937 6375:1938-2416]
CAGAAAACAAAATATTGTTAGCTACATTTATTCTTTCAGCATCAACACTGGCCTACATCT
CTTCAGGTTCTCATTGAGAATTAGTGTATAACAGGTGTTACTCTACGAGCATGTCACTCA
GCACTTCGCGAGGCCACGCATTCTTTTATTATGGGTAGGCCTAGTAGAAGAGTCGGTAAA
GACTAGAGGCACCGACTAGAGAATATGAGCTTGCTCATGGCAATTGCGTATTCGTCGACT
GCCGGAAGTTCTTCAAAAAACAGCTGTATTTAGAGGGCAAGAAAGTATCAGTGTGATACT
CATCGAATCTGCCCCTGATAGAACTGATCGAATATTTCTTCGTCAGTGCCTAGGTTTTAG
ATACGTCAGCTGTTCGAAGAAGACAATTGTTTGTTATGATCGTATATTTTGTGAATAAGA
TTTCGTGAAAAGTAATGCAACTTGGCGAAAAAATTCCTTTAGTTTAAACTCGGAAAAACC
GCGAACGCTTTTCGCGCTAACTGCACAGCTTGAATAATTAGCGTGGCTTTAGAACGTCCG
GTGTATGGAATTTTCTTTTTTGTAAAAAGTAAAACCATCTTCCCTACCTGACAAAAACTG
AGAGGTAGAGAGAATTATAAAAAATTTTCGAGTGATGAGAGATATACTATCGTATAAGCC

GAGGATTGCATGCTTGAAGGTTACTTAACTGAATGGTAAAAGCGTACAATGCAGGGGGTC
GTTTATCTCATAAGTATTTATTAGTTGTCTAGTACTTCGATTGCAGGTGATAATAACACA
CTGGACAAATTGCACGGGAACCATACGCCGTACGCCAGCCAGACTGGGCAGTTTCAGATC
GAATATTTTTCTGTGATTAGAAAGAGCGTCTGTATTGGCTATAAGTCTCATTTATCGTTT
TAGTTCGATGTTAGATTTCTAATGGGTAAGTACCTTAGTAAGTGATTGTTATTAATAGCT
ATAACTGTTAATGATCAAAGCCCTTCTGCGTACTTAAGCAATCAGTTTTCCGTTCTGCCA
AATAAAGCAGAAGCACTTGCTTTCCCAGGCGAGCTCCGCACACAGGGTAATTAACGTTGC
TACGGCCCCCATTCCATAAATAATAAGGGGGCCCTTTAGCTTCGATAAACCCAACGTATA
GACCGTGGGCTCCTCCTGTGTAGGTGCTCCGTTTACGATTTCACAAGTGAAAAAGTCGGC
CATAGCAATATTAAGCCATTTACGAATATTTCCGGTTTCTTGCAACTCTCGAATTGTTTT
GTTAATGACGCGTTTGAGCATTGGATGCAGCGTTTTCCGGAGGGACATGCTAAGTCCATG
CGTATAAACGGGCTCCTTGGCGAAATAGAACTCTCCAGGGTGACCTCGAAGGGCATCGCA
GCTTCGACCGAGGTTGTACATAATCGACGACTGTTCGTTGATTATAACTGCTCGACCTTG
GAGTACATTCATGAAGTTTTCATTAGTATACAGTTGATTTCCAGGTAAAATACCGTTCTT
CTCCATTGCGCGGCTGTACACTGTCTGGTAAATATCCATGTCGGCCCTCTGGAAAAGCTT
ATGGTACGCGGTCCCTCTCCAAATGATTGGTACTACTTGTTTTCGTACCACTAAATCCCT
CATAGAGTCAATCCTACTTGATCCTGAGCTGATGAGTAAACTAGCCTTCATCGTTCCTTG
GAATATGCTCATAGTAAGATAGATGGAAGTCAGCCAGAGTCCAATTAAAATGCGGCCAGA
GTTGCCCTTGGGAAGATCTCGGTCCTCAATGCTTTCGAACAATATTGTTCTCACAACGCT
CATTAGATTCCTGCCATAGAGGACAAGCATTCGCGAAAATAGCGTACGGTTTACATTTCC
TTCGTTGCGTTCCAGAAGTCGACTAAGCACCAAAATAACAGGCATCACTAATGTCGCCAT
TAGCAGGCACGTCCAGACGTTTGATTCGAATGCCCCAAATAATCCGAAGTAATTATCTTG
GAATCCGAATGTGCGGCCAGCGAGTATACTTAAGTCCTGTACCATGAACTCGGAAGACAG
AGTCACTACCCGATGTCGATCCAATGTTTGTCCGAATGCGTTCGCGGCCCAGTCGGATTC
GTTTCGGGTGAGCATTCCAATCATGCCCGTGGCACTGCCGTTCGGAAATATCACGCCCCA
CTGTTGATCTGCTGGAATGTTCGCTTCGTACCTATGGTGCACAGTCGTCACTCGGTTAAT
CTGGTACTCCATGGACTTCACTGCCATTACTGGCGTGAGGCTTAGAAATCGTCCAGCAGT
ACAGTTCTAATGCGCGCGTTCAACGACTTGTTAGACGTGTCCCCTGTATATGCTGCTAGC
TCAATGGCCCATTCCTTTGACCAGAAACCCTCAACGGTCTCTTTCAGGCACACGCATACT
ACAACTGAAAGTGTCCA

>Vd19098 len=4303 path=[4685:0-822 312:823-972 @5657@!:973-1577 6262:1578-2128 6813:2129-2855 9711:2856-2857 7541:2858-4302]
GTTTACGTCTTGATGGCCGAATTTCAGTGTTATTCAGGCGCCAACAACAACTAGCAGCAG
ACTCTCCGCCGAACGAAACGGCACGACGCACCATCCTGATCGTCAGTTTCATTCACTCGT
CGATAAATCAAATGTAACTAATTTGCAAGTGGCCTCAGAAGAGCCAGTGGGGATACCATG
TGTCCGTGAACTGTTCGGGGAAGATAGTGATTTAGACAGCGTTTCCATTAATCGGAAAAG
AGCAAAGTCTTGGAAAGCTGCGTCACTCACCGGCGTGTGAAGATGTGGCTAGGAGTCTTC
GTATTCGCCATACTCGTAGGTGGATGGAGATCATATGGCGAAGAAATCAAAGCTGTGGTC
CTACCCGACAGTCAAATCGAGGTGGATGGGCAGGATATTCGCGCTTTCATATTTAACAAC
ACTAACAAATACAACATCACTTTCGGCGAGGTGGGCCATTTCCTCTCCTTCATAGCGACG
AATATCATGGACGTTAGTGAGCTAAAGGAAAGGGTAAATCCGGAGTCGGAGAATAGCATC
AACCTAAACCTGCTTGTGACCGCTCTGGATTGCGAAGACACATACAGTGCTATCGACGTG
CAGATGAAGAAATTTCCCACTGCAATGCTTCTTGCTTTCAATAGACCCTTTTGTCCCAGA
TTCGATGCAGATGACCAGGAAATAGCAACCAAAAAACCTTTTGCTATCAGCATTCCCGTA
ACTGAGGGGCCGACTGATGTCTTACCAATTCTTGCGGACTTCCGAGCGATGCATCCACTC
AGCCGGTGGAGTGAAATCACCATTATAGTTCCGTACTCATACACTGGAGAATATCTGGAG
CAGATCATGCGCGCCGTGGAAGGTGTCCGCAGCAATGATCAGGTCAATTCGGCGGCATCA
ATCGCCCTTTGGAAATATTGCCCTCCATCGGCACGCTCTGTCGGAAAGTGTGGATTAAAC
TCTGCGTCCATGAGGGGAATTCTGGATGCGTATCGTGCAAACCCGAAAAAGAAGGATAAT
AGGGAATTTATCGTAATTGGCGATGAAGAATTAGCGTTGGATTTCTTTGAACAGAGCGTC
GACTACGGGATGTTTAGTGTTTTTCGTGAATTCTTCATTATTCTCACCACGCCTTACTCC
AATGTCGTCAAAGATTTCTTGTTTGACAAAGTTACGAGCGACGCCAATGTCGCGATCGCA
TCGACCGATGTTAAGGATGAGGATTGTCCTGTAACGGACAATTGCCACATTGAGCTGCCT
ATGAACGCTTACATTAATGCTGTGAAGAATAATCCTGATCTTTTTGGTCGGAACGTTGAT
AAATTTAATCATAAATATACCGTGAAAAAAGCTATTCTGAAAGAATTGCGTGGATCTGGT
CAATGTGGCTTCTGTGCGAAATATACTATACGAACCCTGACTGTGAAGGCTGGTCGAAAA
ACGTTTGACCCAGTGGCCGTTTGGGACTATTTCAATGGACTTCAAATGATTGACGGAGCG
ACCCGGGTCAACTATCAGCTATTTCCACGATTTCTTGGAGATATGGGTGGAGTGAACCTT
CGAATCGGCGTTATTAACGTGCGTTCTCCTGTCCACAATCATTTTTGCTAGCTAGGCTCT
CAGTCGGCTTTCCATTAAATAAAGAAAATGTTTCTTTGAAGTGCATAATAGCTCTAAAGA
TATAGACACATCTTCATGATTTATAGGTACGTATACAGTTTTATATCTAAAAAGTCATCA
GGCCATGAGGATACATAGCACATTGCTTTAAATATTATGTTGTTGTATTCAGATGGGTCG
AAGTATGCGTCACATACTGAAATCTTTATTATTTACAACCAATACGCACCGTTAGAAGAA
CGTTAAACGGTAACGACAATGAATGAATTCGCGTTTTCGTTTGTAGTTTGTTTTTGACGA
TGTTCCAAACTATACTAATTGCTCCTTCAACACGTTGTGTCTTGTAACTTCTTTTTAAGC
ATAACTTCAATTCGATCAGCGCGGTAATTAATCAACTGCTGCTGTACCGTGAGATTACAG
GATTCAATTAGCGTTTATTTCATTCTACTGGATGATTACAGTCCATTTTAGATTAAACAA
AAACCGCTAAATTTACGCGACCTTCCTAGGAGCCTCCTATGACAGAAGTAACTTCTCGGG
AAGGACGGGTGGTCAACGTTAACGGAACCCTCGTCGAGCTCTTGAAAGAGCTTGCGAGAA
AGCTCAACTTCACATACACGTTCATCATGCCTCCGGAGCCTGTACCAGGCTTGAAACAGA
GCGATGGCCGATGGTCGGGTTTGATTGGTCTGCTGGTTCGTCAAGAGATCGATTTAGCCC
TTTATGATTTCACGCCAACGCCGGATCGTCAACAGGCTGTTAATTTTACCGTCGCTTTCG
ACGAGTCGCCGTACAAGTTTCTTGTTCCCAAACCGCAGCCCAACTACAAATATCTATTCC
TCGACCCATTCACTTGGGATACCTGGTTGGCCGTGCTGGGAACTGTGTTAATCATCGGTC

CTATCCTATGGTGTGTTCATATTAACTCCAAGTTCTATGACTACTACGACATGCGCGACG
GCAAAGGTCTCTTCAAGTTAGCCAACTGCGAATGGTACTGCTTTGGAGCCATTATACAAC
AAGGCGGCATTCATCTTCCTGACGCTATCTCTGGCCGTATACTCGTTGGGTTCTGGTGGC
TGTTTGTAATTGTTACCCTAACTACTTACTCCGGCAATCTCGTCGCTGATCTAACTTTCC
CCAAGATTCGAAATCCGTACGATACCGTTAGCTCGCTACTGAATTCGGACATCACGTGGG
GCGCTTTCAAGGGTCACGCAGTTATCGAGATCCTCAAGCTCCAGCAACAAGGGCTATTAA
CCCAACTGTCTGCTAAATTAGCCCATATTGAGACGGCGCATGAAAGTTGGGCACTGAAAG
AAGTTGCTGATGGTAAGATGGCTTTAATAGGATCCGAAGTGACTCTGTTTCATTACATTG
GCAAGCAGTTTATAGCTACAAACTTATGCCAGTACGCAGTAGCCAAAAAAGAGATTATCC
GACAAGTTAAAGTTCTCGCGGTCCGTCCGGGATTTCCATTCCTCGCTCGTTTCAATACCT
TATTAACTCGGATAGTTGAGACAGGCCTAATAATCCGTTGGAAGAAGAAATATTGGCCCA
AAGAGAACGAGTGCACGGTCGATTCGAAACCTCAAGCCGGGGATATTCGACGAATTACAA
TTGCCCATATGGAGGGTTCGTTCTGGATATTGGGTGTCGGTTTTCTCGTTTCATTTACAC
TACTAGCCCTCGAGATATTTCGAAAGAGACGCGAACTTCGAGACCCCAACGGAGGGAGAC
CCCAGCGTATCGGCCCCAGAGATTTCAAGGACAATTTGTTCAGAAAGACGAATTACTCAG
ACAAAGATCATTACAATACCGATTATGGCGCACAAGTTGGAGGCGCTGGTCCTGGCGCAG
GAAAAGGTGGTCAATCGGGTTACGGTTTCGAACCCTCAAACACGCCCTTCCGAGGTTATT
CTGGATTTCCGAACAGAACCGATCTCATTCCCTACAATTATCCAGCCAGAAGATACTAAT
TCCCCATAGTCAAAACTACAATCAACAATGTCCTAAAAAAGGATCAGTTCGACAGACGTC
GTCCGTGCAAAAGATAGTGTCGAATGAAGTTTCCTTTGTTCTTCTCCACTTTGACAGTAT
TTACTTGTTCTCTGGCTTTGGAATGACTCAGGTTGTAGACGGACCAAGACGAATTGTTGG
TTCTAATAATGTGTCCCTGAATCACTAAAAGAAAGTACAAAAAAAAAAACTTTGCTAGCAT
ACGTTTCGAAATGCTGCACCTTATAAAAGTTGTTATTTTGTTTGTAATTACAACGCAATT
GTTTTTAGTATCGTGTAAATTAAAGTTGTAGCATTGTTGGAAATTTGTAAACGTTGACAA
TTTCATTTAACTTTATACTGTAATGCCATGCGTAGCTGTGTACATCTATAAGTATAGACA
TATTGACCTATGGCTCATTCAAAAAAAAAAAAGTAAACATTGGCTAGCTCTATTTTATTTT
GCTGTGGTGTTTTAAGTTTGGTTGGACAAAAATGATCCCTTGCCCAGTGGCCTTTTATAC
GAAGTTGTCGATAATTTATAAAATACAAATGAAACTGTGACATGAAGAACAACGGCTGAC
GTAACTTGATGTAGCGGAATCGAAGAAGTTGATAGGGAAACTAGGATGGGAGAGCTGCGA
TTAAACTAGAATTAGTTTTGGTGTGAATAAAAATTCTATAAAA

>Vd22240 len=1315 path=[54524:0-59 @54584@!:60-168 @54693@!:169-536 @55061@!:537-811 55335:812-1314]
CTTAAGAACTTTACCAGGAACAACGTCTCTGACGTAGCCGATGTCAACAGTACCGCTTGC
CGCATCCTGGACAAATTTCCTCATAGTGAACACATCGGGCCCATAGCCGAAGACAACAGT
CTGCACGATCGCCACAAATGCGGATACATAAAGAACGGTAACCATTGCCAAAAAAAACCA
CCAGAATAAAAGTAGTAAGCGTGATGCGCGGCACCTGCCCTCGGGGCCGGGAGAGCGGCA
CAGGAATGAGCCAGCCGAAAGCCATAACGTACGACCAAAGCTGTCAGCTTCTTCCTGGGG
TGACACGAGTCGATTGAGGAGATAGGCAAGCAATACAAATGTGAAGAACACACCTAGTAT
GCATAGCCACAATTGCCAAGAAAAGATAGTTATGAAGCATATCGAAAGCGATGGTCGACC
CGCTTGTGATTTTCGTGAGACGACAACGGACAAATCATCCATAAGAAAAGGCTGGGTATA
TTCGACATGCTGTTGCCGATCGGCAGTCAACGGAATGTCCGCAATAGCAATCTCCACTTT
TTTGTTGATTACTTCGGATATAAGTCCAGTCCACGAAGCGTTATTGTCAGTGCGTGCTCC
GGCTTGCCCACGTGGCACTGCATGTATTTTATATGAGAACGGCATTACGTTGGCGAGGGC
TTCTAGCAGCTCAGGGATCAAGCCGGTGAAGTCGGCGTCACTTCCACGCTTCAACATCAG
ATATGGTGGACTGAGTATTCCACCGACAGATATTTCTTTGTTGGGTGTGATGGCACCAGG
AGCTTTGATTTGAGGTAATGCTTCAGGTTTGTCGGTCAGCATAAGGCCTTTTTTAGGTGA
CCAGCTTCCTAACTTAACGTGTCCAACATGTGAGCTGTGGATCACAGCGATAGTAGGCTG
TAGTCGACTGCCATCGGCGAGAAATTCAACTGTGCCCGTTTGTCCTTCTGGAAGTTTTAT
CTGTTTCAGCAGGGAGGCGAGGTCCTTGCCGTGCTCGAAAAAGCGTGACTGATCCATACA
ATTGAAACCAACAGTTCCCTTGGAACCATCTTCTTGCGGTAGATAGGCTTCGAGTAGCGT
CGGTTTATCTTGTAGGGCACGATCGTAATTAGTAAGAAGTGCCGTAACAGCGTCGAGGAA
TAGATAACTCTCGGTACCTTGATTCTTAAAGGAGTCCCTGTAGTTGGGATTCTGCATCAT
TTTTTTATGGAAACGCATGACATGTGGCAATTCAGAGTCGACGAGCCGGAAAGCGGTCAC
GTTCAAACTGCTACCGGCATCACGAAACCGGATGCTCTGCCAGAAGTCGTCCGTC

>Vd18951 len=6474 path=[7111:0-2634 9746:2635-2669 9781:2670-2992 @10104@!:2993-3330 10441:3331-6473]
GATCGTAGTGAGCCAGCCGCAGAAGAAGTGCAGGAAAAGGAAGACAACGGTGTGTGTAGC
ATTCGCTCCGTCTGTGTGTGATTGTGTGCGCGCGTGAGTGTTTTCATGTAGATGTCCGGT
GACAATAGCTGTTGCTAATTTGATAACTTCTACTACGACACTTTACTATACGTCAACCTG
ATTTGGAACTCACCAAGTAAACGACCCTCACAAAGAAGCGCCAGCGAAACCAACCACAGC
GGCCGCAGCAACCAGAGATAACGACTGCCCTGGCGGGGACGTCCTGGCTGCGCTCTGTCG
ACGACAACGACAGTCAGAAGATAAAAAATCAATAAGGACGGCTATTCGGCAACGCTAAAT
GGTATAAAAAAGGATCCCGATGCCTGACAAACGCTCAAACTTGGGCTTCGCGATGTCACT
GACACGAAGACACGAGTACGTCTGGCGATTATCTACGTGAATAGTTTGATTACGTTAGCT
CAAACGGCGTGCCCAATCGCGGACCGGAGAGTCGTGCAGATTCAGCTTAGCCATCCATAA
CAGAAGCTAAGAACAATACGCAAAACAAGTAATATGGCTTAATCGAGTTACCAATCCATC
AACGAATCGATTTGGCTGGAACAGCTATACATCGATTGATTTTGAGTCCATAGTGAGCCG
ACGATACAAATAATCTTTCTGATGATTCTTGAATTGGAAAACGAGCCGCCGACAAGACTT
TTGGGATCCTGATGCAGCCAGGAATTGAGGCCGTGAGTCGAGTCCGCTAGCAGTTGCAGG
AAGAGCAAACAAAGACAAGGACGCGTCACACACCGGCGCACGGCATGAACACGTCCTCTG
TCGCTGAATTTCAAACTGCCAGTCGCTGAGTTACAACGTCAGCGACCTCACTAGGCTCAC
GGACCATAAGCTTTGAAAACTTCTCCGCCGTGTGGTAGCCGTGGTGGAACGGCGAACGCT
AAACGCCGCTAGAATTACCATCGTCATCGAGGACGACGAAGTTTATTCGGTTGTCGCCTG
CAAGCCTAGCGGCTGCCGCCATCGCTATTTTTGGTGCTGCGCAAATCCCTGTTGAACCCC

TTGTTCTTCCGGGGATCATGTTATTCCTGATAGGCACTCAGCTGAGCAGGAGTCGGCCAC
CGTCTACCAGGCCAAGCCCGCAAAGTCGAACTGGCAACAGCAGCGCTGACTGGGCCGATT
CGCGGCGTCTCTTCGTCTATTCCAGCCTTCTCCTTCTCTTCGTCACGAGATGCGCGGCTC
TCCCGCCCGTCATCAATGTCGGAGGGCTGTTCGACAACGAGGACGACGAGCAGGACTTCG
CGTTCCGCGTGGCCATCGAGCGCGTCAATAACGACCAAAGTATTTTGCTCAAATCGCGGC
TAGTGCCGCGAGTGGAGCGCGTCGAGAAAGACGACTGCTTCCGGGCGACGAAGAAGGTAT
GCACGCTGTTACGGGAGGGCATCGCGGGCATCTTTGGACCAACATCCGACGTCACCTCGA
TGCATGTCCAGTCGATCTGCGACGCCCTCGACGTGCCCCACGTTGAGATGCGCTGGGACT
TCCAGCTGCAACGCGATGATCTTTCCATAAACCTCTTTCCGAAGCCCTCAATTTTGGCCC
AGGCTTACGTTGACCTGATCAAGACGTGGGACTGGAAATCATTTGCTCTCGTCTATGAAG
ACCACGAGGGTATTATTCGACTTAAGGATTTTATCAAGGGTGCTCGTCGCGAGGGCTGGA
AGATTCAGATGTACCAGTTTGTGCCCAACGAGCCCTACAGAGATCTCTTTTGGAAGATCA
AGAAGTCCGAACAGAATAGGGTCGTTCTAGATGTTCATAGAAAAAATCTATACGAATGCC
TTAAACATGCTCAGCAAGTCGGAATGCTTACGGAATCTCATAGCTATTTAATATCATCCT
TGGATCTGCATACGGTGGATCTGGAGGAGTTTAAGTACGGACAAACAAAGATCACCGGCC
TGCGGCTAGTGGACGTCGAATCGCCGGAACTGCAAAACTTCCTCGATGATTGGAAGCGGC
TTGCACAGCAGCCGAAACATGTTCGGGCACCGCCTGCTCCCGGTCCGCACCTCATCAAAA
CAGAAACGGCACTCATGTACGACGCCGTTAAATTGTTTGCGATGGGCCTACAACAGCTCG
ACCTCACCAAGGCGGTCGACTTCCCTGTGATATCGTGCGATGCGGCTGAGTCCTCTTCAG
ACGGCAGCAGCCTTATCAATCTCATGCGACCGATAACTTTGCAGGGTCTAACCGGTGATA
TTAGTTTCGACTCGCAAGGCTTCCGTTCAACGTTCCAGCTCGACGTCATGTCTCTCAAGA
CAGACGGCCTACAAAAGGTGGGCTACTGGAATCCGAAGCAGCGGGTCGTTGTTGAGGACA
ATTCAACATCCGACTATGACTCCCTTCGTCTTAGGAACAAAACGCTCATCGTATCTACTG
TCCTCACGGACCCGTACATGATGCTGAAGGAGTCTGCCAAGTTCCTAACTGGCAACGAGC
GCTTCGAAGGTTATTGCGTCGATCTACTGCAGGAACTAAGCAAGGACCTTGGATTCTCAT
ACGAGATCCGGCTGGCAGCTGATGGTGCATACGGCATAAAAAGCGACACCGGTGTCTGGA
ACGGAATGATAGGAGAGGTCGTCTACGGAAAGGCGGATCTTGCAATAGCCGACTTGACGA
TTACGTCAGCTCGAGAGGCAGCAGTGGACTTCACGATGCCGTTCATGAACACCGGCATCT
CGATTCTATTTAAAAAACCTACTCAAAAGGCCACCTCATTGTTCGGTTTTCTGTCGCCGT
TTTCTACAGAGGTCTGGACATACGTGGTGGGCGCATACCTGGGGGTATCATGCGTGCTTT
TCCTGGTCGGTCGCATGTCGCCCTACGAGTGGGACAATCCTCATCCCTGCCGCCAAAATG
ACCAGGTTCTCGAAAACAGCTTTTCACTACTCAACTCCATGTGGTTCACTATCGGGTCTC
TCATGCAACAGGGATCTGACCTGGCTCCTAAGGCCATGTCGACCCGTACGGTAGCGGGTA
TCTGGTACTTTTTTACACTAATCATGATTTCATCGTACACCGCCAATTTGGCCGCCTTTT
TAACCGTCGAGAAAACGGTCTATCCAGTCGAGAGTGCCGAGGACCTCGCCAAACAAACAA
AGATCCAGTATGGCTGTGTCAAATCCGGCAGCACCCGAGCCTTCTTCAAGGAGTCCAAGA
TCCCCACATTTATGAAGATGCACAAGTTTATGGAGGAACGCAACACCTATGTCGCAACCT
CCGCCGAAGGAAAGCAAAGAGTTAGCAATGGCGACTATGCGTTCCTCATGGAATCTGCCT
CGATCGAGTTCCTCGTCGAGAGAAACTGTAACCTAACCCAGATTGGAAGTTTGCTCGACA
ATAAAGGATACGGCATTGTCACCGTAAAGAACTCGCCATTCAGACAAGTGCTATCGTCGG
GCATCCTGCAACTGCAGGAGGCAGGAAAATTGCACAGCTTCAAGGAGAAATGGTGGAAGG
AGCGCAAAGGAGGAGGCAAATGCACCGACGATACCAAGAAATCATCGGCAGTCACCGAAC
TTAGCTTAGCCAACGTAGGCGGCGTCTTCGTTGTCCTTCTACTCGGTTTGTTACTGGCAG
CGTTGGTAGGCATTGCCGAGTTCTTGTGGAAGGCGAGGTCCCTCTCAAAGGAAGACAAGG
TCTCGATTTGCTCAGAAATGCTGAAGCAATTGAAGTTCGCGGTCTCGTGTAGAAGCTCAA
CGATGCCCGTGCGTAGCTCGAGGCAAACGCCCGAGCTTGACACCGTCGACGCTAACAATC
TCTCCAACCGATACGGCATCTCGACATTGCCCTCGTTTACGTCGGACTTCTGAGTGTACG
CGATACAGCCGGACGGCGGAGCACCTCTGCCATTAAGCGAGTATAACGAAAAGCTCGCAT
TGGAGCTATTGGCACGTGCGTCCGGTCTGAACAGTAACAATAATTCAAGCATCTGCAAGC
AGAATCCCGGCGTCGGGGCTATCAGACTTCCGGCAACAACAGGATTTGAAAACAATCACT
AACCTCTTGGATGACCATTACGTTCAAACGTTATTGAAATTGCGGAAGACTTATACGATC
TGAATGGACCGCAGTATAGGGCAGTTGGCACTGCCTCAGCGACCGTTCAGGCCAAGGCAA
AAGAGGCAGCGCGCACGAGCGCCTCGTTGTGCTGCCCAGAGTTCCGGGGGGCCGACTTTT
CGGATATGATCGTCCAGTTAAAACCAGTAGCACACACAAGCGACAGCAGAGTTGGGGTAA
AATGAAAACGGAGACGCTGAAGAAATCAAATCAGGAAACGCAGCAACAGTATCAGACAGC
TGTACTGGTCTGATCAACAGAAGAGATGTGCATGCGAGTAACGCTATTGCCATATGGAAT
GATCACTGTATTGATGTGGATGAGGCGGGTTGCTTTTAGACTAGTCTCGAGTGCTCCGGA
TAATTAGCTCGATGACTACGGAGAACGGATACGATAAAACAAAAAAAAAGCTAGCCGGGA
ACCCTCTCTGCCACACCACCTGAGTTAAAGAATGTTGATGTTCCGTTAACTCTTGCCAGA
GCACTAAACGTAGCATGGCCGCCAATTTTAGCCCATTTATGTGCAAGGAAGCATGTTCAC
AGTCGTACAGAATAAGAGCATACCAATCGCGCACGTATTACAGACATTTCTTCCTCGTAG
GGACCGAGCGTCGTATACCATGCAGACCTTACTTTTGCAAAAAAGTATTTCAGGACAAAC
ATACCTACAAAGGTTCCGAATCAATTATTACTATAATGATTGAACGAACAGATAAGCTTC
TACTCTGCTGCACTCTATCATGAAGATGAGTTCACGCTGAATGCTGGATTACGGAACTGA
CAGAGAAAGAAAGAGTTCAGAATGAAGTAACCTATATGTATGTAACCTGAACACAAGAAA
GTTAGCGAGCATTAAGGAATTTGGTAAGCGACATCTTCTCGATATCGAAGAATGCATTTA
GAAAAGGCGATAGCAGTAGTCAACAGCTGGACCGTCCGACGTACCTGCTTGACGCCCTTG
GACTCCCGTCATCCGGCACGCGACACCTCAGACGCAAACATAGCGGACTCATTCTCGGCT
TCCAACAACAGCAAAAAAAGAAAAACAAAGTAAAGCAAAGCAAAAATAACATAGCAACAA
ATAACAGTCACTGGAGTATAGCGATATGAATCAGCAGCCTCAACGGACTGTCATCATAGA
CACTTGTATGTTCATCACGTAGTGACAAACAAAGAAAACCAATGAAACTAACTACGTACA
GAGGATGACTCCACATGCCACACAAACAGTAGCACCAGTAGATAGAATAAAGCACTTGAG
GAAGCAAACAAACAATTAGAAGGTGAAAAATGATAGCAGAAAAGGATCAAAGACAAGAAA
GACCAAACAGAGACCAGAACGGCGACTATGGCATCGCCGGCAGAGACGCTTTTACAATCG

CGAACCAAGAACGCTGACGCATTCGGGGCTCAACGAAATCGCGCAGCAACAGCTGCAGCC
ACGCCCAGCCTCTGTACAGCAATGTGGCCCTATCACTTACAACTATGCGGACTCACGATC
GCCGCTGGTTCGAGATAACAGCTCTGTCTCCTATAGATATGAAAGAATACTTCAATAAGG
GTGGCCGAATGTCACAAAACAGAGATAAAGTCAACATACTTCGTCGAAGTTGAACGAATT
GTACATAGACACAGACCAATAACAATTCATTAAGGATGAGAACGATACGAAAAGTATAAA
CTAAAACGACCCATTTTCGAAGATTGCTCAACGCCTATATATCGACGACAACAATATGCC
ATCAGTTATGTAGTAAAATACCGGAACAGATAAGCAAAACGTGCCGATCCCGCCAACAAA
CTCACTACGTCGACTGAGCGATAGCTTGTCGCGCGACAACGAAGATCGAACTAATACCAT
AGAACTACATGGCCTGTCAAAACAACTACGATTACCATCCATTATTATAATCTAGCGATA
ATAATAACAGAAGTAACGCCAATTAAAGAAAAAATCGATACAAATACGCGCAGCGGAGCT
AAGGTCGTTAAGAGCAAACTATACTATTTTAGTGTGCGTTCACACTGGAGGCGACGGTCG
ACGGCGACCTACGAACGACCATTGTGTCACACTGGCACTTTAGTAGCAGTTAACATTGCC
CTATTAGAATATGCCCAGCATCACTAGAACGACAAAACAGCTTCGAGCTGACCGCAGCGC
GTCGACAACAATGTCCAGAACCCGCAAATTTACCGCACCTCATATGACCACATGGAACAG
GGCTCGATGCCAAACACCTATCAGTCCGCCGACCCGCAGTAGGTGGCAGAGGCTGAACCT
GATCAGCCCCCGTTTAATGACACCGAGCAGACGGGAGTGGACACGGGGGGGGGG

>Vd20015 len=7051 path=[18935:0-930 19865:931-952 13631:953-1094 13558:1095-2036 @13532@!:2037-2396 4159:2397-3804 5567:3805-5139 6902:5140-7050]
ATGTGTGTGTGTGTGCGTGTGTGTTCGTGCGTGCGTGCGTGTGTGTGTGCGTGTATTCTA
TGCGGAAAACAAGTAGCGATATCGATTGCTAGAATACGGTTAGATGTAGTGGTAGTCGTA
GAAAGAAGAGGTGTAAAAGAGGCAAAGGCCATCCTCCGGAGAGGAGCGACTCGCCTAAAG
GCGGGCATCGACGGAGTTTAGTTGTGGTTTGGCATCATTTTCACAAAATGTCCAGATTTC
GTTCGTTGATTCATATACCGGCTCACATATGGCAATAAGTGTTCTCGCATGCGGGCACGG
TACAGTAAAAGATGGACAATATAGAGTTGTTTTGACTTATCTTGCATTACTTGAAATAGT
CGAGTGTTAGTAAAATAGAACAGGCGCTATACGGCGAGTGCCGCTAGAATAATTGTCGTC
GAAAAATTGCTTGTCACATTGTAAAGATATACGGCAGAGTAAACTGTCGATTCTCAACTA
GTTGAGCATCATTGGCATTAGTGATTGACTGCAGAGTTACGTTGATGCTGGTGGAGAGCT
TCCACCGCGGTCCAGATCTGCTCGAAATTTTGGTCACCTCTCACTTGAACCTTGTCGTAT
ATTGTTCCCTACGTAAGCTTTGCGTGTGAATGTACATACGTATCTTTATCTATGTTCATA
ATCGATGTCTTTCTTCCCATACACGCTCGACGTCCCTGTTATGATGGAGCAAGAAAACAG
CCTGAACACAAGGTTTCACTTGTAGTTACCCTTCAATTTTCATAAACTCCCCAATGATTC
GAATGTACAGAAGGCATTGAATGAACTGAGTCTGTCGTATGACTCGACCTTACCGAGTGG
TTCGAGTGTCTGGACTGCAGAGTGCCGGTCCTACTGAGGTGGCTGGCGGAGGTCTGTCGA
GAGAAAGAGCCCGACCCAATGTGCGCCTTGCTTCCGCTCTGTGGATCGCCGCTGCCGTGC
GAGTTACCATTATATTGCTGGTGCTGTTGCTTCTGTCGAAATTGCTGATGATACTGTAGG
TCATGCAAGCGAAGCTCGCGTATATCCTGTAGGGTAGCTGGACGGGCTATCTTGGTCTTA
GCGACCACGCCGCTTTCACCGCTGCTCATGCTGTTGAGATTGGCTATTTGCCGTTGCAGC
TGATGAAGATGGAGCGAATCTGCCAATGCTACGCCAGGAGCATCAGCCTCGTCGTGTAAA
GCACCAGCTGCGCCTGTCCCTACGGCACCGTCCATATCCTGTGTCTGATCGTCCATGTCC
TCTCTTGAATCAAAGTTTGGTCTCGAGCCCTGACATGCGAGGACGTGCCGCATCTCTTTG
AAGAATTCGCACAAAATATGTCCTCGTTGGCCGTAGGGCAGCTTGGCGCGCTTCCAGCAG
AACTCGCAGACAACGATGATTACGCCGACGGTGCAACCCACGATGAGGGTAAGGAAGACG
CCTCCCACCGAGCCCATGTCCATCTCGCTCGTCGACGACGCGCCCTTCTCGTCGTCACAC
TTCACCAGTCCGCCTTGTTGCTTCCACCATTTCATTTTTAAAGAATGCAGAACTCCCTTT
TCCTGAAGGATTAAAATTGCCGATGTGAGAGCACTGCGTAGAGGGGACTTCAAAGGTGTA
GCGATACCGTAGCCTTTGTTATCAAGCAGACCTCCGATCTGTGTGAGGTTGCAGTTTCTT
TCAACGAGGTACTCGATTGACAGAGCCTCCATCAAGTAAGCGTAGTCCCCACGCAGCACC
CTCTCTATACCCTCCTTGTTCGACGACGTGAACACTGCCGGTCGTGCCGTCTCCATGTAC
GTCCACATCCGCTTCATCACCGGGTCGTTATGTTTCTTGAAGAAACCATATGTAGATCCT
CCTCCAAGGCATCCGTAGGCGATCTTAGTCTGCTTGGCCAGATCGTTGGCATTTTCAATG
GGGGCTCCCATCCGGGCGGCTGTTAGGAAAGCCGCGAGATTCGCTGTATACGATGAAATA
AGGATCAACGTGAAGAAGTACCAGATCGAGGCCAGGACACGAGTTGAGATGGCCCTAGGT
ACCAGATCGGAGCCTTGCTGCATAATGGAACCCATAGTGAACCAGAACGCGTTTTGCAGA
GAGCTGAACTGGTTTTCCGGCTCCGACTGGGGATCGCAAGGGTGTGAGGGCACCCATTCA
TACGGCGTGAATCGGCCCAGCACGTAGACGAGGATCGATACGAACGTGTACACGGTGAGC
GTGTAAAACCATACGACGATCGAAAACGGATAGAGAAATGAGAATATGGCCGGTTCCGAT
TTTTCAGGCTTTTTAAACAGGATTGATATTCCCGTGTTCATGAACGGAATAGTGAAGTCT
ACCACCTGCTCACGTTCATATGTGATCGTTAGGTCAACAACTGCAATGTCCGCTTTCCTT
TGAATGAGTTCACCGACCATTCCGTTCCATTTTCCGTCTGGTTGACGACTTCCGTACTTT
CGATCTGGCGATATGTAAAACGTGTAGTTAAATTTGTACAGGCTTGCCAGTGCGGCGATA
AGATCAATTGCATAGCCGTAGAACCGATCATTGCCAGTACGATTTTCCGAAGGCGGATGT
AACATCGTGTATGGTTCATTAAGCACGGTTACAACTCGCAAGGTCATTCCTTCGAGCGTT
CGGCTGAGGTCCGCTTCCTGTTCAGTGCGATTGGCAGCATTAAGCTTGAGACCCTCTCGC
GGGCTCCATGAAGCAATCTTTTTTAGCCCCGTGTATTTGAGCTCCACGACGTCCAGGCCG
AACTCGTCTCGTGTCCCAGTGCGACGGTTCAAACGTAAGTCCCCACTCAGCCCCCGGATT
CGCGCCCCTTTGATGGCCTCAAGCAGGGCTTTGCCGTGCTCCCAGTGCGCGCTCCGGTTG
CAGTCAAGCTGCGAAAGGACCAACGGGCCCGCCCTTAAGTCCTTGTCCTTGCTGTTGTCC
TTGCCTTTAGCGGCTTTGTTATCCTTCGCGGCCTCTTTATCCTTCTCCAGGAGAGCCTCT
ACGCCCGCTGCAACCAAGCTGACTGCGTCATACAGCAGAGCCTCCGCTGTCGACATCTGA
TCAATAGAAGCTGGTAGTCCGCTGCTAAGGTCACGGGGTTTTCGCCAAAAAAGCGCTGTT
CCAGTTAAACCCCTTTTTCCTGGCGATCCTAAACCCTTTGAATGGCTGGCACTGACACCA
GAATCCGGTTGGAGACCCTGAGTAAGCGCCCAGTCGCGTCGCACGAGCTGCACGGCTACG
CGATCCGGGTCGATGATGCGGAATGCGGTCACGTTAGCTTTAAGCGAGTAGAACTCGCGT

AAATCCAAGGTGTGGAAATCGAGGCCCATGACAATATAGTCGTGGTAGAGTGTCGTTTGA
TTCACCTCCTCCGCCTCTCGGAATAGCTGCGGCACTTTGGTGAGTTCAAGGTGTAGAATG
ATATTGTAGATGTTTTTACTGCCGATGTCCTTGAGCATCTTCTTGAAGGATAGCGCGGGG
TTGTATGGGTACATCAGCACATTTGGAGGATTTAAGGAAGCGTTGAGCACATCTTTCAGC
GCGACAAACACATTGTCATTATCGTAGAGCACGGCGAACGACTTCCACTTGCGGCTCTTA
ATGAGATCATAGTAGGCCTTATTCAGCTGATTTACGGCCGGGTGCAGCTGCAGTGAAAAT
GGACTTCGTCGTTCACGGAATCCTCCGGCAGATGTCTTTAGATGAGGCACGTGAAGACGA
GCGGCCGCAGATGCCACGTACGTGGCCACTTGCCACGACGTGGGGCCTACGAGCGCTACC
ACTCCTTGCCGAGCCAGTTCACATACGCATTTCGAAGCAACAAAACTGTCTCCCGGATTT
GTTTTAAAGGAGATCGACTGCAGTTCCCTACCCCTGAGTAGGGCTCCATCACGATTTACC
CGCTCCACAGCCCGCGAGAGATGCTCATCGAGATGGCTATTTTCTCCTTGGTCAAACACG
CCGCCAATAGCAAATGCGTCCGTTGACGACTTCGCGCCGTCGACAGCACTCGGAAGACAT
GCGGCGGCTGCCAAGACAACCATTAGGGGCAAAAGAGCGTTGCCCATCCCTGTTCCGGAA
ACAGTATACAGTGACGTAGCCACGTATCGACTGGCTAAACGATGTATTAGCTGACGAATA
CAAACTAAAGGCTCGTCATTTCTAGCAGTGACATCTAGCGATCTCTGTCGATTTAGTCTG
TATCTTTTTCTTGGCTTTGCTTCCGGATCACGCAGGTATTTGCTTTCAGCTTTGCTGGGC
ATATGTGCGGGCCAAACAGGAGATCTCCATAGGCTTAAGAGTTCTGGTTTGCCGATAGCA
AGCTGCTGGCTGTTGTGATCCAAAGTAGACGTCGTCGAACTGTAATTATCATCGTCGCCC
CCCTTTTGCTCGTCGCCGTCTTTATCATCGGCATAAACTTCATTAGAGTCATTTCTACAA
ATGTTAGAGATCATCCCTCCGTGATTTTTGCACGCGTTCAGTACTGTGGACGTTGTCGTT
ACTGCTTTGCTTTTGAGCGTTAAATCGTTATGATTGTTTGTCGTTATCTCCGTATTGTAA
ATCTCTAAAAGGAAGACATTCACGTCGATTTGTTTTTCCAGTGAGCTTCTCGTTGGGCAT
AAAAAAGACGAAGCAGAAGTTTGTATACTATTGACTACCATCGACGATAAGACGTGATCT
CGCGATCTAGGTTCCAATTGGTAATCGATAAAAGGGGTTCTCGTATCTAAGCAACGACAT
TGAAAACGATGCAGATTCCTTAGGTTTGATACACGATCCCCCGGTATGACCCTTTGACCG
GCTGTGACCTTGTCGAAGTCTTGACTGTTTAGCAAATCTTGATAATTTGTTTTATTCTTT
TTAATGCCATACTTATAATAACTATAACGATTGGTGTCATTAACACGCTGATTCCACTGG
TTGTTCATGACGGCCTGGGTTTTAGCAGAGCCAGCTTTTAATCTCAGCTCTAAACGTGGT
ACTTGCCACGCTGCGGTAGGTCTGGTTTTTGTAGCCGGCGATGATTGCGGCCAATCCGAT
ACGTCGTCGGAACTCATTTTTCTGCTTTCGTCAGTGTCAATTAGGCGTATTAACACTACG
TCGATTCTTCTCTTTTAGCATCGTATCGAAAAGCAGAACGCAAAATGATACGGGAACGTA
AAGCGCAATTAGTTTAAGTTGAGATGGTAGCTAGTTTTGAATTGAGCCACAGTAGCTCAT
TTGAATATTAGTAAATTGTTGAAGGACAGTTTGTGCTGAACAGAAAAGCACCCGAGGCAA
ACGGTCGTCATAAAAGGGATAAAAATGGTATTAAGCCGATTACTATTCTTCACACTGTTG
CATACACAAACTGATCTTGTTCTTCTTTAGCCTGACCAAAATCGAGATTGATTCCAATCT
TTGCAACGTTTAGCGTATGATGCAACTGGTCCGAACGTGTTAAGGCAACTTTCTTGCGAC
GCAAGTTATTCACTACGGCAACGTCTTTGCTGTTCTAGTTTGTCGATACTTCTGCTATTG
TATTCCTGGCAGGGTATCTCGAAAAGTTGACATTGCGACCATTACGGTCGTCGGCTTCGC
GTGATATATGTATATAATGGCATACAGTGATAATGTAATTACCAGAAATGGTTGTCGCTT
GGCAAATAATTACTGAATCATCACGCTCGCGTCTGATCGGACAGTCTAAAGCGGTCAGGC
ATACAGCCGACATACTATTTACTGTTGAAAGAGGGGAACACTGTAGCTCACCATAATTTC
CTTAGGGTCTTGATGGTCAATGGTAGACCATTAGGTGATTTCCTTCAACGTACGAAAATA
AATAAAGGATAGCACTATACGGATGGTTAATAAAGGCGGACTGCATTAGGTCTCCAGAAC
AAAGCAAACTTGTTGAAGATACTTGGATCGATGGTAGGTTTGTGTACAATGCTTTCTTCA
ACTTTTATACTTTTCATATCACTACGTACTCTGCCCTACGTTTGCTTCTATGTTTGTCTG
TCAAAATTGATAACTGCTCATCTTGCTATCAAGCGTGGTTCAAATCAAGGTCGAGTTCGG
TCATTCTATTGTCGAAAAATGTACTTCATCAAGCGGAGTCGAAAGTTGTCCGGCGCTCCC
CAATATTTGAGGATCACGTAAGGAGTTACAGATGACTACCCAATTGGGTAAATTTTTGTG
TTCTGTTTGACCAAAACATAGTCGTTCACTGTGAACGGAGGCGGCAAAGACGGTTGCGGT
ATGATCCTATCCTATCCTATCGATGTTATCGGCGTTTATTCTTCTTGATTACTCTTGAGA
ATGCGATGTTGGGTAATCCAGGACAACTTTTGATAGGATAATCGTAGCACTCTGTTTTTT
CTATGATGAAGCAACACACACCGGTGACGCTTTTTTCGGCAACACAAACAGCAATAACAA
CGTTATATTACATAGACATTGTATGATGAGATGCCGCGTTGCGGCTGCGGCCACATGATA
TTTTCTTGGCTTCAACTTTTTGTCGGCTCGAACTCCGCTGCAGACGCTGCGTTGGTGAGG
CTGGTGGTGACTTGCTAGCGTCAAAATGGATGCTGGTAGACTTCATTTGTAGTAATGATA
CCAGTAGTAATTGTAATAGTAGAAGTAGAATGGTGTCTTCAGTTGTAGAGGTAGTATATG
TAACATGAAGAATAGCGTAGGGCGAAGGCGGATACGGCAATGGATCATGATCGATCAGGA
TCAATTGTGCAATGTTATCTCTATTGCCAGTTAGATTTGGCTGCTTGAGAGGAAGATGCA
GTAAACGTCAAGGAGAAAAAGAAGAAGAAGGTTAAACGTGAACCTGACGAGACGGCGGCA
CAGCAGAGATATTCGAACAGTTATCTTCCGCTTTACATTTACAACTGATTCATTTGTTTT
TAGCTTATTTTTTGCTTGCTTGCTTGCTTGAATGTCTGTTTTTTGCCGTCGTCTCGTAGT
GCACGCTATATAGGGCCTACTCAGTGCCAGCCACCACTGCCTTCAGGTGATGGCTGTGGG
GTGAGTCACACGCAAGGCACTCGCGGTTCCA

>Vd20731 len=4898 path=[15299:0-425 23:426-568 7644:569-886 7961:887-888 166:889-889 10775:890-1035 15869:1036-1045 10868:1046-3057 5324:3058-4897]
AACGGAACCTATAGCGCTCAGCTCGCAAGCAAACCATAAGGGTCTGGTAATGGCCAACCA
GTACCCGGGCCGACTTCTTCCGTGTCGCCGTCCGCGAAACGCGGGTGGCGCTTCGGTCTA
GATCGAGGCTTAGCTTACTCGATTAACTGGCTTCTTGGCTAAGGCCCACACTCGAACGCG
CTCAGTCGGCGCGCTAGTAGAACCCGCTCACCGAGTTACCCTCAAGGCAACTTGCGCATA
CCTCAGCTGTTACACAAAGGAAAAGGAAATCTGGCCTCTTTTCTCGTCTTTTCTCATCCA
TGGTTTAAGCGACCTAAGGTTTTCTTTTCTGGGTTTTCGTTTGACCACCCTTTCGTGTTG
TTATCGTTCGTCCAGAGGCCATACGACCACAGCTCAGTAGGCAATAGAAACTGACCGCAG
GGTTCAAGGTAGGTGGTTAGATAGAAGGGCTAGCGCGAAGACGGAGATAATTTTCCCTGA

CATAGAAAGATTCTGCAGAAGACATTTTCGATGCGCCAATAATACTGTCGGACGACTCAG
CAGTATCGTTGTACCATAGAGTTATTAACCCGAAGGTCATGATTAACGAGTACGTACACG
ATCCGATGTCTCCGAATATTTTGGAAGGGCACCTGTCTTTCAAACACTGCGTTACGTCAC
CGTATTCTTGAAGAAAATCAGGACTGATACATGGGACCATATTTTTCGCTCTAGTTGACG
CCGTGATTACAGAAGATAGGGTTCGAGAATAGACTCTTTCTTTAAGATAACATATAGGAA
ACGACAGCTTCTTACGAACGGGAAATTAACCACGAAACATTTGGAGAAAGCTTGAAAATG
AACTGTAAGTACTAAATGTAGATGCTATTTGATTGTTTTTTCCCTTTAGCAGGAACGTTT
CTTCTGGGCAAAGATGTCGTCCTTTGCGGCGTCCCTCGCCGCGACAATAGATCCTGTTCG
AAGCCAGCTACTGCTGTCGTTAGCGTCATTGTCTATACTCCTCAGCGCGCTTCACCGTAG
TCAGGCAATAGATCATGTTAATATCGGAGGAATCTTTGACCAGGAAGATGAAGGTCTAGC
GAATCATTTCACGTTAGCAGTAGACCGAGTGAATCGGGATTTGGGTATGCTCGGCGCTAC
CAAATTTCAAGCAAAGATGTTTCGCGTTACACCAAAGGACAGCTTCGGCGCATCAAAACG
CGTGTGTGAACTTGCTCGGCATAAGGTCGCAGCCTTCGTTGGTCCTAGTTCTCCGAGCGT
TAAAGAACTAGTGAAGGCGACAGCGTTTAGACTCAACGTTCCGCACGTTTCAACAAGTTG
GGAGCTACATGAACGCCAATCACCGTATACGCTCCGGATGTACCCAGAGGCGGACCTGCT
TGCGCGGGCTTACAGAGACATCATATCCGAAAAGAAATGGAAGAAATTCGCGATTTTCTA
CGATTCCGATGATGTGCTTGTGATGCTGAAATACGTACTCGCGGACGGCTTTAACCAGAC
AGCGCCTGACGTATTATTATACGAATTTGATCCAGATTTAAGTTACGAGAAGATGCTCAA
AGATATCGGAACACGGAACCACTTCAACGTCATCATAGGGCTTCGGCCAGATAGAGTCAA
AGAAGCTTTGCTGGCTGCTGAGGGTTCTTATAGCTCGTTTTACCACTCCTATCTGATACT
CGGCCTGGACTTTCACTCTTACAAAATGGAGGAGGTCGTAATGCAACGCTATTTGGCGAA
CGTGACGATGTTTCGTCTGGTGGATCCGTCACGGTCGGAATTTCAGGAAGTCAACAATCG
ACCGAGATCCAACAGTCAGCTACCCACGGATAGCTCAATATTCGGCCATGTCGACGCGCC
AAATGTGTGGGCTCCTCCAAGTACTACGCCGCCCGATGACCTAGATGTTGAGCGACTGAC
GACAGAGGAAGCCTTAATGTACGATGCCGTATCTCTGATCGCTAACGGTGTACACAACCT
TTTGCGGATGAACATTCTCGGCTTTCAACAGTTAGACTGCAATAAAACAAACAGCAACTG
GGACTATGGACAGTTGTTCCTCGACCACATGAAAGAGTTGACGATTCGTGGTTTGACGGG
CGATGTGCGCCTCGATAAAGAGGGCAAACGCGATATGTTCACATTAGACGTACTCGAATT
GAACAAAGGAAGTAAACAGTGGTCATTCCTTAAAAAAAGTTCCTGGGATCCTACGACGGG
CATTAATAAGCCGGAAGGTGATGAAGCTGTTCACGACGTAGAGTTCTCCGTCAGCGGAAT
GATTCTCAAAGTCGTCACTGTGGAGAACGCACCGTACACTATCATCAATCACTCACTTAC
TGGTCAAGATCGTTTCTATGGATATGCGATTGATCTGATCAAGATGTTGGCTGAAAAAGC
CAACTTTACCCCGGAATTCTACATTGCTCCGGATTCAGCTTACGGTTCGCACATTGGCGG
CGGTGTTTGGAACGGAATGATCGGTGACCTCATGAAACACAAGGCTGACATTGCAATCGT
AGACCTGACGACAACGGCCGAACGCGAGAGTGTCGTCGACTTTACGACACCGTTCATGAA
TACTGGAATCTCAATTCTACTTCGAACTCCAGAGATGCAGCCACCATCGTTGTTCTCGTT
CTTGCATCCATTCTCACCCCTTGTCTGGTTTTATACGCTGACTGTCTATATTCTGATCAC
AGTCGCTGTATATGTTTTGGGCCGCTTTACACCGTACGAATGGGTGCCCTCACATCCATG
TGATCCCGATTCCGAACCGGAAAATCAGTTCGGAACTATCAGTGACTGTTTTTGGTTTAC
TATGGGCAGTATTATGCAACAAGGCTCAGATCTTGTTCCCAGGGCTGTCTCTACTCGTAC
ACTGGCTTCGTTATGGTACTTCTTCTGTCTCATCCTGATTTCGTCCTATACGGCTAATCT
GGCCGCCTTTCTCACTGCAGCTCGCATGAGTTCACCAATTGAGAACGCAAACGATCTTGC
AAAACAAACTGATATTGCGTATGGTGCAAAAGATGGCGGATCCACCAAGAGATTTTTCCA
GAACAGCAACAACACTGTCTACAAGCGCATGTGGGCGTACATGGAGTCCCAAAAGCCGTC
CGTCTTTCCAAAGACAAACGAAGAAGGTATACAACGGGTGTTAAAAGGCGACTATGCATA
TTTGATGGAGGTGACATCCATTGATTATCTTGTTGAAAGGAATTGTAATCTGACAAAAAT
CGGAGGCCTACTGGACAACAAGGGCTATGGTATTGCCACACCCCAGGGATCTCAAGTACG
TGGCATTCTGGAGCATCATTTGATCATCATGCAAGAGAAAGGTGTTCTACAAGAGCTTAA
GGACCTTTGGTGGAAAATCCCTGGCGAACCTTGCGACAGAGTTAAAGATGATTCTGCTGA
GATGAGTATGGAATCCCTCGGAGGTGTTTTCTATACGCTGTACGGTGGCGTTTTAATAGG
CGTCTTTGTCGCACTTATTGAGTTTTGGTGGGAAAAGTCCCAAGCTCCCTACGGCGAGAG
AGACCACATCTTAGTCGAGTTTCTTCGCGAGCTGAAGTTAGTGTTGACATGCACTGGTTC
ACGGCCAAACCCTGAAAGCGCAGATAATTCGATAGCCGACGACGCTGCCTCGAGAAGATC
GGGTCATTCAAGGCAGGCGAGCCAGGTGTTCGCTACGTTAAATACCTAAACTATTAACTT
GCTGAAAAAAAGTTGAACTCAATTTAAAAAGTGCTACCCCTAAAAATGAAAAGAAAACGA
TAGGTGAAATAAATATAAAGTGTTCAGAACATGATTTATACATTTTACCGGAATTTTGCT
CATGTTCAGATAGAGGAAATTATGATGCAGGTCGACGTCATCGTCTCCACAACCACCTAC
ATGATAGTAACGATAGCTCACGTACTTCAAGTTTAGCGCTACATTATTGAGGTGTTCCGA
TACGATAAACCAAATAGCGACTATCAAGTGGCTCTTGAAGATGGTTTGTGTTATTAACTT
TTGTTGTTAATTTAAAATTACATAACATAACTTTATAAACATTTATATTTATTAAAACAC
CGCTAATGCGAATGCATTCATTGCTATCCATCTCCTGCCAGTTAACATTGGAAACTACGA
TGTAATACAGAGAGTGTGCAAGTATTACGGCGATAACTTTAAAATAACAAATTTAAACCA
GGACGCACAAATATGTTGTCGACTTCAATAGACTTACGTCAAGCTGCTAATAATAATAAT
AAAACTACAAGCTCTATTTTCTACACGAATGAGCTGCTGTGCTATCAATTGCTTTCTAGT
TTTCGAGTTAGGCTAGAGTCAGTGTTTTTTTGAGTGAATTTGAAGCCGGTCAACACCAAC
AGATCTGCTGTCTTTCTACCATAGTTTATAAAATTATTCCATTTAAAAATAACAGCATAA
TCTTATCTTGCGTTAGGTTGAAAACACAAAATGATCTGTATGATTGTTTCATAAAAATTT
TATTAGGTTACTTAGCGTTCTTTGCTTAGTTATTATCAGGCCCGTGTTCATGGTCGCTAT
TACGGTGATGATGATATTCTCGGAAAAGATGAATTTAAAATATGTTGTAAATAAAAAGTA
CCGCAGTATCCACGCTGATTAAACGTAACCAGCTCGTATTAAATCGAAAACTTGATGAAC
CTAAATTGGCCGCCTTACCTATCTCGGCACCTGGTCAGCCAGTACAAATACAAATACAAT
ATACAACATAATACATCGCTAGCTGTCGCTTCACGACAAAGAAAAAAGGTACATTCGGGA
TATTGATACTGAGATTTTTACTAACTTTACACTCGCACAAAAACTTTTTCGCATACCATG
CGAAAACTATTGCTACTTTCAAAGCAAGTCAACTGAGC

>Vd20855 len=5670 path=[6262:0-1620 7883:1621-1814 @8077@!:1815-3097 9360:3098-3119 @9382@!:3120-3329 9592:3330-3641 9904:3642-5669]
TATATATATATATATATACGTTTACCTGTGAGCGAGAGCTAGATAAAACATTACGATGTC
TTACCTGTCTAGTATACGTCTTTAACACTGCTTCGTTTTAACTGTATTTTCCGCCATTGA
CAATTTGAATTTTCGAGAGTACATCACAACATACGAGCTTCTCTTCTAAGATCAGCTACA
CATTAATCTCAGTTTCCTTAGCGTGACATAATTTCATAGGAATGGTAATATTAGCAATCT
GTAACAAACGAAAGGGTTCGGATAAGTTGTAGCACACACACAGTATAAATTCAACTGGTA
CGTAGCTTGCCTCCCCTTTCCGCGTGCCCTTTGTCTAGTCGTCTTATCATGTATGGTTCA
ACAAGTCCAACCATTTACTAGTTTTACGTTTGATTATTTTTACCCGTCCGTTCGAATCCA
GCGCGCCATCTATTCAGCGAGATCATCATTTAATCTCAAGCGATAAACTCAATTTTAGAC
CATTTACTAAGTAAATTCTACTAGCCGATCTGGTCCAACAAACTACTGACTCAAATCATC
TAACCGCAAAAGATTTGTGGGAATCGTCGCACTCTATCTATGTAGATCCACTCGTACGTA
TTGTGTGTATATATATCAATATATGTATTAGCGTATCGTATAATTGCATTTTAGCTATAA
TCATTTACATTGAACGGGTGAAACGATATACGAGAACAGAATCTCCCCATAGGGTAGAGG
AAGAAGCCGGCATGACTAGACACTGATGAATTTCAATTGTGAATGTCTAAATATTGGCTC
TAGTTAGCAAGTATTGTGATCCCTCGTGTGGGGCACTGCGTCAAAAATCTCAGCAGTAAG
ATTCTATAAAAAGAAGAGATTCATATACCTACATCACTTCCCTCGAAGGTCGTTTCGTGC
CATCTGCCATGCAATAATTTTATAACAATAAAATAATAATGCATTGATTTGTTCCCATTG
ACAACCGTTGTACGCTGGATTATTTTTAACGAGTTACAGTAGCATGTCCAGTTCATATAT
GTAGTTATATGCAGGCATTTTTTTTTTCCACATTATTGTTCTTGTTCCCTAAGGTGTCTG
CTCGAGCACCGTCTGCTGTTGTTCCACGTATACTACGATTCTTTAAAGCCAAACTGTCTA
ATAATGAATGGAGAAACAGCAAATTTGCAATAATAAAACGAACCGAAAACAATTTAACTG
ACTTTCTGAGCTGTTGGAAAGCTTTGAAAATCCTGCAAACGGTTTCTGCCAGAGAGCTGT
GTCAGGTCAGTACTCTGCGTATGAAACCAGGCTGTCTGTTTAATAAAGTTTCGTTATTAG
TTCATTTCCTTGCGAAGGCGATCAGCTGTATGAAGCTCGTCTCTCTTTATCACGGAGAAA
TTAAAACGGTACAATGTTTACTTGGAGAGATATCACTATTTTGTCTAAATTATGACGATT
CTTAAAAATTATCAGTAATTTCTTGCAATCGACATAGTTGAAGAAAATGCAGCAGTGATT
CGCGATCCGTCTTCAGCATTTTGCTTGCACTGAATTATTTCTATACCTGAGTGTGGGTAT
TGACAGATATCCGGTCGTCCAGGAATTCCGGGTTGATCAGGTGTGGTCCCGGGTTGTGAA
AAATTACGCACGCTGCTTAGATTCAAACCATCTTGATTGTACAGGACGATGTCTTGTTCT
TTTGTGATGAGCGTTTCGACAAATAGCTGTAGTTCTTAGTGCTTGAGCGCCGTCGTCAGC
CACGGAAGCTCAATTCCCGGGTTAACCAGGTGACGATGGAGAGTCTCCGTAAAGCGCCGC
TTCCGCCAGGGATTTTCTAATGCGATCCTCGGCCAGGCCTTGTACACCCACGCCCCCTGC
GACATTGCCACCACCACTTGGACCCGTCCCGTTCTGGGTGCCTGTGATCGACATCCGGAC
CTTTGCCTTCATTGCGTTATACATGGTCGTTTTTGATCGTGTGGCTTCCAGGCGCGATTT
GTAGCAAAATTCTAACAGGGCAACAAGCATGGCTAGCACGAGACCTCCTATAAGGATATA
GAAACAGCCGGCGACATTGCTCAGGGTTAACTCGTTTTGCGTAGACTCCTTGTTAGCATC
TCCAGTCTTGCACTCACTGCGGTCATACCACCACTTGTTTTGCAGACGGGCCAAGTCGCC
GTTCTCCTTGAATGAAAGAACCGCGAGGTTTAGCTGATCCCTGAGCGACGATCCAAGCGG
CGTTCCGACGCCGTAGCCTTTGGCATCAAGGTTTCCGCCGACTTTCATGGTATCGCAGGG
ACGGCGCTCATTGATGTAATCGTTTTTTGTAGACTCCATTAAAAAGGCGTACTTTCCTTT
AGATTCACGCACTCTTTGAATACCTTGCTCGTAGGTTGCAGCAAACACGTTTTTTCGAGT
ATGCATAAACTCCCACATACGCGCATATACGGCGATCTTCGATTTCCTAAAGAAATCTTG
CGTAGACGAGGATTCAAGTGTGCCGTACTCGACCTCCGTTTGCTTAGCGAGATCGTCCGC
AGAATTGATAGGCGTCACCATGCGTTCAACAGTAAGAAACGCAGCCAAGTTGGCAGTATA
CGATGAAATGATGATTAGCGTAAAAAACCACCAGACGCCCCCGACAATACGGCCGGATAC
TGATCTTGGACAAATGTCACAGCCCTGCTGCATGAACGCCCCCAACGAGAACCAGAGGCT
GTTGTATAAAGAGAAGTCGTTGGAGACGGTGGGTCCGACGAAGGTCTCCTCGTAGCGCCA
TTCATGGGGCGAGAATCGTGAGACGAGAAAGAGAACGACGGACACGCCAACGTACGCGAA
GATGATGCACATCCAGATCTCACGTGATAGAGGATTCATAAACGAGAACACGCCTGGCTT
TTTTTTCATTGGTTTTTTTATCATAATGCTGATACCCAGTGACATGAAGGGTTTTGTGAA
ATCGATTACTCGTTCACGCGCTGACGTAATTGTCAACGGGGCGATAGCCATGTCCGCTTC
GTTTCTGATGAGCTCTCCAACCATTCCGTTCCAACCGGACTCCGAGTCGGCGCTTTGACC
TCCATATTTTCCGTCTTTGACTAGCCGCAACTCGTACGTAAAGTTGAGCTTTTCGGCGAT
AAGGTCGGCCAAATCTTTGCAGTAACCTTCGAACTGCGAATTGCCGGTAAGATTAACGCC
AGGCTCCATTTTGCGTCGCATTAAGTACGGCTCTTCAAGAATGCTGGTTACGATGTAAGT
TTTATTAGATTTGAACCCGCTGTCGACAAAAATCCTGCGATATTTGGGAGCGGCAAAAAC
AAACCCCATCTGTGAATTCCATTCGGCGATTGTGGTGAGTTCACTGTTGACGGTAACTTC
GACAATATCAACTGTGTAGTTCGACCGTAATCCGTACGGGTCAAACGAAATGTTCCCGCT
CAGGCCCCGTATTCCTTTGATGTTGCGCAACCTATGGAAGATCTCATTGCCGTGTTCCCA
AGGTATAACTTTCTCCCGCTTGCAATCGATGCCCCGCGTGTTGTTATTGTACACTTCGCC
GCGCCGAAAATTCTTCGCGAAGATCGCCGGGTATTCGTTGAGCAGCTGCTTAAATGCACC
CAGCATTACTCGAGTGCCGTCGTACATCAGCGCTGCTTCGGAAGTGATCGTTTTGGTCTT
AGGATCAGGCCATTGATGACGACTCTTTTCCGTAAATACTTCCTCGTAGGCGCGACTATT
GTTTTGAACGAGGCGGAATCCAGTGATATTCACCGCTCCAAATTCTATAGCCGGATTGTT
AGCGAACTCATCCATTATCAATCCACCAAGTAGAAAATGAAAAGTTCTTCTGCCCACGAT
AGGGTGTTTCACATGCTTAATAATGATCTCTCGCGTCAGGTTAGCCTCGCAATCGATGAC
CACATGCTTGAGTTGGTCTCGAGTCTCATTGTCGTAGTTGACGAGAAACTTGTTCGCCTC
AGACGCTGATGTCACCCTTCGAACCTTAATTATTTCCAGCTTGTACTCGTCCGTCGTCAG
CTTGTAGAGGTTTTGCAGCTTCAACAATCCGGCGTCAGAATCGTAGATGTAGACGATTTG
TTTCCATCGATAATGCTGTATGAGGTCAAGAATCGCTTTTAGATAGTCAGGCCGCATTGA
AATACCGTACCTTGCCGGCCGGAGCCTGGTGTGATACGGAAATGACGGACTAACAATAGG
AATTTGAATGTATTTGAATAGGACACCAGCGTCTCGTAAGATGAAGCCGTTGTCGTGGC

GACCATGGCGAAAGTTCCGTTCTTCAGGATGGAGCAAATTTTTTTAGCGACCTGAAAAGG
ATCATCAGATTCTACTAGTTCCACATGCGGCGCCACCGAGAAGTGTGAATCGCGGTAATT
GTGCACTTCGATCGCGTGCTCGAACGCCGCTCGTAACTCTGTTTCTCCCTCTTTTAAGAA
GATCGCACCAACGGGTATCCTGTCAGGCAGTCCTTTGGCGGCTCCGACGTGGTTTGGAAG
CATCAGCAACGAACAATCTATCACGAACACGGCTACCGCCAGGTGCCACCGGCTGTGCCA
CCGCGGCCAAGTTTTGTTGATCAAATTGGGCAAACAAGAAGAAGAAGAAGGTGAGGACCA
TTTGTGTCGGACCGAAGGCCACTGTCCGTGATTGGTGCGAGTCACATAGGCCGCTTCAAC
AGAAGAGTTGGATCTAAGTGGGTGTTCTTCAATTTGGCGACGCGGTGACGTGCCTATTTT
AAATGCGGCATCATTGGCCTTTAATGGCCGCTGCAGAGTAAAACTCCTACATTTGCCCCA
AGCGGGGCCCCGGTGGTTTTGGTAGGTTGTCAGACGGTGGATGTGGCCTGACCCATTGAG
AGTGCAGGACTGTCCTGTCTTAGAAGATGTTTCAATAGTAATATGCATCGGGGGACTGCG
CGTAGCGGAATTATTTGTGGAGCAGCAGCTTCTACTATGCGGCAGCAGCAGCAGCAGCAA
GCAGCGGAACAACGCACATTCGAGCCTACGTTCGACCCCTGAGAGAGCTACGATTCATCC
AGGAGTGGCATACTGAAGAAACGAAGCTCGCAGTACTGAGGATAGAAATGTATAAGAGAT
AATCAGGAAATGCTGCTCTTTCTAAATCTTGTGTGCTTCTAAATCTTTGATGTAGTTATT
ACGTCTTCGGCACGTTCCTCCAGCCTCTAGGTGACGTCTTCATTCACCTGTGCCTGCCTT
TCTTTGTTTCTACTTTGGTCCTTTTTGGAACGATCTCTTGCTACGAATGTTCTCTTTCTC
TCGGTCGTTAGCAACACTTGGCAACTTCTTAGTACTCCTTCGTCTGATTTTTCACGGCTA
TATATTGTAACTTAACACTATGATAGGCTAATGTTGTTCCGCATTTTTAGCATGTTACGC
GAACCAGGTTCGATCTATGTATCGAGCGGTATTTCCTACTTTAGTCAGCTGGTTCATTTC
AGTGTGATCGAGACCGGGATCTTCCTGACAAAGGTCGCATTTTCTCGCCTACGCTCTTTC
AACAACTAATCGATATAGGGAAAAAAAAACGGAGAAGCAACGGTTTTTAGTACTGCTGATT
AATTCACGACAAAACTCAGTGAACAAGTTGAACTGCTAGACAAGGAGCCTGGCGGAGGAA
CGGGACACGTCCTCGGTCCGTGTATCCACT

>Vd21106 len=4056 path=[53:0-79 133:80-2372 2426:2373-4055]
GAGAGAGAGAGAGAGAGAAAGAGCCAGGCAGAACAAGAGGGAGAAAAAGAAATCATCTCA
AGACAGTGTGGTATAACATCAAAAAAAAATATTAGGTGACAGCAATTATCCCGGCTCTAC
GTCCGTGACACTCGTTGGATTGCTATTGCCATTGTTGGAGAGTGCTTCCCGTTGTGGTAA
TTGGCAGTCGTGTACTTGCTGTCGTGTGTGGCGTGTTTTTTGTGCAGGACCATCCGTACT
TGTACTAGTCATCTATAGCCAGAAAGGTCCACTTACATTGAGTCATCCCGGTGTAACCAG
GGTTAGTGATGCAACTGCACTGGGTGTTAGTGGGGGCTCTCGGAGCACTGGCGTACAGCG
CGCACGAGGTGCTCGCCAAGGAAAAACTTCGGATACCCATCGGTACGATTTTACTGCCGA
ATCAAGAAGTCTATGAGAACATTTTGCGATATGCAGTGAAGAACTTTGACAGCTCGCTCT
TTGACACTCGGCTTGTCGCGATGAGATTGAGTAACGCCGATTTCGTACAAATCAACATGA
AAGTGTGTGCGATGATGCGCGAAGGTGTCCTCGCATTCGTCGTAGCTCCTGATTCCGCAC
CTGCACTACGGGAGATGCTCACCTCGTATTCATCGCACTTTCACATCCCGTTGGCAACGT
CACTTGCTTCACGGAGCTTCTCTCGAAGCGAGTACGCTATCTCGACGCAAGTGTCGCTAG
CAGACGCCACCGCATCGCTGTTGCGCCGCTACAGATGGTCACAGGTGGCCTACATCTTTG
ACGACTCTGCCTCCCCCGATATTCTAGAGGCAATCCAGAACTCTGGTGTGAAAATTTCGA
TCGCGAAGAAAGTAAACAATGAGGCCGAGGCAAGTGACGCTGTTCTGGAGCTGTCTTCGC
TGGGCCGATTAAAGCCGGAAAAGATCGAAAAGATTGTACTCAATGTTAACCGGCCGAGCT
TGGCTAAGACAGTGCTATCTGGAATGATCAGGGAAGTCAAGAGCTTCAAGAACCTCAACA
CCCAGTTCATCCTCGCCAATCCGGTCTCGGACGATTTCTGGCAGTACATCCGATTCCGCG
ATGCCGGGAGCAGCCTCAACGTGACCGCCTTTCGGCTCGTCGACTCTGAGCTGCCGCATG
TCACGCGCTTCCACAAACAGATGATGCAGCTGTCATCGCACCGATCCCTATTTCACAACC
AAGGCACTCAGTCATACTTATTTCTGGACGCAGTTACTGCGCTGCTTACTGGCTTCGACC
GTGTCCTCAAGGACAATCCTCGCCTTCTCGACCGCAACCTCCGTCACCATCCCGGCTCTG
TCTACTTTAATGGCACGAAGGGACTCGACTGTATGGACCAGTCGCGTTTTTTCGAGTATG
GTGACATTCTGGCAGCGTTTATAAAGCAAATTAAGATCCCACAGGGACAATCAGGTCCTG
TCGAGTTCCATGACAACGGTGAACGTGTACAACCTACTATCACAGTAATCCAAGGCTCCC
ACAAGGGCCACGTTCGTCTGGGTACATGGACCGCTAAAAAAGGACTCATGCTTGCTGACA
AGCCTGAAGCACTCCCTCAGATTAAGGCACCCGGAGCTATTACTCCAGCTAAGGAGCTCA
GCGTCGGTAGTGTCTTGAATTCGCCGTACCTCATACAGAGACAAACAGACGGCGAATTCT
CTGGCTTCGTGCCCGATTTTCTTGAGGCTCTATCCAACGTCGTCCCCTTCGCATACAAGA
TCCAGGTCGTACGTCAACGCGATTATGGACGCCGATCTGAAAATGGAACTTGGAGTGGAC
TCATGGCTGAGGTGATCAACAAGAAGGTAGAAATTGCCGTATCTGACATGGCATTAACAG
CGGAACGTCAAGAAGCCGTCGAGTGTACCCAACCTTTCTTCGTTGATGACCTAGCCGTTG
TAATGGGCCGAACCACCCCCGCAGGCCGGCCATCGCTATCCTTCTTCTTTGTACGGGTCT
TCTCCTGGCAACTATGGGCTTGCGTCGCGGCTATACTCGCAGTCTTTATTATCTTCTCAT
ATATACTGAATCACCTCGCTCAGTCTCGGCAAAGGAACAGTTCGGAAAAAAATCGACTTC
TATGTCTCGTCTGGTTTACACTGGCCTCGGTGTTCGCCCGTTCGCAAGGACCCCATGTCA
ATTCGAAGGCTTACAGGATCCTGTTGGCATCGTGGTGGTTATTCCTGGCGGTAATGACCA
TCACTTACTTATCAGCGTGCATTGCGCTCCTACAAGCTGTCCTGTTTGGCAGTGGACCCG
ATCTCTTCAATAAAGACAAATTTTGTCGGGATGCTGCTAACGGAGCCGTTGACGTCGGGT
ATATGCGACATGGAGCAGTCGAAAAAATGATCAAGAATCAAGCGGAAACGCAGCTTTCAT
TTTACGCACAGCTGTTGAATAACTCTCGGATCCGCATTGTTTCCGGCATTGCTGAAGGAA
TGCGTCACGTGGGACCTAACTATGCTTTCCTCGGACTCAAATCGCAGGTGGATTCGTACA
TCGGACGCGATTGCGGTATAAAGTCGTATACGCTCATTCCACAGTTCGCCCAATATGCTC
TGGTTATGCCCAAGATGTCACCGTACAGGGATATGTTCAACCAGGGCATTGAGAAGCTTC
GCGAGTCTGGCGTGCTGGCTGATTTAAAGGAAAAGTGGATTGATTCACTAGTACGATGTC
CGCGCGAGGTCGAAGTGCTGGAGGTGCCAGCGCCGATCAGGTACACAGCCCTTTGCGGCA
TGTTCATAATCGTGTTGGCCGGGTTCATACTGGCACTTCTCGCCGGAGTCATCGAGTTTT
GCTGGAGCGCCAAGCGTGATTCAAGCAAAAGTGAGAAACCAATTCAGAAAGTGATGTGGG

ATAAGGCGCACGATTCGTTGAAGCCATTTACCCAAAAGGATCCAGCTTTGGGGAACGACG
AGGAGATGACTCTCAACCCAAGTCAAGCTAATGGCACCCCTAAACAAGTCCGGGTTATGG
TCTAGAACACCTAAACCCAGCATTCGTCTCCATCACTCACAAATCTTTTATGTCTTCTTT
CACTACTATAACTCCCTCGCCCGAACTAGTCGTATGTACGTAGTGAATCCAGCGCCGTGT
TGTACCATGTCTTAGCCATGTATCACCGCCATCATTTTATGTACCTACTCATGTCTTATG
CGAAGACTTCGATAAATACAATTTCATCGTCCACAAACATTCATGTTTGCACCTTCTACC
AGAGACCAGAACGAAAGAAAACATTTGCACAACATCTGACTCAACTGACAAACCCCGATT
AATTGTATAGATGTAAAGCCTTCTCGTTTGACGATGCAGATCCAGCTGTGACGATCAGAT
ACGGTAACAAAGACTGACGCAATGCACCATACCTAGTGCGTTACATCACGTAAAAATTGA
ACCAGAAGCAAATCAATAACGATATTTCTAACAGATAATTAAAAATATAGTCCCAACATA
TATGGGGCAAGCAGGAGTATGATTGACCATATAAATGCACCTCGAACTGAAACCCAAATA
TGGAATAGAAAGCAAATCTACAATCCAATATGTAATAGCTATCCGTAACGCATTTGAGGT
GAACCCAGAAATTAAATAGTGAGCTAATCCAATGAGAGAAGGTTTCTACGTTCTATACGC
GAAGGTATTGTATATGTAGTTAATAGCTACCCTTCTGTTCAATATGACTCGAAGGTGAGT
CAGCGGATCTGCCGTCTGACGTAAAGCTGACTGCTCGTGTGCCGTCTAAACAATCGGAGC
ATAGCCTATGTGACTAGAGCTGAACACATTGAGATGTTAAGTATGCATGATGAGGACAGT
CAGTCATCATGCATCGTGTGCGGTCTCGGTTGAGCCAAATGTGACAATAACAGCTCCTCA
AGCATGATTTTGTCGCCATTACCCTCAAGGGAAAAGAAAGAGCTTGCGCGGGTGAAAACT
AACGGTAAACCTACGATTAGAGAAGGTATTTAAAACCATACCTTCAAATTGTATTTGCTG
TATATATCATGTACTTACATGAACTTCGCTGGAAAA

>Vd21675 len=4956 path=[1:0-1556 1558:1557-2475 2477:2476-2636 2638:2637-3234 @3236@!:3235-3877 17566:3878-4013 17702:4014-4014 @3879@!:4015-4335 4200:4336-4937 18420:4938-4955]
CTAAGGAAAACTTGTTATAATATTTTTAATATCGTTGTCTACTTACAAATAAATAGTGCT
AGTCACTAGCGTCATTTTTGAGGTTACTGGTGCATATGTACATATCTAGTGTATACGAAT
ATAGAGACTCGTGTTAAATTGCTGGCATTAGTAGAAATTCTGGATAATAAAGATAATAAG
TCGAAGCTTTTTCAGGTGGTTTTCATCATATATTGTGTGTGAACCTAGATTACCGATATT
TCAAGGACTATAGCAACTCGGTCATACGGATCTTCGCCCGGTCAGGAATAAATCGTATAG
TCTTATGGCAATAGTTGCGGCAGCAGTAATAGAAAGAAAACGAGGCAGTCAGAGAAACAA
AGAACTGGCCGCCTTGCGGACGCGGCGTCGAAGCGAATGAGAACCCTGACTCTAAGGAAG
GGTACGCGGAGAGGGTTGCTTTCCACATTTGCGGTGGAAAGGGCGGTGTCGTAGGGTAGT
TGCTCCAAAGAGGCAGATAGGGAAGGAGCGTTACGCAAAACACAGCCGACGGAAGGATGC
TCTAAGGCCTATAATGCGTCACGACCATCAATTTTAGCCTATCGTTATAGTCACACATAC
AGATGTATATGTGATAAAAGTATGTGACAGAGGAGTCTGTGTGGATCAATATCATTGAAG
GCGCCAAGACAGCCTGGGCATTTCCATTAAAAAGAGGTAGGAGCGATCGTGCAACGTGTT
AAGTGAAGTTTCGTTATAATAACCTCAGGATAAAACGGAGAAAAGCATCAAGACGAAAGG
ATTAGCGAAAGACCATCGTCCAAAAAGTGATAAACAAAAAATGGAAGACAATGTTAGCTA
GCTGTTAATGAAACCGATAGGGTCACCTTTTAGGGTCCATCAACAGTCTAAGGAAATACA
AACGGTGATGGATGGCATCCCTACGAAATTCGTGTACTATCAATAGAATCTCTAGAATCA
ATAGTAAGCTTGTGTCTGCGAGAGAAAGCAGGTGAGATGGTGTGATTCTTTACTTTACAT
CACCAGCCTTGTGTCAAGCAGTTAATATTTGTATCTGTAGGCTACTGATTAACCTACACG
CTATATTTCAAAAAGACGCGAGAGTGTAGACAGTCCTTTGAAAATGGACAGGTAAAAATA
CGTGTTTCTATAATATGAAATTTCACGTCTTGTAAGAAATTCAATATAAGTTTTTCAAAC
AACGATTTGGCATCAAGAGAAACGCTAAACGTCATTAAAGTTGCTTCGGAAAAAGGGTGT
GATTATGATGCGGTAGAAGTATACGGTCGTTGCGTATTTCCGCAATGGAAGCAATGTTGG
ATAGAGTGATTACCGCCACGGTGAGTTACTCCTGCTGCCGAATGTCGTACGAGGAAGTTC
ACGCGGGATAGTTGATGGGCAACCCGCAGCCAACACACTGAGGGCGAACAGTTTTCGTGC
GGAGTCCTGTTCTGCGGTGAGGGTGGATCAGGACCCCGCTCGCTAACAACCGCGATGCGG
TGGATTGCATTCGTCCTCGTTCTTTGCACGACAGTGCTCGTTCAGGCCGGAGACCTGCCG
GTCATGAAAATCGGCGCTCTGTTAGATGAGGACGAACAAGTAGCTTCCGGAGCACTAATG
GATACACTGGTAGCCCGAATCAACGCTGACCATAAAATACTGCCCCGATTTCGACTACAT
GTTGTCCGCGTAAAGGTCACAAATTCTTTTGCTGCGGCTAAAGCCACATGCGCACTGATT
GATGACGGTTGTGTCGCACTCATTGGACCGACGCAACATGAGTCGCACAGAATGGCTCAA
GAGATTGCGGCACGTCTTCAGGTGCCTTTTTTTGCGCGCGGTCGGCCTGGGGCATCGGTC
GGCATGACAGGGGCGTCTCCAGCAGGTGGAGACAACTCCGGACCATCCTCTTCTTGTTCT
ACTTGCTTCGATCTGCTACCGCCATCACGAGCGCTCAGCCGGGCCTTCGCCGATATCCTC
AAAGCTAAGAAATGGAAAAACTACGCTCTGATTTACGAGAAAAATCAAGACCTTGTCGAG
CTTGCGGAGCTTATTAAAAATAAAAATATTCTGCTTTATCAATACCAACGCGGAGAACGG
GGCACGAAAAGACTCATTCGCGAAATCGGCAGTGACCCGCAATACAACTACATCGTTCAG
TTGCCCGCCGATCGCGCTAACGACTTCTTCCGTCTTGCGGGTATTGTTGGTTTGTTATCT
GAATACTTTAGCTACTTCGTCACTGCGCAGGACTTTCATACGTGGCAACTACCAGCCGAA
GCACTGAGCAATATCACAGCGCTCCAGCTCCTCCACCAGGAGTTTGTTTCACTACCGTCG
GATAGCAACGGAAAGCGTCAACGAGGCGGCCCTAAACAACAACAGTTTATTCACCAACAT
CAAGCATCGCAACAACAGCAGTTCCAACAACAACAACAGGCCCAGCAGCAGCTTCAAACT
CCCCCTGCCCCACTTAAGTATCACACCGCGTTGCTGCACGACTATATCTTCGTGTTTGCG
CGTGCAGTCGGAACACTTTCACGTACGCAAAATATGGCTCCCGTTTCCGACGCGAGTTGT
CACAAGCCAAGGAACGGCTGGCCGATGGGTCTGACAATTGCTACGCAGATCAAGGCAACA
TCGATGCAAGGACTATTTGGAAATATTCAGTTCGATACATTTGGAGCGAGATCGAATTTC
AGCGCCTTCGTGACGGAACACAAACATGGCACGATTGTTAAGGTGGGAACATGGGCGCCC
TCATCGGGCTTTAGCATGACAAGGAACGTATCTTGGGACCGAGTCCACGCGGAGCATTCG
TTGCGGAACCGGCCTCTCCGCGTCGTGACGCTCCTCAGTCCTCCGTTTGTGATGTGGCGC
CAACAGCAGAATTCAGCAAAATCCGAACGAAACGGCGATTTGGAGGGGTTCTGTATCGAC
CTGCTGAATGAACTTTCCCGAATGCTTCAGCTCAAGTTTGATATTCGGCTTGTCAACGAT
TCGCAACACGGTAGTCGAGATCGCCAGGGTAACTGGAATGGCCTCATTAAGGACATTCTC

GATATGGAGGCTGACGTAGCGCTAGCTGACCTCACGGTGAACGTCGAAAGGGCGCGGGTC
GTTTCCTTCACGACACCGTTTTTGCCCTCACAACTGGAGCTGCTTTACCGGCCGCCTTCA
ACAGCGGGCGATCTCGTTTCTTTGGTCAAAGAGCTCTTTTCGCCACTATCTCAGGAGGTT
TGGCTATCTGGTGTAGCGTGCGTGTTCATGGCTACATTTGCCATGTCATACGCATCAAAG
TTCAGTCCTCGGGATAGGGTCCGCCGAAGCTCTTCGAAACAGGCAAACATCCAGACCTGG
ACAAAACCAAGATTTTCGTTTGGAGACATCTTTCATCTTGTTGCTTGCTGTGCTCTGAGG
CAACCGCCAATAAGCAAACGTCCACGTGGCGTAGCCACACGTTTCCTTACGTCCGTGCTT
TGTTTTACATCGTTCGTCTTTTGGTCGGTGTATACAGCGAGACTAACAGCACAATTCGTG
CAGCGTAGGATGCATTTCGACGAATTGCACGACGCTGGTGACCTTCTTAGAGTAAAAGGC
ATTCGTTTTGGATACGTGGCCAACACTTCTTCCCAGCTTTTTCTCCAGGGCGCCAATTAT
GAACCGTTCTCGACAATCTGGTCGTTGATTATGGGTGCAGGAGACATTTCGACACTGCCA
TCACTTGAAGAAGGACTTCGTCTGGTGCAAGCCGGTGGTTATGCCATGTTTACAGAAACA
CCAGCAGCTGAATACCATCAGTGTTCTATTAGCGATTGTTCTGTGCTTCGTCTGGCCACA
GGCATCGAAACACCTGGATATGCTCTGGCTTTGCCAAGAGGTAAAGCGTGTATGAAAAAC
ACCGTACCTTAGACTAGCGTAGGTCTGTGAAGATCCACAAAAAGCTAATGGCACCTGCAT
TCTTAGCCTAATATTACGACTTTTCCTTTTGTCTCAAAATCGTTTCTGTTTGAACAGCGT
CCCCCTACACGGAGTTATTTTCGCAAAGCGTACAGAAACTGCGGGAGAAGGAGGTGATCT
CCCGGTTACAACGCAAGTGGTTTCTAACTCCGCACAGTTTAGTCGGAGCACCGCACCCAT
TCTGTAGGTCAGAGATTTACCGATGGGGCCAACTAACCGCCCCTGGCTTCGCAACGGGGC
CATTTATTCTTCTAGGAGCAGGTGCCCTCGTTGCTGCATTACTTGCTCTTATCGAGTGTT
GTTGTATCGCAAATGCGCGAAGGAAACGATCCCGCAAACAGTCGAGCAACAGTGTTGCAA
ACAAAAAGCCCAAAGGTCAGCAAACCTGTGAGCCGAAACCACTTCCTAGGGACGGCCCGG
ACGATAACACCCAGATTGTAGCTGCAGTAACCACATTGTTGGCCGTGCCTTCGGCAACAG
GTCCGATACCCCCTATGCCGCCGGCGCCTTCCGAGGATAACATCGAAATGATGCGGCAAT
GTCGCACGTTAGACCTGCATGCATTACCTAATATTCTGCCACCGGCCACAATGGACCTTT
ACAATAATATCAACACTATGCCTATCAGCGCCACCACGGCTGAGTCGGTGATTCTGACGG
GCGGTGTTCCCGCAACAAGGTAGCAGAGGGAGGAGGGCAGCGTAGTTGTGGCTAACACAC
TGCCTTCTAGCCACAACCTAAGTTCACGCCCCCAGGAGCATGTCAGAACGGTTAAACCGG
TTCAGCAACAAACCACATCGATGCAACAACAGCCTCAGCCAATATGCGCCGCTACGGGCA
CCGACAAATCTCGCCTACTACTGCTCAAGACATTCATGTAAAAAGAGACGACATGTCGTA
ATGAGTTATTTAATCAAAAAAAAACAAAAATAGAAAAACGAAAACAACAGGCCTTCAAGT
AGCTTATTGCCGATTGGGAAATTCGCTAATCTCATG

>Vd21743 len=8234 path=[4623:0-98 14090:99-2604 16595:2605-3097 17088:3098-3262 17252:3263-3547 @4185@!:3548-4534 4370:4535-7912 75:7913-8233]
GCTCAATCCAAATAGACCTAATGTCACATATGTGATGATTGTGCCGAGTACGCGTTTTGA
AAGTGTGCGAAGAAAGTACCGTAGTACTATAAACGAAGCGCTCAACCAAATAAAGAATGG
AAAAGTTCCCGGCAACCACCTCAATAAACATTACTCCCTACAGTTTCAGCTGGTGTACCT
GAGTTTGGCGCCAACGCCTCGCGAGATCCTAGACACGCTCTGCACGGAGATACTCAACGC
CTCCGTCCTCAGCGTGGGTTATTTTACCAACTCAGAGACGTTTGGTTCCAACGCCGCTTC
GGTTGAATACGTTCACCAACTACTCGGGTACCTTGGCATTCCTGTCATCTCCTGGAACCC
TGATAACATCGCTCTTGATGAGCGGGTGACAGACGCTCACATTCTGGGGTTGTCGCCTTC
CGTGGACCACCAAGTAAACTCGATCTTCGACTTCCTGGACCGCTATCAGTGGACCCAATT
TGCCATCATAACGACTCAGCTGGCCGGCCATGAAAATTTTATTCGCGCCATACGCGAAAA
GGTCTTCCACAAACAACACAAGTATAGCCTGATCGCTGCTCACACCCTTCCTCGAGGCGG
CGATGCTGATAAATATTTTAATCTGCTACAGGAGCTGGCAGACGGTGAGGTACGAGTAAT
CGTTCTATTTTGCGGGACAGAGGATGCACGAGAGATCTTTCTAGCGTCAAGTAGACATGG
AATCACAGGAAAGAATTACGCATGGATTGTAACGCAGGCTGTGATTGGTTCCGCGGAGCG
ATCCCCTGCCGAATTCCCAACGGGACTCATAGGGATGTACTACAATTTTACACTGCCGGT
TCTGCTGGATGAAATGGAAAAGTCAATGTATATATTCGCGTCCGCGTTGGAGCGTTTAGT
GAATCATACAACAGAAGAAAATCGTAATCAACTGTCAACGGGGCTCACGTGCAATGCTTC
AGAGTACAGCTACTGGCGCAAAGGGGAGGAGTTCTACAAATACCTCAAGGAAACTGAATG
GACAAGTAAAAAGTTTAATTCAGTGGCTTTTAACTTTGATGGAACACGGAAAAGGGTTGA
TCTCGACATTCTCAACCTTGATGCGAGAAATGTTTGGGAAAAAAATCGGTGATTGGGGAGA
AAGTGGTGTAGACATAAAGGATATTATTTGGCCTGGGGAGGAGCGTAAACCCCCAAAAGG
TGTACCGGAAAAGTTTAACCTCAAAGTGACCTTTATGGAAGAGAGGCCCTTCGTTATAGT
TGGCTTGCCCGATCCCGAGACGGGCGAGTGCGAGTCCAGTCGAGCCGTAAAATGCCGTAT
TGCGCCTGAATCGGCCCTTATTGGGCTCAATGATACGATGGCACGCAGACACCCCAATTA
CTATAGGTGTTGCATGGGATTTTGTATCGATTTGCTGGAAAAGTTCGCTCAGGACCTTGG
CTTTACATACGACCTGTCCAAGGTCGAGGACGGCATGTGGGGAGTCAAAGATAAGAACGG
AAAATGGAACGGGCTGATCGCAGCTCTGCTAAATCGGCATACAGATATTGTGGTCACTTC
TATCAAAATCAACTCGGATCGTCAAGAAGCTGTCGATTTTACTGTACCCTTTCTCGAGAC
TGGAATCGCCATTGTGGTCGCCAAACGAACCGGAATTATCTCGCCGAAAGCATTTTTAGA
ACCGTTTGACACAGTATCCTGGCTACTCATTCTTTTGGTGGGCATCCAGGTAGCGGCCTT
TTCCATCTTCATTTTCGAATTCTTATCGCCCGATGGCTACGACATGAAGATCGCGCCTCC
CAGAAATTATAAATTTTCGCTATTCCGTACCTACTGGTTGGTGTGGGCGGTACTATTCGG
AGCGGCTGTGAACGTGGATTGCCCTCGAGGTTACACAGCTCGCTTCATGAGTAACGTCTG
GGCGATGTTTGCCGTAGTCTTTCTTGCCATCTATACAGCCAACCTCGCTGCGTTTATGAT
CACTCGCGAAGAGTACTATGACCTCAGTGGAATTGAGGACTCTCGTTTAAGGTATCCTCA
TCTGATGGAACCACCATTCCGCTTTGGAACAATTCCCTATGGAAACACAGAGCAAGTTCT
CCAGCGAAATAAGCCAACAATGTATGAATACATGAGACCCTACAATAGATCCAACGTTAA
TGAAGGAATTAAGGCCGTCAAAAAAGGGACATTGGATGCGTTTATTTACGACGCGACGGT
TCTTGACTATTTCGTTGGTCAAGATGACGAATGTCGATTGCTCACGGTAGGCTCGTGGTA
TGCCATGACAGGCTATGGCTTCGCTTTACCTAAAAAATCCAAATATTTACAGATGTTTAA

TCGCCAAATGATTGAATATCGAGAACACGGTGACCTCGAGAGATTGCAACGATTTTGGTT
ACAAGGTGCCTGTAAACCGGACAAACGAAAAAGAAATGCAGGCAATCCGCTCGATATCAA
CCAATTTATGAGCGCCTTTTTGCTACTCGGCTGCGGGGTCCTCCTCACTGTGGTGCTGCT
CATCCTCGAACATGTCTACTTCAGATACTGCCGAAAACAGTTAGCCAAGGCCAATTTTGG
CAGTTGTTTCTCCCTCATCAGTCTGAGTATGGGCAAGTCTCTCAGTTTCCGGGATGCTGT
GTACAGCGCCCAGGGAATGCTGCTCAGACGCGCTACGGAGAACCGCTTTTTGCAAGCCGC
AGAGCACGAACGATTGCAAGCTGACGTCGCGCGTTTGGCACGCGAACTAGATGTATTACG
TCAAAAAATCAAGACGTTAGAAAGTGATCTAACGACGACAAAAGCAGGTAGCAATGGAGG
GAGTCTGGATGAGCTGCGCGGAACTGGGATCGACACCACGGGATCGATACCCGGCTCACA
ACGGGTCGCAAGACCCCAAGCACCAAAACGGACACCATTCGGTGGACCGCTGGCTATTTC
GGGCAGTCATCCAAACGTGGCGGACAAAATTGTCGGACTACGTCAAGCGTCGATCCATAG
CAACACTTCAGTAGGAGCCGAGTCACCTACCAGAAGTTTGGCACGATTTACCGGCAGCCA
CGAAGATCTTCTCCGCCATCGGGATCTCACCCGAACCAAGATTCATAGTAATGAGCCTCT
GGTAATTAGTCAAGCAAGCATCCTACCGGCTGTTCCTCCACATAGTGTCCTTTTTCAGCA
GCAACAGGCTATGGTTAAGGCGCACATCATGGAAAAGGAGACCGTATTATGATGACCGCC
TCTCCTTCGACAGGTCGCTGAATGTCGGTGACCGCCAAACATGTGGCAAACATTTTGCAC
TATTCTGTTTGACACTATTGCAATTATGTTGAGGCTGGCAATTTTTGTTTAGTTCCGTAG
GGAAAGTAAGCAAAAGGCAGTAGCGACCTGTACCGTTGATGGTACCGCCGCCTGGTCTCG
TCACAGTTATTCACAGAATCGGAAGAAATAGATTCGAAATAGAGATTTATCTCGCGTCCA
GTTATTGCCAGGGCGTGCTTGCTTTTGCTCAAGCGACATAATAATGATATTGTTATCGCT
CTTCACAGTCATTACACCGTGGATGAAGCTATCATCTCACTGACGACCTTGTGCAGGTGG
CTCCGTATGCTCTAAACTGTATCATTCTGCGTAGTAAAACGCCATAGAGCCAATATGCTG
TGGGGATTTCCGGGTTGGTGCAGACGCCATGACGAAGTGGCCGACATAATACAACTAAAA
CGTGACAATAACAATTTTCGCTTGTTACTGTGAGGTTACTACTGGTACTAGTGCAGCAAC
GTGGCGCCACCTGTGGCAGGTGGCGCCACGTTGCACTCAACCACCGCTACTGGTACCGTT
AGCGACCGGTGATAATAAGAGTAACAATAATGTTAATTAGCAGCACTGCAAATAATATCT
ATTGTGTAATAATATAATACGTTTACTAAGGAGAGGCGAGTAATAATAATAATAATATAA
AATAAGGTAATCGAGACGCTTGCGATTAGCAGGGCAACATATACGAGGCACCGATGCAAC
TCTCTTCATCCTGAATTGATTTTTGACCCGATTAATTAATGAAGGATCATAGGGATAATA
ATAACAATGATATTGATTGATGATGATTACAATAATTGAAGTAACATAAACATAACCATG
TTAAATAAATAATATAAAAAATATTAATCGCACCGTTAGTACACTCCTTTAGTTCAACAA
CAATTCCAAGGGCATCAATAGTCCGCAGGGTCACGGTAACGATGTAAAGCAAACACAGTT
CGTTTCCTGTACAGCCTTCTCATTCGCCCATAGCCAATAAACTTCCAGCTGATTTTTTTC
CGATTCAAATCAAGCAACAGCTCGTACCCATGTCGTTACCATTAACCGTCTTTATTATTG
AACACTAGGTTCCCTTAGGAAACAGCAGTAAATTAATTAAAAACTCACAAGGAAAACACC
GTTCCCTCAGATAACGCAAATACGAATTTCCGTTTTTGGGTCATTCATTTTTACTCATAA
TTATCAAAAAGCCTTACATTTATAGATAAAGGTGCAAAAAAATATGGGAGCGCTTCGTCAT
CGTTGATGAAACCCTCTAACGAATATTTCCAAAGCGCTTGCATCCCACAGAATTCAACAC
AACTAGACTACCGGCTACGAGGGTCATAGGAACCTAGCAGGACCAAGGAAGGTTGGGTAA
ATTTACTGTATATTGACATCTATATTCAGGACTATTAGTGTAATCTGAACATATAGACAA
GATATCTACACCTCATGTACTACCCTCTTGAATAACACCATAGCAATAGAGCGGCAATAC
AGATAACACGAACCTAAGAACATACTATGTAGGCAAAACAACTGTCAAATCAAAGAAACT
CTTGAAAACAGCAATGAAAGAAGGTACAGGCGGCATTTTAGATCAAGGTGTCATCTATAG
AGAAGAATAGCCGGCGAGCGATGTGTCGCTAACAATTGAACAAATCAGCTAAGGTGCAAA
CCGAGCCTGTGGGAGCCTGCCTGTATTATAGGTCTGGGCTTGTAAATGTGCAATATAACG
TTTCATAGATAAGTATGCAAAATTAGAAAAAGAAAACGCGTACAATCAAATAATAAACAT
CAAGTATAGCACATCATTTATTTTATAAAACAATGCAAGCGCAAGGAAAATGTTTGAAAA
TATTAATCTTGTAAACGAAAACAGAATGCCCCCTTCCTTCATTAAATCGTTCTCAGCATT
GCTGTCCTATATACTTACGACGTCGATGTTACTCCATATATTACATGCACACACGCACAC
ATATATATACAAATACTGATCAGTAGCTTAGCGTATACTTGTCTATGTGCGGTCGCCTGC
GTAAAATGAAAATAATTGATAGTCGGATTACTTTAAAAAAATGTGATCACAAAGATGAGC
ACCATTAAACTGTATGTAGTTTCAACGTCACCGGGAGGCTTATAAACTTGATCTGAGAAT
AAAAGGGCTTTTTTTTCTTACTATTAATTTACTGTCTGATGTGGACGACATGGAGAGCAG
TAGGCATACGTGAGCGGGACAGGTGTAAACACAACGAAAATGGTGGAAAAGGAAACAAAT
TATAGGGAGAGGCTATTTTAATTCATATTCAGATAGAAGAAGAAATGATATGCGCTGATA
AGAATTCAGGATAAGAAAAAAAAAGGATTATTGAGTCTGATAGAAAAAGGGGTCTTGTGTA
TTGTTTAGCTATAGGGATACTGACCGTTGGCATTATATAATCACTGTTTTAGTGCAAAAA
AGTCATAGTTTGACGCAAATACCACGAACGAAGGTTTCAAATAAATAAGACAAAATAGAC
GTAAAATGGAATAGCTATGGGAGTTCAATATTATTCTGGTTTAATGATTAGTAGAAAGTA
GTACACACACAAAGAATCCAAGAATTTTAAGCGTGATTTAAGCAATAGGCTCGGCATGCA
GCAATTGAGATACCGTTATGTTACCCCGGTAGGTAAAATCTAATACATTATCACTCGTTA
GATTACAGAAGTGTATTAACCCTGTCTCTGAATATTCTGATTTTTCCGAATATTTACTTA
ACTCTCTTAAGTTACTGGCCTTGATTAGAACTGTGTAAAGCAGTTGACATAGTCGCAGAC
ATGTAGCAATAGTCTTGCTTGATAATAACGCCCGGTAGCTCCCTATTTCCACAACCCATC
ATCAATTTTGATGCTCTGTCCCGAGGGGATAAAATTGACCCGACCCATTTAGATATCTAC
AGACGCCGAAATAGCACGTGTGACTAACAGTGGCCAAAATCAACGACCTGTGCTATACTG
TCACCAACGCTAGCACGATCCTGCTAATAATTTAACATTTGTAGACTATTACTTCCTACC
CTCTTTCAATAACAAACGCAAAAACCGTAAATATCGTACACGACGAGACTGTGGGTTTTC
TCGAACAAGCAACCACCAAAAGATAAGCCAGTACTAACACGCCGCGGCATTAGAATATAG
CCAGAAAAGACGCGATGAAACTGGAGAGATATCGTCCAATTCAATGTTTGATCACTAATC
GCAGCCCAATAAGAAACCCCATTATGGCGCCAAGCCCATAATGAGCATGCATCCACCATA
GCAATGACCACTAACTGAACAAAAACCAAGTAAAGATAGCAAAAATCAACACAAGCCAGA
TGGTATCCTCAACTTCCACGTTGCACGCTGGCAAAAAATGCGTATAACGCAAGTAATACA
TGTTACATCGGCTACAAATAATCTATTGATCGTCCACCTTAACAAACAACCTAATAAGAG

AACTTATATAGGTAGACAAACAAGCCGGGAAAGAAGTTTTAGGTGACATTCTAGCGTTAT
ACGGGATTGTTTCAGCAACGTTACAAAAGGATGATGGGCAACGAGAATTCTTAAACCAAC
AAAAGGTTCAGTGCCATTGTAAATGATTACAATAAAAAAGATCGTACTTTGCAGGATGTA
GGATTATGATCATATACGATGAGGTTTCTAAAAGTCAATTCGACTAGGCTAGAATATGAG
TCCCCGTATGTCCGTTTCTGATGAGGCCAACATTGTTGATTAAGGCGCGGATAACTGAAA
AAAAGGTATAAGGGAATCATAGAAAAAAATTCGTCTGATTTAGAGAAGAGCGCAGATGCC
AGAATACATAGAATAATCTTTCATACGTACGATATGAGAGAATCTTGCGTAAATCCAGAG
TTACTTAGCACCGCAACCAATTGATTCACTGTGTAATCTTTTCTAGTAACTTATTGGCCG
GACGGTTTCAGTAAAATGAGGACTATGACAATTCTATGTAGGAAAGCCCTTATCCACTAG
GTTTTCGATAGCTGCATTGAAAGCTATTTTGAAATGTAATGGTTGTTATCGATAAGGCGT
TGATGGTTTTTACCCCGTAAACCATCAACAGTTATCGTATATACATGTATGCAATTCCAA
TGGCGGTAACTGTAACAATAATTATATTCATTAAATCTATAGAAATAGTCTAGCAGAATC
AGTAGTATCACACTATCCACACCCCCTGATGTAGTTGGTGCGTAGAATAGCACATATCTA
TACGTTGCTATCGTTTCTTTACGTCAACGAAATATATTGAAGTACACCGTCCGGCCGGCC
GGCCGGCCGGCCTTCGAGTCATGTATAAATGACAAAGCCTAATCTAGATAATGCAGATAA
GAGAACAAGCCCTTATTGAATAAATGTCATCATACGTATATATAATGGGTATAAAAGTCC
TCTAATGACTGTAAACAGAAGTCGAAAACCTATAATATGAACATGCGATAACGTGAATTA
GGTTTAATGCAAGTTACGTGAAAATTGCAGCCTGTAAGTATATATAAAGAAAAAATATAT
ATATACATATGACGAAAGACCGGTAATAAACCCAACGTTGTTACTAAACGATAGGGTCGT
TTAGATTGTGTGCTAATAGTTCATTATTATTGTGGGCTTACACGGTTTTTATGTTTTTAC
TTCGAATGCGGACGATTTATTGAATACATATGTTGCACTGGTTCAATAAAGCCCAACCAT
ATAAATTTATAAAGCGGCTTCATTTTATATCCGCTACGCGTGTTTATTCCAGAACAGTGC
GAAGCGTACAATACTACCCTGTCTGATTTATATTTGTTGCATACCCATTTGTCACATATA
GAAATACCTGATATTAGGATTAAGATATCAAGGTTAGATGAAATAAATTTCAAAACCTTA
ATGGGCACTATTTA

>Vd21758 len=3164 path=[23942:0-20 23963:21-157 58044:158-159 24101:160-3163]
ATCCTATACGTTCTTACATAAAGGATAAGGATATTTTGTGACTTAATCCGAGTAATTCTC
CTATTAAGCACGCGATAAAGGAAGTTTTTGTCATCTCAAAAAAATAGAACAGCCGTTTTT
AAGCTATGACTATTGTCCTGCAAAGGTAACAGCACAACCTCAAAGAATTTCCTGCTGGAA
CCAGACGCAAGGCACCCATACTCAATTGTAGATTGTCGTGCAAGATCGTCGGCGGAATCA
ATTGAAGAAACGAGCCTCTCGGCGGTTAGTGTCGCCGCCAAACTGGCGGTGTATGAGGAG
ACGACAATCAGCGTAAAGAACCACCAAATCGAAGCTATTGTACGACAGGAAAGCGACACG
GGGTTAAGATCACATCCTTGTTGCATAATTGATCCTATTGTGAACCAGAACCTGCTCATA
ATCTTCATCTCTTGATCAAGCAGTTGTGGGTCGCGTTGAGACAGGTCGTCTTCTCCCAGA
TCACGATTCTCCGTCGGACTGATAGACGCTATCAGATAGTAAAGGACTGTGACACCCGTG
AAGGCAGTAAGCATGCAGATCCAAACGTCAATCGAAAATGGCTTCATAAATAGAAACAAG
GTAAGTCCTCCTTGTGCGGGCTTCCGAAATAATATTGAAATTCCAGTTTTCAGAAACGGC
TGCGTGAAGTCGACGGCTTGCATTCTTTCGGCAGTGATTGACAGGTCGACTATTGCCATG
TCAGCTTCCATATTAATAAGTTCCTTGATCATGCCATCGAAGGTTCCGTCGGGTTGTCTT
GATCCATAACTATTATCCTTAACGAGATGCACACTGTAGCGGAATCCGATATTTGCGGCC
AGAGTTTCCATGAGTTCCATACAAAAACCCTCAAAGCGGTCGTTTCCAGTGCGATTTCTA
TAGTCAGACTTCAGCATTGTATATGGAGGGTTCACGATCGTGGTCACGTTGAACACCTTA
GATGCCATGAGATTTTCCAGTTCTTTGCGAAATGTCGCTGGATAAGAGGCCTGCGTTCGA
ATTCCGCTATCTTGGTTCCACGTCGCTATCTTAGCAACACCATTCCACATGATTCGCATA
ACATTGACCTGAAACCGCGAGCGAAGACCATGACGATTCAGGGTGATTATGCCAGTGACA
CCACGGAATGAACTTCGAAGCATCTTCTTAATGACGAGGTCGCCGGTTTGCCAGGGCCGT
GGAGACTGGCATTTGAAGGGCGGTGGCGACCTAAGCTTCGATGATGCTGATAGTTCATAC
AGCGTCCGAACCACAAACGATACGGAGTCTATTAACAACGCCTGTTCAGTCGTCAAATTG
AGATTGTCCCTGATTCCATCCATGAGTTGAAATCTTCGAAAATCCCTTATCATTTTGAGA
TTCTCTTTTGATTTGAGATCGAGTAATTGGAGGAACGTAATATTGGCCCTTGATGGAAAA
AATGGACTGAGATCTAGAGTATGCAGGTCTAAGGAGGTGATGACGAAATTATGGTACTCG
GTCATAAGACCGACGTCTTTAGCCGATCTCAGCAGTTCGGGAATCAGTTGAGTATCAACA
TCAAGCACAATGTGCGTGTCTTTGGAGCGTAGCACCTCCTTAAAGAGAGTTCCCGGTGAG
ACACTTTTGTCCAAGGGACGTAGCGTCGTCAGCGAAGGCGCCGCCCGAAGAATCGGTGCG
AGACGAAGGAACGCATCCTCTCGGTCGTACACAATGGTTAGAGATCTCCACTCCGTCATA
TTCAATACGTCGAAGTACGCCCTGCCTAATATGTCAGAAGGCGGATGGAGATTTAGCATT
TGCGGAAGCTTCCTAGGTGATGCGGCGCTTGTACGTGAGAGCAAAGGCCCTATGGAGAGA
AATGGCACATGAAACGCTCTGCAGGTTGATGCCAAAATATGGGATACCGGAACGACAGTG
GGGCCCACGACGATCGAAGGAGCACGGGACAGCAACTGACAAACAGCTTTCGATGCGTCA
AAGCTGCTATGAATGTCTGGGATGACTTTTTTCATGCCGATGAAAGCATCTCGAACAATG
GGAAGTCGCGAGCTCTCCGGCGCACGACGTATGGCGAAATCGAATACGCGTACCAAGGGG
CTATTACTGGCAAAGATCCCACCAATATTAACAGAGAAAGGTTGTCCAGCCTCAACGCAC
CCGGCAAATAAAGTCACTACGAGGAACAGAATTATTGACCTTATGCTGAGCATACTGATA
TCATCCGTTATCAACATCGATTACCGCCAAATATGTGCCAGCTGTACACATTGGCCTCCA
AAATGATAGTTAACTGTAGAACAAGGAGACGTCCTATAAACACCTATAAACACTGGATCC
TTTCTGCCATTTTTCACTGTTACGAAGAGATCAATCACTGGTAACGATTATAGAGGTTCC
ATATTGCGCGGGGCAAACCAGCATCATAAAAATACAACAACAACACTGGACTTAATACGA
AACTTTAATACGACTTTAATACGAAAGTTTTTATTCGTATTGATCGGACACAGTTTATCT
TATGTAAGCTATTAACTAGTGGAACAACCTTTTCTTGCAATGAAGCACTGACGAAATTCG
TTATGTGATGACGACTGTGGATTGCAGGACTGCTTTGATACGCTTGTATCAATAGACTTA
TTAAGCTACTTTTTTAGAACCAGCATGATGTTTCCGCTGAAACTTATCTCCGAAAATCTT
CCGTTTTAAGACAATTGTCTGCAAAAAGGTCTAAAAACACTTCAATATAGCCCAGTTGAA
CTTGAGAGCTAGTGGAAAATTTCTAAATTAATGTTATTCACGGGCATCAGTTCCGGACGA

ATATAAGGTAAAGTATTGACACCCTGTAAGCCGGAGATTTTTCGTTGGCTCGGTGCGCAC
AAGGACAAAGTTCTTGATGTTGACAGTTGATTATTACCCGCGCTTTCTCCGTTTGCGATG
CGCGTTTGGTCCGCTTATCGCAGTCGTGGATATGGTTCACTGGCTGAGTTAGGGCTATAA
CGCTGTAGTATTAGACAGCGACGGCTTTTGACGAGCAGAACCACCAGACTGTCTGTGAAC
CATATAGCGGGCTTGGCAAAGCTGGCCAGTTATTATGAACATCATATCTATCTACAAGTC
TGAGGGGATTGAATGCAGCAGATCTGACGGAAAGCAGAGAGGGA

>Vd21835 len=8381 path=[16061:0-3723 19782:3724-3725 8116:3726-4439 @3961@!:4440-4696 4218:4697-4716 @4238@!:4717-6474 5996:6475-6475 5997:6476-6625 6147:6626-6834 6356:6835-6868 6390:6869-7202 6724:7203-7289 6811:7290-8380]
TATATATATATAGATATATATATATGCAAGTAAGTACAAAATATGGCAGTGTGGTAAGTTTG
GCGTCGCGTCCGACGAGGCTCGGTATTCGCTCATAGTCACCGAAAAAATGTCATATCTAT
TCTTTTATATAAAACGTATCTTCTGAACGCTAATTGATTTCCATAAGAAAATTCTTCAGA
TTTAACGAACGAATTTGCTTTGAGATCGAATGGATACAAAATGTAGGTGCCCTGCTTATT
GAAATGCTTCCTAAATCTAACTCGGTTAGGTGTTTATATGGGGTGAATAACGGAAGCGTC
CTCATCTTGCACTCACAGATATAAACACGTTTTCACTGGTAGAAATGTTAGTCGTACAAA
CAGGCTCATTAGAGTTAGGTACATCTTAGTAGTCATAGATCCAAGAGAATGACAGCTGAC
TCGAATGAGACATTGATCGACATCTTCTAAGACTATTCTATTAGTTTGGCACAAATCTAT
CAGTGTAAAAGCTAGTTACCTTGCGATGTATTTATAATACATTTAGTGCAACGCACTTAT
AGAACACAAAGGAAATAGCTAGCTAAATATAACAATAAGGTTTTGTGAGTCGTCGGACGA
GAGCCTGGTAATACATCAGCTCCGCCTAATTTGGTCAACCAACTGCCAGGAACGTTTCGT
CCAATAGATCGCCGTTGGATTAAAGAATACCACCAAGCCAGACACGAGATCCATGCATGC
TTACGTATTCACTTTCATGTTCCGTTTGCTGACGCCGTACGAACATCAAAACAGATGCTT
GTCATTGAGGAAACCGACTTAACACAGGCCCTGATCAGTAATTTAGGCAAGAAAATTTCG
AAATGCACGATTGTATCTGGCTAGATGGCTGTTAGCCAACAGGAACCCGCTAATGTCATA
CCCTCCGCCACAACGCACCGCGTTGCTCTCATCATAGTCTAATGACCCAATAGACGACAG
GGTCACTTCAAGAATCAGAGCAGTTGCCGTTTTTGGAGTTCCTATGTCGTTTTCTAAGTA
AGTCCCGTCGTTCATGGCGGGAAATCTCATATTGTGCGCGTGGGGGACGGCGGGACGGGG
GGTACAATAAAAAGCTTTAGAACTGCGCGACAGGCCGTCCAATAAAAGGAGTTTTCGCTA
CATCACGGATATTTTTGTTGAAAGCGACCAATTTTCTTTCACTTGTATATTCAAAGAGGT
CATATACAACAAAGTATCATCATGTTTCATGCTTTTCTCAGGTGCAATCTTTGTGGCCAA
TAATACAAAACGTGAAGTAACACATACATGAGGTTATTTAAACCTATGTTGTTTATCTGC
TCCTTACGCGGCGTTCGTATTATTCAGCGAACAAGCTCACTCTGATCTCTAAAGCACCAC
TGGAACAGCAACCTCAGCCCTTCAAAACTGAAAGAAAAGGTCGTATCTCTCCTCTGCATG
AAATAACCACAAAAAATGAATTGAGCTCATTTTTGTTTCGAGAAGATATTTCCACTACAAC
TGATTTTGACAGAGTTTGTTGTCAATTAGGTTGTATCTAGTCGGATACATTACAGGACAT
ACAAATGGCTCCTGATACCCTACACAGTGCTTCTGGACAAAGGGAACAATAGAACCCTAG
AGAAATTATGCGACCGTTTTCATTGGTTTACCAAATTCATTGCGAGTACGTAGCATTTCT
AATTGTATGCTATCAGGCTTCTATTGTGTCTTGAAGTTGATCGTGCGATATCTCGTAATT
TCTTTTGCCACTGTGGTCATTGTATTAGGTACGATATCATGAAATAGTTTTCCTTGTTCG
GCTTGTACTGCATGAAAATTTGAGCAAAATTATCTATTTTTTAACACCTGAATTCTCTGG
TCTAATATATATATATATATCACTTGGGCGGATATCTAGAAACATTGTGTACGCATATATGA
AGGTTGTATATATATATTATTCTGCAAATGATGCGATTTTCGAACCAGTTTTACCAGACTGC
TTCGTACGCGGCATTCGTGTCTCGATTCAGCCATTTTTTTTTATTCCTTCAATGATTATT
GCAGGCAGGATACCGCTGTGGGAAAGCAGGGAAGTATTCCATCGACAGGCTGTTAGAAGG
ACTGCTATGTTTTCGTGTCGGGGCATCATAAAAACGTATGCCCATTGTCCTTTCAAAAAA
AAAAGTGCATAACGTCACATAATAACTTCCGTAGCCAGTCAGATCCTGTAGCCGTGTGCC
TTTCTGTCACACTACCTAATCAATTAAAATAATCTATTTGGTCTCGTTACCGTTCATTTA
AAGTTCACGAATAGATACGCTTGAGGCATGCTTATCGTCACTGCGTACGTTAATGCTTAA
GATCATAAGTATCCTTTGGATAATATAACCAGAGGGGGGACATATGCTCAGCATAGACGC
ATACCGTACAGTCAAGGGGACAGCTTAACCTACCGACAATTGGAGTATTGTTTTTACTAG
GTTAAATTAGGCCCGATCTTTTTCTCCTCCTTTCGTAGAAAAATAAGTGCAGATTAAAAC
TTCGGGGAGACTGGAGGGAGATGTTAGGAGTCGTTTTTAACAAAACTATGCATTTATTAT
AATATCGATTCCGTCTGCGTGTATGTATAAGTGAACGATTGTTAAATATATAGATGAATG
TTTGATATATATGTATATATCAATAGAAAAATCCCTCGTTTGAATTAGCTCTTTTGCCCG
CAGTTGCTACTAATACCGGGGTCTATTCAACTTCCTGTTTAGAATCTATCCCTTTTGCTG
CTCTTACTTGTTTCAAGAAGACGCAACAATAATTAATTATATGCATGGTAGTTGCTACAC
ATGTCATCGTTGAGACACTTTACGCATACCTATCTTCATAAAGCCCGTCACTATTTTATC
ATCCTCGGGACTTTATACTTAAACTCTACTGCTAGCTAAGCTTATGTTGTTCGTATACTT
ACTTGTGATGCTTGTTAGGAAACGATCTTCGTAAGATTTTTGTCTTATAATAACTTGAAT
AATAAGATAAATCTGACGCTTCATATTGATCACCATCAGCATGGGCCATTGCCATCAGCT
GTTTTCAGGCAAGGGCCCACGAGCATACTCGCCTATTACGACCTTCGACTTTTAAATAAG
CCGAAATACAAATATCGTGTAATCCGTATGGTAGCATTAAATGTCTACTGAGTAGGCGTT
AACAGACATCGACACTTCACGATCGTACCAGCTAACCAATTCGTAAATTAACTTCGTATA
TAAATATTAAACGTATCATACCGTACACTGAATGAACTTAGGTATACAATGACGTCAGCG
ACGAATTGCAGTTTATCACGCCAACCGATGTTACGGGGCAACTCGTTATTGAATTCCCTA
TTGATCTGTCTTCTAAGAACCTATATATGGGAATTACCGACCATCAGCCGATCCGAGTGC
ACTAGCAGCCCGTTACAAATGTGTACTCGATATTGAAGGTTCACGTCGCAAAGATCAGAA
GATTTGAAGACTACTGATCTGTTACGGGGGACAAGATAAATGCCCCCTCGCAAACAAAAG
AACAAATTTGTATACGTAGATCCTAGTCAGCATGAAGTCTATAGAATACCGAACACTTGG
TGATTTTACACTTCGGAGCTTTCATAGCCGATCGAATCTTGGTTCTGGATTGTGGACCGA
ATGTTTGCAGGAGGCGGCGGGAGTCCGGCTAAGCTGCCAAGATTGCCCAGAATGGGGTCG
ATCCTTTCAAGGTCGTCATGCGGCGTACCAGGTGTGTCAGCCGGGGAGCGATCGATGGAC
TTCAGCTCGGGTTGACCCTCAAGTTCCATTCGGGATAGCGGGCCCTGGTTGACAACAGCC
GGTGGGGGCGGATCGCCACCGAGTTTACTTAATCCACTTAACGGTGGCAAAGCGTTAGGG

CTGGCTGGTGATGCCGGCGACGCAGGTGAAAGGGGACTCATTGGCCCTACAGGGCTCATG
CTGGGCAATGTTGACTTAGGAATAGGCGGTCCTGCTGGTGCGGGCGAAGGTGTGCGAGGT
GCGGAAGTTGGCGTCGCTTGAGCTGGGGCTGTGTTCTTTATTGGAGCCATCGTAGGTGGC
AGTTGTACAGGTGGCACCCGCTGGCTCAGCGGAGGCAGGCTAGCCACGGGTTGTGACATA
GACCTGCCCATCTGGGCCACTTGCGGGGCCTTCGACATCTGCGGAATAGTCGATTTCGTC
GTAAGCGGTTGAGTCTGGAAGGTCGGTTGAATTTGGGTGGTGATCGGCATTTTCGGTTGG
GACTGTGTCAAAATTGGTTGGATCGGCTGACACGGCTGCTGTGGGCTCTTCAAAATCAAA
GTAGGTGGACTCTGACTCGATGGTTTGATTCCTATCAATCCTGCATTAACTGCTTCTGTA
GTGTTTCCTGAATGCATTTTGATCGTCGATTGGCCAGACACCGGCGACTGGATAGCATCT
CTGGAATCCGTGTCGGTGAAAGGCTCAACATCAGCGATCGGCGGGAGTTCGCTCCTCCGA
AAGCCACCGTCATCTCCGTCGAGGTACTCCTCGTCAATCTTCATCTCTCGCGGTAAACAA
CCTCCCTTCTTCAACGTAGCGTCATCTGCAGCATTTGCAATACCCACACCCATGCGGGTA
GCATCGTCCATCTGCGAATTGCCTATTGGAGCTTCATGTGCGGTCAGCTGCTGGGTTTCG
ACCTCTTCCGCCTTGTCTTTTTTGCCCTTGCTTCTACTTTTTTTGGCTTCGCGAGCGGTC
TGTCGCTCGACGATCTCTTCAGGCGTTCGTTGTTTTTTAGGCTTCTTACAATAAACCTCG
CAGATGGTGATGAAGAGTGCTATGATTAAGCCTAAGCCTAACACTATAAACCAGCCTAGT
ACACGCCGAACTTCCAGTGGTTTCATTATGTCCAGTGGTTTCTCACCACCGTAGGCTAGG
CTGGGAGCGATAACAAGATTGTCAGCGGGACAATCGAGCGCGTGGGCTCCAAACCATCGA
TCTTCAAGCTCTGCTAGAAAGCCTTCATCTCTCAATGAAGAGATGATATCATCGATCTTC
TTGCTCAAAGCGAAATGTTTTGACATCGCTACAGCGTACTGACCCCGCCTCAAATCCGTT
CCTACCTGGATGGTATCGCATGGGGTGTGCGCTGACGTGTAACGAATGAAGTTACTATCC
GCAAATACGATGAACTTCGAGTTGTTGCGAACCCGATCTATGGCCTTGGGGTAGCTCTCA
ATGACGCTGTCGTTGAAGGAGAGTATCCGCCTGTACATCGCCTTGTACTCACTGTCATCT
GTATTCTGGAAAAAGTCTTCAGTAGCTGAGCCTCTAATGAGACCGTATTCGATGCGATCC
TGATAGAGCAATTCCTTTAGGCAACGAGGCAGCTTTTCTCCGGTCGGGGCAAATGGGTCG
TTGATCCATAACTGTGCCGTATAGATGGAAAGCACGCAGAGAATAAAAAACCACCACGAA
CACGCGATTACACGCGAACCTAACGATTTTGGACGGCAGTTGTTGCACTCTTCCGACATG
AACATGCCAAAAGCGTACCAAAGCGAATTGTAAAAATGACGACACGGCGTTGGCGGGTCG
ACAGGTGGTGTGGCGACGGCCAAGTTTATCAGATTGATCAATAGTAACACTATAGCCACT
GTTCCGTAGGCGCCAAGAAACAGCAGCCAGATCTTCCAATCGAGTGGCCGAAGAAACGTG
AACGGGGTCCGAATGTTGAGTAGGTCATCAGCGTCGTTACGGGCAACAGCACCAATAGCT
GTAGCCATGAACGGTTGGCTGAAGTGCACCACCTTACTTCGTTCCGAAGTAATCATTAGT
GGCGCAATGGCCATGTGGGCGTCTTTGCGTAAAATTTCACCAATGAATCCGTTCCAACCA
GACGCCTGCTGACTATCTTTGTAACCAAATTTTCCATCCTTAACGAAGTGAATATTAAAC
TGAGGCCGTCCGGGTATGTCTTTCATGAGGCGTACAATGAGGTCCTTGATATAGCCGTCA
ACGTTGTCGATGTGTGGATTGGTCTCATTGGTGTTTTTGAAGAACAAAAACGGCCTTTCA
ATTATGGTAGAGATGATGATACTTTTGGAGAGTTCCTGTTTAATCCGAGCTCTCTTCGAA
TTCCGTTCATGTTCATTCGAAAACGCCAGACCCTCCTGTTCATTCCATTTGCCCACCCTC
TCCATGTCAGCGTCTGAAGTCAGTTCTAAGACATCAAGGGTATATCCGGTGCGTCGGCCA
TGGTCGTTAAAACGTACTTGTCCTGTACTTCCTTTGAAATGTATCTTTCGCATTACATCA
GCGATAGTTTGTCCCTGGGGATATTCTTCCAGCGGAGATCGGTAGCAGGTCACCGCCGCT
GTTATCTGGGACAGTTCCTTAAATATATCCTCAGCCGCTAGTGGTTGCTTACCGCTCAAT
GCCACCATTGTGTCGGAGAGAAGTCGCCCGGCATCCATCAGGAGAGCTTCTTCTTCGTGA
ATGTGTTCATCTTCAGAGTTTTTCGGTGTTGATACACCAGCACGCATTCCCTTCATTGTC
TTGAACTCGGTTTCCTCGACAATTTGAAACGTTGCTACCGTCGACTGATCGAACGCACGA
ACTTCCGACCAGAAATGGCGGACCGTGAATCTCGGTACGACGAAATGGTAGTACGCCCGC
CTTTTGCTGTATGAATGTTGTATGATGCGTTTCGCCATCTTGACATCGATCGCCAAAATA
ATGTGTTTTTCTACGTCCCACGCTGCGTTCTCCTTCTCCTGCAAATACGTCATTGCGGAT
GTATTCGACGTCACCTGATGAATACCTTTTACCTGGACCTCATCCTCTTCGAGCAGGTGC
GATAGGATTAATTCCAGCTCCTCAAGACCCTCGGGCTTGTCGTAAATGAAGTAGATATCT
TTCCACTGGTTCTTCCGAATGAGATCTGCTGTTGCCTTCGCCGTAGACGGAGCCATAACG
ATCGTGATGCTCTTCTCCGATTCACCGAGTTGTTTTGTGTTGAATGTCGTATCGACGAGA
TCGGGCGCTGTATTGATATAAGGTATGGCTAATCGGCTTGCAAGGCTTTGCAAGGTTGCG
TGCACTATACCAGTGCAAGGCGACACAATACTCGACACTCCTTGCCCAACTTGGGCGCAG
GCAGTATTGCTAATGCCGAAGGTTGCATTTGGCTCAACATACGCCAGCTCTGGATGCATT
CTGTTTTTTGTTGGTAGACCGGACGATCGCTCCTTGAGTATTTCTTCCAGGAGCTCCTTT
GTCCGAGGTTGGCCCGCTGCGATGATCCCGCCGATCTTGACGGCAGCGAGGGCTGGTCCC
AGCAGAACTAGGAGAACGATGAAGCCGAATCCTGGTCTGTGCATCTCTACGTTTTTGGCC
TGCAGTCTCATCTCTTAGCAGCACAGCCACAGCTGCTGCAGTAGAACCCACTCTCCTCCG
TTGTACCTCCGCTACCACGTCAAACACTTAGTAGAGTTGTATCAGCAGATGCTGCTAATC
AGCGGGGGTCGCTGTAACAGTGACGGCGAAGATATTAGCGTCGAAGCATTCAACGCACTA
CGACGATCCGCACTTCCAAAGTTTTCTTCGTCGCACGCGTGTTTTCCCTTTGTGCGTTTC
TACAGCGGATACAGACTTGTCCCCAGATGCTTGTCGTCGGTTCAACTTGGTTTCAACACA
ATCCGGCCGGCCAGTCTTCTTTCCGGCTCCCAGTCCACGAGCAACTCCTCAGTATGGGAG
GTTTTGCCTAAATCAAGCACGCATCTCTAAGCAACAGTGAATCCAGCACCGTTAGCTATT
CAGCCTCGCTAATTAAACGGTCTCGTTCGCTTGCTCGTCCTAACACAGCCACTGATTCTC
TCTGAATCGCCGCGTTACGTGCAGCAGCGCGCATCCGAAGCGACCGATCAAAGCGGCTGA
CCTATGATCACTAAGCTATAATCTCTAGACCGGCCGACTTAACCGGCGGCGGCGGTTATC
CGATGATGATCTGTGGGCTGGGATGACATTGGCAAAATCCCACGCGGCACCGATTCGACA
CGGACTCACTGTTCACTGCGCTACTGTTTCCTGCTGCTACGACTCACGGAAAACGGCAAT
CTGACCAATCCCTATTCCTTACCACTTCCTTTCCTGTCCCTGCTTCTGCTACGGCTGCTG
CGCTTCCTGTCGCGGGTGTGGCAGAAGCCAAGATGGCCGGCACCCAAACACCGGGGCGAG
AGGCAAGCTGGCTGGCAGGAGCTACTAGCTCGGCCTGCAACACACTGCCAAATAGACCCC
GGAACCAACGGACACAGTGGACCGGGAACGCGCGCGCGGCAGCGACTCAGCGATAACGGC

GAACACCACAGCGCGCACCGGCAGCGGCAGCGGCAGCGGCA

>Vd22269 len=3992 path=[4672:0-453 5126:454-493 @690@!:494-1222 44898:1223-1250 44931:1251-1251 44932:1252-1256 5919:1257-1270 210:1271-1505 44980:1506-1508 6170:1509-1806 6466:1807-1808 @6468@!:1809-2497 7152:2498-2509 7164:2510-2589 45213:2590-2590 7245:2591-2663 7318:2664-2672 7327:2673-3991]
GTTGTTGTTGTTGTTGTTGTTTTCATTGTCGTCAAATCATTGTAGCTTGTTGAAACGAGC
TGCTTGATAGAAACCGATTTGTTGTTATTAATCAATCACATAGAGAGTTATCAGAGTTGT
TCATCCATTGTAAACGTTGTTGCTTCGTGAATAGTATAACGTCGACGGGGTGGCGCACAT
CGCAAGGCCCATCACCTGAGGAGACCAAACCATCGTCTAGGCCAAACAGTTCACCTGGCC
CCTAGCGGCGTACAGAACACGCCGAACACGTTTAATTTGTACGAACGTTTACATGTGATT
TTCCTTTCACGTCTTGTATGTGTTCTACACATTTTGTTTAAAAGCACTTGGCCATACGCT
TCCGTTCCCAGAGGTGATGCTTACTTCGTCGACAGGCTTACATTGCTAGCTCTGAAGAAA
ATCCCATCCAAACTCAGCTGAAAAAGTTCTGGCTTGAATCAAAACAGAATGAAGGCAGAG
GCGATCCGACGCAGAAAAAAGTCTGTCGATCTGATCGCGCAGTCTTATGGGCTGATCGAT
TGATTGAGTGGCTGACGTTGATCAGAATTGCGCTGGGACTGAACTGAGCTTATGCACGGT
CGTTAGAAGCTGCCAGAGATGGAAGCGCATGGGATGGTGCCATTAGAAATATTTAAGGCC
AAATTAGGCCGTTGAGTTGAACGGTCTGCCCGGCACATGTGGCCTGGTCAGGACCCAGCA
CATCACTGGGTCTCTCGCCTCCATCACAGAAAGCAAGTCTGCTCGACATGAAAACTTCCA
ACGCCAAGTTACACAACAGCATTTTTATACGTCAATTGGGTCCTCTTGAGCAATCCTGGC
CTCATATCGGTAATATGGCCCATAGGTGTCCGGGCTTAGGTGCGTGGCCAGGTCATCTGC
GCATAGCTGATCGGTGGATCACACAGCGGTACATCGCCGGGTTGTTGGCCGGCTGGTTG
ACCCGCCTGGCCCCCACCTGCTGCTGTAGCGCTCTTCAGGTTGCCGATAGACGTGGCCTG
ATGAAAGGCCTGGTTGAGGCTTAAGTTTGCTTTGCTTAAACTCAAACTGCTCGGGTTGGC
TTTTAGCTGGCCGAGTTGGCCGAGCTGCAGTTGACCTGCGTCGTCCGGATGTCAGGAAAA
CGTGTTCTTGTTGTGAAGGCCAAACCCCGTGATTGGACCCATAAAAGGACCCATGCCATT
GTCGGTCGCAGCGATGGCGAGGTCTTCATCCGAGCGAGGGCGTGTCAGGGGTTTTTTTGC
TTCCGCACGTTAGCGTGAACTTAAGTTCTCTGCAGAGTTCAACGCAAACGGGATCACGTT
CTTCGGGCACGATCTTCTTTGCTCGCCAAACAAATTCGACAATGGCCGAGATAATTGCGA
CGCATGATCCGACGAGGAGCACAATGAATACACCTCCTACCGAGGCCACGGTCATCGCCG
ACGTCCCTTTATTGATGATATCCTTCGGGCACTTTTTGCTTATGCGCTTCTTTTTCCACC
AACGCTCCTTAAGCACATGAAGGTCACCGTTCTCTTGTAGCCGAAGGATGGCTTGCGATA
GCATAGTCCTGTAGGGGGACCCCGGAGGAGTGGCGATACCGTAGCCCTTATTGTCGAGCA
GTGAGCCGATCTGGGTGAGGTCGCAGTTCCTCTCAATGTTATACTCGATTGAAGTGGACT
CCATCAGGTAAGCGTACTTTCCCCTTAACACACGATCGATGCCCTTCTGGTTGCTCTCAG
CAAATACAGTTGGCCGTTGAGATTGCATGACGTGCCACATTTTAGCGTATGTTGGGAATT
GCGAATCTTTGAAGAAGGATTGTGTGGAGCCAGACCTAAGACAGCCATACTGAATGGTTG
TCTGCTTCGCCAAATCTTCAACGCTTTCAATTGGAGATACGAGACGTTCCACAGTGAGGA
ACGCAGCCAAGTTGGCGGTGTAAGAAGAGACCATGATCAAAGTGAAGAACCACCACATGC
CGGCTGCTACACGAGTCGACAATGCTCGTGGTGTTACATCGCAGCCTTGTTGCATGAGGC
ATCCAATAGTGAACCAAAGCGTATTCCATATTGTAAATTGATTGTCCAGTTCCTCAGGGT
TTCCTTCACAAGGATGTGGATTTACCCATTCATATGGCGAGAATCTCGCAAGCACGAACA
GGAATAAAGATACTCCGAGAAATGCTGTCATCATGTAGATCCAGACCTCGAGAGAGAGTG
GGTCTAGGAACGAGAATAGTCCGGGCGGGGGTTTCGCGGTTTTCTTGTAGAGGATCGAGA
TCCCCAAAGTCATGAAGGGCATCGTGAAGTCCACGGCCTCTTCACGGACGAAAGTGATCG
TCAGATCGGCCACAGCCAAGTCCGCTTTCCCGCTTAATAGTTCGCCGATCATCCCGTTCC
ATCTTCCATCGGCGTCCTTCCGGCCATAACTGTTGTCTGCGACTTCGCGGATAACATACT
TCATGTTGTTATCGAAGTGCTCGGAGGAAGCAATCGCGTCGAGCAAATCTATGCAGTAGC
CTTCATATTTATCGTTCCCTGTCAGCCCGTTATCTCTCCAATTCTCTTTGTACATCACGT
AAGGTGGTTGCAGAATAGTGGTGACAACCAAAGTCTTATTGAGGAGTGAGAGTCGGGCCT
CCTCGAGCTCTCTGGTGTAGTTTGCAGTGATGCTGATGCCACCGTGCATCGTCCACTCGC
CGGCCTCTCGCCAGCCTGAGTGCGCCATGTCCAGCACAGTCAGCCGCATATCTGTGCGAT
GGCCGTCTTTGTTAAAGCGGATATTGCCTGTCAGCCCCTTGATGTTGATCATCCGCATAT
ATTTGACGAATGCGTTCCCCTGAGACCATTTGTGCTCCCGTTCGCATGATAGGGGCGCCA
CCTGAATTTTGGGCCCGCTGTCCGGGTGCAGGTCCTTCAAGGCAGTGGCGAACAGCCTCA
CAGCATCGTACATCAGCGCTACTTTGGTCGTTAGATTCTTGTAGAAATCTTGAGCACCGG
TGTTGAAGTTGCTCTGTACCGATGAAGACTTCTTGCTCGAGAACCTATGTCGCCGTTGGA
TCCAATCGCGTGCGACCTCGTCGATTTCCTTCGATGAGAAGTCGATCAAACGAAAAGCAG
AAATATTCGTCCCGCCATATTGGAAGTCCTCGGTATCTATGGTGTGCGCATCTAGCGAAG
TGATGAAATAGTTATGATATTCGGACATCATGTCCACTTGCTGAGCGTGTTTTAGCACCG
TGTAAAGACTCCTTATCGGTACATCGAGTACAATATTTTTGATTCCTGTCTTGCCAATAT
CCTTAAGGGTTTTTCGATATTCGGTTCCAATTTCGAACTGACGCACCGTCACTCGGTTCT
CCTTCATCAGTGTAGCGTCCTTCAGTATCTCTTGCAAACGTACTAGAGCGTCGTTCTCTT
CGTAGAGAATGGCGAAATTCTTCCAGTCCTTATATTTTATGAAATCGAGGTAGGCCTTCC
CTAGGGCGGAGGGGTGAGGGTAAAGGTTAAGGGAGTGATTGGTAGTTCGGGAACGATAGT
CCCAGTGAGTCTCGATGTGTGGGACGTCCAGGGTCTCGCAGGCTGAGCGCACGGCAAAAG
ACGCATCTGTAGACTGCGGCCCAAAGACGGCGGCAACCCCTTCTTCTAGCAATGTGCAGA
CTTTTCTAGAAGCTTGAAAAGAGTCAGCTCTCTCGATGTGCTCTACCCTAGTGAGTAGGC
GATCCGAAGTAGGAGTATAGCGTTCACCAGGCTCGGCGAGCCCATGTGAATTAATTCTCT
CGACCGCACTATTGAACGCCATCTCCACGATGTCTTCGCCTGTCTCAAATAATCCGCCTA
TTTTGATGTCGGCCACAGCGACGCCCAGAAGGGCAAGCGCCAACACCGGCGCTACCACCC
CGCTCATGGTAGCATCGGGCCTTCACGAGGCGGCAAACAAGTCGACTTCAGTGCACACAA
GTTAGTAACAAACTAAAACGACTACATGTGCG

>Vd22292 len=7715 path=[1649:0-3060 @17652@!:3061-5333 6949:5334-5335 @235@!:5336-5861 7467:5862-5960 7565:5961-5985
@828@!:5986-7218 8818:7219-7243 8843:7244-7448 9046:7449-7472 9070:7473-7483 18287:7484-7714]
TTTTTTGATTTTTTTTTACCTTAGGTGCTTTAGTTTTTTTTAGACATTATAATTTTATCC
AGATGTAATAGTAGTAGCAGTAGCAGTAACATCTAATCGGAATCAATACCAGCCATAACA
GCTTGATCTAGCTTATTTCTTTGATACATTCGCCACTTCGTTAACATTACTTTCTCGTTC
GCCCTCGAACGAAGCAATCTGCATTAACAATGTTCAACTTTGTGTATAATGCCATCGCTT
TTCGAAAAATAATCACACTGAGTTTGAATTGTAACATCGATAATTTATTATATATATGTA
GATATTCTGGCCGTATGCAAAAAAAAAAATGGTGCGAACTAATTTCCAAAATATTAGACAC
AAGACCTTCTCACGAAGGTTTTTGAAGAGGTCAGAAATTAAACGGCTGGCATAACCAAGT
CACTCATACATTTCCTTATTTTGTTTGTGGTTGCTTGCAATTATAATCTTTTTCTAATCT
AAGCGTATTATAATCTGTGTTTAGTTATTCGTGTTGTATATATATGACGGGCTTAAACTA
TGTGCTGACTAAGTCATCAGCACAATTGCGAGAGGTTGTGGGCCTTCATTCAAACCATTT
ATTTTCGACGGCGGTTTAAGACAAGGATTTGAAAGAGTCCATGTTGGAATTGTTGAACCT
CATCATTTCTCAGGACACATTAACCGCATGGTTTCAAAAAAAAAAAAAAGCTTTGATTTCC
ATTGTAATCTGCCTATTCAACAAACAGAGCAGCTAGTCAAGTACTGCGTATTCGTGAGGC
GTTTGAGCATATTTCTAGATCGCTTGCCTAGACCCTATCCTCCAGTATTCTAAGTACGGC
GCCCCATCCCACTCCATCCCATCTCAGATCTGCTATGAGTAAGAGCTCTTGCACTTAATG
ATGAGCGCGAAACCCCAACTGGTTCAATGTTTTAACCTTACTTTACCTAATAAAATAATT
TGCCAGGCGCTAAAAAGTTTTCGTCGTAAAAGACTAGCTGCCTGAGTTATACCGCCTGCT
GAGTTTAAGGTAATCTTACTCGCCTGGAAAATTGACGTGTTTCGGAATGAATAACAATCG
TTCGTATTGCTCCAGCAGGTTTTTATGCTCCGACTTGCTTAATACTAAACTGCTGGAGCT
TGCTTGCGATATTTCGATAGAAAAGGAAGTGTCATAATCAACAATCGTGGTACAATACAA
AAGATAAATTGTAGCTTCTGATCATGCAGTAGTGATTTCAAGTTTTTTCTAGCACTCAAA
TAATGCACCGCTTGGGTAGCGAGCGTTAACAAACAACTAATTTAGAAACATGGGAACGTT
TTTCAAATTTCTACTCTTCGAAACTGCAAAAATCAGCTGTTTCAGCTTCTTGTTTATCGT
CCCGCGCAGACGAAAAACGTCGCCATTGGGCTAGCGTCAGCCAAAGCGTGGACATGATGT
GGCAACGATATAGCGAGTGTTGTCCTAGTACGTTCAGCAACGGATAACAGGATGTATATA
TATGGCCAGACAGAAATGAGAAGGAATTAGAGAACATTAACGTACCAAAATGGTACCCAA
TAGAATCTCATTGGTGTCGGGATAGCGAAATCGATTTGTCATCACTTAGACATTGTAATG
GTCTTAAAGTTTCCAGAAATAACTATTTGCGTTATCTATAACGATTGGGGGCTGCAGTGT
GTAGTCAATCAACAATTGATCGCGTCGATATTACATGCCAGTTGCTAGTAGTGGTATATC
ATTTGAGAATTATTATAGCGACGAAATCCTTTGGGGGACGGAAATTGACAACACCGGGAG
ATGGCAAAGCTGGCAAGGGTATGGGTGGACATGATTGCAGTTTAATCTGTTAGCCCTGAC
TTCCAACTCTGGTCCTGATGTAAGTTAAAGAAGAGTACTGTTGTCGCAATTCTATCCGTT
GTGAGTAAAGTTCTAATGCAATAGCGTTGCCCGTACAAAGTAATTGGCGTATAAACACAA
GACAGATCTCTGTGGTATCATGTACTGCTGAGGTCACACTGCCGAGACATAGGTTCATAT
ATATATTAGCTATGGGGCGAACATCTTTTTAAGAGTCACTCGTAATCACATTGTTTCTAT
GCGCAGAAGTACAGCTCGTTGCGGAATCGACACGTGTAATAACATCTATTAAATCAATAA
CCCGAAAACTCTACCCGCTGTTAAAGGCCGCTTATTTTGACGACCTTTATCCAAAAAGAA
CCTTCTCTCATCCAAAAAAATATTTAACATATAATAGCGGTCATTGCAGATGCTTATCGT
TTATGTATCGTGTTAACTATGGAAGACTTCACTGGCGAAGCCGTCTGACACTTCGCCTAC
GTAGAACATCAGGACCAAAAGAGTCAGATACGATCATCCGTCGTTGGCGCACAAACTAAC
AGGGCATTTCATTATTTTACCTCCCTAACGCGATTACCACCTCCTTCATCATTTTAATAA
CGTCCCCCTTTTCCAGTCACTTGATATTTTTTGGGTGATGTAATTAAGTACTGTAATGGC
AATGTGTTCATAATTCAGTTTTATAGAGCAAATACCTATATATTACCCATTCCTTTCATT
ATGTTGTGAGTAACTGTCGGTCTTGAGCTGCCTGCGATGTATTTCCCTTTATGTTGTTGT
TGTTTTCTGGTTTGTTTTATTAGACATTAATGTCAAACGAGACGGGCTTGTTCTCCTGAG
CGCCAAGGAGGTAACGCGTAGAAGCGTTCATTCGGGTGCCGGGCGTCGCCTGGACCCGCG
CAGGCGGTGGTGGCGGTGGTGGCAAATCCGCCAAATCGGATCGTGTTTCATGGCTGCTCT
TTATCGACGTAGAGGCCGACTTTAATCGACTGAGACCCTGATGACCCAGGTTTTCGTGTT
TACTTGCCCCGCTGTGGGCTGGGACAACTGTCTGAGCAGACGGCATTCCGGATGCCAGCG
ACCCGATGAGCACCGGCAGCTGCTGAATGTTCGATGGCAGCATTATCCGATGGTGAGACG
GGCGACGAAGAGATCTCCGCCGCTCAACGATGCCACGCCATTTTGCTGCGGCCTTCCTGG
CGATTTGCATCCTTCTTTTCTGTTTGGCCAAGTAGCGTTTATAGTTGATCTCAATAATAA
TAAGCACCAAACCCCCGAATATTCCTGCCCCAACCAATATAAAGACGCCTGCCATATTCG
TTAGTCCAAGGGTTGCTGGCGTCCGTTCTAGCTTGCTCTCGCATCGCCGACCTCCATTGT
GAATCCAGAGAGAGTCCAGTTGCTCCATAAAGCCACTTTCGTGCATCTCAAGCAAGGCCA
GTGTTACTTTATCGACCCAAAACGAGTTCCTTTGCAGAGCCACTCCGTAACCAGAGCGAC
CAAACTGTTCGCCAGCTGTTAGTAGTTCACAGTGGCGCGCAGCCTCGTACTCCAGCCGCG
ACGAGTCCCAAATGAACGCATCGATATTTCCGTTCATTAGTGCGTGTATACCGTGGTCCA
CCGTGTCGAAATTCTTGCCTTCCATGATGCGGTACATGTTTTGCAGTTCCACCTGCCGCT
TAAAGTAGGTGTCGACGGCGCTGCCTCGCACAGTGGCGTAGGTGAAATTCTCCGAGGGGT
TGCGCAGACGTGGGTCGTTGATGCCTGCCAGTGATGATTCAGGCTTTTCAAGCACAAGAA
ATGCGGCTAGGTTGGCAGTGTACGATGCCACAACGATCATGGCGAAACCTGCCCATACCA
TACCCAGTACTCGTCCCGAAAATGACCGAGGAGTTCCCTCAGCCATTCCCGAATTGAGCA
GGACTCCCCAGGCAAACCACAGTGCCGAGGAGAGATTGAGCCCGTCCTCATCGCGCTCGC
TGGGTAGCACCCGGTAATTGCCAAACGGACTAAATCGATCCAGCAAATAGAGTCCCAAAG
CCACCACGTGCACTGAAACCACTACCAATATCCACAGCGACTTTTGGAACGGCTGCAGGA
ATGACGCCAGGGTGGAGCTTTTGTCTTGCTTCTTGGCTAGGATGGTTATTCCTTGGTACT
TGAATGGCTTCGTAAAATCGACCTCCATTGACCTCTCAGGGGTAATGGTCAAAGGAGCCA
CGACCATATCAGCTTTTTTACGAACAAGGTCTCCGATCAGACCCGTCCATACACGTTTCC
CTTCGACATTCGAGTCGAGGTTTCCGTACTGTCCGTCCTCGACGAGGTAGAGGTCGAAAG
TAAAGTTTAGCTTCGAAGCGAGGAAATGCAACAAATCCATACAATAGCCAGAGCAGCAGA
AGGCCTCGTCGTGGCCAGTGTCAGCGGTCCTATTGTACCACGGGCACAGAATCTCTACGG

ATGAACACTCCGTCGAAGAGTTAACTTTACGGCTCCAGACGAATGGACGCTCGGCCAAGG
TAGCCACCCGAAGGTGTTTAGGAATGACGAAGCCAAGAGGCTTTTCTGTAGCTTTTCCGG
GCCAGGAGACTGCATTTAGGTTGATTTTGAGTTCCATCTCCATATCGTATTCGCTATACA
TGTACTCGCCCACTTTCACTAGGTAACTCTCTTTATCTCGTTGGATGTTGAATATATCGT
AGTCACTATTAAGGCGATCTCCTCTCTGATCAAACTTTACGTGGCCGTTCTCTCCGCTTT
CTAACACCTGCTCCTTAAGAATACTGACAAGTTTGAGTCCAGCATCCCAGTTGTGCTCGA
GATTACTGCAGTTCAGCTGAGGAGCGGCAACTTTGGAGTCCTTCTGGAGCTTCTTAAGAG
CGAGGGCGATTAGGTTTACTGAATCGCGGATGTGGGCTCGCTCGTCCACCGCGTTGACCA
ATTCTAGTCCCAAGACGCCATCGGGACAGTTTGGTGCCTTGAGTGCCTGCTCCGACACGA
TCCAAACATAGCCTGAAGTGGTCATATCCAATTTCTTGACAACGTCGAAAACCTTCGATG
CTTCGCTGGTGTCGGCGTATAAAACGTAAACGCGACAATGTCGCCTCGAGGCATTTCTCA
GCGCGGCAGTTAGGTCCGTCTCACTTACGCCAGACTCGAATTCGATCACGTGCTCGATGT
GCACAGCATTTTTATGTTGCGCTGCGACGCTGTGGAATCGTCCGAGTGTAGCGCGACCAT
CGTTATCCGACGAGTGAACGAATACAACGCTCTGATAGCCCAGATGGCGGAGAAGCTTCA
TCCAGACATCAGCCTGATGAGAATATGGCGGCACCGTGCGCAAGAACGTCCTGTGCAAGT
TTTTATCCGATAGCGAACTGTGTCGAGAGCTGATTCCAATAACAGGAATGCTATAGAAGC
CGCATGTATAGGACACTGCGGCCGGTGAAAGTTCACCTTTTAGCGGGCTTGAAATTACCA
CTGCATAGACTTGTTGTGAGATGAGTTGCTCACAAACGTTTTCAGCCATGCGAATAGGGT
TGAGGCTCATGTGTACGCTCTTAGCGACTAGATCGATATTATAAGTGGCCATGGCGTCCA
AAATAGCCTCTTCAAAGTAAGCGATACCTTCCGGGTGCGATAGTACTGCGCCCACATTAT
ACACCCGTGGAAGCTCCTCAGCGGCCAAGTTGATTGCGTTCACGGATAGATCGGCATCTA
GTACGGCCTTTGAGATGATCTCCGGGTCCAAAACGGAGGCACTATTACCATTGGAGTGCA
GATCTGCTGTGACAGTCGGCACACTGCTGGCAAATACGCCTGGTACCACTAACAGGGATA
GTAAGAGATTCGCCCCTGCTCGCCCCGATCGGCACTCGAAGATAGTCGGATAGTGCACTG
AAGTTTTTACCCGTCTTCGCATGGCTTCGATTTGCTCCAGAGCCTTTAAGGCTTTTTGAA
CGACTGTCATGATGTTGTGCATCAGCGGTAACTCGGAGAAAATTTTTTGTTCGATTTCGG
CGTGAAACCACACCCTTACCCCTCAAGTTTCTATGCTTCTAGCGTATTTCGCATCCATTC
TATCCTCCGTTGCGTAGCCGCTTATCTCCTATTTGTTTATCTGCGTTCTACGTCGCAATA
ATCAGTATGATATAGATACGTGGGAATTCACAATTTACGTACTGTCGAAAAATAGTCGAT
TGTCACTAAAGAAGCAGGAGTTTAGCGATGGGTGAGCAGTGGCTGCAATGCGGTGCCAAA
TTTTTTTTCCTAAATGAAGGTTTCATTTAGCACCTATAATGATCTCTCTTTTGGCAGGCA
GACTACTGCGGTCCTCGGTGCTTGGCAGGGTTAACGGGAGCGCAGGAGGGTCCTGTTCTA
TCGTCTCTGGACGAAGACACTCCGTGTGTATTTGTGACGCCAGATGAATGAAGACGATTG
TACTTCAACGAGCGGTGCCTTTTAACTAGGTGGGGATGGGGTTCTATAGTCAGCATCCGC
ATTCATGTCATATTAGAGTCGGCTCGAGTACTTGTGATAATTTTGTTTCGACCATTTGTT
ATGTTTGTTGATATGACGTAATTCACTTAATTCATTCCCGCTGGTCGGTGGTTTGCTTTG
TTCCTTTCTTCTTTGGCTCCCTATCGTGTTCGTCTCTAGACGCACGCTCTATGATTTCGA
GAATGAAGGTAGCTTTCGAAAAAAGCAAAGCGGACAGGGTATATACGTACGGTAGTATCG
CGATATGGCACGCCTCTTAATTCCATCGGTTTTTGCTGTAGAAGCAAGTACCTGTTACTA
GTGTCTAGTATGTGGTCATCAAACGTCGATAGCATAGATGATGGGCAGCCACTGCTCCCC
GAGGGCTCGTATTTACTGATGTCAGGTCGTTACACGGGACAGACGGACGGACGGCGTTAT
GCACCTCACACATGCTTTAACTAATTAAACAGTGCAATGTAGGAGCAGGGGCAAAGAGGC
ATAAATTATAGGTCAACTCAAGGATGAGTCCAGACGATAGAATGCAGATAATAGGTTTGC
ATGCACTCGTTTACCGGGAAGCTGCTCGGTCGATCGGTGGGATAGTCAATGGGTCAACGG
CACTGAATAGATTATCGTACTGCTTGGATTGATAGATGACCGCAGACGGAGGCCGAAAAC
AGCAGCCTATGAATTGAAGTCGAAGTGGAAGTTAATGTTGAATTGAACAAAGCGGCACTT
CACAATGCCAGCGGCAACACCGGCGAGCAGATGCTAAGGTCGTTCAAAGAGAGACAAATA
GTGTCGCACAGGATGAGCTATAGTAGATATAAGATAAAATTATGGCAACCGTATCAATGC
TAGTAGCAATAGCAGAAGCTGTAACCGCCATTAGTCACAGCTTTTGCAGGAAATGCCGTT
AGAGAAATGTCACTACACGAGAAGATTTTTGTGTGCAGCAGAACAAAGATTACAGCAGAT
AGTTCTACATAGTTGTCTCTAGATAAAGAGACAGATGGTTAGCGATAGCAGGTAGGCGAA
AAGAGCCAGCGGAGTATCGCAGTGGGAGTGTTGTTGTATACAGGCGGATGATGGCAGTTC
GTAGCATCAGGGCATCTGCGTACGCGTGGCTGCTGTGTGTCTCTGTGATCCGCCCTGCTC
TGTCCTGGCCGTATAACTATTTGCCGTGCAAGTAGCTGCAATAGAGAAACATAGTACGTA
TGTCGAAGTCGGCTACATTTGTGTACGTTGCATCGAGGTATGGCAGGGGAAAAGCGGAGG
AGGTAGAAAAGCCGAAGGCGGATCCTGTTGTTGATGATCCTACTGGTAAAGAAGAAGCAA
GAGGAGGACAGCTAAAATTAGGTGGTGAGGGGCTG

>Vd370 len=4530 path=[669:0-2306 2975:2307-4529]
TTTTTTTTCACCGCACAAAACGCATTTATTAAAACGTAGAACAGGTACGTTATTTTCTTG
AACAGTAAAATTTTATTAACTTTTAGGTGAAAATCAATTTGTTTTGTCACACTAAAAACT
AGAAACACAAATAGTATATACGGCGTTTGCAATAGATCTTACAATACATCGATTTACCAT
GCATGAATAGATTTGACACTTATCAAAATTAAAAAAAATTTAATTAAAAACAAAGGTAAA
AGTTGTGTTGAAATATTCGATGGCCATTTAGTGTTAACTATAGTGGGAGCTTTTAATTTT
TTGTAATGATAATTGAAAAACGATTTTTTGTTTTAAGCAAACATCTACACAATGTAAATG
GTGCATTTTATTAATATTCTATACATACTGATCAAAATCGATTTAATATCGTCAGAATCG
TTTGAAAACCATTGAGAACATCATTTATTGGTAGCAGCCAGCTCTAAACTTGCGCCACTA
GTTCTGTTACCGCGGCGCCAAAAAGTTATCTGTGTACTTGTTTACAGCGCGCAATTATTT
TTAAGAATTATGTGTCTTTAGCGTGTCGAGAACTTGTTAATAACGAAAATCAAGGTCTCT
GGATTGGATGCAGCGGGTTCGTGCTTGTAGGTAGTGCCTGTTATTGCATTTATGGGCTAA
TATAGGAAATTACTCGCTCTATTGTTTAGTTCGTTAGAAACAAATATTTGATTTATTTAA
ATTTTCTGCTCGATTCAGGATTTCGGGCACAAAACTTAATGTGTTTTATTGATTGTCTGT
CTAATAATAGTCGGTTTAATGTCCGGTACAAACGAGCTCGTGGATTAGCTGGTTTGTAGC
TAAGAAGACCAACGTTATCTTAAAGCAATGAATTTGTTTCGTTAATGAGCGATAACAAAA

GTCACGCAGAATTTTGCAATGTGTGCTGTGTGCCGTATGAGAATATAATCGTTTATGTAT
AAAGCGTTTACTTCTCATTGATATTGCGGAATCTTCGAGCTCAGTGAATCCGTTTTCCGG
GGAGACGTGGGGTCGTTAGATTTCTGGCCATTTTCTCTCGTACGATTTTCGCGTCTTTTT
TGTAGTTGATCCTTCCAGTGATTCCTCGGCAGAGATGTCTCCGACAGGTCAACTTGATTT
GTTTGCATTTTGAATGCAAATCGAATGTGATCGACAACAGTGATCATGTTGAAGTACGTG
TCTCGTTTTTCTCGGGTGAGATGTTCGACGATGACTTCTCCAATGTTGATTAGCAATGCG
AAACAAAGACCCAAAAAGAGTAGAATAAAAGCACCTGAAAGGTCCCCTGGCGAGAGGGTT
CTGAGTTCGACCTTCTGCTCATTGATGCTGTCGTCATCACCATCATCGACGTCGTCAAAG
TCACCGTCGTTATCTACATCGCAAAATCTTTTGGTCTTCCACCATCGCTCTTCACGTTCC
TGAAGAATGCCTTTAGCTTGTAGGTTAAGTACGATTTCAGATAAAAGGCGGTTTGTCTTG
CCGGGATTCTTTTTATTTAAGGCAATACCATACGAGGCATGATTGGCAAAAGGTTTTGTC
ATAACGAGATCGCAAGAGGAAGCCACCAGATATTTAGCTGAGGCGTATTCCATAATGAAA
GCTAGATGTTCATCTTGGGATATCTTGGCTAGGGCATGGGTCATGTTTTTGACTTTGGGT
GTTTCATCTTTGAACATTCGTTTGTTGATTTCCCAAAGAACAGGTGCATGCGATGTCATG
AGTGTTCGGCAGGTTGCTCCATGCTCAATACACGTGAATTTCATTCCGCGTATGCGAAGT
AACCCTTGTATCGTTTCCAGCTTCGAGAAGTCTTCATCCTCATAAAGGACACTTTCACAA
AGGTTTCCTACTATGATGGCGAGAATGACAAAAGTGAAGAAATACCAGAACCCAGCGAGA
ACACGAGTTGCGGTGGCGTTTGGATCGAGGTGCATTCTTTGATGTAATAACGTCGTAAAC
ACAAACAGTAAGCAGCTAACAATGCCCAGTTGGTTATCTACTTCTTCCGCCGGCCCGTTT
ACGCCATCGCCCGTATTCACGTACTCCGACAGGCAGAAACGTGCGCACATGTACATGATC
AGTGCGGTCAGGAGTCCAGCGACCACCGTATACATCCACACCTCTCTAGTTAGTGGCAAA
AAGAAAGACCAAACCCCTCCTGGCTTTCGTGGGACGGGGGCTTTGACAACGGCCACAAGT
TGGGCCGTCATAAACGGCAGGGTGAAATCGACAGCCCGCAGGCGCTCATCCGAGACAATC
AGGTCGGCAATCGCCACGTTAGCTTTCTCTTTGACAAGCTCTCCAATCATACCATTCCAC
GTGATAGTGCCGTTCTCATAGCGTGTCTCAACCCCATAACTTCCGTCGGGGGAAGTATAC
AGCTCATAATCCAGCGGTCGTGGCGACCTCTTTTTCATATCCTCCAGTAGATCAATCAGA
AAGCCTTTGTATCTCGGACGGTCCTCTGTCGATAGCTTCTCGTCCTTCATTATCCATGGA
GGAATCATCACTGTGACAATCCGAAGATGTATATCGTCGATCTTCGTTTTTTCTTCCTTG
CTCAATTCAAATTCATTTTCATCATTCGTAATATTTAGTCCGCTAGACTTCGACCAGATG
CCAATCTCCTCAAGTCCGTCAGTTCCGAGTTCAGTAACGAAAAGCACAAAGTCTTCTCTG
ATACCCTTCTCGTTAAAGTTCACACGGCCCGTTGCTCCTTCGAACGTGCGCCTTAACATG
GTCTTGGTGAGCTCGGTCCCCAGATGTTCGCTGTCGAGGGACTTCCGACCGTTACATTTA
GCCGTGGGTAGAGCTTTTGTGTACGAACTTTTTTTGTTGACAAGATCTTTAACTCCAAGC
TTAAACAGCATAATTGCATCGTATATTAACGCGACCTCCGTCTCAAGGCCTGGTGGGAAA
CTGGCGTCCTCATCGGCTTCTTCCTCTTCATCAGCTTCACCATTATGTTCATCGCGCTTC
AGAAGACGGCGATCATTACGGTCCCGTCTGGCGCGCGCCATTGCGGCCTTCTGCTGGTTG
TGACGTCCGGACGCTGAGCCTTTCTTATTGTCCTGAGGGCTATCTTGGCCCCGCAAGAGG
TCATCGGCAATATGATGCTTCATGTCGACGAGGCGCAGAGCTGTAATATTGGCACGTGAA
TGCATGAAGTCGTTCAACTTGGCCACATGAAAATCTAGCGAAAGGAAGAAGTAGTTGTAG
TTCTCATTAAGCATGGCCACTTGCATGGCCTGTTTGAGAAAAGATTCGATTTTCCCTTCT
TTGTCCTTTTCCCGCTCGGCTTTTTGCTCCAGATCGACGACAACATTTCGTATGTCGCGC
TCCTTAATCTGGCGTAAAAGCGTCCGGAACGTTTCGCCTTTATCCTTCTTGAAATAATTC
ACGGTAATCCCGTCTTCTTGAAGCTCTTGAAGCAGTCGACGCATTTCGATGATATCGTCA
TCACTTTCGTATATCAGGGCGATATCCCTCCATTTCCAGTACTCCATAAGGGCTGTTTGA
GCCTCAGCAATCTGTACGGGATCAGGGAATAAGTTCAGCGAGTGTTCGTCATCAATCAGG
GACGGATCCCAACGAAACTCAATATGAGGAAGATCAAAATGGCGAGCCATAGATGAGACA
TGAAGAGAGCTCACTGGTGACGTTGGCCCAAAAACGACGTTTACATCTGATTCCATTAAC
GCGCACATGCTTTGGATGGCTTCGAGAATGTCGTCATTCTGAATGACTTTGGAGACAGCC
TTGAGGTTCGAGTCGACTAATTTAGATGCGATATAGAGGAAATGTTTCGCAAGATCCTGA
TCCTCAACGATGATTCCAATGGTGCCGACATCTTTGTGCACTCGGGCTGGTACCGCGCCG
GTTGTATTCATCCAGCCACAAAGGAATAGTACGCTCAGCGTTGTGACTCCTGCTAGATGT
CTCGATCCTGAGCGGTAGCCCATGGCGCCGTAGATGAATCTTCACTCTGAGCTAATATAC
TAGCACATCGGTTATCGGGTATTGTTAATATTAGTCACGACACAAGCATACAGAATCCGA
ACAGCGATGCACGCGACAGTTCACGTTCATTTCACCGTAGCACGACACGACGATGGATTA
GCACGGATACGTGCCAGCAATAACGGTCAAGCGCATTTTAACTGTTAGTCAATAGAGCGA
ACCTACAATCGTTCTTAAGTCATAGTCGCTTCTCATAGAATGGTCCCACAATGTGTTTGT
TAGGTATCGGCGAACGTTAGAGGACGGAACAGAAAAAAGCGTGATGATGGCAACACAGTA
AACCAGCACAGCAGAACTCTAGCTTCCCGTGGTATCTTGATGGCGAGTTCGCCTGGCTGC
TGTTGCTGGTTTGCGTTGACTAGAAAAGAAGAGGCAAGCGAGCTTTCGCGGAAAGGGGGC
CAGGTTCGGTCAGAAACCTGAAAGCGTGCCTCTTTTCGGGTGAGTCGTTCGAGATTGCCT
AGAGCCGCTCAGACTAGACGACAATGAAAA

>Vd52944 len=4189 path=[1:0-4188]
CAACGATAACCCACCGGCGACAAAAACCAGAGCCCCGTGGCCAGTAAAGAGGGCTGCCTC
ACATCAGAGTCTTTTTCGTGTTTAGAATCGTCACGGTCGGTACTTGGACATCGCACACAG
TCCACAGTGATCTGCTTGGCCCTAGGCTACCCGGTTATTTTTAGCTTGCGAAGAACTCGT
CTGTACCGCCAGTCACTGCCCACTTGGCCTGCACGAGTGTCAACAAACCGCTATTTAACT
CTTCAGAACAGACAAAGCATAATTTCCTTCAATTGACTGTGCCTAAAGACAGCGCCGCAA
GTTGAAGCACAGTAAGCGGTGCTTATGTGTCCGTCTCCATAAGAAGATATTGTTTATCGT
ACGATAATGTTTGGAAATTGCTGTTTAATAGGTTGAGGTCATTTTAAATTGAGCTACTAT
CGTTCGGGAAAGAACACAGAGTGACAAAATGACTTGGCTGTGTGCAGTGTTAGCAAGTGT
AACTGTTGTATCCATCTCGTTTTCAACAGGAGTGCACGGTGGCACCTATAAGATTGCCGG
TGTATTCAGCGCAGAGACTGGTCAAGTAGAATCTAAAGTATTTGCGCGCGCAGTCGATCG
CGTGAACACGGACAACCGGGAGTTCCCGGAAGTGCAAATGCAGGCCATACCGATCATGCT

GAAAGAGGGACAAACCTGGGAAAATTATCAAGAACTGTGCCGTCAATTTGAGAGCGGGGT
TGTGGCAATTGTCGGAGCGGAGTCATCGGTTGAGGACTCGCTGATTTCATCGGTATCATC
TGCCGTCCGCATTCCGCATATTCGTACAGGTTTCAATCGAGAGCTCGAAGATCCTGACGA
TCTATCGCTCGGACTTGCCCCGTCGCCATCGGATCTTGCTGAGGTTGTCAGAGAACTAAT
ACTCCACATGGGCTGGAACCACATCGTCTACATTTACGACACGAGCACTGGGCTTTTAAG
GATGAATGAGCTTGAAGAAGATCCAGAATTTAAGGGCCGTCGTCTTGTTCCTATTGATCT
CTCCGTTGACGCAAATCCCTGGGACAATATTATTAAGCTTTCGATTCGACACGTTGTGGT
CGACATCGAAAGTAAATCCGTCCATCAGGTCCTCAAAGAGATTTCCATGTCGGGCCTGTC
GCAGGACTACTATAGCTTTATTATGCCAAACAGCGATATCCGATTACCGGAAAACTCAAC
GCAGCGGACGTTTTTCATAGATGCCGACGTCTACACGTTCTCACTGGTCGATCCAGACAA
CAGGGAAATGCACTGGATCTATCGTATGCTTGAGATGAACTCATCTGAAAACGAGCCAAA
CCTACCATTCATCTACGATTCGGTGCTCCAAATCGGACACACGTTAAATAACCTCACCAA
ATTGCAAAGGCCAATTAGGAGTTCCCCTGGCAACTGTACCTTAAAGGTCGCCTTTAAAGA
CGGCCGTAAACTATATGACAGCATTCGAGAGTCAACGAAATTTGAGGGTGTCACAGGCTT
CGTCGAGTTTGATGATAAGGGGGCTCGTAAGGTCAAGCACGTTCACGTCAAATTGCTGAA
AGATGGCAAACTAACAAAGGTAGCCGATTGGCGTCCATTGGATGGTCTTACATTCATTAA
AGAACTGGATCCAAAGGGCGAACCAGATGAAGCCCTTGAGCACGATGAGGGTCTCAATAA
TCGAGCCCTCGTTGTTACAACCATTCTCTTGCCTCCGTTCTTGATGCGCGATGACAGTCC
CCATCTAAAAGGCAATGAACGTTATTCCGGCTTTATCGTGGATTTGTTGGAGAAGCTTGC
ACGACTACTCAAGTTCCAGTATCTCATTAAGGAAGTTGAGGATGGCGCTTACGGGACGTA
TAATGCGACCACCAATAGCTACAACGGAATGATCGGAGAAATACTGCTTGGCAAAGCTGA
CATCGCAGTTGCCCCGTTTACAATTAACGCTGAGCGTTACCAGATCCTCGATTTCAGCTT
TCCATTCCTCGAGGCGGGCCTGGGACTTCTAGCTAGACGGGAGGTCGACCCACTCGAACA
CAACTTTTTAAGTCCGTTCTCCGCGATAATGTGGTTCTCTCTGTTCGGGACAATGATGTT
CGCCACATTCGTTATTACGTTCATCGCCCGATTATCGCCTCGGGAATGGTCATCGGTTAC
CGATACCGTAATTGAAACACATTTCACCGTGGGAAACTGCTTCTGGTACCTCTACTCGAC
CCTCATGTTCCAGAGATTCCAGCATCGACCGAATTCTCTTGGAGTCATAGTTGCATTTTT
TGCCTGGTTTATCTTCTGCTTGGCTATTATGGTGTCGTACGGAACAGTATTGGGTGACTT
CATTATCAAGTACAACGAACGGCCGGCCCTCTCGAAGATTGATGATCTTCGATCAGAGAA
TAACCGTGTAAAGTTCAGTGCAATCAAGACCGGATCCACGATGCGTTTCTTTGAGGAAAC
GGAGTATCCCAACTACAAGCAGCTGTATGCCAAACTGGACAAGGACGCAGCAGTTCGAAA
TCTTAGCGAAGCTATTCAGCGGGTGAAAAATGAGGACTTTGCTTTATTCTTGGAGACACC
CTATATCGAGTATTTCGTAGGGCACGATTGTTCACTGCAACAGGTTGAGGGCACAGTGGG
AAGTGTAAGCTATGGAATTGCGATGTCACAAGGGTCATCTTTACAAAAATATTTATCGGC
CGGGGTATTGAGTCTGCGTCGTGAAGGTTCACTGCAATCTATGCGTGCAAAGTGGTGGAA
CGAGCAACATGCCGGGCGTCATTGTCCAGATCATCTACACATTGTCAGGCCCACTCCGTT
TACGGTTCGTTTCGGTCGGGTAAACATGTTGTTTGTCTTCTTGTTAGGTGGCATCATTCT
CTCGGTTGCCATCACAATTTACAATTACGTCCGAAAACAAATGACTATTGAGGACAAAAA
CGAGTTTCGGTACTTTTTACCGACGCTAATCCGAGAAGTTGAAACATTGTTCCGAAAACG
TCCAGTTCCAACTAATCCCTCCCCGGAGCCGACAGCAGCTTAAGTTTCAACAGCTGAAAG
CAATCGCATATTTAGTGATGATGCAAATACTTCATCATATGGCATAAGATACACTTTTGA
AAAAAGACATATATGTTAGGTTTAGTTATTACTATGGAATCGTTTTTTAAAAAATAAGTG
AACAGATTTGGGTTAAACGTTTTTCGTGGATGTGTCAAGAAACGGTTGGGTGCTTAGAGA
ATTGTTAGTAGAGGAGTTTCTGCGTCACAGAAAAACGGTGTTTAACGAATATGATCATTT
ATTAGTTATGTTATCAACTATTATATACAACTGTGCCATAATTCAACTAATCTATAGTAG
TATGCAACAGTTCTGAAGATGTACATTAAACATATTTACTGTTTTGGTATTATATTCGGC
TTACTGAACCAATAGAGACAATGTACTCATATTTGTTTGAATTTTTCATTGATAAAGGTA
ATGGCTTAAGTAGTGTCCTCTATCAATTTACTCAGATTTCAAATCAGAAATGGAAATGAT
AATTAACTGTAGTAACTAGTAAGTATTTTTCCGAGAGACAGATACGTTCAGCTTGTTCAC
GATGTGTTCATTAACGAGAGGATACAAAATATCATGCAGGGATATCAGGAAAATACTGCA
GAGTCCCTATGCGGCTCTGACAATGCAGGCAGGTGACACGCTTATAGCACTGAGTGCATA
TAACTTTATATGCTCAATCGTACGCCATATGTTTGTCCGGAAGAATATATGTTAGTTATT
CCTACAATACGAGCAAAAGATCAATTTCGTTGTCGGAATTTTCTACGACGATGGCGACCA
TGCAGCATGGTTTAAAAGTAGTCTGTTTCGCAGGTATGTTAAATTACAACGGTGCAATGC
TGATATTACATACTAAAAAAAACATAGTTTACTGAGTTAGCCGTCAGAGAGATTTGCACA
GAAAACACTATTTACTGTTCTTCGTTATGCCATAGAGTCTCTAGTGAGGCAAGGCTCGAA
TCCATATAAAACTTGTTACGATGATCATAATGAGAGCGAAACGATTATGTGTTTCAACTT
TCTTTTTTCAAAATTATTGAGATACTACCAGCGCTACGGACAGTAATCTTTTACGCGTTC
TACGTAGAGGAGCAAATTTTATTTACGTGAAGTATATCGTGTGATGGGG

>Vd11771 len=4072 path=[1:0-3316 3318:3317-3325 3327:3326-4071]
TTTTTTTTTGTATAAAAGCGTTTCTAGTTTTTGTTTATACAGCAACGTTGAGATAAAGCC
ATCTAAAAATATATATTCCGCTATTAATACTTACAGTGTTTACTTTCTGCAAAATAGTTG
TAAAAAAATAGTAGTAAAAACCTGGGATCTACTTTTGTGTGCAAGTTCTATAGACGATTT
AATTGTCTACGAACTTCCGTCGGCACAGCTTTTAATTAAGAATTTAAATGCAACCACTCT
GTTAAAAGCTACAAACCTACTAAAACATATAAAATAACGTTACTGTCTGTCAGTACCATA
ATGAATCTTTCATCCATATCAATCCGATCTGTTTCTTTTTATGTGTGAGTCAATTCATGA
ACTAAGATTGTAGATATTTAAATAACTACGATATGTACTTCACGTCGCTTAAATCATTGC
TACTTGGTTGCTACGCCTTCACGTATTTTAACGAGTTTAGACCTACAGGCTTCGTGTTTA
GGGTCTCAGTCATATCATTATTGTACATTTAAGGTAACTTTCTATTTCAAGCCAATGGCA
CATCCTTTCTAAGGAAACGTAAAATCCCACCGAATGTATTCATGGTGTCAGGCTTTAAAA
GTATGTTGAACGTTTCCAACATCTGAGCAGACATGTAAGGCCCAATATTAAAGGGTAGCC
GCAGTCCCGTCAATTCAGATATAGCGCTGACATACGGGATACTCGAACGAGAAACGAAAA
CAGTCCTAAAGAGGGACCTAGTTTCAGGGGGAGCTGTTTTTCGGCAACTTTCTCTCTTAC

TCTTTCTTTCCCGAACCAATTTTTTTCACAAATACTGATTAATACGCACACACACACACA
TTCGCGTGTTCGCTTTCGTCGGCGCCTCTTCTATTCCCCCTGGTAAGGATTGCGGTTAAA
GTTACTGTGTAACTTTAACCGCAATCCTTCAATTAACAAAAAGCTTCTTCGATACAGCTA
ATTATACGTAGTTCAACATTTTTTGCAATGAAAGGCTTCCTCCTCATTTGATTTGTTTTA
TTCTTTAAACAGATCTCATCCAACATTTTGTTCTGCCCCTGGGTCCTTGAACAGGCACTG
TGTATTGGATAATACCCCATAATTTCCCATCATCTAAGCCGGATTACAAAATACAGCACA
ATGCTCTTTCAGCCCGTGATTTATTATGGTAACTAATCTAACTGATAGCCATTTAGTCTG
ATCGCTGCTGATCAATGAAAATTAGACTCAGCCCTCTCTTTTTGGACGCATCAACGAACA
ACGATATTCCTAGAATGACCTAGAGCTCAATATAGAGTTGAGGCCTAGCGCATATTTACC
CGCGTGCTGTCATGTCAAGGATTGTGTAGACCAGTTCTTGGTTAGCTTTGTCGAGAGTGA
CCGGGAGTTGCGTTGTATCAGACGACAGCTGAGGGACTCATTAGGGGGTTTTCCTTTGCT
ATTGTTATATAGTTATTTTTCTTGCTAATGTAACTATCGTCTAGCCTATTGCTGCTACTG
TTGCTGTGAGCCATAAGCTTTTCGAATATCTGCAGTTTCTATGTTATTGAAATCATTATA
TAGTAGTCTTTGGCAAATGGTATAGTTGTAGTTGTTCTGTTATTCGGTGCTCTTCAACTC
CTATCATACACTCAACGTTGAGTGACGTCTACCTAGCTGATTGTATCTTGTTGGCTATGT
TAATTTTTAACTCGATCATCGAGACATTCTGGTGTTGGTAGGTGCATTTTCTTCAGGAGA
TTCTCTTTTTAGACGAGTTGGTCGAGTTCGGGCTTCCTCGTTGCCTCTTGCGTAGCCATT
TTGTGGTACTTAGTCGGATTTAATTGCTCAGTTTGACGCGTCCGCCGAGAATGATTCATA
AGGAATCCGGCTCCAATAATGGAAATGATTATGCCAAGCAGCAGAACAATGATGAGTGCA
ATCTTAGCGAGTCCGGGGTAGCTAAGAACCTTATGCAAGAAGGCAACGATGCCAGGCCCA
GCCTCCACATACAGTTCTTGCCAAAATAATGGCATGAGCAGCTCTGGAAGCTTTTCACCG
TCCAGGCAGTCGAGCTGAGTTACACGGAAATTGGCCTGAAGCCTGCCGCGGACCGTTACA
GTCATGCCGGTCATAGGTTCATGATCCATGATAAATGTATGTCGTTCAGGGTCGGCGTTC
ATTCCTATGACTTTCTCTGAAAAGGACGGGTCGGCGTGCATGAAATGTGGCATTGAGAGT
AGAATTGGAGCACCTTGCTTGCACGGTCCTGCTTCAGCCACACCAGACGGCTCCCTATAT
ATCTCATCATAGCAAGCATTTGCTGGTTCAGCCTCGCGATTATAAAAATTCTCCTTTCCA
AGGATGAAACGACGCAGCCGGATCCCATTGGTATTAACATCTTTCTCATAGCGAATTGTT
ACCGGACGGCACATATCGGGAATATAGACTTTTTGCTCATTGGACTCATTCATCGGCGGT
CGCAGGTACCCGAAGGTTCCGTCAACGATGTTGCATGTGGTATTTTCGATGTTCGGCCAC
ATGGAAAGCACGTGTTTGTGGTTAATGGAGTAAATGCGATTGAGATCCATGACTCCCGTT
CCGCCAGTGAACATGTTGAATGTCGTTGGATCATGTTTGTCAACACCGAATCCAAACTTT
CCTTTTTTGTTATCCTTGGAGTTGAGCGCAAAAAATTGCCTCCAGAAAACCTTCCTTTATC
TTTGACAGTGCTACTAAGTTGTTGTACACTCCGTCGTAAGTTAGCTCAGCAACGGAATAG
TTTGCAGTCAGTTGCTGATCTGCAATTGCGGCTTTAATAACCGGATCCACCGCTAGTCGG
GCGAAGTAGCCTAAAGGCTTGAGTCTCTGAAGGGCCGACATGACAGGAACGTTAATCATT
GTTATACGTGTGGTATTCGGGAGAACGGAATTTTCCGCGTCAAACTCAAACTCCCGGTGT
TCTTCAAATGTTACTGTTTTATTATCGAGAAATTCGATATTTATTTTCTTCAGGATTGTT
TTATACCAGAAAGGTCCGACTTCTTGCAGAATGGGTTGTGTACCCTGCATAAACTCTTCG
GGATTGGTGAGATTGAAAAAGTACCAGGCTGTTTTTGTATCCAAATGGTCGGAAACGTCT
CGCCATATGGGAAAGCCTTGAGCTGTCGACGACAAGACAACCTTTCTACTGAGGATAAAT
TGAAACGTTGCCGTGAAGTTGATAAGTACCGCCGCCCCCAACACAACTAAGAATGCACCA
ACGACGACGCAGATCACCCCTGCACCTCGTCCGGCGTTGACACCCATCTTCAGAAAGTTC
AAAAAAGGCGCTGACTTCTGTTTTCCTCTGATTAGTTTCATTATATCACGTACGATTCAG
AATATTCGTTCTCACTCCGATGATCGTAATTGCAACGTCACAATAGGGACAACAAGAATC
TTCTGAGGTAAAGTAAAGTACAGAGGTGAAGCACAAATACAGCACAGCAATACACGGTAT
GGTACGCTACAATACTATACACACGGAATGGCACATTGACACAAAATATTAGGATTTCAA
CGCCGGTTTCGTACGTTTAGTTAGTGAAGTTACACGTTAGTTACAATAAGGCTATCAAGT
TATTTTGTATCGTTATCGTCGGCAAAGCTGCCCGTCCTCTTCAGCAGAGACTAAAAACAC
TGCGTAATTGCACCATTAGATTGCCGCTAGTGTCGCCCTTAGTAACAGTAACCATTCTTT
TTGCATAAGGCCTATTCAACCTCGCAGGCAGATTCGAGGCTGGAAACAGAAGATCGCGAT
CGAGAATTAATCGTTGCTCGTTCACTAGTTTAGCCGATTGGCCGATCCTCCACAGGCCGT
CGCCAACCCACGATTGTGTCTGTTTAGCTGCTGCATCAGTCGATCGCTCGTTGGTTCATG
CCTTTTTGGCCTCTCTCTCAGACGAAGAACGTCGGCCGCTGTGAGCAGCAGGCTTCCGAT
CAACTCTCTCGTAATGATGATCGTCCGATCCCAGTGCTGTTGCTAATACCTAACCCAACG
TTTCTATGAGATTGCGGCAGCGACCGCAGCAGCGGCGACGGCGGCGGCGGCG

>Vd15725 len=5002 path=[157:0-3164 78:3165-3351 9916:3352-3615 10180:3616-3634 3507:3635-5001]
CTCGTCGTGATCGTCATCAACAACTGCGTTCTAGTCGTCATCCAAAACAGCGGCAAAGCC
GCCAGCGACGCGAACCTGCGTCAGCGATGCCCGACCAATATTGTGGAGCTAACAAGATTT
ACTTCGCGCAGGCCTTCCCGTGTTGGAGCTAATAGTTTGACACTCAGAAGCAAGAATATC
AGTGCGCACAGCGCGGCGTGAAACACCCTCTGAACTGAGCAATTTTTGCCACCGCCTATA
TACTACACATAAAGAACATTCGCTTGTAGAGAAATTTTGTCATTGTCTGTGTGCATGTAT
ATGTGTCTGCAGGTCTGCATGCAAAAATTGAGAAAGTAGCCAAAGATCGCCATTCGTGGA
AGTCAACGATTACCATCACTGGTGCTGCTATGTGGCTTCTCAACGCTACCACTTCAATGT
GCAAGGTCGTTGGATTGACTAGCTATAGTATGCACTCGTATTGTAATCGCGGTCGTTCTA
TTCTATATGTTAATGTAAACAGCAGCAGCGGCACCACTAGCAGATTCAAGAGCACCATCT
GGTGCTCAGTGAACTGAGGGGCATTATTGTAACGTGCTGTAAATAACGGGTCTTCATTGG
GCATCATAGGTGAATGAGTGCTTAGCGAGCGGGCTGTGGTGATAATGGCGTAAGGGCGTC
TGCACTTTCGACGTCTTGTTTCGCGCGTAGTCGCTGGCACCATCAACACCATTGCTGACT
GTTTACTGCATTCAGTGATGGTATGTCTTGACAGACATCCGGCATGCGGTGCCGCTGTGC
TGCGCTACTGGCCCGTATCTGACCACACTGATCCGACAGTGGAAAGAGCAAGAAAAGCGA
CGAACAGCTTGTGAACCAAGCAGAACCGTTGTTTGGTGTGTGTATAACTAATATGTGCCA
ACTTTGAGGGCGCCGGATCTTGGCAGGGTTCCAGTACCAGCAGTACCCATACCTATTAGA
CAAACCCCGCAACTGCTGGCCGTTACGTGAGCTGCTGATCTATTGTTACGAAGGGTGTTT

CTGCCTCTGCTGCTTTAACGTGAACATCTGACGTTTTGGTATCGGTGGGTGCCGTTAATA
CAACTATTGCTATCGGCCCTTAGTGGCTGGCATTGTTGCCGCTCACTGAAAAAAAAAACCG
GTAAACAAGGAAAAACCATCGCGCCATATTGATATTCATTCGAAGCAGCGAATCAGCGCA
AGCGCTACTGCGAGTCACGACACCATGAGTATAGGGATAGGGGATCACTTCTGCAACTGG
CGACAAAATTAGATCATGACTAATTGTGTTAGTAGTTGAAATATGTTAACCGTGTGTCTA
AGCCTTGCCAAGCAGTGTTTTCTCTAAATAGGAGTCAAATGGAATAATACTTTTTGTCTT
TATGTGTGGGTATGTGTGTACTATTTTATCCCACTGTTTAGGTTTGTACTTGTTTTCGGT
AAAAATCGTCCGTAGTCATTGCCATATACACATGCCTAATCGGTTGTAATAGTAAACTAT
AGCAACGGCAATAGCTAAACGATAATAACGCGTGTCGATTTGATGAAAAGAAATTCTATT
CTTAGATAGAGCATGTATACTGAAGATTACCATTTCTTTGTTGCGCAGGGTATTAACGTG
CATTGTGAAGTCCAATGACTTACTGGAAGAGTTCTATCTACTTTTTTCTAGACCTTGTGT
ACTCATATAAAATATACACACGTATCTTAACCTCTATCACTATATACATACATATATATG
TATGTTCTTATTTTTCTAGTATTCTAGTCTGGGTAGTAGTTTGATCTGGTCTGGGTTTAC
CCAAGCCTTCTACCAGTCCTTGACCAGTTCTCTTATTTCTGCGTTTGTGGTGAAGAAGAT
TCGCTTTATAGCACTTCTGTTACTATTCTTCTATTGATAGAAATCGTCGTGCGTTTGCCG
CGATCGATGGAGTGTTAGAGTTGCATAGCAGATCTGTTTTCTGTCGGAGCCCAGTTAATG
GCGCTTTTGCTATGACCGTTGTCGTTTTTATACCTTTTACTTCACTTGATTCGCTTGTTT
TTTACCACAAGTGCACGTCCTCATGGACCACTTGACTAAGACTATTCCCTCACCGTTCGC
TTTTCGCCGACTTGCAACGCGCTCTCTGTTTTAAGGCAGAGGCGGCGTTCACGGCCGCTC
GACCAGCGTAAGCATCGCAGTTGTTTCTCCCAAGTAGCAGCAACAGGCTGTAACGTTTGT
ACGGTCATATACGGTAAACGTCCTTAGTTACTGTTCTCTGTTAATGTTTAGGGCAACGTC
AAGATTGTAACAGCTTTAGTCAGCAGGTGTTTCTGTTGGAGTCACCAACGGAACGGCGCT
AGTGCGCTGTTTTTGTTGTAAGGCATCGTTATCTTTCTGTTCTTTAGGCCTACATCTGTA
GCGACATCATGAAATCTCGGGGAATTGCCGGTCTAGGTATTAGCTGCGCGTTCTTTCTAG
TTGCCTGCCTATCACTATTGGCATATCTCAAGATACCATCATTTTTACAGCTGGCAGTCT
CACGGTCTGGACTGAGCCCGGGAAGTTTTGCAGCAGACCTATTTTCCGCTATTCCGTTTG
ATATCCGCCAGAATGTTTACATGTTCAATATCACTAATTCGGAGGACTTCTTTAATGGCG
CGAAGCCGCGTTTCAAACAGCTGGGACCCTATATATTCAGGATTGGCCTACGAAAGCAGA
TGACGTGGTCGGAAGGCGATTTACTTACGTTCAGAGAAAACAGGAAGTTCTGGTTTGACG
CTGATCTTTCTCATGGACATCTGAACGATACCGTGTATACGGTCGATCCGATCTACGCAA
TGGCGCAGGAGGTAATCGATGAGCTGCCAGAATTCCTGGAACGATTGTTTCGGCCGCTGT
TGAAGTCGCGGAAGGTTCTTTTCAAACACAGTGTCGACGAGCTTCTTTATAACGGGTACA
GTGATTCCCTAGCAGAGCTGGCGCATTTGATAAAGCCTAATCTTCCTGTGATCGGTGGAA
AGATTGGTTACTTACGGGCGTTCAATGACACCGATGATGGCGATATTACAGTATTTGCTG
GCACTAATCCCTTCACGAAAGATAAACGAAATTTAATCACCCAATGGAATCACAAGAAAA
CAATTCCTTATTACGGCGACGGGTGTAATGGCATCTGGGGAGCAAATGCCGAGCTCTTCC
CGTCGTTTTTGTCATCGTCACCGCCTGCGCAGATCGGCGTATTCTTGCCCCTGCTGTGCA
GGCCGTGGTCCCTACACTTCAATGAAACACGACAAGAAAATGGCATTGAGCTAGCGAGAT
TTGCCACTGGACTGGATATATTTTCTCCAAGCAACAGTAGCCAGATTGAGCAGTGAGCTT
TGACAATTCATTCACCGCCAAGATATAATTTCTAAAGAGAAAACCCTTTTCAAAGCCATC
AGAATGTGAATAAACCTGTAAAGATTAAGCAACTTAGAGCATGTTTAGAAAATATCGGTT
TCAAGGTATTAAGCAATCTCTCGTAAATGAAAACGAACATCGTCTAATCGGAGCTTAATA
GTATAATTTACATTGGATTACTTTGTTTTGGATTTGGAACATAATATGAACGCAATTACC
TGTACTGCTATTGTTGACTTTCATTTGCTATTCAGGTGCGCCCAGCCGGCAGGCTGGCCA
CGCGGTGTCTTCGACGTCCGGGCATGTCAGCATAGTTTCCCTGCACTGATTAGCTTACCG
CATTTCTTGCATAGCGATGAGAAGATTTTAGACGCCGTGGACGGACTAACGCCGAATGTC
ACAGAACACGATTTTCATCTCGACATCTTTCCGTTATTGGGAATTCCCGTTCGCCCTGCA
ATCCGCGCCCAGATAAACGTGCGAATTTGGCGCCATCTCGACACGTCTCAATCGAATAAC
CCACCAATCGTTTACCCCGTCCTCTGGCAAGAAATCGTGCTTTCAGAGTCTGCCATCTCA
TACCTTTCAACAATGATTTGGTATATCATTCTACTGCCTGAGACCACTTTGCGGGTGATT
TTAATGCTCCTTACGATCCTGTCCGTCTCCCTTGCTGTGAGCTACGCGTATACGTCCTTT
AGTAAGGTGTCTGACGAAGAAACAGAATCGATCCTAGCATCGGCTGAAGATAGTGCAGCT
TCTTCTTAATACGCACAATAGATTCTTCGGGATCAGCGTGCAGAAGCCACTCTTGTGGCC
TAAAACAGCATCTTGGAGAGTATACACAAACGAAGACGTCACTTTATCCTAAGTTAGCTG
TCGACGAGTATGCCATGCCAAACGGCCGAGCTAACACCTTCAGGGATGTAACATTGAGTG
AGATTATAGGTAGATTACATAGTTGCGAAGTGGTCGTCGATTTTTGAACTGTCCATAATT
TGTCCGAAAAAGTGACGTAATCAGCCATTATTGAATATCATCGAACTACATCGAACACAA
TAATATGTTTTTCACTAAAAACCATGTAATATTTTTAAGTTATTATCTTTAAACATATCT
GCTTTAAACAGTAATGACAGATGTGTCAAGCCGTTAATTACGAACTCACGGCGGGCTCAC
GTATGCACCTCTTAAAAAGGGCTTTTTGTTAGACGTTTTTATCGAAGAACAACCATTTGA
AGTCGAGCAAAAGTTGTACAAAGTCTATTACCTATGCTTATCTAGCCCTATGGTTGATCC
CAGTACAATTTAGAGTCACGGTAAATTTATCGTACTGAAAGCATTTAGTTTATAACTCTG
AATTATATATATATATGCTAACGCTGCGGGTGGGGGAGGGCAGCAGCTTATGCTGTTAGG
GGTACTGTGTATAGTCATTGAAGCCTTACACAGCTGGCTTGTAGTCAGCTATACCCGACA
CTGATACCGAGCTGAAAAACCTATGAACATTTCCCCATAACAGGGAATAATTTTTTAATG
AATAACAAAAGCCTGTACGAGCATTGTATTCTAAAGCTAAGCTGTAAATAAAAATCAAAT
TGTGATGTTTCAGTAAAAAAAA

>Vd20660 len=4082 path=[6516:0-270 @583@!:271-1741 8256:1742-1886 1916:1887-1995 1452:1996-2096 2888:2097-2237 1481:2238-2777 11077:2778-3021 10159:3022-3044 10182:3045-3317 9402:3318-4081]
AAGCAGCTGAAGCAGTAACATAAAGAAAAACTAAACAGCCAGAGGATCGGCCGCCTTTAG
CCTAAAATTGTACTGGCTCCATGGCCGCGACAGTCACAGATGAGCGAGCCGAGTGCCGCG
GGAGGAAATAGATGCTTACGACGACCACCACCACTGTAGTTGCGGTGTATATCGTGAACG
CCGGCGGCCTATCCTTCCTCCACTGGCCGACAGGCTGTTGTCTGTGTGTGTTGAGATTGT

CGTTCATTGGCGTCATTAACTGCGTGAAAATGAGAAAGGAACGCACATCAACCGATGCGC
AGGAGGATCGAGGGAGGCACTGAGTGAGCACAGTAATGGTGTTCGCGCTGGCAGTAAATC
TAGACAGCTGAGTGACGCTGATAAGTTACGAGATGGAACAAAACTGAGCATCAACGACGC
TATTGGCAGCAGCAGTAGTTCTAATAGTAAAAGACCGGCACAGTGCAACCGTATTCCTAA
CTGATTAAAAGTTGTCGAGGTGAAATGGGGTTTGTACCATGGACGAGCAAGAAAGCGTAT
GACAGAACACCAGCGGTGACCTCATGCTCGTTCGCTTGAAGTTTATTTATATAAGCATAC
ATAGAGAAACTTATACGTATACCGACGTTCTGCTGGAAGAACCGCTCGTTGTAGGTGGTG
GTCTGTAGGAAACTTTAGATACCCCACATTTACGAGGAACTTGTTGAAATTACAGTATAG
CCAACGTCAGTCGTAGCAGCTCCACGAACAACGTGCGTATGACAGGTGCATAATGGTTAC
GATCAGCAGCGGAAGCAGGGACCAACTTCGACATTTTCTCTCGTTTCGTATCGAAACAGA
AAGCTTTGCTCAATGTTGGCTGGCTTGTTTAGTGTCTATGAATTGATTCCTGCATGGAAA
CGAGGCGTTCGAGTCTCATACCTAGCATCACAATGTTATCATGTATGCTGCATAATCAAT
AATCATGTTAGCACCTGTAGAAGTAAGTCGCATAAAGTATGTGGAATTTATCGCTGCTTG
TGTGTCTTTCGTTCAGTTTGAGGTCAACAACTCGTTTAGCGTCAATTATTGAATTTTTAG
ACGATATGCCTCTAGATGTTACCTCAGTGTATGCCTATTCCTTCTGCTCTTCATAGCACA
GAATTTGATTTGATCTAAAATCGCGACACTAAGTGTACCCAGTAACTTATGATTTGACGA
TTGTAACTGTATAAAACAACATTGAAAGATTGGTGCCCCTGCATTGATGTCTGCGCTGTT
CCCAATCGTAAACCAATAGCGCAAACGTGGAGAAATCAACTACAGAGAGCCTGTCGTTGA
TGGAGGTCAATGAAAAAAAGCTTGAAAAATGAACTAATTGGCGTATTGTGTCATACCGAA
GAAAACGGGTTGTTCACGATGTGTAGCGGTGGTCAGCGCTTGTCGGAGCAGGAAGTTCGT
TCAGATCAGCGCTACGATTCAGCGCCGACTGGTTGCAGCCCGCTTTTGCGAGTGATTATT
TCGCTGTGTATAGGTCTTCTCTTTTGTATATTGGGCGTGGTGTCATATACTACGTTTGCA
CCCATACTTGAACAGCAAGTTAAAGCGAATTTAGTAATCGACCCCTCCAACGAGGTTTTT
GAGAGTTGGCAAGAACCCCCCATAACCATAAATGTTAAAATGTACTTATTCAACTATACG
AATCCGGAGAAAATACTGGCTGGGCTCAAGCCTACAGTAGATGAACTGGGCCCGTTTGTT
TACAGGGAACGTCGCCAGAAAGTCAATATTACATTTAATGGAAATGGTACAGTATCGTAC
CGTCAGATTGTTTCGTATGATTTCTTACCACACCTCAGCGTGGGAAACCTCGATGATGAA
CTCTTTACTCTCAATGTACCGATGATTGGCTCAGCATATAAAAATAGAAAAGCTCTTCTT
AACGAGGAGTCAATGATGGCGCACACTATAGAGGAACTCTTCGAGAAACACAATCAAACA
TTTCTGATCCAGAGGAAGGTTCGCGAGTTACTGTTTGAGGGTTACCAGGATCCGTTACTT
AGGGTGGCAAAGGAGATGGGGTGGTCGTCATCAACACGATTCGGGTACCAGGTTAACCGT
AACAATAGCGATGACGGAATTTATACAATCTACACGGGGGAAGAAGGGATGGAGAACTAC
GGCACGGTGGATAGCTGGGACGGCAAGAAAAGGGTACTCGCTTTCCGGCGATCGTGCAGC
TTCATCAATGGAACTACCGGCTTTACGATATCATCTTAGCCAACGGCTGTTTGACTATAG
CATCAAGGAGAATCAATGCTTCTGTACCAAGAAGAAGGGCAAAGATCCTGAATGCTTTCC
TAACGGTCTTCTTGATCTCAACCGATGCCAGCCAGACGCACCAATAGTAGCATCACTCCC
GCATCATCTGTATGCCGCCTCATCTGTTTCTGAAGCCGTTGATGGCCTATCGCCCGATCC
CGAGCTGCATGAGTTCTTCATGGATGTCGAGCCCACAATGGGCATTCCCCTTAGGGTATC
CGCTAGACTGCAACTTAACGTTATCGTCGATGCTTTTAAATACTTCAAGCAATTCCAAGT
ATTTAAGAAAAGAGTATTCTTGCCAACGTTTTGGACGGAAACGACGGCTATGATTAATGA
CGATATAGCATTTAAGATACGTCTTGTCACTGAGGATCTGGCCAATTATGTGACTTTTGC
CGCACTTGCTTGGGTACTCATAGGATTGATAGTCATCATATGTACGTTCGGCTACGTCAT
AGCGTACACAAGAAGATCGGTACGTCTCATACAAAAAATATACGTCGACTATAATTCCAT
ATGGGACATCAGTAATCCGGATCCGAGCTTTTTAAAGGACAGATTAGTGTTTTAGCACGG
CCACTGCTTTCTATACAAAACCGCGAAGAGAGCTCCAACACTGTATTGCCAGCTCCCTTG
AAAACAGCATTTGGTCAAAAAGAAAGACAAAGCAGCTACAATTTCAAGCTTCGCTTAAAG
ATAAACTGCCCAAGATCACACCAAGTCAAAGAGGAAAACATCGAGGGTAAGAGCAAAACC
TAGTGTTGACGAAGGCCGTAAAAAAAAGCTACAGTTTCTTTCGACTGAGCAGTCACTATG
ATTTTCGGTTTGAATGCCTTCACATGTCCTACCTTCCGGGTTGCTCTTACGTTACCCCTC
TCCACTTCCACGTACTTTGCGTATTTAAAACTAATATTTTGCCGCCTACCTTGTGAAGTT
TTTATCAAGTTCATTCCATCGATAAATGTAGCCAGGAACTCATTATTCTGTAATGTCGTT
GTTCCTACTGTTCTTCCAGAAGCAGGAGAAGCGGCCTTCGTCAAGCGGTTATGCGGCTGT
AAAGATGATTGCTACGACGCGTTTAGCTTGAAGCCTTCTTTTTTTTAAAACTGCTAAACG
GTCGCTCCCCAGCTTGTCACTCACCGAAGGCTGCGGACAAAACGAATTTACTACGCAATT
ATATTATTAGAACCTTCAAGTGGGAGACTCGTCGTGAACCTCGTTGCTCAACAATAGTCT
AGCAGAGCTTTGCCATTAGGTGTACATTGGAGAATGAATATTGACTCTGTTCAGGTTGAC
ACGAGGTTCGATGTGTAATCGTTTGTGTTGTCTCATATATGGAACGAGGTCAAGCACAAT
GAAGAACTGCATTTCCAGGTTATTCATTATATTTTAATGGCTTATTTATGCCTGAATTGG
ATGTTCACAAATGCCTCGGTATTCCAATGACGTCATGTAGAAGCTACAGATTCTGCAGAA
TTCTTTCTGCAGGACACTAGCGCATCGTTATCGTTTTACCGTTCACATGGCTACCCATTT
ACCTTAACGACGAACAAAAGTATAGATACGGCTGTTTTATAGCACGATTTTCCTCAAGCT
CGCCAGAATTCTGAAAGGATTCCCTGAGAAATTGTAGAACCAGTTAACATGGGTTTGAGT
TTTTAGATTCGCAAGTTTGCGATCAGATAAACGCAGTGACGAAAATGCGGAAACTTTTTT
CGTTAGTAAGATATAAAGAAGCTGCAGTATATACATGGTCCTATATATATATATATATAT
AT

>Vd21127 len=5041 path=[5019:0-919 5939:920-1872 @6892@!:1873-2378 24097:2379-2379 24098:2380-2381 7401:2382-2382 @7402@!:2383-3250 8270:3251-5040]
GCAGAAGCGAGGCACTCAACCGAGCACAAACGCATTTCTCAGTAAAGTAATAAAACATTA
TGTCTAGAAAGATATTTTAGCCGAAAATTGATAAGATTTCCCCTATGAATATGCTGGCAA
AGAAAACCCAAGTGGTATATCGCACATTACCTAACCCAATATCGATTTTTCCGTATCCGC
CCGTTTTTAGGTCGACAAAAAAGTACTGATATTACGTAATTGCTTTTTTATTCTGCTTAT
TTTTTCTGTATTAGACTAGAGGCGCTTATGTAGTAAACTTAGAGCTGGGTCAACTGTCAT
CAGTCATGTGAATACGACCCAAATAATATCTATTCTCGAATTTTTCGCATCCTACTTCGA

CGTACACGAACGAATTTAATCGGAACCACAACAGCACTATCAAAAAAGCAAAATCGAAAA
CAAAAAAAAAAAAGTTTTTACTAGAAATGCATGAGCTTGTTTAGCGGAAATCGATCTAC
AAACCCTCCCCCCATCCCAGCCACGATCGAATCATGTACTTATAACTACTTGAAAACAAT
CGATGTCGTTGTATATCAATACGTAGTGTCAAAAAGTTATAAACATTAGATGGTTTCATG
TGGTACCCTAGGCCTTCTATACGGATTAGGCGCGGTCTGTTTGGAGTGCACCGGAATTGT
AACGGCGTATTCTCAGCGGGACCGAGCTTTGCGAGCCCGCCACTGGGGGCGCTGCCGCTT
GTTTTCAGTCTTTCCATAGCTGATCGGAAAGGATCGCGCTGACATATCGGCGAGGAAAGC
GTATGTCGTCTCGATGGCGTTGTAGCGACGTTGCATGTGCGGCAGAAATCCGCAGAGTGA
ATGACGCAGCGCCGCTGGCCAACGGCGTGATCAAATGTGAAAGTGTCTGCACTCCTCTGT
AATTTCGTAGCCAGAAGGAGCTATGCGATAAAACGGCGAGGGGTGAACCGCCACGGCAGC
ATGGGGTTGAAGAAAAGCTACGTGGTGCTAATCTCCGCGAGTTTGTTGGTAAGTTTCCTT
GGCTGCATAGGAATCCTGACGCTTCATCCGCTCTTCCAGACTCTGTTGAGCCATCGTTTA
CCCCTTAAGCCGGGAAGTGAAAGTTATAACGGGTGGAAGAAAGTGCCATTTCCAATTTAC
CAACGATTCTATTATTTCAACGTTACCAATCCCGATGAGTTCTTAAATTTCGGTGAAAAA
CCCGTCTTGGAAGAAGTTGGACCGTATACATGGCGATCCGAATGGGTAAAAGAAGCTGTC
GAATGGCACGCAAACGGGACTCTTCAATATCGTGAAAGAAAACGTTTTTGGTTTGATCGA
GAACAATCGGCGGGCGACCAAGAAGATGTTATCTTCACAATAAACACACCACTTGTGGCG
GCTAGCCAGAAAATCCGAAACGCATCGCCGTTAATCAAATTGGCCTTTTTGATATTTTTA
AATGCAGCCAATGAAACGCTGTTTATCAGGCGTTCGATCAGACAGCTTACCTACGAAGGA
TACCCGGACGTTCTCGCCCATATTTCTCACGTGATCGACCCAAATGTCCCGGTGAAGGAT
GGCCGCTTTGCGTACATGTCGGGCAAAAATGATACCGACGAAGGCCTTTTCAATGTGTAC
ACAGGAAGCGTTGATCTCCGGTACTTCAACAGGATTGACAAGTGGAACGGAAGAACCGAG
CTGCCCTGGTGGGAATCCGGAACCCCTTGCGCTAAGCTGCTCGGCACGAATGGGGAGCTG
GTACATCCAATCGTTTCATCTGACGAACATATCTACTTTTTCAATCCTGTTTTCTGCAAG
CCGTGGAGATTAACGCGAGCGCCCGACGTCGATAGTCTAGGAATTACCCTCACACGATTT
GTTGCAGGACCCGAGGTGCTATTCAACTCATCACGAGAACCGGCTAATCGCTGCTTCGAA
ACACCGGGTAAATCTCTACCGTCCGGCGGAATGGATCTCTCGCGCTGTCAATTTGGCCTT
CCACTCGTGCTCTCCTACCCTCACTTTTATGCCGCAGATTCGAAGTACCTAGAAAAGGTT
GAAGGCTTAAGCCCGAACCAAGAACGGCACCAGTTTCAAATCGATATAGAACCGCGTCTG
GGCATAACGTTAGGGCTGTCTGCGAGAGCTCAGATCAACGTCAAATTGGAGCGCGTCGAC
TTCCTGAAATATTTCCGTTCTGTTCCAGAACTAGTGTTCCCTGTTTTCTGGCAGGATGTT
GTCATCGAGCAGACCCCGGCGTTTGCCGACCACATTCGGGTCCTACTGGATAGGCCTTTG
TTCTATGCGCAATTTGCTTTCCGGTTCATGACGATGTGCGGGTCATTGGTTGCTCTGGGC
GCATACATTTATGTGGTCATTCTGGCTAAAAAGGAATCCAAGAAAGACCGATTCGGTCCT
CTTTTGGACGACGAAGAAATCAATGAAGATGAACACGGAGAAGGTGACGTCGCAGAAGAC
TCCGCTTCGGGACAAAGTCAAGGACATTGATAAAGTCAGCAGAGAAAGGAGTCTGAAGGA
TAGCAACAACCAGACAAAAGCGATAGCCCCCATTTCGCCGTCAGCGCCTCCGTTGAATAA
TCAAAAAATACTAGAGTCAGCCTTAGCTGGAGGCCCCCCGAGGTCGGCGAGTGCGTCATC
GACTGATGTTGACCAACAATACGCCAAGGACAATGCGCGAACGTGGCATGTAAACATGTT
CAGTGCGAATCATGAGAACAAAAGGACCTGTAATATCATCATTCCCAGATAAACTTTCGG
TAGAACGGCAATCCGGTAACAAAGCGATTTCTGAAAGCTGGTTTTTCACGGCACAACTGC
ACAAAACGGCTGATTAGCATATAATGTTATGCTGCTTAGGTTATATGGTTAGATATAATT
CTTGATCAGCGCGAACAAAAACCGGTTTCAGCTTTCTACATGTTGCTTCCCTTTAACACT
AAATCAATGCCGTAACACAGTTCGTAAGCTGGTATACATAAAGGAAAGAAAGGTTATGCT
AACCTTTCAGGCTCCCTACTATTGTGTAGCCGTTGCGCCGTGCGCTTGTCATAGCTAAGA
AGAATGTTTGTGATCGTAGCACTCCTTTGTGAATCTGTGATGCTAGTGGCTTGTAGATGG
CGTAGGTACACGAGGTCTGATTATAGGATTCTAGATGTACGGGTTACAAAAACATTAAAT
ATCTATTTGATGATGTATGTACGTACATACACATACTCAGTCGTAGCCTATGGCGAGAAT
GATCGACCTACTTTTTTGCCACCGGCATCCACTAATGAACGCATATAGCTAGCTATTCAG
ACTCGAACACGAGATACCTCTGCTCGGGAGCAGATCTGACCCGATTTAGATTGTCAGCTT
CTCTATACGTTGTAAGTATGCTTGCTTGCTTGCTTGCTTGCTTGTGCTGTGAATAGCAAA
TAATACTCTACTGAGACGTACATTTGTTTTTTGAAACTGTCTAGAAATTCAGTTGTCTAG
GTTTTTCCTTCGGAAATCTCATTTTAGGCTCGCTGTTGCCGTCTGCGTTTCATTACGCAG
TACTAATTATCGTACTAACAAACTAACATAATGAATTGATAGTATATAAAAAAAAAACCAC
GATAACTGTTAAGGCCGACCGTATTTTGATACCAAATATAAACAACGTTCGCTTTACTTT
GTGCGTAAACAATATAGCAGTGGTTCCTGAGGTCAAATTGTAGATAGTATTTAACGTTGT
AGATTCGCTCTCGAGATGTGCCATTGAAAATTGTGTCGAGTTATTGATGCATGTTTTTCC
TCCTTTTTTTAATTTTTGCTTTAATTGTTTTCTTCCTGCGCCTGACGGAAGTGATTTGCG
CCAACGCTCCCCGTCGAAAATTCCCGCCCGTATATATGCATGGCTATCTCATGTATCCGG
TGTGAATACGGCGGGTTCAGAGTACTAACTGGATGTTTTCAGCAGACAGCTAATTCGGCA
CAAAACGAGGTGATTTAGGTGTCGAAGAGACTAAAGTCTGTAGTTGAACCACTTCATTGT
TTAGCTATTCCGCTAGCTAGTTAGCGTGAAGTCATCACTTTTATATAGAAACAGCTCAAA
TTCCGTCACTGCACATTGAATACACGTCCTTGTTACATCAACGGTTTTATATATGAGCGT
GTAATAAACATTAAAGATGTTTGTATTGTTCCATAAACAACGCTGTTCTCATTCATGTCT
CAGTAGGTGACAGAAATATTTAAAGCTTTCAGGCTTTTGGCCGACGCCGGGCCTAAAGGG
CCTCATCGTTTCCGATGCCATAATCCTCCCTGCACGCTATTAAGGTGCGCACACTCGGCC
TATTAGCTAGGCATTTGTAACAATGCCTTGAGGCTTAGGTAAACGATGTCTTGTTTCCCT
GAGCTAATTCTGTGTTATGTTTGAGCATTGGTGCACTGCGATTTCTGGGTAGCTGGTGAA
GATCTATTGAGGATCGCTCGAAATGATGTCCTAACGGGAAAATCTACTCGACCTTAGCCT
ATACATGCAACAAAGGTGAAGAGTCTAAGCGAATATGTAGGAGGACGTTGACATAGCTGT
GGTCTGAATTGAAACTGTAAGCGTTGCCCACTGTTATGTAGGCATAGTTTCAGACAGTTT
GCGTCGCGATACCGGCGGATCCTTTTCTTTTTTCATTTGTAACTGACCCAATGTTCTGCA
ATACGTACTTAGCAGTTGGGCATTTTGCTAACAGTTTCATGTTGGGTTCAGCTAGGGAAC
CAAACAAGAAAGTGGTTGCTCTAGCAAATTACGGTGACATCGACCGTTGAAGCTCCGTTC

CTATTGACGAATATAACTGTAATGACTGATTGCATTTTTTTCAAAATTAGGACGGTATTA
CGGCACGTTTTATAAAGTGAGCGTCCTGGTATTCTTGAATATTAGAAGGTCAAGTGCATG
CTGCCGTTGATGGTTACTTTTCATTTCACAGTGCCTATTACAGTTCCTTTGCATGAGTCA
AACTTATACTTTTCTACTATATAGAATTCCGTGTCTCTTTTTTCTTTTTTTTTTTTTTTA
A

>Vd19882 len=3914 path=[6636:0-1196 480:1197-1406 3545:1407-1664 9520:1665-1666 738:1667-1667 805:1668-2070 854:2071-3268 2052:3269-3913]
CAAATATTGACCGACGCGCGTTTGTGAGAGTCAGTGGTTCGTGTATCAATGGGAAAGAAA
CAAGCCGACGGCGACGCGTGTGCCAAGTGGAAACGCAGTGTTAATCTTGGCTTCTGGTTA
GAGCCGTTGTCAATTGTTGAGGGTGCACGGGCCCAGTAGTAACGATGGGAGAGTTGTAGG
GTGCGCACTCGCATGTCGAGTCCCATCTCGCAACTGAAAGGCCGTGAGAGAATTACTGCT
TGTACCAGCAACGGTTAGAATTATGTCGGCAAACATCGCACCCAAAAACAGAGAACTCGG
ACGCGACAGAGCCGTACTCCAGAGGTTTCATATGTGGTGCGGAAACCAGCTATATGGCTT
TCATTGATTCTACCTTCTCACATAACAGCACTCTATAGGGGAAAGTAGCCCCGAGAGTGT
CCCCACTTGAAAAATACGAAAGCCAGTCAGATAAACGGACATTACGTTATTACAGCATTT
ATGAAATGGCACTGGTATCGCTGCAGACGCCGCTGGATTGAATAAGTGCCCTGGACCTGT
AAAACATTTCACCGTAATCGGTGTGCTTCCAGACTCAACGATGCACCAAAGACCACAGAA
AAGATCACGTTCCTTCTGTGTTATGGAATGCACCTTAGAGTGATAGTAAATCCACTGGAT
AGATTGATCAGGCGCAATTAAGTCGGAAAACGGATACTTCCTTTCAAAATGTGTGGCGGT
AGTTTCTGTCAAAGCGAGCTGAACAAGCCAGGAGGTCATCGCTCTGAAGCTTCAGTCGGC
AGGTGTACACCACATCTGGCAGTGACCTGTAGTCTATGTATTGGACTATTTTTCTGCGTC
TTTGGAATGGTGTCCTATGCTGCCTTTGGACCAATTTTAAGGCAGCAAGTCAAAGCGAAT
TTGATAATTGACCCATCAAATGAAGTTTACGAAAATTGGAAAGAAACGCCCGTACCGATT
TACGTCAGCATGTTTCTGTTCAACTACACAAACCCTGATGACATTATTCTCGGTGCTAAA
CCAAAACTGCAACAATTAGGTCCGTTTGTTTACAGAGAACGTCGCCAAAAGGTCAATATC
ACCTTCAACGGAAACGGTACTGTATCTTATCGGCAACTATTGTCGTATGAGCATTTACCG
GAGCTAAGTGCAAGCAGCCTTGACGTCAAGTTGTACACGCTCAACGTCCCAATGATCGGA
GCAGCTTACAAGAATAGAAAAACGCCTTCAAACGAAGAACAGCCTATGGCTTCTGCACTT
GAGGAAATGTTCAGTAAAATGAATCAGTCTCTCTTAATTCACAGGACGGTACGCGAACTT
CTGTTTGACGGTTACGAAGACCAGATGCTCAAGCTTGCCAAGCAATGGAGCTGGTCTCCA
ACAACGCGTTTTGGGTACCAGATTGATCGTAACAATAGCAACGATGGGATCTACACCGTA
TTTACGGGAGAAAATGGAATGGCCAATTACGGCACAATAGAAAGCTGGCAGGGAAGACAC
CGTGTAATAGGCTTTCGTAAATCATGTAGCTTTATCAATGGCACAACTGGCGAAATGTGG
CCACCGTACACGCTCACGTCAAAGAGTTCGCTTCTATTTTTTACCTCGCCTCTCTGTCGG
TCTCTTCGGCTGGATTTTCTCCGCAACGAAGTTGTCAAAGGGATCCAGATGCACCAATCG
TGGTATCCCTTCCTCATCTACTCTATGCCTCCCCGTCCATTACTGAGGCAGTAGAAGGCC
TTTCTCCAGATCCGCAGTTACATGAATTTTTTATGGACGTCGAGCCGTCCATGGGAATTC
CTTTAAGAGTGTCTGCTAAAGTACAAATGAATGTCATTGTTGATGCCTTCAAATACTTCA
AATACTTCGAAATGTTCGAGACAAGGAAATTCCTGCCGACATTCTGGATCGAAACTGCCG
CCGTAGTCAACGATGATTTTGCCTTTAAGATCCGTCTCGTTGTCCAGGATCTAGGTAGTT
ACGTGAGCTTCGCTTCATATGCCTGGGTGCTTATTGGACTATTCATCATCATGTTTACTC
TTGGCTACGTTATAGCGCATACGCGAAGATCGGTACGTCGTAAGTACATGACCAAAGTCC
GGCCTGACGTTGCAAAAGGCACAGAAAGGCCGGGCAAGAGCCTTGATACATATCGCGTTT
TCAGAATCACTGATAGTTCAGGCACAAATTCTGGAAGTAGGGCATGTTATGATAAAAACA
ACTGCACGATCACTAAGCGTATGGTCAAAAGATCAGCGATATGTAGAGGCACTCAAATCA
AAGTAGATAGCACTGAGCAGAGCGATAACATGAACGCGGACGAGTTAAAAAATGAATTTA
AAAGTGACAGTAATCTAAAGCTAGAAGAAGAGGATGAATATAGATTCGGCAATGCCTCGC
TAATACAAAGCCCGGAGGATAAATCGGAAAATGTGAATGATCAAAATACAAGCGTTCCTA
AATCAGTACTTTCGGGCAGTCGGGAAAACCCAATCATTGATAACAACTATAAAATCCTAG
TATCTACTGAACAGGGTGGCTATCTAAACGTCGCTTTTGAAGAGGAAACATTACCATACA
GTGCAAAAACTGAAATAATAAGCACTCTGTAAATATAGTCAATCGAATACGAACCTTGTC
TCTTAGAGAAGACACCAAAATTAAAGTAAGTACTTATCACTGAAAACTTGGCCATTTTAC
GCATCCCATCTCGTACTTATATCGACAGCTTTATATAGCAAACCTTTGGAGGTGTTCATA
TGAATTAACCCGTACTTCCGAAAAAATAAATAGCTAATGACATTAAACAATGAAGAAAAT
ATGTGCTATCCTTTCGTGACATGTCAGTGCATTTGGGGTCAGAGTGTTTTGCAATACAAG
TTTCTGGGTTCGTGTTTCCTATAGCCGACGAGGGCAACGTTTGAACGCTGTCTTATTACA
ACAACACAAGCATGACTAACCATCTTCCTACTTATCTGTCCAAAATACATACGTAGTTAA
TGCAATAATATGTTGTTGAAATTTTTAAACTGATTCAGGTCCGAACAAACATTTTACCTG
GCAATCTCTTTTATATTGTATTCATCCTTTTAATTTGGCTCTCTTTAACATCTTTTTTGT
TTCATAGATCCTATAGATACACTAATTACTTTTAGCTGCACTTCATTCCTCTGTAGTATG
GTTAATCAATCTATCTGTATGCCCTTAACACTTGAGCTTTTTTCTGTCCTTTGGTCCTGT
GCGATTGTTCATTGTTATTATTGATTTCAGAGAATGGGACAACCTTCGCCTTACGGCTAC
AGTACTATCAAAATGGCCGAAGTCGTTACTGGTCGTTGAATTTCTTTATCGAAAGGCAGA
CTATACGCTTCCTGATGATTTCTTTCGGCGTCCAAAAGCATCGGCAAGAGTAATAGACGC
GTTTCGCGCATAGGTCTTTTAACATTCTCGCGCGAGCAACTAGACTAAAGTCATTGACAT
TCCGACGAACCATAACCATGTCCTTTCATGTGCCGCTAAGGAAAAAGTTCAGCGGGAAAA
AGGATCGTTTGTGAACGTCTCGGGGGGTTGTCGACTAATAGTTTTTCGTTGAGAAAAATA
GCAAAATGCTATTGGCACCGAGAGATTACCGAATTTTTAGCTAGTGACACAAGTTTCATA
CTCATTTTATACGTTCTTATCTAGCAGAAGCTAATGTATAAATGCTCAATATTGTCAAGG
TGAGTAGGTATTGTAAATAGAAAATATTAAAATGAGTAGTATTCCTACTATTGCACAGTT
TGTTATGCCACAAAATAATCCGTGTTCCCAACTTGACATTTATACAATGAGCCGATTTTA
CTGACGCCTAAGTATTGTAGCAGGAATGACTGCTTGGCAATAAAAGGATGTTTCTGAGAT
TTCCATTCAAAAAA

>Vd22104 len=4577 path=[7720:0-1169 @10178@!:1170-1764 10770:1765-2376 11382:2377-2630 11636:2631-2631 11637:2632-2783 11789:2784-2901 11907:2902-3563 12564:3564-3733 29315:3734-3975 12732:3976-4450 30229:4451-4451 30230:4452-4454 13210:4455-4516 2065:4517-4535 2084:4536-4541 2030:4542-4561 30266:4562-4569 2058:4570-4576]
GTTTGTTTGAACTCAGCGATTCCGGTAGCTTTGGCGGCGTTTGAATTCGTTTAGATAACG
TTGTGTTTGTGATGGACAGCTATTATCACGTAGAGCATAAGTGTGATGTTTCGAAGTATC
GCCGGGCTAGCGAGACCGACGTACGTCTAGGGGAGCAACATAATGTCTATGCAGATACCG
TTAGGTGCGTTGTATTTTAGCCATTTCATTCTGGTATATGGAGACATCCTGGACAATATG
GCTTAAATGGAAAAGTGACCTAGGTGCATTGAACCAACCGACTGAATATAAATGCATTGT
GTAACAGTGCAGATTTCTTGAAGCAGTATCTAAAGCTTCGATCAGGTGTTTGCTGCATTG
AATTCGATATTCTGACAGAAATGTACGCCTGAAGCTCAATGTATAGTTTTAGGTGTGTAA
GCTTATTAAAAACGTGCCCTTCATCTGTATCACAACAGCTGGCGCCATTACTTGAATCAC
TCCTGTCTTTCAAAAACGAACATAGCAATAACCGTCGCAACTAAAGCAACGTCGTACGCT
TTAACTGACACAATAAGATCGATAATGGATTGATCCGATTGCGTCAGCTAGAATTAGTCG
ACAATAATTTACGATGCTATTATACACCGAGAGTCTGTTAGACTCTTTTTTTAATCGTTC
AAGACTTAATGAACTTCCTGCACATTCCGTTCGTATGAGTAGCTGTCGTGGGTTTTCTCG
TCCCGATCGGAATAGTTAGTTTGACCTATGCCATCTACCAGGAAATGACCGACGTAGCTG
AATCTATGGATGAAGATTCCAAAGCGTACGATGCATACGTAAATTCTCCAGTGCCTACTT
ATCAATCGATTTATATGTTCAACCTGACGAATTACGAGGATATACTAAGAGGCAAACGGC
CCATCGTAACCGAGCTCGGCCCCTACGTATTCAAAACAGTGACACAGCGAAATAGCTCCT
GGTACCATGACCTGGTTGAAACAACTGAAGTAAACGCTTATTATTTCGAACCGAATATGT
CTAAGGGAACTCTGGACGATACAATTTTTACCATCGACCTTTATGCCATTACGCTGTTGG
ATATGCTGCAAGAGAAGCCGCACTTAAAAAACATTTCAGGCAATATGACGGTGTTTTTCA
ATATGACAGTTCGGAAGCTGCTCTACGAAGGCTATGAGGAGAACAACGAGTGGGCGGACA
TCTTGCGATTTCCCGGCAGCCTCGTCTCTTATCTCCGACCTAATAATGGAAGCATCACAA
AATATCAAGTATATACAGGTCGAAGTGGACGTTCCAAAGACACCAACGCCTATTATTCAT
GGCAAAATAGCAAAACCCTCAACTACTTTAAGCCTGCTTGCAGTAACTTAGACGGCACAA
ATGGCGAATTCTATCAACCCACACTGTATCCACCATTAATCGTCAAGATCTTCCGCCGAT
CATTGTGCAAACCGTGGAGATTATATCTAAAGGATCGCTTTTTACGTTTAGGGGAGCCTA
TCGTCCGTTATAAGGCACTTGCAGATCTGTTCAGTGAAACAGGCGATCCGGCTTTGGATA
GCTGTTATAGTCCCAATCCGACGCATAGACAAGGAACTTTCGATGCAACCTTGTGTTATA
TGCAGGACCATAACCTCACAAGGTACGCAAGCCAGGTGATACTGAGCTCCCCGCACTTTT
TAAATGGAGATCCAACACTCACTCAGCAGATCAGCGGGCTGGCCCCAAATGAGTACGAGC
ATGGCTTTTATATCGATGTTCATGCGGTAAGTCAGCATAGTCTGTCACTGTCTCAAGTGC
AACAGTGTTTCCTTATCCGGCCGATTACAGGTAATTTTATAGGAGATTTGCACTCGTCTG
ATTATTTTGATTAATGCAGAAACAAACTCTCGACTTTGTCTTCTAAAAAAAGAACGATAC
GAAGAGTTCTTATTGTTTCGAGTAGTCAAACAAGTACGCCAAAGGATCACGTTTTTGTCT
TATACTTATTCTGTTTATTGGAAATCCACTGTTCTGCCTTTTTCGCGACCAAGCGGTAAC
TTTTTCCAAGAAGTCGATAGTTTCGATAAGTTTAAGAAGAGCTGTACTCAAACAAATCCC
GAATTTCTCTAGTGTGGGTCATTTCTACGCGTTCGGGTTGCAGACTTTAAAATCTGCCAA
AAAAACAGATAGGCTCAGTGAACGATCGTCCAGCATTAATTTACATTTAATAAAAAGCGA
ATAACCTGTATCGTTTCGCATAAGCGCAGCAATTTTAACCCGGATTTGGGGGTCTATTTT
TGTCGCTATGAGAACAGGGAGTGGCTAAAATGCCAAAAATTATGTGTTCAATTGTCCAGT
TGTGTATTCAACAACAAATATGGCTGCTTTTATAAATTTATTCTTAGTGGAATGTTTTGT
CGTGGATCGAGATCTTAGTAGGTCTAATTTAGTATGTCAAAATACCATTACACTGTGCCT
GTCGATATTGAAGTCAACTTGCAGGATACGACAATAGGTCCTCATCTGGACAATTAATGC
TAAGAGATCTTGACTACAAATGCTGAATTTACAACTCATCGAACGTTCCTTTACTACTTG
AATTTAAACGAATGCCATACGGCGGCATTGCATTTATCGTATACGTTTAGGTTTTTTTGA
AATCTAGGCTTACCTATTGTGTCTAAATGGCTTATATGATCAACAGCCAGCTAAGCCGAT
TAGGTCAAGGCTTCTTGGTAACGTATCGGTGTATATTGTGCATCGTTAAAATACGATGTT
GCTTCTAATGTAAAATCTGTTTAGAAGAGCGGATTCGTAGTTGACATGCGACGTCGAGT
ACAGTACAATTTTAGGCTTTTGCCGACGCTGCAGGAGCCCCATCTGCCACCGGTTATATA
TCCCGTTTACTGGGAGCAAATAGTATGTATCTAGCCACTAATCATTATATGACATTATGG
ATGGAGTAAGCCTATTTTCTACGTCTTTTTTGTCGCCTTGTATGTCGCTCACTTTCTCAC
ACGAGGCAAATTAAAGCCCAGGAGAATGTTGAAGGTTCATTGAAAGAAGCAACAGTGACC
ATTTTCACTATTGAACTCTTTCGACGGAAGCCTTAGAGATGGACTAACCCCCTTTTTTAA
TTTAAGTAGTACACATAATGCTAGCAATAGTCATAAAACCTATAATAATAGGGGGTCAGA
TTGGATTTTTAAAACTATGATATCGTTTTAATAATACAGTTTAGAAATTCTGTATGACCC
TTAACCTCCTAATTTTGATAACACTTCATCCAAAAGCGTAGACCCAGCAAAAGGAGCTTC
TATATCAGCTGTATACAACTATAGACGAAAGCCATAAATTGGCAGTTTGATTTAAAAGCT
TGCTGTTGGAGGTTACTTAGGGTGAAATTTCCGAGAAGGATTAGCGAAACCTCCAACGTT
CCCCATCTTTTGATAGCATCTGACATCTTGCTAGTCAAAGTTAATCTCAGAGTTCACGAT
GGTTTACAATTAAGTTACATTTTTGCATACTTAATAGCACTTACTCAAGTTTATAGATGT
TCTACCAGGTCTTTCGTATTTTAGTGGCGCCTGCCTAAAGACCGTGACCATATAATCGAC
TACCTGAAACGAGCCTGGTTTCCAATAGGATGGATCGCTGTTACGGCGTTTCTGTTTTTT
GCTTTTGCCCTAATTAATCTCGTAATCGCTGTGGCATGCTTTGCAAGAGCTCCTCATGAT
CCAGTTCAAGAAACGGTAAGCACAACGAATGTGAGGCATTTCGTGGCAATTCCTAATAGT
GATTCCCATCGCGACAAGCCGTAAGCGTATTTAGAGACGGTTAATGCTGGCTGATTTTGA
TACGGGCGAAAGTCAGTTTAGCGTCATATAACAAACCGGGCTATGTAATATTTTTGCAAA
TGTGGGTGTTGGTGAGTCCTAATAGTTGTTAACATGTGCTTTTTTTTTTGTTCTTATAAC
CATATCTAAATTACTAGGTAAGCCCATCCACTAAGCAGTTGCTTGGGAAAGGCGTGAGTG
AACAAGTGCTTTTGACGGAACCTGTGTCCTCAATGACAAGCAGATTTTTGTCAAAGTCAA
CTCAAGAAACCTCTCCCAAAGAAATTGAAAGCCTTAAAAAAGGCTTGAAGTCGACACTGT
TTGATGAGGGCTCCGTCCTCGAAGCAATAGACTCTGATGGGTTTTCCAACTCGCAAACTA

AAAGTGGGAAACATGGCGAAGATGATGATCCGGAAACTCAAACGGACAGCTCGGCTGGAT
TACGTCCCTTCGTGGAAATACAACCTAAGCCGATTTTGCGATCGAGACCTGCAGCCGCCA
ATCCGAAACCCAAGTTTGTCATACCACCGAATACTTCCAAGAATCCGTATGGTCCACCCC
CGTATCCTACGCAGACATATCCCCCGTCCCTGTATCCACAGGGATATCAACAGTCTTCAA
ACCCAGCTCAGAGGTATGCCCAATCCCAGAACCAGTCTCAGACACAGCAACAAACCTCAA
ACCCAGCTCAGCTAGACACCCAGTCCTCAAACCAATCTCAGGGGCAGCAACAATCCTCAA
ACCCAGCTCAGATAGAT

>Vd21844 len=3819 path=[48262:0-758 80329:759-760 @49021@!:761-1250 @49511@!:1251-1874 57125:1875-2218 50135:2219-2441 78982:2442-2820 50358:2821-3009 80790:3010-3010 50548:3011-3349 50887:3350-3818]
CGGCGGCGGCGGCGGCAACAACAAAAACCAAATCGTTGCCATTGTAATCGACTACCGATC
TACGAGGATGCGCAATAGCCCTCTCCTTTACTCCCGAGCGCGGAGAGATCTAAGTCTCTC
AGGTGTTGCAGCGACTCCTCACGCTCAATGCTGAAGCAGGAAGAGTCTTAACGGGAAGAA
CGACGGCAAAAGTTCTTGCCGATAACAGCAGTAACATTAACACCATCACTACGACAATTG
CTCGTAGCAGCTTCAATAGATAGAACTGGACTGCTGACTCCTAGAGCACACTATCGGGAC
CGGCAGGACCGACCACGACGACAGTCGCTATTCGATTATTACTGCTGTTCTCTCTTTCTC
TTTCTTTCTCTGTCTCTCGTGAACTTCATCGCTTGAACGAACCGAGCTTTTTTGTGTAGG
CAACCCTTAACTAGTGACTGCGCTTTGAGTGGATCGTGTGCAGATGCAGCTACCCAATTT
CTTCTGCGGTTTTTGCGTTCGTAAGTGCGCGCTATCATTCACCAGGCTAGCCGATGGTAA
TCTTCTTCTTAAGGATCTTATTTCTTCCTCTGGACGGAAAACTGTCAACGAGTGATAAAT
AATCGATCTATCAGTCTTTCTGTTTGTCTGATTGATCGATTGATTCTCTTCGCAAGAAAC
TTTAGAGAACAACGAAGCAATCGTAAAATGCCTCGTCACGTCCTAACTATTTCGCCGGTG
AAGACGGTCAGTGCAGAGATAGAGCGCGATCCCCTCGAGAGATACAATGCGATGACGGCA
GATCTTCAGAATGGAAAGCAAAAAGAAAAGACGAAAACCCCGAGCAAATGCTGTTGTTGC
AGCTACAAAGTCTGTTCTTTGCTGCTTTTCATACTGTCATTCATTATGACAGTGACAGCC
GTCGTTCTTCAGAGCCAGTTCGATGCGTTCTTTGATCGTGCCCTTAAGGACCAGATGCAT
CTCGGTCCTAGCGGTCTCGTGTTTCCGGCCTGGAGAGTATCCGATCTGAGGATGCGTATG
CGCGTATACTTCTTCAACATCACGAATCCGGTTGAGGTCCAATTGGGCGACAAGCCAATT
CTCAATGAGTTGGGACCCTACACATGGCGGATCTATATGGAGAAATTTGATATCAAGTTC
CATGAAAATAGTACGCTGAGCTATAGGGAAAAAAAGTGGTACCAGTTCCTTCGAGAGGAG
AGTATAGGCGGCTATAATGATATAATAACTACCGTTAATGTACCCTATGCCGCTGTTGCA
CAACGGCTTAAGGACGGTGGAACAGTTGCTAAAAGTACAGCTACCTTTACTTTGAATGGA
CTCGGGCAGACGGTAATGCATGCAAAGCAAGTTGGTGAACTCACGTTTGAAGGATATCCG
GACTTCCTTATCCTGGTGGCAAACGCTGTCGAAGCCGGCAAAAAAGAGGATTCAGGTTTT
GGAACCATTTTGGGTACTGGTCTTCGCATCTTCACCAACGTGCTAGCAGCCGAACCTGAC
GTACAGGGCAGTTTTGGCTATTTCGTTGATCGAAACAATACGGATGACGGCATAGTGACG
ATCTTCACCGGAGAGGATAACCGTCAGAAGATTAATCGTGTCAACGTCGTTAACGGGCAC
ACAGAACTCACAACATGGCCGCAGCACAAATGCAACAAAATAAAAGGCACTATGGGTCAT
CTTCGGCCACCGATGAGTTCGCGCGAGAATCCGCTTCTCATCTTTGTTCCCGATATTTGT
CGTTCGTTACCTCTGCGTTACGAGAACGTGAGCGTGTACGAAGGCCTTTCTACCTTGCGC
TTCGTCGCGGGCAGAGAGACCTTCAATAATTCCGGTCCCGATCCATGTTTCGCTGGTTCG
AGGAAATTCAAAAGCGGTAATACGCCCAAGAATAGTATAAATGCGGTGTCTTTACGACAC
ATCATTGCATTCCTTGACGTACGTGCATAGATATTGGTACCTTTTCGTTTTCAACTAAAG
TCAACCGCTTTAGCGCTAATATTACGCTTTATTTAGTAAGCGATTATAATTAGAGTATCT
ATTTAGTGGCTCAGAAAATAACTCCTGAATTGTATATATCTTTGAGCTGTTCGTACTACT
TTATTAGTATAACATTTGCATGGGTTGTCGAGTAGTTTAAATAAGAATCTACATTTGTGC
GCATGTGCTTCACTAGTAGTCGAGTTAACAACCCAGGACTATTAACTGCTATGCTTCCAG
GTGTGATGGACATTGGACCTTGCAAAAAGGGCGCTCCGCTCGTCATTTCATTTCCGCATT
ATTTGTTTGCTGATGAGGCCCATCAGAATTCCGTGATCGGCCTAAATCCACGAAAGGAAG
CTCATCAGTTCTATCTCGAAAGCGACCCCTTGACCGGTGTTACGGTTTCGGTAAGAGCGC
GATTTCAGGTGGGAGTCGTCATGGAGCGCGTACATGGCCTAGGGTACGTAGTTGATTACG
AGTTGATCGATAGCCAATAAAATAGACCTTTTGCTAGTCAGTCTCCAGCATTCGTAGTTT
AAGAAATTAGATGAATTGTGTTTTTTCATTAAATAATATGGAAACGAATTAGTTGATTCA
TCTGTTTAGAATTACTTGAAATATGTAAAAGAAACCGAGTTTTTGCAGCACCTGAAACTA
GTCATTGACCGGTGCTTGTAGCCGGTTCGGATGTGCCCATTGGGCATAGGGTATGACTGC
TTTACTCATATAGTTATCTCCAACTGGCACATGTTTGACTTAGCTGACGTTATGGAGATT
AGACTCTTTTTTTAATATAGAGAGTCGTTTCTTTTTGCCTTGCTGACGGGTTACGGTTTC
AGAAATCTCGGAACAATATTCGACGGCACCATCCCATTGTTTTGGCAAGAGCTCCAACTA
GAAGCACTTCCACCAACTGTCTTTCAGCTCAGCATTCTTCGACAACTTCCTATTTATGCT
AAACAGACCGCCTTGGTACTCATCGGTACGGCTATCCTCATCAGTTTGATTGCTGTCTTT
CTCGTTTATCGGTACGTTCATTCTACATACGTACTTTAGTTTGCCACGTTTCGGTTGCCC
TATAACGTTATTTTGCAAAATACGTACAAAGTGTATGTTTGAAGGTTGAACGATGCTTGT
GTTTTTTTTTTGGTGTACTATTCATTTTAGTTGCTAGCAGTCGTGTCTATCTAGAGCGAA
GCTTTGTGAGCCTCACTCTGTAATGCTCTATTATCATGGTTAATTCAGTCAAAGTTATAC
GCTTCATTGATCAGTTTCCTTTTTCTAGTTTACGTACAATTGAACGGTAAAATGCTATCT
GTGTTATGTAATAGTACTTAACCAAAATTAATCATATGTAAAATCTCTCAGTGTACTTAA
AGGCACATTACTCCATTAGCACCGAAAACCTATCCACTGATATCGCTGCACGCGTCGACA
GCAGTAACGGTGGTCAGCTTTTCCCCAGACGAAAATAACTCATCAAATTTCCCCCTACTT
AGCTTAAGCTTAGTGGTTTTCGATAAGCTAAATGGCTGTTACCATCAATTCCGTCCAAAA
GGCTCCAACACAGAGGCCATATTCCAAGATAATAAGTAAATAAAGCTGTAGGTACACATA
GTGAGATACAGGTGTCCGACAAACGGTGGCATCTGGCTCGCAAGGCGCTACTTTTTCTAA
CGCAAGCGTGACACGAGCAAAGAAAAGAAATTAAAGGAACATTTTATAATAACAAATAAA
TGATCTACTTGAATACTGAAGCGTGCAGTGGGAAAATTGCTCAAAATCTAACAATGCCTT
ACTCAGAATAATTGAGCCATATATATATATATATATATATA

>Vd19004 len=11392 path=[21612:0-1262 8169:1263-5078 11985:5079-5191 12098:5192-5192 12099:5193-9383 @16290@!:9384-10102 23592:10103-10152 @17320@!:10153-10831 24318:10832-11216 24703:11217-11391]
ATCATCATCATCATCATCGCCATCGCCATCGCCATTATTGTGATCATCGCGTTTTCGCTG
ATAAAGGTTGTTATGGCTCTACTGTGGCATCTTCATCTCTGTTTGTGAACGTGTGCTGTT
AATGAATGCACTCAGATTGATGTCGCCTCCGTAGGCCTGCATCAGCAGGCGTCATCTGTC
CTCAGCTAACATCGCCTTCTTGATCAGTGCGCGCGACAGGAGATATTCATCGGATATGCG
TATAGGGCAGTTGTGACACATATTTGTATGTGTATGCACTGACAGTGATCTGTGACCCGT
GTCGGATGTTGGTGACGTCGTCCGAAACTGGATATAAATGTTCGGAGCGGCGAGCTAACA
TGTGAGTCGAGACATTCTCAAGGCATGAGTTTTGTGGGGGATGTCGACCTTCTGACCGAG
TGCTGGGGGCACTGCTTCGAATCCTGCTTGTGGTGATGGCGCAGTGAAGCTCATATCGTA
ACAGAATGCAAGCAGAAAGGAAAGCTGAAGTACAGCAACTAACATAATGGGAGCACCTGT
ATTGTGAGAGCAGTCGGGAACGGGTTTGCCAGCGAATCTAGCGTACTAGACGAAAGAGAA
TAACTCGGTAGGTGCAGCGGTCAGACTGTTCGTGCTTCGATATACCAGCGTGTGGAAACG
ATACAAAATAAGTGCTTTGTGGACCATTCTTCAAGGGAAGAATTTTCTTGGACAGTGTTG
ATTCGTGTACAGAATACGATGATTTTAACGGGTGTACCACGATGGGTAGCTTTCATAGTA
TTTGCAGCTTCTGCCGTAGTTTTCATTTTGGGAGTGGTCGGATACTATATGGTGCCCTCG
ATCATTGGCGCAAAAGTCAGCCAGCAAATGCGCCTTGTCGAAAACGGTTCAACATTAAAA
CGATGGGCAAACGTGGCTGTTCCTATCTATTTTTCGGCATTTATGTTTAATATAACTAAT
CCGGAGGAGTTTGCGAATGGAGAAAAGCCTCAAGTGCAAGAGATCGGACCGTACGTTTAC
CTTCAGAAGCGACGCAAACTCATCACACACATCGACAGGGACGTTGTGACGTACAAGGAC
TATAAGTCTTACCATTTTCTTCCCGAATACTCAATCGGCACTCCTGATGATAGACTCTAT
GCACTAAATGTACCGTTAGTGGCCATGGACAAACTGATTGGCTCTAAGCTGCCAGGAGAC
CTCGCGAAATCCCTACTACAGCCGATTCTCGAGGGCCTGCTCGAAAAACATAATGAGAAA
CTCGGTGAGTACGCTTACCTCCATCGACTTCACCAATTGATACTATTGAAGCCTACTATT
TGTTCATTACTTGAATTAAGTATAGCCTTGCTACCTTTCAACAGACTTTTTCTGGTCTAA
ACTAAAGACTAACTGCGTTACAATTTTTTAAGTTTGAGCTAGCCTATTTCTTTCTAGCCA
GCAAATCTTCATTCTAGTTGTTTTTACCAACCATCTTGGTTACATCGTTACGGTGCCACA
GACATCTTCGATTTTATAATGGATAACATGTTTAATAACAGACATGCACTCTAGTTGTCC
GTTTCGCCCCTCCTGCAAAACTGTGTTGGCGATCTCGTTAGTTACTGCGCGTGAGTTGGA
TATTGAACATTTACCTAGAGTTAGTTATCGCGTCTTACACGCGATTGGTATGGTGCTGAG
GTTCAATGTTCTGTCCTTAATGAAAAAACAGTGCATCATAATTTGTGAAACGAATATCTT
AGAAATAAAACATACTCCAGAATTTTTATCACTCTCCAGACGGGTAACCTTAAGCCCTTC
AAATCGTATTTTTTTCGTCAATGCCGTAGTAGTCCTCCAAATGAAGCCGAGGTCTGGTAG
AACCTGGGCTTTTACGCTGTTTAAAAAAGTATTGCTGTAAAATAATAATAAATATTTATT
TACCGAAATTACTATTGTCCCTTTATAAATAGCTAATTTTATGATAATTTACTATAATTT
ATTTTCTTCTTCCGCAGATATTAATTCAAGCATTTAAAGTTTCTTCAATGGTTTTTCCCT
GGTATCTTTCAATGTGTAAACCTGCTTCATCAATAAGACTGATATGTATATACATGCACA
CGGAAGGTTTATGACAGTTATTATAATTGGATTGCGCGATGATAACCTGAAGCGACAGCG
AACGTAGGTCGAAGACGTCCTGTTAGAATTATAGGCATCAGTTTCTCTAACGGCTTGAAA
TACTTAGTTGTTAGAGTACATAACACTGTAATTTGGTAAATATTTATGTTCTTAGCGAAT
TATTTATATTTATTTATAGCTATTTTAGATTGTAATGCATTTAGAGACTAAGATGTCGTT
TGGTATTGTAGTGTTTCTCGAAAGTGTGAGCCTTGAAGGAATCCTTTTAAAAAAAGGTAA
AAATACTAAAGGGACATTCAATAAAACGACAACTCAGCTGAAACATTAAGAAAGCTGCTA
TTCTAAACCAATTTAGACCCTTCAAATGCTGGAGGAATATATATTTAGGTCGCGTAACTA
TGAAGTTAAGTATATGCACCTCACCGGTTCCGTAGTAGTGTTTACAGAGACTTCAGGGTC
GGAAGTTGAGACCATCCAGACTGAGGGCCGGCCGAGCAAAACGTTCATTAAAGAAAAAAA
GAGGTCGAAAAATAGTATCAGACAGTAGAGAGAGAACGACTTCAATGTCTGTACCGAATA
TTATAGGTTCGAGCGTAGGCTCAGGTTCATGCTGGGAAAGCATGTTGAGGGCGTGCGTTA
CTATGAAATGTTGCTTAAAATAAAGTAATCAGTGCTGCTAGGTATCTCGAACTCTTGAAA
AACCTCGTAAACATGAAATATGGCGAGCAATAGCACGCGGTCTTGCTTCTCGTCAACACA
ACTCGTTGCGATAACCTGCAATTATCGCCTGAATTGAACAAAAGAGAATCCAGCGATGGC
TTTAACCTTTTTTTTTTTCAGATTTAAGTCTGTACGATTCTGATTGTTCCTTCGCCTGAA
AACAGGAACTAGATGGCATAGAATATCCTATCCTTGATTCTTTCAAACAGCTATCAATAA
CCAGAACAGGTAGGGTGATACAAATCACAAGTGTGCAGCTATTGAGAAGCTTCCAAAAAA
TTAACCCGTGAATAATAAAGTGGATTATATGTAACATATACCAACATGCCACTGGAAGGT
AATTCAAATGTTTGGATCTGATAAATAGCTCTGAATAAACTAACAACATTTCACTACAGG
TGCACATTTCTGAAAGAGAATGTGTTTTGAAACCGATTTACCCAGTCATTATGGTGGTAA
CCATGTTTGTCTATTAAAGTTATGCACACATTAGTCGCCTAGATTCGAAATATTCGCGGT
TAACTGGCCCTTATACAAATATTACAACCAGAAAAAGGCCAAGAATTATCACAGACTTAT
TTTTACGGATACTTTTCATGGTCGATCACAGGGACTAAGAGTGGCCACATAGCCATCAGC
GCAGCTAAAACTACCACTGGCGACGAGGCTATGTTTGCGGAAACAACCCGTAAGGAATTG
AACGCTGAAGTAGGCGTTCTGAACGCGGTTGGTCCTTTCGCAACGGTAGTTCTGAGCGAT
GTAAATCCTTTTCTGAAGCCCGAGCGAATAGAAAACGATCCGAGAATACGATAAATCTGA
AAGGAAAAGGTGCCATAATTGTGGCTAAACTGGTGGCCTAACAAAACTGCAACTGATCTT
CTTGCAAGTATAAATATAACGCAAAGGAACATGCAACTACTCCGTCGTTAGGCTAAAAGC
ACAAGGAAGCAATTGGAAGAAAACGGTACAAGAGCAACAACCGGCGGCAACAGTCAACAA
AAGAAGGTGAAATGGTAACAAGGAAGCAACTGCAAAAGGAACGACCAGTAGTGGTCAACT
TGAAAACGACTCGAAAGAATGATTTAAAGACCGTCTAGAAAAAAAAAAAGACCTTCACCCT
AACCTGTAATGATCAGCCATCTCAGTAAGACAGATCGTAGCAGCGATGGATAAAGTAGCT
AACGGAAGAAGTGATAGGCTTCTATTTCTCAACACAATGTTTGGTATGAGAATGGTGTTT
AGAAATAGAAGCTTAGAATGAAACGGATGGCAAGAAAAAGGGCATACATTATCGTAGGCT
TCCTAAATCTAAACAAACAACCAAATTTTTATAATTGATGCTATAAAACCGACTTCTTAA
GGGACAGGAAGGGTGTTCTTTTTAAATCCTACCGTTGTGAGCACAAATTTATTTAAAATA

GTAGCTGAATAGCAGGTGGCCTTAAGAGAAAAAATGACTGTATAGAACTTTCCATAATGA
AGGAGCATCAAAGGCAGACCCTCGTCAAAAGGGGTTCAGACTGAATAACCTAGAATTCTT
CAAGGATCTAATGCAAAGAGTTACAGTACAACACACATCCACAAAGACGAGGAAAAGCTT
TGCAGAGCATTTTAAAAAGCTCGTTCAGTAGCCAGTGACCCTACCACGCGTGGAGCTGAT
GCAGCCAAAATACTAATGGAGTACAGCCATTGCTGGTGCTAGAGCGTGTTTGCAAATCCA
CAGGGTTATCCAAAAAACTAGAGGAGATACATTAATGATCATTGAGCTGGTGACTACATA
TGAAGAACGCTAAGCCACCATGAAACACCTAATCGGAAACGGAAATACCAAATGAGCAAC
TCGATAAAACTAAGATAACTGGGGCCACTTATACAAAGCCGTCAATGGTAATACGCCTCT
GAAGTGACGTAACCAAGCAGAAGACCATTCACATTTTACGCAAACTGTTATCTCTGCATG
TAAACTTTAGACGTGGGGGCATATAAGCGAACATACTTTTATTTGGGGGATAGGCCACCC
ACTGTAATCCTAGCGTTTTCTCGAAACAATTAGCATCTTCTTCGACGATTTTCCGGAACT
AATCGTGAACATGGCAAGATAAATCTTTAAGGGGATAAGCAGGCTGTCAATGGAAGAAAG
TGAAGATGGTGCTGATACCAAACGGCAACGTGAGATACAGACGCATGTGCTTAACTAGCA
CCCTTATAAAAGGTTTCGAAGCGATTCCAGAAGTTCGCAAAGTTTTATACGTTCTTTGGC
CTACGATATGGTTTCAAGAGGCGTAGCTCGGTACTCTTAGCAACAACACGGGTGCTCCAG
AAAACTGAACCTAACTTCAAGCATATCGTTTCAAATAGGATATCGAAAACAGGCTTAAAA
TGTGCCCCAGAGAAAAGTAAGCAAATCTTGCTCATTGTACAAGAAAGATACGGCTAATCA
ATTATCATCTAAGATCGGTTACCAAGTGGGGCAAGGTACTTGGGAGTGTTTTCGTACAAA
TCACTTGATAGTAGTTTACGTGTAAAAACTACTGTCGGTAAGCGGTGAGTTAGATGGCGA
GGATACTCCCAAATATCTATGAAAGAATAAGCGAGGCCGTCACAGGCTGTTGACATTGGC
CACGGAATTGATCGCTCTGGTCGGAGAACCGGCATCAACGACGACAAAAAGCAAAAATCT
TTGCAAGACTAGAGAATAGTCACACTCCGCATTATATAAGCATATCGTACGATCAGCACG
CCTGCAGCACTTCGATTAGTTGAGACACGACGCTGGGACCTGAAAATCAGAGAAAGGAAG
TGTCCCTGGGGCAGAGACGATTTACAGAAATAAAACAAGCGGTAAGGGTCATCGCCATAG
AAGGGTAGCACCGAAAATATACGGCCATCCCAAAGGGTAATCTTGTATCACTGGTCAAAG
CCGATGGGCTGGACCAATCTGGTTTCACTAAATCACTAAATTCACTAATTCACTAAAATC
ACTAAATTTTTTTTTTTATCTAAACCGTAATAAGCTATGGCAATTTTCAAACCTATCTCT
TCTTTACGCTTCGCTAAAGACTTGCGTCTATTGGAATTTAGGGGCTGATGACGACCCACC
TTAAATTTTACTGATCTGTTCGACATATAGGCATTCCTAAAAACGCGGCAAAAAGGAAAA
AAAAACAGAAAGAACCCAACATTGTGATTGAAGAGGCAGAAATGTACCAGTAATCCCGAG
AGAAGGCAAATAAAAATTTGTGAAGTCTATATATAGCGTTACGGGCGACCAGAAAATTGG
GTCAACGACCATAGCAGAGCTATTGAAAGAAAAAAGATAAAAAGAGCCAGATTTGCAGAG
ACGATACAGCTGCAGATGGCACATAGCGCGTCAAGAGAAGTTGGAAGTTCAAAAGGAGAC
CTGAGATTATCGAGCCGGTCAATTTTTATTCATCGGTCTGTATTCCTGTTCACGAAAGTC
CGAGTTTCACTAGAGGGATCTAAACATGAAGTAAATTTAGAAAAATAAAAACAATAAAAC
TGGATATAACCACACCACACTATCTGCCAAGAACCGACAGTCTGGGTGATGCTTGGCAAA
AGCACCAGACATGAAACTCGAAAAACATGTGCCGGACCACGTATAAGAAAGAATAATTAA
ACATCATTCTTCCATTATGCTGAGATTACTAGTAACGGAGGCTCACGATGCATGATTTAC
TTTCACACTTGTAAACCATCTGACAGCTAAAGCTATTGTACAGTTTCTGATATTTCTCCT
ACCCATACTATCACAAGCCTCGATTCGTCTATTGCAGGTATAATAATAGAACAAATTGAG
CGTTACGTTAGTATCTAGGTTCGATGTGTCCCAGTCCTTACATAGATTTTACTCAAAAAT
GTTTAGCCATATCTACGTACGTGAAGTGCACAGCTTTATGAAGTTGTCCACAAAATAAAC
AACTATTTTAGTATGGTGGCTTACCATAATAATATTGACGTGTTGCTAGCGGCTCGTTTA
ATAAACGTAAACACAATTAGAGGATATTAAAAGGACTTGAAAGGCTTACCCCTTTTTCCT
TTTTCTCATAAAGTGACAAGACAACTCGTGACCTTAGCTTAACACTGGCACGCTGAATTT
CAAATAAGGGTGTATTCAATAGAAAAAATTAAGGCAGTCTCATAGAAGACAACGAACAGT
TACTTTGCTGCTGAGTTACTGTAGCCTTCTCAGATAATTGTATACAATAAAGAAAATAAA
TTTAGGTAAGGGCTTTAAACAGGCGGTCTTTATGACCTGTGTTCTCAAAAGCCAAGTGCC
AATTAACCTCGTATAGTCTGTCGCAGGCTTCAGGTTGTTTGTTTGAGCTAGATTTCTGAC
TTTTAAAGCTAGATTTTTAAAGCAGAAGATTTCACTAATGAAACTAATGAAGACAAGCAT
TTTCGTACAAGGGTCTATAGCCAAATTGTTTTTGTACGCGTCGTTTAGCAAGCCCAACAA
ACTTTTACTACATCCTTTAATTCCCCCTTTCCTCTTTCTTTTTAAACACACGTAGCACTA
AACCACGGCTTTTTTTTGCTGTTTCTGTTTATGCGCTCACGATATTCATTCATATAAATT
CATGTATAACGCAGTTAATGTAACTAATATTAAGTAAGTTTGACTTAATTTGCGTCTTCC
AGTTGCGAGGTGAAATCATGGTAAGTCTAACGAATACGGGAAGTATGTGGGAATTTCTCA
CCTTATTTTTTGAAAAGCATAATGTTTAGATCAGAATCTTGGTCAGCTCATTGTGCTTTC
GCCCAGAGTTTAGGCCCCTTGCCTCAATGTATTGGTATTATAATTTTTATTTTACTAAAT
GATTTAACGTTTGTACATTGAAAAAATGTCATAGAGAACGGAACATATGATTAGACTAAT
TTAAAATTTTATGCTTCATGTGGCGCAACATTCCAACAAAGATAGAATGATTTTCTTGAA
AAACTCTTTGCGACGGATTCTAGCTGCCCCTTCTAAAATAGGTTTCGGTACCTCTGTTTA
GCAGAGCATCAAGGACCTATCAGGACACGGGCAATCTAGAACACCTAGGGGATTACTTTC
GTGTATTAACCGCTCCCCGTATTTCTTGAATTTATTTTAGGAGATAAAATTGTGCCGGAT
TCTTACTCAGACCACTTCTGTGTTATCTACTCTCGGTAACAAACACTTTAGATTTGCACC
GTCACGGGGATCCAGTAATTTCGTTGGTATTAAGTGTATGGTTTATAGTCGCTACCGCTC
GCCTGGTTACTAACTTCTATATAAGCATTGTCTTCTTGTGTACGTGCATACGTGATGATT
AACAGTTCGATTGCTTTTCTCTAGGCCTTATGAGGTGCTATAAATAATTCTGATGCGAGT
TATTCAAAAGGTAGTATTACCGTATAAAAATGTTGTTTTCAAATCCACAGCCCAGTGAAT
TTGCCAAAAACCCAACTTGGCTTTAAGCAGTCCATTCGCATATATTACGATAGTGTTACG
CGGTGGGTAAAATGTGTAAGAAGTAAGTTTATGCAACTTTAACTACCGTGGCAGGGATTG
ATAATAGAATTTTTGTGTTTATCCGAGATAACCTAATATTTAATTACAGGACCTTACATA
GCTTTGAATAGTCAGCTGCCTTTAGATATTTGTGCAAAAATATTTACTTCAGTTCAAATT
ATTATAACGGATGTATGTATTTGAAGTGTTTGAGTGTTGCCTGATAGAATTTAGATCAGT
AAATATGTATTTATCAAAAATGTTCGGATAGAACAGCGCTTAATTCTTTGTGAACATTGT
GCGTGGCGTGGAACAGTCCACGCACAACGGAACACTTATTTATATTGGAAGAGTCAAAGG

TAAGTAATTTTTCTCTTTTTTATTTTTGCAGCAAAAAGCAAGTCATATTCGTCAAAGCTA
AACGTAAATATTATATAGATAAGTATAGAAGCTATGCGGTAAACTTGAAATACGGCAAAA
TTCCTGTTTGTGAAAATAAATTTTGTAAGAAAGATTTAAACCCAAACGACCTACTCGCAG
GTCTTCTATAGGTTCTACCTGTAGGCCGACGGTAAGCACTACGATTCCAAGATAACAAGT
TGGTCTAGTTTGTAGTGATTATTCGATGCATCTGAAGGGTACATCGGTAGCAATTGATTG
CAATTTACGTTAATGATGTGTTATGATTTGATGTATGCACCGGCGGTAAAGTAGTTTCAG
TTAGACGTCTTTTGGTCATCGGACAGATACTAACGATTACGTCTGCGCGGTAGCTTCAAT
TAGTGCCGTGACTCTGATAAACAATCAGATTTTTCTGTAATGTATCGATGTCAATTACCG
CTTCAAACACTCCCTAAACATTCAAACGTTCCAAAAGCGCCAACATGTATGGAAAAAGTA
AATACGTTTTAAAACTGGACAGCTTTTAAAAAGCATCTTTCTTATCAAGGTCGTTTGTTT
TTATATGGGTAACATATTGTATCAGCGATTTTGCTACGTAGCACAATACGATCATAGCAG
CAGAATATTCGCTTCTACCTTTTAGTTGTCCAACGCAACGTAAGTGAGATGCTATTTGAT
GGCTACGCGGTACCAATGATGAATGAATTATCTAAGCTTGCTGCGGCGTTCATGCCTGAT
ACCAAGCTTCCGGCTATCAGCAACTTTGGCCTGTTCTATAATAAGAACAACACGGCTGAT
CAGTGGTTTACCGTCGGAACGGGAGCGGGCGAGTATCCTTTCGCTACGATCCTCGAATGG
AATAACCATACGTCATTAGACTTCTGGAGCAGAGATAATTGCAACAAGATCAATGGCACC
GACGGTGGACAATTTTCGCCTTTTGTGCGAACTGATCAAAAGCTGTTTGTTTTTGCCACA
GACCTCTGCCGATCTATATACTTTGAATACGAAAAGAATACCCAGGTTAAAGGAGTTGAT
ACGAAAAGGTTTACTGTTCCGGATGCGATTTTTGCCTCTGGTAAGCGGCAGCCAGAAAAT
CAATGCTTCTGTGAAGCGATAAACCGATGTCATGAGGGTGGAGTTATTCCCCTAAGCTCG
TGTCGCAAAGGAGCGCCCGTCATGCTTTCTGCTCCACACTTTTATCAAGGTGATGCTAAG
CTGGTGCAAGACGTTGCTGGATTGCGACCACAAAAGAAGTATCACGAGACTTATCTGGAT
GTACAAACGATGACAGGGCTGGTTCTGCGTGCCGCTAAACGTCTTCAGATTAATATCGAT
CTCAAGCAAAGCGATCTACTTTACCCGCTCGCGAACGTGTCCAGCCGAGTCATGCCTATT
GCCTGGGTCGAAGAGCGTATGGAGGCAACCGGCCCGATGACGGATGGTCTCAAAGAAAAA
CTAATCTTTCCTCAAAAAATGGGTGTCCTGATTTCCACTGCTGCAGCGCTCGGTGGCGCT
ATCGGCGCGATCGCCGCCATTCTGGTCATTGTCGTACGCCAGGCTGGCACAACTAGGAGC
GCCAAGCTCCAGCCCTCAAAGTAGACCTAAAACAAGCAATCATTATCTTCATCAAAGGAA
AAAGCTAGAGAAGACTCGGCAGCTACCCATTTTCACTTTAGAGGATTAAGGAGATACTTA
GACAAGCTGATCAGCTCAAAGAACAAATCATTACACAATTACAAGTACATAATACAATAG
CATGTGAGGCAGTCGAACTTCGGAAAAGGCTGAGCAACCCTCTGAGGGTTTGCCATGTGT
AAAAACGAGGGTTTCGTTGTCGATGGTCCATTCAAATTATCCTAAACAACTAAATAGAGC
AAACTGAGTGTCTTCGACGTAAACGTATCGCCCGTAGGACGTTGGCCGAATAAAAAATGT
TCCTAGTGAGACGGTTATGATACGGTCCAATGTACAAAAGGAATTTCCCCGGCGACAAAA
GATCGAGCAATCGATGAGAAATTGCTCACGACAATGAGGTACACGCCAAACGTACATAGC
CTATTCATAAGTGAAATAAACCTTTTGAGAATGGTAAGTTGCAATTCGGTCGTGTGACCG
TGATACGAGACACAATCACGATGTGAAGTAATGAAGCAATGAGAAGCATTTAGCTGAGCT
TGTTTGAAGGATTCACGTCGCAAATACAAAATGTAATAAACTCTCGTTCATAGCGGAATG
AAATCTAAAAATTCATATACTATATATAAATAGAATATAGATATAGATCCTTGCTTTGAA
GCGTCACCTGAACTTTTTGAATTTTAATGAAAGCAATGTACGAAAATAATAAGTGACGTA
GCTATATACTATTGTGCGATGCGAGTCTCAGTCATCAACTTCAATTATGAATTTTTATTA
ATCTACTAACAGATTCAATTATTTTTATTACCCGTGGCCCGACGCTAACACATTTTAGGA
AATCCTGTTTATGAAACACCTGCCCGAACGCGGTAGACTTCAAGAGGACTAGACGTCACC
TTTCTTTGGGGGCTGGACAAGGACAAAAAAAAAGTCGATCATTCAAAGCTAATGATATAAC
TGCGATTGGTTGCATCAATATTATGTTTATTATTACCTATATATATATATATAT

Fig. S3. AmOBP-like and Vd-OBP amino acid alignment

```
CLUSTAL format alignment by MAFFT (v7.309)


Vd14320         M-----------------------------------------------------------
Agam            MKHLKAFDEAQNDIKAVQKRLSTSSTILSGIQKNMAHLNLLQIGVLSLIAVGSVFAGNPC
Agam            MGQR---------------------------QRVVVQLALCFLTFGALLQAGVLAGDNPC
Vd22214         M------------------------MIPPWFQRNSGLPYIIVILLGITLEAQFGFSET--
Vd18044         M-----------------------LKVIGWVRAN--------------------------
                *

Vd14320         --------NAAVYIVTAMLACCCSADTASGDHASDTSQFGKWRTCLA-DKLPAK---KRE
Agam            LKGPPVPKNAAECCVTPFLV-----------------EPSAFMTCHS-KWIGQT---KRQ
Agam            AAGPPVDTNPAECCPKPMLV-----------------DGTIMMDCYK-KYGEQT---KKQ
Vd22214         -----VNMDQYKFHMSKVLSLYM--------NGFNVHQLGTILVSMAYSFSPAE---DRP
Vd18044         -----GNMNLTKYM----------------------------------DYLSQAGLDDKT
                        :                                       .        .:

Vd14320         VFDECFIKPGGTDMNKFRRG---LNCVLTSDGLL-KNHKVD---LKKM--SMTAA-----
Agam            M-----------AMEGIPRGCCVAECVMNSTSLY-SNGKIDREALTKL--YLAST-----
Agam            L-----------QMDGIPRGCCIAECAMNATNMY-ADGMLKRDDLSKM--FMDAV-----
Vd22214         VVEQCL---GQHDNETVTEL---IKCYMEKTNMMDSDKKCN---LTKLKEFITVK-----
Vd18044         L-----------ELTKTAHEKC-------SQRAL-SDGSGDYKEQDKI--YVECIFRHFD
                :                 .                 :   .     *:   :

Vd14320         SAGSPELKKA----YENCPKDDKNTSEDRSIKCLIDHLEVDCKLASK-------------
Agam            KSMAPEWNKITLDAIDGCFK---------MADTIKDEIEAGAKLTPAFEGEQICHPISGT
Agam            KD-KPEWMSLVRDATNACFE---------LAEKKMDEIEAGAKLEPSFEGEKICHPISGT
Vd22214         MAAAPPIAVAVLQKYAECVC---------QISGAEGMICAQQKTMDYV--RTLCSAIVGD
Vd18044         KTCPPNFQAAVKQGYIVGITQ-------ISFVPRYDEVEVESDTDAETEGQA--GPSVAD
                    *                              . : .  .

Vd14320         -------------------------------------
Agam            ILACMGMTLFAECPAKLFTVNDDCNKLKSYHSKCPFL
Agam            ILRCMGMMMFAQCPASVFNVNENCNKLREYGSICPMI
Vd22214         E------------------------------------
Vd18044         A--------TGEAAA-----NDNAN-----GSGGPQ-
```

Amino acid's alignment of three Varroa PBP/GOBP-containing transcripts with insect's OBPs: Agam7 (*Anopeles gambiae* AGAP007287-PA), Agam8 (Odorant binding protein-8). Alignment was done using MAFFT in "Auto" strategy.

Fig. S4. Varroa and insect OBPs amino acid alignment

```
CLUSTAL format alignment by MAFFT (v7.309)


AmOBPlike1      MTHL-----TAFATPFVAFVPGAVFKG---FLQLLLGDGGS-HLSH--------------
AmOBPlike2      MVAF-----KAALLLCVLVLPAAYCQS---AAEPPRPDI---------------------
Vd18044         MLK---------------------------------------------------------
Vd14320         MNA---------AVYIVTAMLACCCSA-----DTASGDHAS--------------DTSQF
Vd22214         MMIPPWFQRNSGLPYIIVILLGITLEAQFGFSETVNMDQYKFHMSKVLSLYMNGFNVHQL
                *

AmOBPlike1      ------------PGQVH-----IV---------P--FHELVAV-----QDTFQALSELAH
AmOBPlike2      -NWGKCPQLQP-SKEERQQKALVIDTCLEKVPLPDVEHANETVIQQHREDVTTCALHSEG
Vd18044         ----------------------VI-----------------------------------G
Vd14320         GKWRTCLADKL-PAKKRE----VFDECFIK---P-GGTDMNKF-----RRGLNCVLTSDG
Vd22214         GTILVSMAYSFSPAEDRP----VVEQCL-------GQHDNETV-----TELIKCYMEKTN
                                      :.

AmOBPlike1      LCSSRCVVAALKHWGFVSGKLIHHALPPLVKLGPAALSPAASAGPGQEAGCQQKHHS---
AmOBPlike2      WFNKNGQYRFDRARTEILNK--------------KLAADVEPKVLAKHDECKKEAEEKFA
Vd18044         WVRANGNMNLTKYMDYLSQA--------------GLDDKTLELTKTAHEKCSQRALSDGS
Vd14320         LL-KNHKVDLKKMS------M-------------TAASAGSPELKKAYENCPKDDKN---
Vd22214         MMDSDKKCNLTKLKEFITVKM-------------AAAPPIAVAVLQKYAECVCQISG-AE
                           :                                      *

AmOBPlike1      -------------SLHHKSSTPTRKDK---------------------------------
AmOBPlike2      HQFVAQVQLYQACMDYHISQICGIQIQGAQ------------------------------
Vd18044         GDYKEQDKIYVECIFRHFDKTCPPNFQAAVKQGYIVGITQISFVPRYDEVEVESDTDAET
Vd14320         ----TSEDRSIKCLIDHLEVDCKLASK---------------------------------
Vd22214         GMICAQQK-----TMDYVRTLCS-------------------------------------
                                :

AmOBPlike1      ----GNSVV-------------------
AmOBPlike2      ----GGAAAPA------------HG---
Vd18044         EGQAGPSVADATGEAAANDNANGSGGPQ
Vd14320         ----------------------------
Vd22214         ------AIV----------------GDE
```

Amino acid’s alignment of Varroa PBP/GOBP-containing transcripts with OBPs-like transcripts of A. americanum (as published by Renthal et al. 2016). Alignment was done using MAFFT in "Auto" strategy.

Data S5. Varroa IGR transcripts (46), as found by conserved domain search

		* Conserved domains presence		
**Transcrip**	**Length bp**	IPR001828	IPR019594	IPR001320
Vd17150	4310		*	*
Vd18743	6368		*	*
Vd18850	2417		*	*
Vd19098	4303		*	*
Vd22240	1315		*	*
Vd18951	6474	*	*	*
Vd20015	7051	*	*	*
Vd20731	4898	*	*	*
Vd20855	5670	*	*	*
Vd21106	4056	*	*	*
Vd21675	4956	*	*	*
Vd21714	9469	*	*	*
Vd21743	8234	*	*	*
Vd21758	3164	*	*	*
Vd21835	8381	*	*	*
Vd22269	3992	*	*	*
Vd22292	7715	*	*	*
Vd370	4530	*	*	*
Vd52944	4189	*	*	*
Vd12161	706		*	
Vd12833	1215		*	
Vd71562	525		*	
Vd8338	678		*	
Vd12191	2335	*		
Vd14911	2584	*		
Vd15706	4713	*		
Vd18297	10525	*		
Vd18566	4671	*		
Vd19478	5267	*		
Vd19637	3044	*		
Vd2075	760	*		
Vd21871	10135	*		
Vd21907	8184	*		
Vd21914	9038	*		
V21983	6868	*		
Vd21983	5970	*		
Vd26894	1075	*		
Vd4840	869	*		
Vd7464	885	*		
Vd78179	346	*		
Vd99137	291	*		
Vd12161	2060			*
Vd1332	1428			*
Vd13379	1946			*
Vd22215	5197			*
Vd22215	972			*

Data S6. I. scapularis IGR transcripts (17), as found by conserved domain search

		* Conserved domains presence		
Sequence name	Length (aa)	IPR001828	IPR019594	IPR001320
ISCW000549-PA	487		*	*
ISCW001842-PA	760	*	*	*
ISCW005598-PA	852	*	*	*
ISCW007957-PA	658		*	*
ISCW008225-PA	238		*	*
ISCW008266-PA	905	*	*	*
ISCW009282-PA	1113	-	*	*
ISCW010976-PA	598	-	*	*
ISCW012402-PA	732	*	*	*
ISCW012407-PA	219		*	*
ISCW015703-PA	423	?	*	*
ISCW016542-PA	725	*	*	*
ISCW017534-PA	914	*	*	*
ISCW017535-PA	878	*	*	*
ISCW022877-PA	454	-	*	*
ISCW023268-PA	736	*	*	*
ISCW023274-PA	808	*	*	*

Data S7. IGR sequences (105) for the phylogenetic tree in figure 4

>DmelIR7a

MFHHLWLLMGLRSLAMGALHPPQPEAMTPLVAAALEILAEQVSPSQSTLAVMDLTQDAEHRDERQEQLMTI
ILRSVGSEMALRTFQKPPAEVPASFVVFLVNSAQAFNTLGFHFTDIHSTREFNFLILLTHRMSSRAERLQVLRDI
SRTCVRFHTSNVILLTEKRDGVVLVYAYRLLNMDCDLSVNLELIDIYKNGLFRHGHEARSFNRVLSLSGCPLQ
VSWYPLPPFVSFIGNSSDPEERAQIWRLTGIDGELIKLLASIFDFRILLEEPCNKCLSPDIKDDCSGCFDQVIISNS
SILIGAMSGSHQHRSHFSFTSSYHQSSLVFIMHMSSQFGAVAQLAVPFTVIVWLALVVSSLLLVLVLWMRNRL
VCGRSDLASHALQVLTTLMGNPLEARSLPRSSRLRILYAGWLLLVLVLRVVYQGKLFDSFRLPYHKPLPTEIS
ELIRSNYTLINQEYLDYYPRELTVLTRNGSKDRFDYIQGLGKEGKFTTTSLIATMEYYNMMHWSTSRLTHIKE
HIFLYQMVIYLRRHSLLKFAFDRKIKQLLSAGIIGYFVREFDACQYRKPFEEDYEVTPIPLDSFCGLYYISLIWL
SAAVVAFILELLSQRIVWLRRIFE

>DmelIR7b

MKYWLYILSCCSLVASTMESSSDWDLAEALAQVVANSEMGRFKTLYIYTHTNSQSTGGHLEELLDQVLMIV
PNNLQARRLLLQQSMEYKPYVHAVLALVDGLPSLSAIYARIRATQDLSHTLIYMSMPTDAYGEEMQATLRFL
WRLSVLNVGVVLRPPGDHILMVSYFPFSALHGCQVISANVVNRYQVGTKRWASQDYFPSKLGNFYGCLLTC
ATWEDMPYLVWRPDGSGSFVGIEGALLQFMAENLNFTVGLYWMNKEEVLATFDESGRIFDEIFGHHADFSL
GGFHFKPSAGSEIPYSQSTYYFMSHIMLVTNLQSAYSAYEKLSFPFTPLLWRAIGLVLILACLLLMLLVRWRH
HHELPRNPYYELLVLTMGGNLEDRWVPQRFPSRLVLLTWLFATLVLRSGYQSGMYQLLRQDTQRNPPQTISE
VLAQHFTIQLAEVNEARILASLPELRPEQLVYLEGSELQSFPALAQQSGSSARVAILTPYEYFGYFRKVHPMSR
RLHLVRERIYTQQLAFYVRRHSHLVGVLNKQIQHAHTHGFLEHWTRQYVSAVDEKDESVARIASTSYSTLDG
IDGDPSLSESEEDQQVAPVRQNVLSMRELAALFWLILWANLGAVVVFVLELLLPRIKLRKILRKMKSDIKKQI
SKLVRK

>DmelIR7c

MLHSAVHNVSLVYALVWAIDNYYGMATSTPLAVVQFPTSRESRRLHNDLIDAALGRSSGTGRIQFLLEDDRV
EMTETDTDPPPPSGLTGRPIAIWFLDSLRSYFRLEMYLNQLGSPYKRNGFFLVIYTGLEDQPMESLKIMFRRLL
NMYVLNVNVFLQRDGTVHLYTYYPYGPHHCQSSLPVYYTAFQDLAAPANGFGLTKPLFPRKLTNMHGCEM
VVATFEHRPYVIIEDDPKTPGGRSIHGIEGLIFRSLAERMNFTIKLVEQKDKNRGEILPDGNFTGILKMMVDGE
VNLTFVCFMYSKARSDLMLPSTSYTSFPIVLVVPSGGSISPMGRLTRPFRYIIWSCILVSLIFGFVLICLLKITALP
GLRNLVLGRRNRLPFMGMWASLLGGLALYNPQRNFARYILVMWLLQTLILRAAYTGQLYLLLQDVEMRSPI
KSLSEVLAKDYEFRILPALRTIFKDSMPTTNFHAVLSLEESLYRLRDEDDPGITVALLQPTVNQFDFRSGPNKR
HLTVLPDPLMTAPLTFYMRPHSYFKRRIDRLIMAMMSSGIVARYRKMYMDRIKRVSKRRNLEPKPLSIWRLS
GIFVCCAGLYLVALIVFILEILTTNHRRLRRAFNVINRYAA

>DmelIR7d

MDIRCVVALLLGLCKVQAVVWPHQHLLEEQLASQISATLQKIFINGLAVYNFGVFISTSYEEMDRDRVILVHQ
VLNRNLYPPNFPVAVVLASKMNRKITAQVFTQLLFVQNAEQAIAIAEGVNRNGLCVIVLLTSQPERPIMTKIFT
YFMQERYNINVVILVPRLHGVQAFNVRPYTPTSCSSLEPVEIDIKDGDLWDVFPRRLKNLHGCPLSVIVWDIPP
YMRINWKSSDPMDGLDGLDGLLLRIVARKMNFTLKLIPNEPNGLIGGSSFMNGTFTGAYKMLRERRANITIG
CAACTPERSTFLEATSPYSQMSYIIVLQARGGYSIYEVMLFPFEKYTWLLLSTILGLHWIVGSRWRMPSPILAG
WMLWIFVIRASYEASVFNFIQNSPVKPSPRTLDQALSGGFRFITDHASYRMTLKIPSFQGKTLISAGQPVDVFD
ALLKAPWKTGAFTSRAFLADHLVRHRKHRNQLVILAEKIVDNMLCMYFPHGSYFAWEINKLLFNMRSFGIF
QHHSQILAWDNLPTTTDTDTPGKRIHSSTESVATGFAESMSFVVAALNCLMGALCISIVVFGLELLSRRRHWT
GLEWLFERV

>DmelIR7e

MNISALLNSYYDLSGEQMNHINEFVARAVLHVVHHYILSVTPSLVLTLCCRSNHTCNFYNKMMSTLFREWG
LAPLQIVNVLRGVPWHPVPGRRHFNVIFTDSFAAFEEIRMEYYSREYNYNEHYFIFLQARDRLLQGEMRLIFD
YCWRYRLIHCSIQVQKSNGDILFYSYYPFGEHGCSDMEPQLINRYNGSMLVEPDLFPRKLRNFFGCPLRCAL
WDVPPFLTLDEDQEEVLRVNGGYEGRLLLALAEKMNFTIAVRKVHVNMRDEALEMLRRDEVDLTLGGIRQ
TVARGMVATSSHNYHQTREVFGVLASSYELSSFDILFYPYRLQIWMGILGVVALSALIQLIVGRMLRERMGS
RFWLNLELVFVGMPLLECPRSHTARLYCVMLMMYTLIIRTIYQGLLYHLIRTHQLNRWPQTIESLVQKNFTV
VLTPIVQEVLDEIPSVQHMRFRLLEANSELDPLYFLEANHQLRQHVTASALDIFIHFNRLSADKVHQRGEQGS

GAHFEIVPEDIISMQLTMYLAKHSFLIDQLNEEIMWMRSVGLLSVWSRWELSESYLRNEQSFQVLGTMELYAI
FLMVLVGLIVGLLVFILELVSMRSIYLRKLFT

>DmelIR7f

MNTTSDSNAGSSLSSGSGYSIYKSYLENSRIDMQGEDANLYVARALRLVIENVLAQLSTTLVVTISTRHLGTA
HWFEYMMNILMDSWRMVAVQLLRIRPDLVVNPVPGRKRVSLLMVDSYQGLLDTNITASNANFDDPDYYFIF
LQARDHLIPKELQLILDHCLAHFWLHCNVMIQTAQVEVLVYTYYPYTADACQKAYPIPVNTFDGRKWKASQ
MFPDKLSQMHGCPLTVLTWHQPPFVELVWDPKHNRSRGSGFEIQLVEHLARRMNFSLELVNIALLRPNAYRL
AEGSSEGPIEKLLQRNVNISMGYFRKTARRNQLLTTPMSYYSANLVAVLQLERYRIGSLALLVFPFELSVWML
LLLALLIHLGIHLPSARRGNEEDGGGGLQVVALLLGAALARLPRSWRHRFIAAHWLWASIPLRISYQSLLFHLI
RLQLYNTPSFSLDQLLAEGFQGICTANTQRLLLEMPQLARDPDSIQSVDTPFDWDVLNVLTRNRNRKIFAVAN
QDVTLSFLHSSAHPNAFHVVKQPVNVEYAGMYMPKHSFLYEKMDDDIRRLDASGFIHAWRRASFASVHRKE
QVHMTSRRYINHAKLSGIYMVMAGLYLLAGLLFAGEVLLRQRN

>DmelIR7g

MNVTSLLNFESMKYIGAQTQAASINHHVAQALRVFIEDFYQRIAPAFIVVLSCRRPSPMNFYRNIMQLLYESV
DTMIVQLVLVELGRPRRIAGPRTHNLLLVDSLDALLDIEIHTYTAQSDTSEYYFIFLQQRDALIPHDMQGVFAY
CWRHQLINCNVMTQSSGGQVLLHTYFPYAPGQCNDSQPTRINMFLGESWKHRDYFPSKLHNLNGCPLIVLA
RKVSPFLDLDEGQRELRGLEGRLLQELSRRMNFSIQFSGLQDQLKNRTTWTEKQLLQKLVQERIAHLAIGYV
RKRIQYATNLTPVFPHYSNRVVGCLLLNAHNLTSLEIWSFPFQALTWICLLLFASWLIFGLIVRSMYSALLFFIL
RYHLHQRLPGNLQDLTHGDYAAVMGRTTLQDLREVPSLQDLLGLKSVIVTSEREEEVLRTLDRCTLREGAGS
HPLFFGLISQDALLHLTQRGHRAGAYHIIPQDVLEQQLAIYLQKHSHLASHLDHLVMSIRSVGLVHHWAGQM
ASERYFRSRFLYREKRIRQPDLWAVYILTAGLYLLSLVVFICELLASRRAGL

>DmelIR8a

MELPLLVLLLALRFAGSEVLKITFWIEPVQRAEFDTDIAMVLKELDALRLDVKVDDTTLTLTRSEDGLDMQR
FCEILSTVGASAVIDLTYSHWEEGYNLVRSLGIGYVRLERIMRPFLDMFGDFMRQKRANNVAMVFMNARDA
VEAMQQMLVGYPFRTLIMDASQTDPGQHFLERIRSLRPAPTYIALFARAAAMNGIFEKVQKADLFQRPLEWH
FVFLDTRDRVFKYRRQAELCTRFTLNPRAICRSMPMPDLYCGSGFTMQRAMLLNVLRSLINAAQVSPGYPLA
IYQDCNATASSSEVSDPLEKDDYNWLDMVHWSNFLAYAPPLPHIQDQFQSPVPGLTFAVNISAGYYSSEHEA
KTDLAAWSSVGEMRLLNETISPARRFFRIGTAESIPWSYLRREEGTGELIRDRSGLPIWEGYCIDFIIRLSQKLN
FEFEIVAPEVGHMGELNELGEWDGVVGDLVRGETDFAIAALKMYSEREEVIDFLPPYYEQTGISIAIRKPVRR
TSLFKFMTVLRLEVWLSIVAALVGTAIMIWFMDKYSPYSSRNNRQAYPYACREFTLRESFWFALTSFTPQGG
GEAPKAISGRMLVAAYWLFVVLMLATFTANLAAFLTVERMQTPVQSLEQLARQSRINYTVVKDSDTHQYFV
NMKFAEDTLYRMWKELALNASKDFKKFRIWDYPIKEQYGHILLAINSSQPVADAKEGFANVDAHENADYAF
IHDSAEIKYEITRNCNLTEVGEVFAEQPYAVAVQQGSHLGDELSYAILELQKDRFFEELKAKYWNQSNLPNCP
LSEDQEGITLESLGGVFIATLFGLVLAMMTLGMEVLYYKKKQNALEITQVRPVNDSSGSGGNSSTAPPTATST
TKQAWHIPVLEAEEKPAKVSPPPSFETATFRGKKLPARITLGDGKFKPRHGLYARRNLGASDSHSGYME

>DmelIR11a

MRFAILWLFSGCLLPGIQVGIWVVVRAQPTGRDVLLSRLGNQQNELNTRRLANASSYLTRNYIANRINTLVV
REICVECPYELSERQRQLVDQILASLAPELSVLLHKGTAEETTWEYTLFVVNDHTAFTGQVFIFPDELLEREFF
CIVVVSEIQSRQFVRQTVGSIVKSNLQMHFVNVVVVAQLEDGTVGTYSYKLFKANCTPGITVRQINHFDRITG
KPQQSMPDLYPVRNGHLGDCPFNVGAAHMPPHLIYKRHKDPPPASNVSIPAEDLAGIDWDLLQLLAKALKFR
IQLYMPQEPSQIFGEGNVSGCFRQLADGTVSIAIGGLSGSDKRRSLFSKSTVYHQSNFVMVVRRDRYLGRLGP
LILPFRGKLWGVIIVILLLAVLSTCWLRSRLGLSHPIEDLLTVIVGNPIPDHRLPGKGFLRYLLASWMLLTLVLR
CAYQARLFDVLRLSRHRPLPKDLSGLIKDNYTMVANGYHDFYPLELTCRQPLDFSARFERVQRAAPDERLTT
IALISNLAYWNHKHPNISRLTFVRQPIYMYHLVIYFPRRFFLRPAIDRKIKQLLSAGVMAHIERRYMQYENKR
KVASNDPVLLRRITKSIMNGAYRIHGLVIVLATGMFILELLAGRSNGRLRRWMEWVHQ

>DmelIR20a

MLASLNRSTGLSAELLDLYGLVVHFLLSGEHTTLVYFNPAGLDCSWGVLWQRNLTAHPQIVWQRNYSYPDL
YYQFNAKLLVLACLPMDSRAAIQLEILANSLSHLRTVVRLLIEVAGPDQVTLARQYLSFCLRRSMLHVELYFR
DYHHSLILYSFRAFPSFELVMRWISVGQGVKLFLHKLDDLRGHRLRVIPDLSPPNTFFYRDARGDNQVTGYL
WDFLATFAGRLNAGLEVVRPSWRAGSASDSSYMLEYSAKGLIDVGLTTTLITKWNLWAIHQYTYPLLVSSW
CTMLPVEKPLATPDLFGRIVCPTLAMTLLLIILVTWLVFRQLRCLTRLKNSRPARIVPHLLTLLLLTTCSAQLLS
LLIFPPYHVRIASFEDLLRGDQKILGMRNEFYNFDGAFRARYAGVFYLIDDPNELYDLRNHFNTTWAYTMPYI

KWLVIKTQQRHFSKPLFRWSKDLCFFDFMPTSVIVAPDSIYWESIKDFTFRIHQAGLMKHWIRKSFYDMIKAG
KMSIKDYSDLETLKPLNIGDLEIVWRVCGAAIAVASAIFIMELLYFYINVFFNSL

>DmelIR21a

MSYYWVALVLFTAQAFSIEGDRSASYQEKCISRRLINHYQLNKEIFGVGMCDGNNENEFRQKRRIVPTFQGN
PRPRGELLASKFHVNSYNFEQTNSLVGLVNKIAQEYLNKCPPVIYYDSFVEKSDGLILENLFKTIPITFYHGEIN
ADYEAKNKRFTSHIDCNCKSYILFLSDPLMTRKILGPQTESRVVLVSRSTQWRLRDFLSSELSSNIVNLLVIGES
LMADPMRERPYVLYTHKLYADGLGSNTPVVLTSWIKGALSRPHINLFPSKFQFGFAGHRFQISAANQPPFIFRI
RTLDSSGMGQLRWDGVEFRLLTMISKRLNFSIDITETPTRSNTRGVVDTIQEQIIERTVDIGMSGIYITQERLMD
SAMSVGHSPDCAAFITLASKALPKYRAIMGPFQWPVWVALICVYLGGIFPIVFTDRLTLSHLMGNWGEVENM
FWYVFGMFTNAFSFTGKYSWSNTRKNSTRLLIGAYWLFTIIITSCYTGSIIAFVTLPAFPDTVDSVLDLLGLFFR
VGTLNNGGWETWFQNSTHIPTSRLYKKMEFVGSVDEGIGNVTQSFFWNYAFLGSKAQLEYLVQSNFSDENIS
RRSALHLSEECFALFQIGFLFPRESVYKIKIDSMILLAQQSGLIAKINNEVSWVMQRSSSGRLLQASSSNSLREII
QEERQLTTADTEGMFLLMALGYFLGATALVSEIVGGITNKCRQIIKRSRKSAASSWSSASSGSMLRTNAEQLS
HDKRKANRREAAEVAQKMSFGMRELNLTRATLREIYGSYGAPETDHGQLDIVHTEFPNSSAKLNNIEDEESR
EALESLQRLDEFMDQMDNDGNPSSHTFRIDN

>DmelIR25a

MILMNPKTSKILWLLGFLSLLSSFSLEIAAQTTQNINVLFINEVDNEPAAKAVEVVLTYLKKNIRYGLS

QLDSIEANKSDAKVLLEAICNKYATSIEKKQTPHLILDTTKSGIASETVKSFTQALGLPTISASYGQQG

LRQWRDLDEAKQKYLLQVMPPADIIPEAIRSIVIHMNITNAAILYDDSFVMDHKYKSLLQNIQTRHVIAIAKD
GKREREEQIEKLRNLDINNFFILGTLQSIRMVLESVKPAYFERNFAWHAITQNEGEISSQRDNATMFMKPMAY
TQYRDRLGLLRTTYNLNEEPQLSSAFYFDLALRSFLTIKEMLQSGAWPKDMEYLNCDDFQGGNTPQRNLDL
RDYFTKITEPTSYGTFDLVTQSTQPFNGHSFMKFEMDINVLQIRGGSSVNSKSIGKWISGLNSELIVKDEEQMK
NLTADTVYRIFTVVQAPFIMRDETAPKGYKGYCIDLINEIAAIVHFDYTIQEVEDGKFGNMDENGQWNGIVK
KLMDKQADIGLGSMSVMAEREIVIDFTVPYYDLVGITIMMQRPSSPSSLFKFLTVLETNVWLCILAAYFFTSFL
MWIFDRWSPYSYQNNREKYKDDEEKREFNLKECLWFCMTSLTPQGGGEAPKNLSGRLVAATWWLFGFIIIA
SYTANLAAFLTVSRLDTPVESLDDLAKQYKILYAPLNGSSAMTYFERMSNIEQMFYEIWKDLSLNDSLTAVE
RSKLAVWDYPVSDKYTKMWQAMQEAKLPATLDEAVARVRNSTAATGFAFLGDATDIRYLQLTNCDLQVV
GEEFSRKPYAIAVQQGSHLKDQFNNAILTLLNKRQLEKLKEKWWKNDEALAKCDKPEDQSDGISIQNIGGVF
IVIFVGIGMACITLVFEYWWYRYRKNPRIIDVAEANAERSNAADHPGKLVDGVILGHSGEKFEKSKAALRPRF
NQYPATFKPRF

>DmelIR31a

MNLLISMFILILAAGEGEIIPSMEESVVTNFVKSLVKTKQAIVFSCLFKDFKEISLALMRINQFVSVVNL

NQSYSLTSILTRENYARTSVMVNARCSGSSELLFEASENRYFNKTYQWFLWGVDLEVQSLFPLNLNVGPNAQ
ITYVNETADGYAYWDIHSKGRHLKSNLEINLIATLINDTLNIARDIFHLQSIDFRGQFNGLTLRGSVIDKEDIISN
EQIESILSRPTKDAGVAAFIKYHYELLGLLRERFNFTVNFRNSRGWAGRLGNTTFRLGLLGIVMRNEADIAAS
GAFNRINRFAEFDTIHQSWKFETAFLYRYTSDLDTHGKSGNFLSPFSDRVWLFCLLTLGAFSIIWVLFEIIDYKI
LRIRVNSQKLEHLNQKSSVICIKTTCIERILQTFGACCQQGLDPNPVDRSVRFLVMTLFLFSLVMYNYYTSSVV
GGLLSSSDQGPSTVDEITASPLKISFEDIGYYKVLFRESQNRSITRLIEKKLSSSRSLNELPIFSHIEDAVPYLKAG
GFAFHCEVVDAYPVISEYFDANEICDLREVSGLMEVEILNWILHKNSQYTEIFKTAMCNAQEKGFVERILRRR
QIKKPACQSLYTVYPVSLSGVLPGFVILICGFGASLLLLCLEKVYAHFGPRKFCGF

>DmelIR40a

MHKFLALGLLPYLLGLLNSTRLTFIGNDESDTAIALTQIVRGLQQSSLAILALPSLALSDGVCQKERNVYLDDF
LQRLHRSNYKSVVFSQTELFFQHIEENLQGANECISLILDEPNQLLNSLHDRHLGHRLSLFIFYWGARWPPSSR
VIRFREPLRVVVVTRPRKKAFRIYYNQARPCSDSQLQLVNWYDGDNLGLQRIPLLPTALSVYANFKGRTFRV
PVFHSPPWFWVTYCNNSFEEDEEFNSLDSIEKRKVRVTGGRDHRLLMLLSKHMNFRFKYIEAPGRTQGSMRS
EDGKDSNDSFTGGIGLLQSGQADFFLGDVGLSWERRKAIEFSFFTLADSGAFATHAPRRLNEALAIMRPFKQD
IWPHLILTIIFSGPIFYGIIALPYIWRRRWANSDVEHLGELYIHMTYLKEITPRLLKLKPRTVLSAHQMPHQLFQ
KCIWFTLRLFLKQSCNELHNGYRAKFLTIVYWIAATYVLADVYSAQLTSQFARPAREPPINTLQRLQAAMIHD
GYRLYVEKESSSLEMLENGTELFRQLYALMRQQVINDPQGFFIDSVEAGIKLIAEGGEDKAVLGGRETLFFNV
QQYGSNNFQLSQKLYTRYSAVAVQIGCPFLGSLNNVLMQLFESGILDKMTAAEYAKQYQEVEATRIYKGSV
QAKNSEAYSRTESYDSTVISPLNLRMLQGAFIALGVGSLAAGVILLLEIVFIKLDQARLWMLCSRLQWIRYDR
KV

>DmelIR41a

MFIDLSWSLVLSAIVGKYLNESTICIFWNDKFEFQLLHKSDYISFVGINIKSFDDNGGHYIIDTGLKKKELQNK
HLFLDELVIKIIISIEVTHCETFVVFDKDIDRFVNAFNKASVYSIWRSLHNKFVFAHIANESPESRNHFFEDQPNI
LFVVRDHSSASSFDIKTNKFVGRKAENPSQMILVDRYLASEQRFQFGKSLFADKLNNLQGREVIIAGFDYPPY
TVIKHNMSTNAQDMGVSGESDFKNVYIDGTETRIVLNFCEQFNCTIQIDSSAANDWGKVYPNMSGDGALGM
LINRKADICIGAMYSWYEDYTYLDLSMYLVRSGITCLVPAPLRLTSWYLPLEPFKETLWAAILLCLCAEATGL
VLAYKSEQALYVLPGYREGWWTCTSFGVCTTFKLFISQSGNSKAYSLTVRVLLFACFLNDLIITSIYGGGLASI
LTIPSMDEAADTVTRLRFHRLQWAANSEAWVSAIRASDEALVKDILYNFHIYSDDELLRLAQDQHMRIGFTV
ERLPFGHFAIGNYLGPQAIDQLVIMKDDIYFQYTVAFVPRLWPLLDKLNTLIYSWHSSGFDKYWEYRVVADN
LNLKIQQQVQETMTGTKDIGPVPLGMSNFAGFIIVWILGSAIATLTFLLELSLTYILKQSNLK

>DmelIR47a

MRQIKLLVWLLVVGVVSSTEQLQFLKNFLEAVHKERSISTILLIQRKVHKNDFLHGLYPIFWPIICLDE

KRVELVNNFNKDFLALVYMESEADTLLLSALAADLNHIRDARIMIWLQMSPSENFLDRIVFQASKQFLNLVVI
ENTLKTRRFYPFPQPKVQVIDKPFEEKEIYPALWRNFMGKNAIAVPDLVPPRSFNSFDPKTGHRRESGSIYNVF
KAFTQRYNITMLLKWPLIRNTTQEEIIGKSVRGEIDLPITGQLISFRHPNGSRSQPLLGMTALSIAVPCGPELPMF
DRFFLFYGLATPITITGYYVLLNTIEIILGTLSDRIKRHPRRKKILNLVLNLRVFSCILSLPTPQGNRLRSVKGQLT
MVMSITGLILSCIVAAQTSTILTMKPQYRHIKNFQELSDSNITVVCNHLNYLTIKQQMDPKFMAKFMQNIWIV
NSIEQMKMIFDLNTSYAYQTFSYKKDPFTLLQMHTTRKAFCRTPGLDLVSGLAYTAVLEKNSIYALALQDYT
LKAFSAGLVYYWAEESIRDLISTVGRTQFEKLPIVIGYQSLKLQDYNVCWKILLIGGALAFCVFIVEVVVGLIN
RRI

>DmelIR48b

MILQQSSNLLKLLLLLAISSVRTQGLNDIIIELNQRLLISNNFLYCNQSDKLNEYEIKYLQHMPPISLMI

FTSIESMNFTQVEYNLGADNKLFLIMGNEEPPYDFLHALNLHFQFAEYIIVIDEPVDLKKSTKWLDFVNHLWQ
QGYVQLLIYTSYDEKLYHKIIFPETVIEETLVEQYISIRGSFNNLYGYPVRVAAYNNAPRSMLYVNRWGKHIF
AGFYMRFLRAFIDARNGSFVPVLTPSNSPGNCTLNLVNETVDVCADALAANPAAFSLTHGFRIASANVLVTH
AKPLHSYRYLTAPFQWSVWACLVIYVLLVVNFLSFIGWLRSGKWEFSKYLLEVFSSLLFSGFYLKEIRGRERY
ILFGVLFIAGFVYSTEYLGLLKSMLISEVFEKQIDTFEALVESNITLMVDPYDKILFAKYNMPEILSPIMELVSFE
TLLKHRNRFDQDYAYILFSDRMALYDYAQQFLKHPKLLRIPIDFSFLYTGIPMRKRWFLKHHLGRAWYWAF
ESGLTRKLALDADFEAVRVGYLSFLITEHVEAQPLNVDYFVMPAIALAIGYILALLSFVIEMTAWRIREFLGCR
KATMTSTGCSEGGHVDVD

>DmelIR48c

MSLLRIILIIIFLRIVSSIPDTIISHLSAELQIKIQIYFGLGNDLYDFSRLDGNYQKIIISHNISEEFKTYHDEPVLIIIRL
ERDLNLNLATLDVLRSYLTDRQYNDILLIDNDEENLNSYVDIRKAYWNAGFSQVLIYNSQQRTWSIKPYPYL
QIRPTSLKEYIENRNTRNLMGYPLRVLVTNDPPHCFVDKDELPGSPNRYKGSIVTMLKIFADQLNATFQANPF
REFRRYSTADCVQMVSDDEIDACGSIFIRTYTYATSQPVRLNRVVIMAPFGN

IEKFYYFFRPFDLYVWIGTGIIVVYIAVMGSLLHRWHFKEWNVGQYLLLAVQTLLNRELSLPQSSSGKFMLLL
LLFAIGFILSNLYVALLSMMLTTKLYQRPIENLADLKAANVNILLQTHNIRPNSVYGSSEELRERFLLVEESQH
LEKRNGLDPSYAYVDSEDRMDFYLYQQKFLRRRRMKKLSNPVGYTWAVQVIKQNWVLEKHYNDHVQRFF
ETGLQNKLVDDVHELAVKAGFLHFFPTQTQTIEPLRLEDIVMAAMVLGGGHALAVICFLVELFA

>DmelIR51b

MCKVLTLLVVILLLALTNAAYNVTLLKSVLSLISTREPWINTPIFVGHNTQGGDLNDLIIWLHQTMGVTSLTM
NLFLQPEHIRPLGHFKITRYNGIALFFCHDKHDIMWLTLDRNLRKLRRIRLIIILRNQRSGSQGAIKSIFNALWQ
YQFLNVLVLQRDQLYSYTPYPAMRFFKLDIHTEPLFPHAARNFHGYVVSTPAENDIPRVFHVHDPLTKSRKV
LGYAYRTFVEYLDHYNASLRLTNPDENLDPTTSVNMNHIVQLIIDGQLEISLHPYVFTPPTATKSYPLLIYPNC
LIVPMRNEIPRHMYLLRPFQLYSWYILLFAVFYITGILYCISPKLNKSSWPQRLGLNFLDAISKILFISPPITIYRPT
WRHLIIFLQLSVLGFMSTSWYNIELDSFFTTIVVGEQVNSMDQLVHQQQRVLVKEYEINTFLRHVEPRLVEKV
SRLLVPVNASEQVSALLSFNRSFAYPFTEERWQFFAMQQQYAFKPIFRFSSACLGSPHIGYPMRVDSHLETSL
NHFILKIQDTGLLNHWVVSDFNDAMRAGYVRFVDNVLGYQSIDVDTLRLGWCVLGIGWILSALVFSCEYWH
LYPWRFIA

>DmelIR52a

MALGWSVIILGFIGQLSAQILNYTQSRDLELLEGSLFRVLSRLNLEEEYNTLLIYGKECVFHSLLRKLEISAVTV
PSGSTDYDWSFSTAILILSCGYDAENEENSYTLMKLQRTRRLIYLEDNSEPESVCMRYSLKEQHNIAMVKSDF
DQSDTFYSCRLFQTPNYVEGHFFKDQPIYIENFQNMRGATIRTVADSLVPRTILYRDEKSGETKMMGYLGHMI
NTYAQKLNAKLHFIDTSKLGAKKPSVLDIMNWVNEDIVDIGTALASSLQFKNMDSVWYPYLLTGYCLMVPV
PAKMPYNLVYSMIVDPLVLSIIFVMLCLFSVLIIYTQHLSWKNLTLANILLNDKSLRGLLGQSFPFPPNPSKHLK
LIIFVLCFASVMITTMYEAYLQSYFTQPPSEPYIRSFRDIGNSSLKMAISRLEVNVLTSLNNSHFREISEDHLLIFD
DLSEYLVLRDSFNTSFIFPVSVDRWNGYEEQQKLFAEPAFYLATNLCFNQFMLFSPPLRRYLPHRHLFEDHM
MRQHEFGLVTFWKSQSFIEMVRLGLASMEDLSRKRNEEVSLLLDDISWILKLYLGAMFISSFCFILEILRCGER
CKRLWRCRW

>DmelIR52b

MTWLVILLCFLGYMAAHIADISVQNQSLMDNELINLLLKLRNEEFYDTLLVYGKDCEFHSVIKNVDVAVVLV
SDSMNFEWNFSSLTLILSCGPDIDNGGPNSTSIKLQRNRRLVLLKEDFQPSNICNIYTQKEQYNIALVRENFTKS
KSIYTCRYFQDPNVDEVNLSGTKPIFIEQFQNMKGKAIRIVPDLLPPRVMLYQDANDGELKMIGYVANLITNF
AQKVNATLQLDFLKPSTSITEISRMAKDDELDMGITLEASLNTSNLETSSYPYLLTSYCLMVQVPAKFPYNLV
YALIVDPLVLGIIFVLFLLLSVLLIYSQKMSWQDLSVANILLNDKSLRGLLGQSFPFPLNASKKLRLIFTILCFAS
IMLTTMYEAYLQSFFTNPPSEPEICSFQDVGSYNRRIAMSALEVNGLIKTNNSHFREIRMDDLEIFDNMPECYE
LRDAFNLSYNYVVTGDRWRSYAEQQTLFKEPVFYFARDLCFSRLIFLSVPLRRHLPYRHLFDEHMMQQHEFG
FVNYWMSHSFFDMVRLGLTSLKDLSRPLAYTPSLLMDDISWIMKIYLAAIVLCVFCFLLEIGVDKWKRWMK
FRNLQILNTC

>DmelIR52c

MVWLIIIILFCLGNSSSQILDVTNNSHLDFDYRLFGLLQRLQVEKSYDTLLVYGEDCAIPSLFERLQVPAVLVSS
GSTNFDWNFSSLTLILSCNFQDEREENYRTLMKLQTSRRLILLKGHIKPESVCDFYSKKEQHNVAMVKENFY
QLEVVYSCRLFQDQNYEKLNLFDGKSIYKDQFRNMHGAPIRTLSDKEPPRTIPYIDSKTGEEKFKGYVGMLIS
QFVKKVNATMQIREDLIKDDEEVSFVDITNFTSNDILDIGICEARTLEMSNYDAISYPYLMSSYCFMAPLPDSL
PFSDVYMAIVAPSILIMFLIIFCICSVLIIYIQERSYRSLTIRSVLMNDICLRGFLAQPFPFPRQYNRKLKLIFMLVC
FSSLISTTMYTAYLQAFLWGPPIEPRLTSFDDVKKSRYTMAINIYEREFLEALNVSLEDVEIYDYGKFSKLRSTF
NTNYLFPVTALQWFTINEEQKLFKYKIFYYCDAFCLNQFDILSIPLRRHLPYRDIFEEHMLLQKEFGLTKYWID
QSYRDMIRANLTTFKDFSPLLENDYIEVHNLYWVFTMYFVGMGMGLCFFILEILRPLRYWRNCKIKCEYCYA
FLKNFAK

>DmelIR52d

MVRIIIILLCLGYTKARILDATNTNHTDLEERLLSLLLRLQQEQFFNTLLIYGEDCAFSSLSRRLQVPTI

LVSSGSTSFEWNYSSLALILTCEFKAEREENYQTLKKLQMNRRLILLNGNIKPDSVCDFYSKKDQYNIAMVNN
NFHQVGIIYACRLFQERNYEKVYLSEGNPIYVDQFRNMQGALLKSITFNLIPGSMAYRDPKTGQEKHIGYVAN
LLNNFVEKVNATLDMQVKLHKAGKKTSFYNITKWASEDLVDIGMSYAAYFEMTNFDTISYPYLMTSTCFMV
PLPDMMPNSEIYMGIVDPPVLVVLIAIFCIFSVMLNYIKQRSWRSLSLVNVLLNDICLRGFLAQPFPFPRQSNRK
LKLISMLVCFFSVITTTMYTSYLQSFMWGPPIDPKMCSFADLENSRYKLAIRRYDIEMLRPFNVSMDHVVVFD
ESSQLEYLRDSFDDNYMYPMSALSWSAFKEQQKLFAFPLFYYSEKLCLKPISFFSFPIRRHLPYRDLFEEHMLQ
QNEFGLSTYWIDRSFSDMVRLKLATMNDFSPPRLEDYIEVSDLSWVFGMYFTGLGISCCCFGLELLGLPSWTR
RLRLTNWLRVRN

>DmelIR54a

MWTVITGIVLWAPVLVAGSAVDFIFRAAAEHSLSVIMIRIDYCPYNWAKDIFENQTIPVVVLSDSETFINIRMF
SRPLHVACLPGHELQKDLALLENFTSSLMDFPSQKKIVYISNNFSDPTRMDYIFETCYHRRIWNIVGLLASDEH
RYFYRYHLYPSFRTEYRSLESSTIFDKDFPNMHGHPLTVMPDQWLPRSVLYVDRRTGKQILAGSVGRFFHVL
SWKLNATLQLSKKVTTGRFLNATALKELSESFSVDVPASLTIMERVEQLASTSYPMEVTHVCLMVPVARRIPI
KDIYFILSSASNMFLAIVIVSSYGLALNLLRNMTHRDVRLVDFVLNDKALRGILGQSFNLPLSRSFSTRLIFLML
GIVGLNVSSIFGAGLDTLMAHPPRQFQARSFAGLRRTKIPLVTTEEDFPTWMKLRVPMLVVNVSEYNHLRNG
RNTSNAYFASRLYWNLFSEQQKRFTRELFIYSTDDCLWSLALLSFQWPQNSLFTEPVSQLILEVNANGLYDF
WVGMHYYDMTAAGLSGLEDPSLQLKEREHPTSLRIVDFQWMWQAYGTFMVIAILVFLLEVSWHRITSLFVS
LVY

>DmelIR56a

MGSRFFIRNLILFGLLASSNMQIPFGELEKKFELDVDFLLGVTELVGHIQGLYSITVYADCIDIHPSIQQ

RIMDKFMVPVNTIGSNLSRPNYHKLDNSRIRIVLFTGLNDTILVNLNKTDVPYSDNFYMLAYASAIKNKCIEL
DFIEEVFTLLWKMSIQNAILLIRGEFMMEMWSYLYMGKIHKIKLTKPNSYLESLRKYNYRFSLEVINDPPAIF
WYNSSEQADVTGGGNLSVSGPLGLIIINFLRHLNVTIDIVPIPGKQTSQYELFQQPDNLRAENGVNMVGSALL
KYSPMVTQSRMCLLVSNRRMIPFSRFLDRLVSPGVHKLTFVSSIGIFVIKYFSHRPRSFVDAIFCTIRFFFAIPLPS
IILNRLPVVDRFIEVFIIIFVQILLSSNISITTSALTTGFWEPPIINVETMRASGLHILTEDPTILQAFKENILPSSLAD
LVILVDEDTYFHHVTTLNNSYVYVVQAHNWQIFRLYQQQMTNEPFEIASEELCSKWRILGIPLNPKSPLRFMF
KDYFYRILESGLREQWVHSGFKKFCEFNNLKKLPVDSVDSWQPLSIEFYSNVIRAYIIGLVIATLAFVAELLHN
GYRRKNVKKT

>DmelIR56b

MLLDTDLASGVIRSPYSFDIPHAFIFNETQFVVPKFCGPYMEIVKHFAEVYHYQLFLDSLESLPKKSVVEQDIIS
GKYNLSLHGVIIRPEETSDFFNATQHSYPLELMTNCVMVPLAPELPKWMYMVWPLGKYIWTCLFLGTFYVA
LLLRYVHWREPGNATRSYTRNVLHAMALLMFSANMNMSVKLKHASIRVIIFYTLLYIFGFILTNYHLSHMTA
FDMKPVFLRPIDTWSDLIHSRLRIVIHDSLLEELRWLPVYQALLASPSRSYAYVVTQDAWLFFNRQQKVLIQP
YFHLSKVCFGGLFNALPMASNASFADSLNKFILNVWQAGLWNYWEELAFRYAEQAGYAKVFLDTYPVEPL
NLEFFTTAWIVLSAGIPISSLAFCLELFIHRRKQRRPQYERFECYDY

>DmelIR56c

MQHLLNLLAPFGRMNVFQEIVWFVSPHQRLDQLDEFIMRIDEAFGKSATQTVVNNNTEMRMIYSSARRNHM
SFVFTTGAEDPIMKVFSKVLLGRHFYVSMVIYVDKVGDMHPIYDLLTFAYNQQFFNSMVHFESMEGVNQLF
GVSKFPVMSFENRTDFLKYMGKIWKQVQNARSDVGGFGFTTPLRQDLPHLFQSQGHYDGSTYRIIETFVRFIN
GSFKELIMPPDSLGGQVINMKDALQLIRERKMEFCAHAYALFMSDEELEKSYPLLVVQWCLMVPLYNSVST
YFYPLQPFDWNVWFFALGALLALVLLELMWLRMFGGWSGYRGAVLNSFCYIINVPIEGQLQQPCLLRFLLL
ATVFFHGFFLSAYYTSNLGSILTVNLFHAQINTMNDIVSAQLPVMIIDYEMEFLLNLNKELPQEFLELLRPVDS
AVFSEHQTSFNSSFAYFVTEDHWEFLDEQQKHLKQRLFKLSSICFGSYHLAFPLQMDSSLWRDIEYFTFRIHSS
GLLNFYARSSFGSALHAGLVQRMPDTQEYTSAGLQHLAIAFILLLVMSFLAGIVFVLETLSR

>DmelIR56d

MDNRAAELILRERNIFPTNGSDNITLLNNMFVLEMFYRITQLYHFKNFIFYISERLDLNNKDSQEFFHNFWTYF
PMAPNLIITREHHLGIPMMQFISTPSLVMVFTTGKDDPIMELASHNQQGIHWLKTIFVLFPSLQSRDFETNPESL
AQFTAEIKDVYDWVWRKQFINTFLITIKDNVFILDPYPTPSIVNKTGVWQAEEFFHKYAKNMKGYLVRTPILY
DMPRVFKSDRPTNRYEKNFIHGTSGNLFLGFLEFVNATLMDTSANVTADYLNMTNLLDLVSQGVYETLIHSF
TEITTKFVVSYSYPIGINDCCIMVPYRNQSPADQYMHEALQENVWVLISLFTLYITVAIYLCSPLRPRDLSAAFL
QSICTLTYSVPTFIIRTPTLRMRYLYILLAIWGIVTSNLYISRMTSYFTTAPPVRQINTVQDVVEANLRIKMLAIE
YERMAKSPLQYPESYLNQVDLVDKHMLDLHRDPFNTSFGYTVSSDRWRFLNLQQLHLRKPIFRLTEICEGPF
YHVFPLHKDSHMRSVMTEYIMIAQQAGLMNHWERETFWEAVHLHRIHVHLFDDEPMALSLDFFSSLLRTWT
LGLILAGLAFAAEMKWHEHVTFKRRPVIRITRKPRSFLRRFMKL

>DmelIR60a

MWCNNPGLIIIIFLGQILNLCQGIVNLSNETANTVIFMLPEKDLGPDVWKAGVGCLDSFAQIFFFRNPKERFTR
AYNLMLVHAFHLSSPADQIQEGFSKLINEAVTNPGPPDREELFQMRVASDYNITNGTEDKGELILADNYVIVV
DSVDRLKELMKKKIVEMRSWNPGARFLVLFHNATCRNRPLGVASNIFKDLMEMFYVHRVALLYANSTMNY
NLLVNDYYSNVNCRILNVQSVGQCHDGKLYPNNAVVKASMQDYVSGFSPRNCTFFACSSISAPFVEADCILG
LEMRILGFMKNRLKFDVNQTCSLESRGEMDGPANWTGLLGKVQNNECDFVFGGYYPDNEVADHFWGSDT
YLQDAHTWYIKMADRRPAWQALVGIFEAYTWIGFILILIISWLFWFTLVMILPEPKYYQQLSLTAINALAVTIS
IAVQERPICETTRLFFMALTLYGLNVVATYTSKMIATFQDPGYLHQLDELTEVVAAGIPFGGHEESRDWFEND
DDMWIFNGYNISPEFIPQSKNLEAVKWGQRCILSNRMYTMQSPLADVIYAFPNNVFSSPVQMIMKAGFPFLFE
MNSIIRLMRDVGIFQKIDADFRYNNTYLNRINKMRPQFPETAIVLTTEHLKGPFFILVVGSCWAALTFIGELIIH
RWRTQLVSTSEQQDRRSDKRRRRRRRRKPEKDNRWQRQVQVAPVVRFTPVKRRKVFQGQTSQK

>DmelIR60b

MRRSLYLIIAIGLVDVHCVSLRYILNALENELQYRAILLVESASEIESCWEQKYIQGAVPILNFNANQSLYLKD
ALNTNILALVCLNENVESTMQALYENLEDMRDTPTILFVLSDSKVQDVFLECLRRKMLNVLAFKGLDRGFV
YSFRAFPTFRVIERNVMDILQYFEQQLEDLGGHTLTTLPDNIIPRTVVYKSPDGSRQLAGYLYPFLRNYVSTIN
ATLKVCWHLVPEDGMIQLGEVVRLSEIHDVDFPLGMHGIEHGSTSQNVPLEVSSWFLMLPMEPSLSRAQFFI
MLGFEKVTPVLLLLTILLSTAHRIEMGLRPSWRCYVLGDRVLQGTLGQAFFLPRRLSVKLMLVYSLILLNGFT
FSNYSITSLETWLVHPPSGHPIHSWEQMRTLNLKVLIVPSELDSMTKALGKQFTESNSDLFELSKSGNFQDKRL

AMDQSYAYPVTCTLWPLLEHAQIRLPKPEFRRSREMVLIPLLIMAMPLPKNSMFHKSLNRYRALTHQSGLYE
FWFKRSFNELVALRKIHYKVNGDHQIYRDFEWQDFSYVWLGFVGGTIASILVLLAEIGYHRWQLNQN

>DmelIR60d

MRLAIYVAFLSSIGNRSGFLSSLLMSLGKELHYKTILLVGGSSTCWSLEPFETGVPILNLRGENNAYPQDTFNS
QMLALACLQTESEDAVKLLYRSLKDMRDTPTLLFASSEEHIHDTLFLGCFRENMLNVLALTASSKEFIYSYQA
FPTFRVIKRKLVEIHRYFEPQLKDLGGHIVSALPGNIMPRTMCYRNAEGERQLAGYLNTFIRNYVESINGTLRI
SWGLVPEDDMRHLTISRLSKIQHVDFPLGIIPLYNKTDKQHVYMEISSWFLMLPMETSVPRAHLFVKLGLERL
LPIIVVVGAVLGNAHRIEVGLGPSWRCYYLADKVLRGALAQPIVLPRRLSPKLMLIYSLLLLSGFFLSNYYMA
SLTTWLVHPPASDRILEWDQLRYLHLKVLTIPEEFKYMSLILGTDFMTAYGSIFQLTNSTDFQRRRISMDPSYA
YPVTTSLWPFLELSQVRLRRPLFRRSYDMVLQPFQVMSLPLPRNSIFHKSLLRYAALTRETGLYYYWFRRSYY
ELVALGKISYKEEEGNPYCDLKWNDFRIVWLAFLGGTIISCLALLLEVAHYRWHLGNSSL

>DmelIR62a

MYLQFLFALFLSRYQIVATENFDRAFELALFLDRIGRVHRLHAITIVNSLGSVDPSYLDDLHRGLMCNSSNHF
YMLPQMTATDKDSSHVHFSSLQDEETIYLVFARDSKDAVIYLQAERARGRRYTRTMFLLRKQESQKDIKYFF
ELLWKLQFRSALVVVAARNFYQMDPYPTVRVIRMRRLSSYDPHHVFPPANRKNFRGYRMRLPVQQDVPNTF
WYKNRRTKAWELAGLGGILINQLMMHLNVTMDLFRFEVNGSSLLNMAALTDLIVKGKVELSPHLYDTLQS
NTSVDYSYPTQVAPRCFMIPLDNEISRSLYVFLPFSLTMWLCLLFVLLVVHFVYVRRLIPDGHFWAILGVPGA
GQVRYGNRKPVRRFSTFLILFGIFILGQTYSTKLTSSLTVTLIRRPDNSLEELFLLPYRILVLPTDVYAIVDSLGH
AEQFSTKFSCTDAENFSQKRISMHPEYIYPISTIRWRFFDMQQRFLRKKRFYFSKICHGSFPYQYQLRVDSHLK
DALHRFLLHVQQAGLHDLWLDTCYRKAHRMGYLKDFSTLAELEEKLRLRPLALNLLVPAFSLFLCGMLGSG
IAFLVEIRHSFGCRQKPPSINRNPGD

>DmelIR64a

MHWWLLVFLPLSCQGLPEHELLELELDYGLAEPQRTSLLQSSLILQFSQDYKHIPRITYFTCQKPHLQTPNQIP
NAAEHRDAFAAKNFQLIKSLYESELFVRIVLLDVLAQSPTSGRPNRPGNGPTGGFSQTPSQAQSNSEWLEGVL
RMEALRQIAVVDLACGAVSRRFLELASAKMLYSEKFHWLLIEDFAWHGRTQTAEGSGKRDDGEMEEEEPPG
QQIQATDDEDLPSIESFLGGMNLYMNTELTLAKRMSEAAHYTLFDVWNPGLNYGGHVNLTEIGSFTPTEGIQ
LHTWFRTTSTVRRRMDMQHARVRCMVVVTNKNMTGTLMYYLTHTMSGHIDTMNRFNFNLLMAVRDMFN
WTFVLSRTTSWGYVKNGRFDGMIGALIRNETDIGGAPIFYWLERHKWIDVAGRSWSSRPCFIFRHPRSTQKD
RIVFLQPFTNDVWILIVGCGVLTVFILWFLTTIEWKLVPHDGSALIKPKGGAPPRHHYQQQQQQEQVEAPVRP
ITAVSVVVSKEKVEEKQEEYEDSTPIDAGTLWQRCYQKLNKYIKDRKAKQKKAPERVGLFLESVLFFVGIICQ
QGLGFSTSFVSGRCIVITSLLFSFCIYQFYSASIVGTLLMEKPKTIKTLSDLVHSSLKVGMEDILYNRDYFLHTK
DPVSMELYAKKITSVPTTKENEADEDEPVDPNPVSTDPAKSYRDIVHSHETGAHAKDNAASNWLDPETGLLR
HLGFAFHVDVAAAYKIIAETFSEQDICDLTEVSMFPPQKTVSIMQKNSPMRKVISYGLRRVTETGILTYHFNV
WHSRKPPCVKKIETSDLHVDMDTVSSALLILLFSYAITLMILGTEILYSKWHNRIQLKWVGAT

>DmelIR67b

MELLYLNTLQSLSLLEGNRLVQTVQELNNIYQTELNVFLEFGNGADILESAQGTFVPTLWIKNPQNQKVMKG
NFTSCTLTILYLEDEHLDRGLYYLANWLWEYHHLEVLIFFNGGSYDKLIQIFSRCFNEGFVNVLVMLPGSDEL
YTFMPYQDLKILNLKSIKEFYSLSRKKMDLNGYNITSGLVIAGAPRWFSFRDRQNRLILTGYMLRMIVDFTNH
FNGSVRLMNVLTVNDGLELLANRTIDFFPFLIRPLKSFSMSNILYLENCGLIVPTSRPLPNWVYLLRPYAFDTW
IAWLIMLIYCSLALRILSKGQISISAAFLKVLRLVMYLSGSRDMGTRPTTRRLFLFVILTTSGFILTNLYVAQLSS
NSAAGLYEKQINTWEDLDKSDSIWPLIDVDIKTMEKLIPDRTKLLKKIVPTLEADVDTYRRNLNTSCIHSGFFD
RIDFALYQQKFLRFPIFRKFPHLLYQQPLQISAAFGRPYLQLFNWFVRKIFESGIYLKMKDDAYRHGIQSGLLN
LAFRDRHLEVKSNDVEYYYLIAGLWFGGLTLATVCFLLELLIGYAKIKVTISCKMNIM

>DmelIR67c

MFCWLIFLNIILLSDRSESWSAREVIHQFNHDQQLQLNIYLDCNDVELQIGQEVSNLFVNSTADKMKILGRFSS
HSLIIACFKDSTRNRTLNGVKELLWGLQYLPILFVVDSNMDFYFQQALRHGFIHVLALNFMNGSLYTYKPYP
KVEVHQIKDMQKFYKLTKLRNLQGQAVRTTVETMTPRCFRYRNRHGQLVYAGYMYRMVKEFISTYNGTEE
HVFGNVDTVPYKEGLAALKNGEIDMMPRIIHALEWYYFYRSHILYNIKTYIMVPWAEPLPKSLYFIQPFRGTV
WITIMVSFVYASIVIWWIRYRQQGNSSLTQSFMDVLQLLFQLPLSKIWHFNMGTHQVVSFIVLFVFGFMLTNL
YTAQLSSYLTTGLFKSQINTFDDLFREKRTLLVESFDAEVLHNMTKEKIIQKEFESIILITSIEEVFKHRKSLNTS
YAYEAYEDRIAFELSQQRYLRVPIFKILKEVYDQRPVFVALRHGLPYVELFNNYLRRIFESGIWIKLQEDSFLE
GIASGEISFRKSKSREIKIFDKDFYFFAYILLGMGWCVSTIALFLELWSFKYSVTNVLHEG

>DmelIR68a

MRCLWILIVAFISLAMATSIPIPIANPAPLSGYEMQLKILLQKILWVANVKRCFAVITDDLHYPIYDRIF

FESVGRRVIPFFVMRTNESDDLQRPSRQVELFVKAIKSSDCELNVITILNGWQVQRFLGYIYDNRSLNMQKKF
VLLHDLRLFESDMIHLWSVFIDAIFLKRQLDNKYTISTIAFPGILSGVLVMKNIANWELGKGLNGRILFADKTS
NLFGTSLPVAISEHVPMVLWANATKSFQGVEVEIMNALGKALNFKPVYYKPNQTENMDWTELDGGASVAY
GSGNPDGYAQNGTHIDSMLVDEVAAHSARFAIGDLHLFQVYLKLVELSAPHNFECLTFLTPESSTDNSWQTFI
LPFSAGMWVGVLLSLFVVGTVFYAISFLNAIINGNVSSEFFRCLRPNRNVPMDPKIYRRISFRIAISRYRSSKGD
RMPRDLFDGYTNCILLTYSMLLYVALPRMPRNWPLRVLTGWYWIYCILLVATYRASFTAILANPAARVTIDT
LEDLLRSHIPPSTGATENRQFFLEANDEVARKVGEKMEVFGYSDDLTSRIAKGQCAYYDNEFYLRYLRVADE
SGSALHIMKECVLYMPVVLAMEKNSALKPRVDASIQHLAEGGLIAKWLKDAIEHLPAEALAQQEALMNIQK
FWSSFVALLIGYVISMLTLLAERWHFKHIVMKHPMYDVYNPSLYYNFKRIYPQH

>DmelIR68b

MKFLVGLLLQWYLPGIYALAEIACRIAVEQNVQVTYLYRCASCPASFDADYSALELDLYRCVGSRLPVITRN
MEAHELEPFRRTDSLSIFQIPAAEKGDSLVRRILDMLNPHQRRKHMHKYLFVWPNAGRHQLLRLFRGSWAK
KLLYGLAITGRENGTFDFDPFAWGGLQVIQRLDGEVPYARKVKDLRGYPLRFSMFTDPLMAMPRSPVETAG
YQAVDGVAARVVGEMLNASVTYVFPEDNESYGRCLPNGNYTGVVSDIVGGHTHFAPNSRFVLDCIWPAVE
VLYPYTRRNLHLVVPASAIQPEYLIFVRVFRRTVWYLLLVTLLVVVLVFWVMQRLQRRIPRRGVIQFQATWY
EILEMFGKTHVGEPAGRLSSFSSMRTFLMGWILFSYVLSTIYFAKLESGFVRPSYEEQVDRVDDLVHLDVHIY
AVTTMYDAVRSALTEHQYGLLENRSRQLPLGIATSYYQPVVRRRDRRAAFIMRDFHARDFLAITYDSQAERP
AYHIAREYLRSMICTYILPRGSPFLHRLESLYSGFLEHGFFEHWRQMDLITRVGASPDAEEFLEDLGDQTDTDS
GSNELAIRNKKVVLTLDILQGAFYLWSVGIGISCLGFAVEHAHWFWRRQTLRNAVEARTS

>DmelIR75a

MQLVQLANFVLDNLVQSRIGFIVLFHCWQSDESLKFAQQFMKPIHPILVYHQFVQMRGVLNWSHLELSYMG
HTQPTLAIYVDIKCDQTQDLLEEASREQIYNQHYHWLLVGNQSKLEFYDLFGLFNISIDADVSYVKEQIQDNN
DSVAYAVHDVYNNGKIIGGQLNVTGSHEMSCDPFVCRRTRHLSSLQKRSKYGNREQLTDVVLRVATVVTQR
PLTLSDDELIRFLSQENDTHIDSLARFGFHLTLILRDLLHCKMKFIFSDSWSKSDVVGGSVGAVVDQTADLTA
TPSLATEGRLKYLSAIIETGFFRSVCIFRTPHNAGLRGDVFLQPFSPLVWYLFGGVLSLIGVLLWITFYMECKR
MQKRWRLDYLPSLLSTFLISFGAACIQSSSLIPRSAGGRLIYFALFLISFIMYNYYTSVVVSSLLSSPVKSKIKTM
RQLAESSLTVGLEPLPFTKSYLNYSRLPEIHLFIKRKIESQTQNPELWLPAEQGVLRVRDNPGYVYVFETSSGY
AYVERYFTAQEICDLNEVLFRPEQLFYTHLHRNSTYKELFRLRFLRILETGVYRKQRSYWVHMKLHCVAQNF
VITVGMEYVAPLLLMLICADILVVVILLVELAWKRFFTRHLTFHP

>DmelIR75b

MLQLHNLILHNLIHMAKLSHVLILHCSLSHLALLAQSKNIFTQFQPLHSDIQLNDDFLNHNILKLGVFLDINCD
KSGTVLDMASAKRFFSHRYHWLIYDRSMNFSVLESHFKEAQIFVDADVTYVTHDPFSKNFLLYDVYNKGRQ
LGGELNITADREIFCNKTNCRVERYLSELYTRSALQHRKSFTGLTMRATAVVTALPLNVSIKEIFDFMNSKYRI
QLDTYARLGYQARQPLRDMLDCKFKYIFRDRWSDGNATGGMIGDLILDKADLAIAPFIYSFDRALFLQPITKF
SVFREICMFRNPRSVSAGLSATEFLQPFSGGVWLTFALLLLLAGCLLWVTFILERRKQWKPSLLTSCLLSFGA
GCIQGAWLTPRSMGGRMAFFALMVTSYLMYNYYTSIVVSKLLGQPIKSNIRTLQQLADSNLDVGIEPTVYTRI
YVETSEEPDVRDLYRKKVLGSKRSPDKIWIPTEAGVLSVRDQEGFVYITGVATGYEFVRKHFLAHQICELNEI
PLRDASHTHTVLAKRSPYAELIKLSELRMLETGVHFKHERSWMETKLHCYQHNHTVAVGLEYAAPLFIILLG
AIILCMGILGLEVIWHRHCTLH

>DmelIR75c

MTSWPLYRLIVFNLLEINLSNLMVFHCWSIKEAFPLVEMLNQNGIFSQYIDVQNPDNLANVHKEYLDSDLVR
LGVFLDLGCDKAELVTNQSSRARLYNQNLHWLLYDEAGNFTKLTQLFEGANLSLNADVTYVSREDEERFIL
HDVYNKGSHLGGKLNITVDQTLQCNRSHCQVKEYLSELHLRPRLQHRMDLSSVTFRLAALVSVLPINSSEEE
LLEFLNSDRDSHMDSISRIGNRLIMHTQEILGFKLHYIWCGTWSVQDAFGGAIGMLTNESAELCTTPFVPSWN
RLHYLHPMTEQAQFRAVCMFRTPHNAGIKAAVFLEPFMPSVWFAFAGLLIFAGVLLWMIFHLERHWMQRCL
DFIPSLLSSCLISFGAACIQGSYLMPKSAGGRLAFIAVMLTSFLMYNYYTSIVVSTLLGSPVRSNIRTIQQLADSS
LDVGFDTVPFTKTYLVSSPRPDIRSLYKQKVESKRDPNSVWLSPEEGVIRVRDQPGFVYTSEASFMYHFVEKH
YLPREISDLNEIILRPESAVYGMVHLNSTYRQLLTQLQVRMLETGITSKQSRFFSKTKLHTFSNSFVIQVGMEY
AAPLFISLLVAYFLALLILILEICWARYAKKKFSTIIPQNQ

>DmelIR75d

MKVQVAHWLPLIFFLLVSGTPRVAGSWRSEYSRQDPDPKTRWGNQLPDMLVAYYRHHGVHSLMLVVCHT
DIADFRLWKLWQHFNLNNFYVQVSTESSLRDLQHVDALDEHKDAPPPKSFHANNSTHWETSFLLPALPYKM
GILLLEFSSECALNLLRWSAASEHNYFTTNRFWLLLTEDPGDIDLLEDPEIFIPPDSELRVLHYENVGNFSCSLI
DLYKVAAWKPLKRTLVGHNIRNSRHVIHALQHFGSAITYRQDLEGIVFNSAIVIAFPDLFTNIEDLSLRHIDTIS
KVNHRLMLELANRLNMSYNTYQTVNYGWRQPNGSFDGLMGRFQRYELDLAQLAIFMRLDRIALVDFVAET
YRVRAGIMFRQPPLSAVANIFAMPFENDVWVSILMLLIITTVVLVLELFFSPHNHDMSYMDTLNFVWGAMCQ
QGFYVEVRNRSARIIVFTTFVAALFLFTSFSANIVALLQSPSDAIQSLSDLGQSPLEIGVQDTQYNKIYFTESTDP
VTKNLYHKKIASKGENIYMRPLLGMEKMRTGLFAYQVELQAGYQIVSDTFSEPEKCGLMELEPFQLPMLAIP
TRKNFPYKELIRRQLRWQREVSLVNREERKWIPQKPKCEGGVGGFVSIGITECRYALGIFGCGAAVSFVLFLF
EFIFRHFKQVYRIIKGYREVQR

>DmelIR76a

MENLLVESYYFSTVLSFFAQQFFADSHATCIFWHPAFDFRLETVHPMPLIIMDWHRWANRSDQDVYDYKIKE
DEFEGKGIPYNDWTLRLTVAIERSHCETFIAFQEQIPEFARYFYHASIYSIWRSLRNRFMFVYTKEFEDKKDSY
LSGYIFQDQPNILVITSQYLNSSTFEIKTNRFVGPRNFNKNPEPVEFYILQRFDAKGTKATWETQSAMSSKMRN
LKGREVVIGIFDYKPFMLLDYEKPPLYYDRFMNTTDVTIDGTDIQLMLIFCELYNCTIQVDTSEPYDWGDIYL
NASGYGLVGMILDRRNDYGVGGMYLWYEAYEYMDMTHFLGRSGVTCLVPAPNRLISWTLLLRPFQFVLW
MCVMLCLLLESLALGITRRWEHSSVAAGNSWISSLRFGCISTLKLFVNQSTNYVTSSYALRTVLVASYMIDIIL
TTVYSGGLAAILTLPTLEEAADSRQRLFDHKLIWTGTSQAWITTIDERSADPVLLGLMEHYRVYDANLISAFS
HTEQMGFVVERLQFGHLGNTELIENDALKRLKLMVDDIYFAFTVAFVPRLWPHLNAYNDFILAWHSSGFDK
FWEWKIAAEYMNAHRQNRIVASEKTNLDIGPVKLGIDNFIGLILLWCFGMICSLLTFLGELWRGQG

>DmelIR76b

MATGIELLVAAALCVACPPLNDSPPTNLIQMGENGTLSPVTELPMDVDASEAGFDADAPVETLETINRKK

PKLREMLDWIGGKHLRIATLEDFPLSYTEVLENGTRVGHGVSFQIIDFLKKKFNFTYEVVVPQDNIIGSP

SDFDRSLIEMVNSSTVDLAAAFIPSLSDQRSFVYYSTTTLDEGEWIMVMQRPRESASGSGLLAPFEFWVW

ILILVSLLAVGPIIYALIILRNRLTGDGQQTPYSLGHCAWFVYGALMKQGSTLSPIADSTRLLFATWWIF

ITILTSFYTANLTAFLTLSKFTLPYNTVNDILTKNKHFVSMRGGGVEYAIRTTNESLSMLNRMIQNNYAV

FSDETNDTYNLQNYVEKNGYVFVRDRPAINIMLYRDYLYRKTVSFSDEKVHCPFAMAKEPFLKKKRTFAY

PIGSNLSQLFDPELLHLVESGIVKHLSKRNLPSAEICPQDLGGTERQLRNGDLMMTYYIMLAGFATALAV

FSTELMFRYVNSRQEANKWARHGIGRTPNGQSVAPSRWLRGWRRLNSGHGQLLGASTHGQNVTPPPPYQS

IFNGGSHGDPLNRWRRPLANGNALGNGVLLGGDSEGGVRRLINGRDYMVFRNPNGQSQLVPVRSPSAALF

QYSYTE

>DmelIR84a

MIKLQVKVISWPLIILTAFLRVLQIESINTNFLELAAFEDFLRSEHLSHVLVVRGDDADGDWKIECHQKL

LANYRVQFYRPEMSANFEDLMFYGSPRTAVLVLNSEHVLVRRQVFGVASEAGYFNNSLAWFILGSGRESL

PVEQLIDQLLSGYRMGIDADITVALRGPDNASMLFYDVYRISRQANTPLIIEKKGLWTHSGGYQKFGNFK

NTWVIRRRNFLNVTLIGSTVLTEKPPGFGDMEYLADDKQLQQLDPMQRKTYQLFQLVERMFNLSLAISLT

DKWGELLDNGSWSGVMGQVTSREADFAVCPIRFVLDRQPYVQYSAVLHTQNIHFLFRHPRRSHIKNIFFE

PLSNQVWWCVLALVTGSTILLLFHVRLERMLSNMENRFSFVWFTMLETYLQQGPANEIFRLFSTRLLISL

SCIFSFMLMQFYGAFIVGSLLSESARSIVNLQALYDSNLAIGMENISYNFPIFTNTSNQLVRDVYVKKIC

KSGEHNIMSLQQGAERIIQGRFAFHTAIDRMYRLLLELQMDEAEFCDLQEVMFNLPYDSGSVMPKGSPWR

EHLAHALLHFRATGLLQYNDKKWMVRRPDCSLFKTSQAEVDLEHFAPALFALALAMVASALVFLLELFLH

WLPDFRRRLGTMST

>DmelIR85a

MSIQWLKHILLLAILVNLAGTRENHIPLDLKKSSIVMVKMSQILCKARIKVLFVYFENQTSHEHTGQILK

EVTKCDISNQNTPLEAVKDDGILMYMVMITTNISQPLELSLIRKKSAAKHRSHVFLLVRDADTVSDAWMR

ASFRQFWKIWLLNIVILYWRDGRLNAYRYNPFMDNYLIPVDNKPNEVPTLEQLFPKTIPNMQRKPLRMCI

YKDDVRAIFWRQGTILGTDGLLAAYVAERLNATMMITRPHSYNNHNLSSDICFLEVAKEYVDVAMNIRFL

VPDTFRKQAESTVSHTRDDLCVIVPKAKTAPTFWNIFRSFGSLVWALILVSVLVANVFCYILKSEVGRVP

MQLFAGALTMPMTQIPPNHSIRLFLIFWLYFGLLICSAFKGNLTSMMVFQPYLPDINQLGALARSHYHII

IRPRHVKHIQHFLTLGHKHESRIREQMLEVSDTQMYEMMRNNDIRFAYLEKYHIARFQVNSRVHMHLGRP

LFHLMNSCLVPFHAVYIVPYGSPYLGFLDSLIRSSHEFGFERYWDRIMNSAFIKSGVKVVNRRRGSGNDE

PVVLKLQHFHAVFALWLVGIGMACIVLAWEHLTHNYNLAVTKRRD

>DmelIR87a

MSTPEQRFWLAALLFLLSQHSEVRGFGINLMKVQTEDKGQEACILALLRKYFDSGDGLSGSVLCINRNYQ

LPNIEEQLLRGVNNYENYPWSLLITNSREGPSPAKFLMNEKPQCYFLIVDNLEDEDLDEVFEHWKGMVNW

NPLAQFVVYLASLEETDEEMNDLMVELLLTFINKKIFNVNVIGQSEENQFYYGKTVFPYHPDNNCGNRVI

SVELLDACDYPSEETDSEDENDEDEGDGAQEEDDGPQEEGDGEQEEEDGPQEQEDGDQAKGDEGQENDDG

GLENKVENEFRIGASDDDELENDLSSNSSEPEAIIEEFFRAKFEDKFPRDLSGCPLTASFRPWEPYIFRN

SEEQPVDDYYYGLQGDEDDYNDTSPNYGESDDESYADPGEDGDGAIPDTETQSGGKLKLSGIEYEMVQTI

AERLHVSIEMQGENSNLYHLFQQLIDGEIEMIVGGIDEDPSISQFVSSSIPYHQDELTWCVARAKRRHGF

FNFVATFNADAGFLIGIFVVTCSLVVWLAQRVSGFQLRNLNGYFPTCLRVLGILLNQAIPAQDFPITLRQ

LFALSFLMGFFFSNTYQSFLISTLTTPRSSYQIHTLQEIYSNKMTVMGTSEHVRHLNKDGEIFKYIREKF

QMCYNLVDCLNDAAQNEHIAVAVSRQHSFYNPRIQRDRLYCFDRRESLYVYLVTMLLPKKYHLLHQINPV

IQHIIESGHMQKWARDLDMRRMIHEEITRVREDPFKALTFDQFRGAIAFSGGLLLVASCVFAFELCYVKY

VYRTEKRERKTKKITKKVHNIKIQHD

>DmelIR92a

MLLQPLVMHLSQLLRIIVGQYFAEFPSILIVYNNSASTTPLQLEYLSALELVLRELSKPIRLQWINVAFL

KDLNDLEDQVMGALNSSVTEGFITILSQTHHFIHARYYATRNANVRLKDKRYLFLCEDESPAELLCMDIL

QFYPHHLMVRPGTETAPTGPTGPHPDPRRGGGASVSTKNKDDGEGGAGNKTTSPYRDINFELWTQKFVGA

VGNLDALLLDAFLPNETFANRVELYPNKLLNLQRRSLLVGSITYVPYTITNYVPAGQGDVDPIHPQWPNR

SLTFDGAEANVMKTFCQVHNCHLRVEAYGADNWGGIYDNESSDGMLGDIYEQRVEMAIGCIYNWYDGITE

TSHTIARSSVTILGPAPAPLPSWRTNIMPFNNRAWLVLISTLVICGTFLYFMKYVSYRLRYSGTQVKFHH

SRKLEKSMLDIFALFIQQPSAPLSFDRFAPRFFLATILCATITLENIYSGQLKSMLTFPFYSAPVDTIEK

WAQSGWKWSAPSIIWVHTVQSSDLETEQILARNFEVHDYSYLSNVSFMPNYGFGIERLSSGSLSVGDYVS

TEALENRIVLHDDLYFDYTRAVSIRGWILMPELNKHIRTCQETGLYFHWELEFIDKYMDKKKQEVLMDLA

NGHKVKGAPQALDVRNIAGALFVLAFGVAFAGCALVAELLIHRMDLSK

>DmelIR93a

MNPGEMRPSACLLLLAGLQLSILVPTEANDFSSFLSANASLAVVVDHEYMTVHGENILAHFEKILSDVIR

ENLRNGGINVKYFSWNAVRLKKDFLAAITVTDCENTWNFYKNTQETSILLIAITDSDCPRLPLNRALMVP

IVENGDEFPQLILDAKVQQILNWKTAVVFVDQTILEENALLVKSIVHESITNHITPISLILYEINDSLRG

QQKRVALRQALSQFAPKKHEEMRQQFLVISAFHEDIIEIAETLNMFHVGNQWMIFVLDMVARDFDAGTVT

INLDEGANIAFALNETDPNCQDSLNCTISEISLALVNAISKITVEEESIYGEISDEEWEAIRFTKQEKQA

EILEYMKEFLKTNAKCSSCARWRVETAITWGKSQENRKFRSTPQRDAKNRNFEFINIGYWTPVLGFVCQE

LAFPHIEHHFRNITMDILTVHNPPWQILTKNSNGVIVEHKGIVMEIVKELSRALNFSYYLHEASAWKEED

SLSTSAGGNESDELVGSMTFRIPYRVVEMVQGNQFFIAAVAATVEDPDQKPFNYTQPISVQKYSFITRKP

DEVSRIYLFTAPFTVETWFCLMGIILLTAPTLYAINRLAPLKEMRIVGLSTVKSCFWYIFGALLQQGGMY

LPTADSGRLVVGFWWIVVIVLVTTYCGNLVAFLTFPKFQPGVDYLNQLEDHKDIVQYGLRNGTFFERYVQ

STTREDFKHYLERAKIYGSAQEEDIEAVKRGERINIDWRINLQLIVQRHFEREKECHFALGRESFVDEQI

AMIVPAQSAYLHLVNRHIKSMFRMGFIERWHQMNLPSAGKCNGKSAQRQVTNHKVNMDDMQGCFLVLLL
G

FTLALLIVCGEFWYRRFRASRKRRQFTN

>DmelIR94a

MALPKQLKFINIFLVLLIIYGSSDGTENQHEIFLNRLLQAVHNERSVETLFLLHHSNLANCSLQDWNPPR

IPTIRSNELTVFNVEKTFNHNALALVCLMKNSYREILNTLAKSFDCMRQERIILMIHRKSDSKFIEDITH

EVKNLQFLHLIVLIVQEKYNGQVFASTLRLQSFPEPHFKRIRNVFAIQRIFYRPINFHGKVLNAIPNDIP

ILFVALNEMFTEYARRYNSTLRIQNRTIKEDIEITEDNYDIDMKIQLHNSQNFLHHMNIAMDIGSNSLII

LVPCATELRGLDIFKELGVRTLTWLALLFYIIFVLVEMLFVFISNRFNGRNFTMRYTNPLINLRAVRAIL

GQTSPISNRYSLSIQHFFVFMSLFGTLFGGFFDCKLRSFLTKRPYYSQIENFSELRKSGVTVVVDHTTRQ

FIEQEINANFFRDEVPNVRTTTIQELINHVYSYDRKFAFVANSIPWRTFREEMKSINQKILCDSKNLTIL

ENVPLTFSIRRNAIFSHHLRNFIINAADSGMITCWFKMAGKVIRKHIKTTLRESEQQPSHLPLSFDHFKW

LWAVLCIAYVMSFMVFVMEILWSKYQRRTRSVSIV

>DmelIR94b

MSLIFNLLFILILSQAVSQETEFLQLKYLNNIVRSMIKLHKMETLVIVKHHLDNNCSLQNWNAHGMGIIR

TNDQGKLIMKDTFNSRTLAIICIGQNSHITLLRNVFETFGKVQQKKIILWTQMELKEKFFQEISKKSRDL

KLLNLLVLKAVTKDKLLIYRLNPFPSPHFKRIENIWTPNDTLFMDTKFNFHGMTAVVKHDYNWTIQMGNI

RKFPISRIEDKEVIEFALKYNLTLQFFNDVERFDIELRKRIILKSNSTQPIDSGIPMVFSSLLIVVPCGN

YLSIQDVIKVSGIEKWIFYIILVYVIFVLIEITFLGVTILISRQSRHQMIPNTLVNLCAFRAILGLPFPE

TRRTSLSLRQLFLAIALFGMIFSIFINCKLSSMLTNPCPRPQVNNFEELKTSGLTVVMDHDAENFIEKEI

GVDFFNQYMPRKVTLTFTERAKLLFSLKGNHAFTLFSESFAIIESYQRSKGLRAHCTSEDLIVAERVPRI

YILENNSILDRPLRRFIRQMQESGITNHWLKNIPSSLEKNLMQITIPYDRERVHPLSIEHLTWLWCILIL
GYSISMIVFFVEMSLKRRKKNLENRAPNICIC

>DmelIR94c

MSKVFKLLVLPLIYLSLTKGSKNPQLKFLRELINVIEEGREIRTIMVIKHSRDEYCHLDQWNPRGSPILR
TNEMGSIRISGYFNDQAVILACMGENSDYGLLKSLANAMDNMRQERIILWSEREPTKMLMDYISQQADRY
NFAQIIIVTMNEDVDAVPSLHQLNPYPTPRFRQITNISNIRRTSFFGCGLSFQGKTAILKESVVSNIRFK
VWSPSGPIPLSELKDYEIVQFAVKYNLSLKLYDQNESKSDHFDIQLGPLFITKDFPTQMAFVSPNTACSL
IVIVPCSPKWRFMDVLHKLGVLKLIGCLLIAYAVFVLIETLILWLTHRISGREVRLTSLNQLLNPRAFRG
ILGLPFPEFRRSSISLRQLFLVISVFGLVYSNFVSCTLSALLTKPAQNPQVRNFKELRDSGLITIMDKYT
HSFIEKHIDPEFFDHVLPHYLILQKKEALRMIWNFNDSYSYVMYTTTWKSLNTVQKSFDERVFCESESLT
IAWNLPRMYVLGNNSVLKWMLSRYITYMPQTGIPDSWTEQLPKVLKLLYNVTSPRRIKEGAVPLSIQHLS
WIWHLLFIGESIATLVFIVEILLQKSNQHTSNMRERSSEDDDFV

>DmelIR94d

MGQLHLLLVALVLLSPGGDSFYHSLIHHLNRELKIEYVLLLGNFDTTWLDILWQLPVSVLQIKEHSRETY
SLLENPSHNVLTIAFVNDSPEDILEILYRNLRMLNTQPVLLVIRKSTIRVNSLLEWCWHHQLLKVVAIAQ
DFMESLIVYSYNPFPVLQFIERRLDNSTVIFEKRLENLHGYEVPIALGGSSPRLIVYRDLEGKLIFSGPV
GNFMKSFEQRYNCRLVQPYPFDESAISPARDLIASVQNGSVQIALGAIYPQVPYTGYSYPIELMSWCLMM
PVPEEVPHSQLYSMVFSPMAFGITIVAMVLISLTLSMALRLHGYRVSFSEYFLHDSCLRGVLSQSFYEVL
RAPALIKAMYLVICLLGLLITSWYNSYFSTFVTSAPRFPQLTSYESIRHSNIKIVIWKPEYEMLLFFSEN
MEKYSSIFQLQEDYKEFLHLRDSFDTRYGYMMPMEKWSLMKEQQRVFSSPLFSLQDDLCVFHTVPIVFPM
VKNSIFKEPFDRLILDVTATGLLSRWRDMSFTEMIKAGQLGLEDRGHPKEFRAMKVGDLIQIWRFVGWML
GLATIVFLLELICFWRHKMWQNMKYMFCRNKNI

>DmelIR94e

MDCPKWILSGLCLISLVSGATVIELLGTLKLELDFEYVLLMKNRNFSLSDQVWNGTSLTKDVMDEVQVPV
LQFNENVSYFLHNSISRRLVTLGFMSDANLDEHRGLLTALVANLRHMTTSRVIFLVQSKASTDFLYELFR
NCWRKKLLNVIVIFQDFETTSTFYSYSNFPILQIEERIYETSLQTLPIFPDRLRNLHGYEMPVILGGTAP
RMIAYRNKKGNVVYDGTVGHFMTAFQQKYNVKFVQPLQAKNPLDFAPSMQTVGAVRNETVEISISLTFPT
IPPFGFSYPYEQMNWCVMLPVEADVPPFEYYTRVFELAAFLLTLGTLVLISCLLASALSLHGYATNISEF
LLHDSCLRGVLGQSFVEVFRAPTLVRGIYLEICVLGILITAWYNSYFSSYVTSAPKQPPFRTYDDILASK
LKVVAWKPEYAELVGRLLEFRKYETMFLVEPDFNRYLALRDTLDTRYGYMITTNRWVLINEQQKVFSRPL
FQKRDDFCFFNNIPFGFPLHENSVFMEPVQKLIMELAETGLYYHWITTGFSELIDAGEMHFVDLSPHREF
RAMQIQDLQYVWYGYAFMVVLSSLVWLLENLAYTVKSKTIFPTHFMQRNKK

>DmelIR94f

MWQQVLLAETSNWFRSDVLQRFWTHLRVEIRFRTMLNYRLESCDCWFDNVLGSDNSTALLWNDQTYPHYL

RRRQDTDILVVSCLRFHQYQEVLLALSLMLDQMRSMPVVLQLCGDEDSMQELNSARLLLKHSQDLKMPNV
VLLSSTFFTSATLYSYEMFPEFNVQKLVYQAYLTLFPYKLGNLKGHPIRTVPDNSEPLTIVRKTLNGSIA
IDGLVWQFMIEFAKHINATLQLPIEPHPEKSIKLVQILDLVRNQTVDIAASLRPYSLNVQRSSTHIYGSP
MMVGNWCMMLPTERVIGSHEALTRLMKSPWTWLILLLFYSVHRFLAQKTRLRSSLIHLIKLLINLSLICF
LQAQLSAYFIGPQKVNHISNMQQVEESGLKIRGMRGEFMEYPIDMRSRYASSFLLHDLFFDLAQYRNSLN
TSYGYTVTSVKWELYKEAQRHFRRPLFRYSEEICVQKLSLFSLIQQSNCIYCYRSRIFILRMHEAGLIRL
WYRRSYYVMVTAGRFPIGDLSTVHRAQPIRWTEWQNVVLLHGVGLLFSVVVFVIELTVHYANVCLNNL
>DmelIR94g
MSTAVNSVHSKLVSLISRGQELTSIFFYAPAKEKCHLEDTISSATWGLPLVIWRTDRTVILNGFIGEGLL
VLACLPGFHWRALLGSLARSLKYLRQARILIELMQDRDEFLVSEVLQFCLSQDMINVNAIFDDFPETENL
SSFEAYPSFEVVNQTFTPDTQVSDLYPNKMLNLRGGVIRTMPDYSEPNTILYQDKEGNKEILGYLWDLLE
AYAHKHNAQLQVVNKYADDRPLNFIELLDAAQSGIIDVGASIQPMSMGSLSRMHEMSYPVNQASWCTMLP
VERQLHVSELLTRVIPYPTLALLLLLWIFYEVLRGRWRRHSRLQSIGWLVLATLVSSNYVGKLLNLFTDP
PSLPPVNSLAALMESPVRIISIRSEYSAIEFTQRTKYSAAFHLALHASILIGLRNAFNTSYGYTITSEKW
KIYEEQQKRSSKPVFRYSKDLCFYEMIPFGLVIPENSPHRAPLHSYTLLLRQAGLHDFWVNRGFSYMVKA
GKINFTAVGERYEAKTLTITDLRNVFIIYVSVLLISLILFTCELFVSWVNYWLGF
>DmelIR94h
MLSNISFSSAPELVDLYGLVLKFLVSSETTLFYFNPTGQKCSWETLPRTILSNHPQIIWFREETYPGLYK
RHSSNLFVMACLSSTSYDGQLQLLAESLTRYRSVRVLIEVQDKEGSFLASQILLLCQQHSMLNVVLYFSR
WTRTLNVFSYLAFPYFKLLKQRLSGSLRPKIFINQLKDLQGYKIRVQPDLSPPNSFSYRDRHGECQVGGF
LWRIVENFSKSLKGDTQVLYPTWAKAKVSAAEYMIQFTRNGSSDIGVTTTMITFKHEERYRDYSYPMYDI
SWCTMLPVEKPLSVEILFSHVLSPGSALLLILAFILFFLIVPQLIKCLGITFRGRLIGMASRIFALVMLC
SSSAQLLSLLMSPPLHTRIKSFDDLLTSGLKIFGIRSELYFLDGGFRAKYASAFHLTENPNELYDNRNYF
NTSWAYTITSVKWNVIEAQQRHFAHPVFRYSTDLCFSSETPWGLLIAPESFYREPLQHFTLKINQAGLIT
QWMTQSFHEMVRAGRMTIKDYSRTNLMKPLRIQDLRKCWVIFAVGLGTSTVVFTIELLLIYTNVFLNSL
>DmelIR100a
MATTLQLIMLALVGGTLGQANNTDHKQVLTSIVKQLEGGLELHLRTSEDGGNDLVQFLMQEKSSIIISAK
QEEVPSRAKIMRHHFFIFDGVHQMQEIRTSLFNTDGFYILALENNTIEDDVLLMEFAADVWLQHGHSRIY
YVQLSKKSVLLFNPFLQRLVVVQDSKTYSRIYKDLEGYHLRIYIFDSVYSSVIGDGENKVLSVTGADAKL
AKTVARQLNFTADFVWPDDEFFGGRLANGEYSGGVGRAHRGEVDIIFAGFFIKDYLTTHIQFSAAVYMDE
LCLYVKKAQRIPQSILPLFAVHMDVWLCFLLVGLLGALVWLILRAVNLILGIEGVPDGSRATRISYFGAA
RRIFVDTWVIWVRVNVGRFPPFHSERIFVASLCLVSVIFGALLESSLATVYIRPLYYRDVNTLRELDESG
QPIYIKHPAFKDDLFYGHNSEVYRRLDAKMMLVAEGEERLIEMVSKRGGFAGVTRSASLQLSDIRYVMTK
KVHKIPECPKNYHIAYVLPRPSPYLEEVNRIVLRLVAGGIVGLWTGEAKERAKWSIQRFPEYLAELDVGR

WKVLTLSDVQLAFYALTIGCLLSAIVCMAEILLGRQRRLHSPK

>DmelCG11155

MVRKKREIVIKENIQGRSYLKKICCSYIILSILVISNALPPVIRVGAIFTEDERESSIESAFKYAIYRIN

KEKTLLPNTQLVYDIEYVPRDDSFRTTKKVCSQLEAGVQAIFGPTDALLASHVQSICEAYDIPHIEGRID

LEYNSKEFSINLYPSHTLLTLAYRDIMVYLNWTKVAIIYEEDYGLFNLMHSSTETKAEMYIRQASPDSYR

QVLRAIRQKEIYKIIVDTNPSHIKSFFRSILQLQMNDHRYHYMFTTFDLETYDLEDFRYNSVNITAFRLV

DVDSKRYLEVINQMQKLQHNGLDTINGSPYIQTESALMFDSVYAFANGLHFLNLDNHQNFYIKNLSCTSD

QTWNDGISLYNQINAAITDGLTGTVQFVEGRRNIFKLDILKLKQEKIQKVGYWHPDDGVNISDPTAFYDS

NIANITLVVMTREERPYVMVKEDKNLTGNLRFEGFCIDLLKAIATQVGFQYKIELVPDNMYGVYIPETNS

WNGIVQELMERRADLAVASMTINYARESVIDFTKPFMNLGIGILFKVPTSQPTRLFSFMNPLAIEIWLYV

LAAYILVSFALFVMARFSPYEWKNPHPCYKETDIVENQFSISNSFWFITGTFLRQGSGLNPKATSTRIVG

GCWFFFCLIIISSYTANLAAFLTVERMISPIESASDLAEQTEISYGTLEGGSTMTFFRDSKIGIYQKMWR

YMENRKTAVFVKTYEDGIKRVMEGSYAFLMESTMLDYAVQRDCNLTQIGGLLDSKGYGIATPKGSPWRDK

ISLAILELQEKGIIQILYDKWWKNTGDVCNRDDKSKESKANALGVENIGGVFVVLLCGLALAVVVAIFEF

CWNSRKNLNTENQSLCSEMAEELRFAMHCHGSKSRHRPRKRSCLNCSSVPTYVPSNVSTSNVGVYYNYFN

>DmelCG3822

MRSSGVLVLPLLLLQLILNCRKAQSLPDIIKIGGLFHPADDHQELAFRQAVDRINADRSILPRSKLVAQI

ERISPFDSFHAGKRVCGLLNIGVAAIFGPQSSHTASHVQSICDNMEIPHLENRWDYRLRRESCLVNLYPH

PNTLSKAYVDIVRHWGWKTFTIIYENNDGIVRLQELLKAHGMTPFPITVRQLSDSGDYRPLLKQIKNSAE

AHIVLDCSTERIHEVLKQAQQIGMMSDYHSYLVTSLDLHTVNLDEFRYGGTNITGFRLINEKIVSDVVRQ

WSIDEKGLLRSANLTTVRSETALMYDAVHLFAKALHDLDTSQQIDIHPISCDGQSTWQHGFSLINYMKIV

EMKGLTNVIKFDHQGFRTDFMLDIVELTPAGIRKIGTWNSTLPDGINFTRTFSQKQQEIEANLKNKTLVV

TTILSNPYCMRKESAIPLSGNDQFEGYAVDLIHEISKSLGFNYKIQLVPDGSYGSLNKLTGEWNGMIREL

LEQRADLAIADLTITFEREQAVDFTTPFMNLGVSILYRKPIKQPPNLFSFLSPLSLDVWIYMATAYLGVS

VLLFILAKFTPYEWPAYTDAHGEKVESQFTLLNCMWFAIGSLMQQGCDFLPKALSTRMVAGIWWFFTLIM

ISSYTANLAAFLTVERMDSPIESAEDLAKQTRIKYGALKGGSTAAFFRDSKISTYQRMWSFMESARPSVF

TASNGEGVERVAKGKGSYAFLMESTSIEYVTERNCELTQVGGMLDTKSYGIATPPNSPYRTAINSVILKL

QEEGKLHILKTKWWKEKRGGGKCRVETSKSSSAANELGLANVGGVFVVLMGGMGVACVIAVCEFVWKSR
K

VAVEERLSAILNE

>DmelCG5621

MWFVKMISTEASFPLGFILTSLLLAFPGCRGERTNVGLVYENTDPDLEKIFHLAISKANEENEDLQLHGV

SVSIEPGNSFETSKKLCKMLRQNLVAVFGPTSNLAARHAMSICDAKELPFLDTRWDFGAQLPTINLHPHP

ATLGVALRDMVVALGWESFTIIYESGEYLPTVRELLQMYGTAGPTVTVRRYELDLNGNYRNVLRRIRNAD

DFSFVVVGSMATLPEFFKQAQQVGLVTSDYRYIIGNLDWHTMDLEPYQHAGTNITGLRLVSPDSEQVQEV
AKALYESEEPFQNVSCPLTNSMALVYDGVQLLAETYKHVNFRPVALSCNDDSAWDKGYTLVNYMKSLTLN
GLTGPIRFDYEGLRTDFKLEVIELAVSGMQKIGQWSGEDGFQENRPAPAHSLEPDMRSLVNKSFVVITAI
SEPYGMLKETSEKLEGNDQFEGFGIELIDELSKKLGFSYTWRLQEDNKYGGIDPKTGEWNGMLREIIDSR
ADMGITDLTMTSERESGVDFTIPFMSLGIGILFRKPMKEPPKLFSFMSPFSGEVWLWLGLAYMGVSISMF
VLGRLSPAEWDNPYPCIEEPTELENQFSFANCLWFSIGALLQQGSELAPKAYSTRAVAASWWFFTLILVS
SYTANLAAFLTVESLVTPINDADDLSKNKGGVNYGAKIGGATFNFFKESNYPTYQRMYEFMRDNPQYMTN
TNQEGVDRVENSNYAFLMESTTIEYITERRCTLTQVGALLDEKGYGIAMRKNWPYRDTLSQAVLEMQEQG
LLTKMKTKWWQEKRGGGACSEAPDSDASSLGFANLGGVYLVMFVGSCFGSIYGLVNCVVSVYLRARENKV
SFKTELLDEIRFILQCSGNTKAVKYPKNSSRSNASSKSKGSSMSVDSLPEDTSEADASGKHNHGKK
>DmelCG9935
MLIASGFLLFQFLSYGLGVPPLVRIGAIFSNQPGMYNSELAFRYAIHRLNMDKSLLPETTVDYYVEYVNR
FDSFETVQKVCKLIRVGVQAVFSPTDSVLATHINSICDALDIPNIGRSAHDFSINVYPSKQLVNYAFNDV
IQYLNWTRFGILHEKENGIINLHQLSRSFHGEVHMRQVSRDSYVSALNEFKGKEIHNIIIDTNSNGISIL
LKNILQQQMNEYKYHYLFTSFDLETYDLEDFKYNFVNITSFRLVDTADVGVKQILKDIGLYSHHIFKKPY
LNLHIKKSTILESEPALMFDSVYVFAIGLQTLEQSHSLTLLNISCEEENSWDGGLSLINYLNAVEWKGLT
GPIQFKDGQRVQFKLDLIKLKQHSIVKVGEWTPHGHLNITEPSMFFDAGSMNVTLVVITILETPYVMMHY
GKNFTGNERFYGFCVDILETISREVGFDYILDLVPDRKYGAKDPETGEWNGMVAQLMKYADLAVGSMTIT
YARESVIDFTKPFMNLGISILFKVPTSEPTRLFSFMNPLAIEIWIYVLIAYFLVSLCIYIVGKLSPIEWK
CINACDLENISIGNQFSLTDSFWFTIGTFMQQSPDIYPRAMSTRIISSTWGFFSLIIVASYTANLAAFLT
TERMINPIENAEDLASQTEISYGTLDSGSTMTFFRDSVIETYKKIWRSMDNKKPSAFTTTYEDGIKRVNQ
GNYAFLMESTMLDYIVQRDCNLTQIGGLLDTKGYGIATPKGSPWRDKISLAILELQERGDIQMLYDKWWK
NTDETCTRKNTSKQSKANSLGLESIGGVFVVLIAGIIVAAVVAFFEFWYNFRYNYEATPSQSVVNNKYNQ
DGILESERNYTPPDRSFWIEIAEELRYASWCMNKQKRPALTRTCSKCTIPKGQRINKL
>Dmelclumsy
MYSLFLTHFLLIALPVLADIDRSQFMVGSIFTSDKDESEIAFRTAVDRANILERNVELVPIVVYANTDDS
FIMEKMVCNLISQGVIAIFGPSTGSSSDIIASICDTLDIPHIVYDWIPNESIPDREHSTMTLNVHPDNLL
LSQGLAEIVQSFAWRSFTVVYETDKELQQLQDILQVGEPISNPTTVKQLGPGDDHRPFLKEIKLSTDNCL
ILHCAPDNLLKILQQANELKMLGEYQSVFIPLLDTHSIDFGELSGVEANITTVRLMDPSDFHVKNVVHDW
EEREKREGRYFKVDPNRVKSQMILLNDAVWLFSKGLTELGIFEELTAPDLECRRKKPWPFGKRIIEFIKA
RSEETSTGRIDFNENGQRSFFTLRFMELNSDGFLDLATWDPVNGLDVLNDDEESEKRVGQKLSNKTFIVS
SRLGAPFLTLREPQEGEILTGNSRYEGYSIDLINEIAKMLNFKFEFRMSPDGKYGALNKVTQTWDGIVRQ
LIDGNADLGICDLTMTSSRRQAVDFTPPFMTLGISILFSKPPTPPTDLFSFLSPFSLDVWIYMGSAYLFI
SLLLFALARMAPDDWENPHPCKEPEEVENIWSIMNTTWLSIGSLMGQGCDILPKAASTRLVTGMWWFFAL

MMLNSYTANLAAFLTNSRQANSINSAEDLAAQSKIKYGAMAGGSTMGFFRDSNFSTYQKMWTAMESASPS

VFTKTNDEGVERVQKGKNLYAFLMESTTLEYNVERKCDLVQIGGWLDYKSYGIAMPFNSPYRKQISAAVL

KLGELGQLAELKRKWWKEMHGGGNCEKSDEDGGDTPELGLENVGGVFLVLGLGLLSAMVLGCTEFLWNV
K

SVAIEEKISLKEAFKSEALFAARIWITTKPVHTSSESGSSNSSSSSSSRSKHSFKSQGLSMKSLKSSGYQ

DVEASVHSKLKKIGSMFSLKSQKTVTPPPEIGWKLDKSTQIDVVPTSDVDQELIPEVEPHLPHRHHHHHH

HRHHHHHHQPDQEHDRNPSPPE

>DmelGlu-R1

MHSRLKFLAYLHFICASSIFWPEFSSAQQQQQTVSLTEKIPLGAIFEQGTDDVQSAFKYAMLNHNLNVSS

RRFELQAYVDVINTADAFKLSRLICNQFSRGVYSMLGAVSPDSFDTLHSYSNTFQMPFVTPWFPEKVLAP

SSGLLDFAISMRPDYHQAIIDTIQYYGWQSIIYLYDSHDGLLRLQQIYQELKPGNETFRVQMVKRIANVT

MAIEFLHTLEDLGRFSKKRIVLDCPAEMAKEIIVQHVRDIKLGRRTYHYLLSGLVMDNHWPSDVVEFGAI

NITGFRIVDSNRRAVRDFHDSRKRLEPSGQSQSQNAGGPNSLPAISAQAALMYDAVFVLVEAFNRILRKK

PDQFRSNHLQRRSHGGSSSSSATGTNESSALLDCNTSKGWVTPWEQGEKISRVLRKVEIDGLSGEIRFDE

DGRRINYTLHVVEMSVNSTLQQVAEWRDDAGLLPLHSHNYASSSRSASASTGDYDRNHTYIVSSLLEEPY

LSLKQYTYGESLVGNDRFEGYCKDLADMLAAQLGIKYEIRLVQDGNYGAENQYAPGGWDGMVGELIRKEA

DIAISAMTITAERERVIDFSKPFMTLGISIMIKKPVKQTPGVFSFLNPLSQEIWISVILSYVGVSFVLYF

VTRFPPYEWRIVRRPQADSTAQQPPGIIGGATLSEPQAHVPPVPPNEFTMLNSFWYSLAAFMQQGCDITP

PSIAGRIAAAVWWFFTIILISSYTANLAAFLTVERMVAPIKTPEDLTMQTDVNYGTLLYGSTWEFFRRSQ

IGLHNKMWEYMNANQHHSVHTYDEGIRRVRQSKGKYALLVESPKNEYVNARPPCDTMKVGRNIDTKGFGV

ATPIGSPLRKRLNEAVLTLKENGELLRIRNKWWFDKTECNLDQETSTPNELSLSNVAGIYYILIGGLLLA

VIVAIMEFFCRNKTPQLKSPGSNGSAGGVPGMLASSTYQRDSLSDAIMHSQAKLAMQASSEYDERLVGVE

LASNVRYQYSM

>DmelGlu-R1B

MRFGLKLSCLWPSFLLWLTWSSGGGGGSGVGVSAQPSLTEKIPLGAIFEQGTDEVQSAFKYAMLNHNLNV

SSRRFELQAYVDVINTADAFKLSRLICNQFSRGVYSMLGAVSPDSFDTLHSYSNTFQMPFVTPWFPEKVL

TPSSGFLDFALSMRPDYHQAIIDTIQFYGWRKIIYLYDSHDGLLRLQQIYQGLRPGNESFQVELVKRISN

VSMAIEFLHTLEQIGRFENKHIVLDCPTEMAKQILIQHVRDLRLGRRTYHYLLSGLVMDDRWESEIIEFG

AINITGFRIVDTNRRLVREFYDSWKRLDPQMSVGAGRESISAQAALMYDAVFVLVEAFNKILRKKPDQFR

NNVQRRSQTLMVAQAAASTSSDGYNYSASGGGGGNGGAGGGFAGSDSGGSGGMASRALDCNTAKGWVN
AW

EHGDKISRYLRKVEIEGLTGDIKFNDDGRRVNYTLHVVEMTVNSAMVKVAEWNDDAGLQPLNAKYVRLRP

HVEFEKNRTYIVTTVLEEPYIMLKQVAFGEKLHGNNRFEGYCKDLADLLAKELGINYELRLVKDGNYGSE

KSSAHGGWDGMVGELVRKEADIAIAAMTITAERERVIDFSKPFMSLGISIMIKKPVKQTPGVFSFMNPLS

QEIWVSVIFSYIGVSIVLFFVSRFSPHEWRLVQQQPQQSQSPDPHAHHEQLANQQPPGIIGGAPLPAPPG

PPTPGAQTAAGAAALQAALSAGSPGSGGSSSAVVNEFSVWNSFWFSLAAFMQQGCDLSPRSVSGRIAAAS
WFFFTLILISSYTANLAAFLTVERMVTPINSPEDLAMQTEVQYGTLLHGSTWDFFRRSQIGLHNKMWEYM
NSRKHVFVPTYDEGIKRVRNSKGKYALLVESPKNEYVNAREPCDTMKVGRNLDTKGFGIATPLGSALKDP
INLAVLTLKENGELIKLRNKWWYEKAECSTHKDGETSHSELSLSNVAGIFYILIGGLLVSVFVAILEYCF
RSRDSRSASSGSGMGLGMGLGGGMSGGSLGKANGSMMLGPSSAVPGGMPSSHQRSTLTDTMHAKAKLTIQ
ASRDYDNGRVGYLNCASLQYYPPAQLSATPPDAGDSLHMNAHGQVXHEHAQESRLXRTPCAPTIRRIMAN
SRQETEKMMGGGHGGTVTVSVPATCSSSMNGSHEGSLALSPSSGSATLMRHSPDINQHPYGK
>DmelGluRIIA
MRLCPVVIYAFIIIIGFLEGIIALGGDDRNEITVGAIFYENEKEIELSFDQAFREVNNMKFSELRFVTIK
RYMPTNDSFLLQQITCELISNGVAAIFGPSSKAASDIVAQIANATGIPHIEYDLKLEATRQEQLNHQMSI
NVAPSLSVLSRAYFEIIKSNYEWRTFTLIYETPEGLARLQDLMNIQALNSDYVKLRNLADYADDYRILWK
ETDETFHEQRIILDCEPKTLKELLKVSIDFKLQGPFRNWFLTHLDTHNSGLRDIYNEDFKANITSVRLKV
VDANPFERKKTRLTKVDQILGNQTMLPILIYDAVVLFASSARNVIAAMQPFHPPNRHCGSSSPWMLGAFI
VNEMKTISEDDVEPHFKTENMKLDEYGQRIHFNLEIYKPTVNEPMMVWTPDNGIKKRLLNLELESAGTTQ
DFSEQRKVYTVVTHYEEPYFMMKEDHENFRGREKYEGYAVDLISKLSELMEFDYEFMIVNGNGKYNPETK
QWDGIIRKLIDHHAQIGVCDLTITQMRRSVVDFTVPFMQLGISILHYKSPPEPKNQFAFLEPFAVEVWIY
MIFAQLIMTLAFVFIARLSYREWLPPNPAIQDPDELENIWNVNNSTWLMVGSIMQQGCDILPRGPHMRIL
TGMWWFFALMMLSTYTANLAAFLTSNKWQSSIKSLQDLIEQDKVHFGSMRGGSTSLFFSESNDTDYQRAW
NQMKDFNPSAFTSTNKEGVARVRKEKGGYAFLMETTSLTYNIERNCDLTQIGEQIGEKHYGLAVPLGSDY
RTNLSVSILQLSERGELQKMKNKWWKNHNVTCDSYHEVDGDELSIIELGGVFLVLAGGVLIGVILGIFEF
LWNVQNVAVEERVTPWQAFKAELIFALKFWVRKKPMRISSSSDKSSSRRSSGSRRSSKEKSRSKTVS
>DmelGluRIIB
MHGLQFLVLLALAIASGANEDTLVIKIGAIFFDTEMKLADAFSAALEEVNAINPALKLDAIKRYVTVDDS
IVLQDISCDLIGSGVAAIFGPSSKTNSDIVEVLCNMTGIPHLQFDWHPQQSNRERMNHQLTVNVAPMELF
LSAAFSDILASKTFDWKSFTIAYERSSHLIRLQHILAWKQLHKAGIKMQEFERGDDYRILWKRINNAREK
FVLLDCPSDILVDVINASIGYNMTGSFNHLFLTNLDTHLSGIDGFYSRDFTVAVAAVRIRTYVPPPVHDE
IDVFDNSVDTRFSSLGSQLVYDSIVLFYNALLEISQRPGFYIPNFSCGRGFWQPGPRLVEQMKQITPKMV
KPPFKTQRLQINADGQREDFNLEVYNPIIDRVTHIWNKEFQLVDFEKLRENSTQALKQKRLQNKEDFSQK
PIRYTVATRVGKPYFSWREEPEGVHYEGNERFEGYAVDLIYMLAQECKFDFNFEPVRDNKYGSYDANTDE
WDGIIRQLIDNNAQIGICDLTITQARRSVVDFTVPFMQLGISILSYKEPPPKADIYAFLNPYNAEVWLFV
MIAMMITAFALIFTGRIDQYEWDQPVENVNREMERQNIWHLSNALWLVLGSMLNQGCDLLPRGLPMRLLT
AFWWIFALLISQTYIAKLAAFITSSKIAGDIGSLHDLVDQNKVQFGTIRGGATSVYFSESNDTDNRMAWN
KMLSFKPDAFTKNNEEGVDRVKLSKGTYAFLMETTNLQYYVQRNCELTQIGESFGEKHYGIAVPLNADFR
SNLSVGILRLSERGELFKLRNKWFNSNESTCDSNVPTIDDGQFDMDSVGGLFVVLIVGVVVGLVIGVAEF

LWHVQRISVKEKIPPMLALKAEFYFVIRFWLTRKPLHTYRQSRDSTSTGYSSLEQITSASSAKKKKKTRR
IEK
>DmelGluRIIC
MWQRILLLGCMWSAFFMCRSRGQQINIGAFFYDDELELEKEFMTVVNAINGPESEQTMRFYPLIKRLKPE
DGSVTMQEHACDLIDNGVAAIFGPSSKAASDIVALVCNSTGIPHIEFDISDEGIQAEKPNHQMTLNLYPA
QAILSKAYADIVQNFGWRKFTIVYDADDARAAARLQDLLQLREVHNDVVRVRKFHKDDDFRVMWKSIRGE
RRVVLDCEPNMLVELLNSSTEFGLTGQYNHIFLTNLETYTDHLEELAADNETFAVNITAARLLVNPDPPP
YSLPYGYVTQRDNIVYESSDPPRTLIHDLIHDALQLFAQSWRNASFFYPDRMVVPRITCDFAASGGRTWA
MGRYLARLMKGTSGVNNTNFRTSILQFDEDGQRITFNIEVYDPLDGIGIAIWDPRGQITQLNVDVKAQKK
MIYRVATRIGPPYFSYNETARELNLTGNALYQGYAVDLIDAIARHVGFEYVFVPVADQQYGKLDKETKQW
NGIIGEIINNDAHMGICDLTITQARKTAVDFTVPFMQLGVSILAYKSPHVEKTLDAYLAPFGGEVWIWIL
ISVFVMTFLKTIVARISKMDWENPHPCNRDPEVLENQWRIHNTGWLTVASIMTAGCDILPRSPQVRMFEA
TWWIFAIIIANSYTANLAAFLTSSKMEGSIANLKDLSAQKKVKFGTIYGGSTYNLLADSNETVYRLAFNL
MNNDDPSAYTKDNLEGVDRVRKNRGDYMFLMETTTLEYHREQNCDLRSVGEKFGEKHYAIAVPFGAEYRS
NLSVAILKLSERGELYDLKQKWWKNPNASCFEEPDPDATPDMTFEELRGIFYTLYAGILIAFLIGITEFL
VYVQQVALEERLTFKDAFKKEIRFVLCVWNNRKPIVAGTPISSVRTTPRRSLDKSLDRTPKSSRRVVIGR
SSEEMREMAQGSGSSSGSNNAGRGEKEARV
>DmelGluRIID
MHFCWISLIILSLSRVQAQFYGGNAYEASSGQSIRLGLITDDATDRIRQTFEHAISVVNNELGVPLVGET
EQVAYGNSVQAFAQLCRLMQSGVGAVFGPAARHTASHLLNACDSKDIPFIYPHLSWGSNPDGFNLHPSPE
DIANALYDIVNQFEWSRFIFCYESAEYLKILDHLMTRYGIKGPVIKVMRYDLNLNGNYKSVLRRIRKSED
SRIVVVGSTTGVAELLRQAQQVGIMNEDYTYIIGNLNLHTFDLEEYKYSEANITGIRMFSPDQEEVRDLM
EKLHQELGESEPVNSGSTFITMEMALTYDAVRVIAETTKHLPYQPQMLNCSERHDNVQPDGSTFRNYMRS
LEIKEKTITGRIYFEGNVRKGFTFDVIELQTSGLVKVGTWEEGKDFEFQRPPQAVNFNDIDDGSLVNKTF
IVLISVATKPYASLVESIDTLIGNNQFQGYGVDLIKELADKLGFNFTFRDGGNDYGSFNKTTNSTSGMLK
EIVEGRADLAITDLTITSEREEVIDFSIPFMNLGIAILYVKPQKAPPALFSFMDPFSSEVWLYLGIAYLG
VSLCFFIIGRLSPIEWDNPYPCIEEPEELENQFTINNSLWFTTGALLQQGSEIAPKALSTRTISAIWWFF
TLIMVSSYTANLAAFLTIENPTSPINSVKDLADNKDDVQYGAKRTGSTRNFFSTSEEPIYIKMNEYLNAH
PEMLMENNQQGVDKVKSGTKYAFLMESTSIEFNTVRECNLTKVGDPLDEKGYGIAMVKNWPYRDKFNKAL
LELQEQGVLARLKNKWWNEVGAGVCSAKSDDDGPSELGVDNLSGIYVVLVIGSIISIIISILCWCYFVYK
KAKNYEVPFCDALAEEFRIVIRFSENERPLKSAQSIYSRSRNSSQSIESLKTDSEENMPVED
>DmelGluRIIE
MFFNHFVILWSLFSIHISVNWAQYENFGGYDNYQSLESVPIGLLTDQNTEQMNIVFDHAIDVANQEVGTS
LTSLKEEVNYGDAYQSYGKLCRMLETGIAGVFGPSSRHTAVHLMSICDAMDIPHIYSYMSENAEGFNLHP

HPADLAKALYSLITEFNWTRFIFLYESAEYLNILNELTTMLGKSGTVITVLRYDMQLNGNYKQVLRRVRK

SVDNRIVVVGSSETMPEFLNQAQQVGIINEDYKYIIGNLDFHSFDLEEYKYSEANITGLRLFSPEKMAVK

ELLMKLGYPTDQDEFRNGSCPITVEMALTYDAVQLFAQTLKNLPFKPMPQNCSQRTESVRDDGSSFKNYM

RTLRLTDRLLTGPIYFEGNVRKGYHLDVIELQPSGIVKVGTWDEDRQYRPQRLAPTTAQFDSVDNSLANK

TFIILLSVPNKPYAQLVETYKQLEGNSQYEGYGVDLIKELADKLGFNFTFVNGGNDYGSYNKSTNESTGM

LREIMTGRADLAITDLTITSEREQALDFTIPFMNLGIAILYLKPQKATPELFTFMDPFSEEVWWFLGFSF

LGVSLSFFILGRLSPSEWDNPYPCIEEPEELENQFTLGNSIWFTTGALLQQGSEIGPKALSTRTVASFWW

FFTLIVVSSYTANLAAFLTIEKPQSLINSVDDLADNKDGVVYGAKKTGSTRNFFMTSAEERYKKMNKFMS

ENPQYLTEDNMEGVNRVKTNTHYAFLMESTSIEYNTKRECNLKKIGDALDEKGYGIAMRKDWPHRGKFNN

ALLELQEQGVLEKMKNKWWNEVGTGICATKEDAPDATPLDMNNLEGVFFVLLVGSCCALLYGIISWVLFV

MKKAHHYRVPLRDALKEEFQFVIDFNNYVRVLKNSASIYSRSRQSSMSVASVAQESQ

>DmelNmdar1

MAMAEFVFCRPLFGLAIVLLVAPIDAAQRHTASDNPSTYNIGGVLSNSDSEEHFSTTIKHLNFDQQYVPR

KVTYYDKTIRMDKNPIKTVFNVCDKLIENRVYAVVVSHEQTSGDLSPAAVSYTSGFYSIPVIGISSRDAA

FSDKNIHVSFLRTVPPYYHQADVWLEMLSHFAYTKVIIIHSSDTDGRAILGRFQTTSQTYYDDVDVRATV

ELIVEFEPKLESFTEHLIDMKTAQSRVYLMYASTEDAQVIFRDAGEYNMTGEGHVWIVTEQALFSNNTPD

GVLGLQLEHAHSDKGHIRDSVYVLASAIKEMISNETIAEAPKDCGDSAVNWESGKRLFQYLKSRNITGET

GQVAFDDNGDRIYAGYDVINIREQQKKHVVGKFSYDSMRAKMRMRINDSEIIWPGKQRRKPEGIMIPTHL

RLLTIEEKPFVYVRRMGDDEFRCEPDERPCPLFNNSDATANEFCCRGYCIDLLIELSKRINFTYDLALSP

DGQFGHYILRNNTGAMTLRKEWTGLIGELVNERADMIVAPLTINPERAEYIEFSKPFKYQGITILEKKPS

RSSTLVSFLQPFSNTLWILVMVSVHVVALVLYLLDRFSPFGRFKLSHSDSNEEKALNLSSAVWFAWGVLL

NSGIGEGTPRSFSARVLGMVWAGFAMIIVASYTANLAAFLVLERPKTKLSGINDARLRNTMENLTCATVK

GSSVDMYFRRQVELSNMYRTMEANNYATAEQAIQDVKKGKLMAFIWDSSRLEYEASKDCELVTAGELFGR

SGYGIGLQKGSPWTDAVTLAILEFHESGFMEKLDKQWIFHGHVQQNCELFEKTPNTLGLKNMAGVFILVG

VGIAGGVGLIIIEVIYKKHQVKKQKRLDIARHAADKWRGTIEKRKTIRASLAMQRQYNVGLNSTHAPGTI

SLAVDKRRYPRLGQRLGPERAWPGDAADVLRIRRPYELGNPGQSPKVMAANQPGMPMPMLGKTRPQQSVL

PPRYSPGYTSDVSHLVV

>DmelNmdar2

MMPSRVKLKRGTDGPTPTPTPMPTTMRKHTPIATLNTASCQHNSTTSRRKRILTPPSGPISLLLLTVLTL

LILDTRSCQGLRLTNGGGSLSKGAAANKEQLNIGLIAPHTNFGKREYLRSINNAVTGLTKTRGAKLTFLK

DYSFEQKNIHFDMMSLTPSPTAILSTLCKEFLRVNVSAILYMMNNEQFGHSTASAQYFLQLAGYLGIPVI

SWNADNSGLERRASQSTLQLQLAPSIEHQSAAMLSILERYKWHQFSVVTSQIAGHDDFVQAVRERVAEMQ

EHFKFTILNSIVVTRTSDLMELVNSEARVMLLYATQTEAITILRAAEEMKLTGENYVWVVSQSVIEKKDA

HSQFPVGMLGVHFDTSSAALMNEISNAIKIYSYGVEAYLTDPANRDRRLTTQSLSCEDEGRGRWDNGEIF

FKYLRNVSIEGDLNKPNIEFTADGDLRSAELKIMNLRPSANNKNLVWEEIGVWKSWETQKLDIRDIAWPG

NSHAPPQGVPEKFHLKITFLEEAPYINLSPADPVSGKCLMDRGVLCRVAADHEMAADIDVGQAHRNESFY

QCCSGFCIDLLEKFAEELGFTYELVRVEDGKWGTLENGKWNGLIADLVNRKTDMVLTSLMINTEREAVVD

FSEPFMETGIAIVVAKRTGIISPTAFLEPFDTASWMLVGIVAIQAATFMIFLFEWLSPSGYDMKLYLQNT

NVTPYRFSLFRTYWLVWAVLFQAAVHVDSPRGFTSRFMTNVWALFAVVFLAIYTANLAAFMITREEFHEF

SGLNDSRLVHPFSHKPSFKFGTIPYSHTDSTIHKYFNVMHNYMRQYNKTSVADGVAAVLNGNLDSFIYDG

TVLDYLVAQDEDCRLMTVGSWYAMTGYGLAFSRNSKYVQMFNKRLLEFRANGDLERLRRYWMTGTCRPG
K

QEHKSSDPLALEQFLSAFLLLMAGILLAALLLLLLEHVYFKYIRKRLAKKDGGHCCALISLSMGKSLTFRG

AVFEATEILKKHRCNDPICDTHLWKVKHELDMSRLRVRQLEKVMDKHGIKAPQLRLASSSDLLNHHHLKE

RPPLLGNLSLAASAQDLYRWSYKTEIAEMETVL

>Vd18951

MLFLIGTQLSRSRPPSTRPSPQSRTGNSSADWADSRRLFVYSSLLLLFVTRCAALPPVIN

VGGLFDNEDDEQDFAFRVAIERVNNDQSILLKSRLVPRVERVEKDDCFRATKKVCTLLRE

GIAGIFGPTSDVTSMHVQSICDALDVPHVEMRWDFQLQRDDLSINLFPKPSILAQAYVDL

IKTWDWKSFALVYEDHEGIIRLKDFIKGARREGWKIQMYQFVPNEPYRDLFWKIKKSEQN

RVVLDVHRKNLYECLKHAQQVGMLTESHSYLISSLDLHTVDLEEFKYGQTKITGLRLVDV

ESPELQNFLDDWKRLAQQPKHVRAPPAPGPHLIKTETALMYDAVKLFAMGLQQLDLTKAV

DFPVISCDAAESSSDGSSLINLMRPITLQGLTGDISFDSQGFRSTFQLDVMSLKTDGLQK

VGYWNPKQRVVVEDNSTSDYDSLRLRNKTLIVSTVLTDPYMMLKESAKFLTGNERFEGYC

VDLLQELSKDLGFSYEIRLAADGAYGIKSDTGVWNGMIGEVVYGKADLAIADLTITSARE

AAVDFTMPFMNTGISILFKKPTQKATSLFGFLSPFSTEVWTYVVGAYLGVSCVLFLVGRM

SPYEWDNPHPCRQNDQVLENSFSLLNSMWFTIGSLMQQGSDLAPKAMSTRTVAGIWYFFT

LIMISSYTANLAAFLTVEKTVYPVESAEDLAKQTKIQYGCVKSGSTRAFFKESKIPTFMK

MHKFMEERNTYVATSAEGKQRVSNGDYAFLMESASIEFLVERNCNLTQIGSLLDNKGYGI

VTVKNSPFRQVLSSGILQLQEAGKLHSFKEKWWKERKGGGKCTDDTKKSSAVTELSLANV

GGVFVVLLLGLLLAALVGIAEFLWKARSLSKEDKVSICSEMLKQLKFAVSCRSSTMPVRS

SRQTPELDTVDANNLSNRYGISTLPSFTSDF

>Vd20015

MSSDDVSDWPQSSPATKTRPTAAWQVPRLELRLKAGSAKTQAVMNNQWNQRVNDTNRYSY

YKYGIKKNKTNYQDLLNSQDFDKVTAGQRVIPGDRVSNLRNLHRFQCRCLDTRTPFIDYQ

LEPRSRDHVLSSMVVNSIQTSASSFLCPTRSSLEKQIDVNVFLLEIYNTEITTNNHNDLT

LKSKAVTTTSTVLNACKNHGGMISNICRNDSNEVYADDKDGDEQKGGDDDNYSSTTSTLD

HNSQQLAIGKPELLSLWRSPVWPAHMPSKAESKYLRDPEAKPRKRYRLNRQRSLDVTARN

DEPLVCIRQLIHRLASRYVATSLYTVSGTGMGNALLPLMVVLAAAACLPSAVDGAKSSTD

AFAIGGVFDQGENSHLDEHLSRAVERVNRDGALLRGRELQSISFKTNPGDSFVASKCVCE

LARQGVVALVGPTSWQVATYVASAAARLHVPHLKTSAGGFRERRSPFSLQLHPAVNQLNK

AYYDLIKSRKWKSFAVLYDNDNVFVALKDVLNASLNPPNVLMYPYNPALSFKKMLKDIGS

KNIYNIILHLELTKVPQLFREAEEVNQTTLYHDYIVMGLDFHTLDLREFYSLKANVTAFR

IIDPDRVAVQLVRRDWALTQGLQPDSGVSASHSKGLGSPGKRGLTGTALFWRKPRDLSSG

LPASIDQMSTAEALLYDAVSLVAAGVEALLEKDKEAAKDNKAAKGKDNSKDKDLRAGPLV

LSQLDCNRSAHWEHGKALLEAIKGARIRGLSGDLRLNRRTGTRDEFGLDVVELKYTGLKK

IASWSPREGLKLNAANRTEQEADLSRTLEGMTLRVVTVLNEPYTMLHPPSENRTGNDRFY

GYAIDLIAALASLYKFNYTFYISPDRKYGSRQPDGKWNGMVGELIQRKADIAVVDLTITY

EREQVVDFTIPFMNTGISILFKKPEKSEPAIFSFLYPFSIVVWFYTLTVYTFVSILVYVL

GRFTPYEWVPSHPCDPQSEPENQFSSLQNAFWFTMGSIMQQGSDLVPRAISTRVLASIWY

FFTLILISSYTANLAAFLTAARMGAPIENANDLAKQTKIAYGCLGGGSTYGFFKKHNDPV

MKRMWTYMETARPAVFTSSNKEGIERVLRGDYAYLMEALSIEYLVERNCNLTQIGGLLDN

KGYGIATPLKSPLRSALTSAILILQEKGVLHSLKMKWWKQQGGLVKCDDEKGASSTSEMD

MGSVGGVFLTLIVGCTVGVIIVVCEFCWKRAKLPYGQRGHILCEFFKEMRHVLACQGSRP

NFDSREDMDDQTQDMDGAVGTGAAGALHDEADAPGVALADSLHLHQLQRQIANLNSMSSG

ESGVVAKTKIARPATLQDIRELRLHDLQYHQQFRQKQQHQQYNGNSHGSGDPQSGSKAHI

GSGSFSRQTSASHLSRTGTLQSRHSNHSVRSSHTTDSVHSMPSVHSNHWGVYEN

>Vd20731

MSSFAASLAATIDPVRSQLLLSLASLSILLSALHRSQAIDHVNIGGIFDQEDEGLANHFT

LAVDRVNRDLGMLGATKFQAKMFRVTPKDSFGASKRVCELARHKVAAFVGPSSPSVKELV

KATAFRLNVPHVSTSWELHERQSPYTLRMYPEADLLARAYRDIISEKKWKKFAIFYDSDD

VLVMLKYVLADGFNQTAPDVLLYEFDPDLSYEKMLKDIGTRNHFNVIIGLRPDRVKEALL

AAEGSYSSFYHSYLILGLDFHSYKMEEVVMQRYLANVTMFRLVDPSRSEFQEVNNRPRSN

SQLPTDSSIFGHVDAPNVWAPPSTTPPDDLDVERLTTEEALMYDAVSLIANGVHNLLRMN

ILGFQQLDCNKTNSNWDYGQLFLDHMKELTIRGLTGDVRLDKEGKRDMFTLDVLELNKGS

KQWSFLKKSSWDPTTGINKPEGDEAVHDVEFSVSGMILKVVTVENAPYTIINHSLTGQDR

FYGYAIDLIKMLAEKANFTPEFYIAPDSAYGSHIGGGVWNGMIGDLMKHKADIAIVDLTT

TAERESVVDFTTPFMNTGISILLRTPEMQPPSLFSFLHPFSPLVWFYTLTVYILITVAVY

VLGRFTPYEWVPSHPCDPDSEPENQFGTISDCFWFTMGSIMQQGSDLVPRAVSTRTLASL

WYFFCLILISSYTANLAAFLTAARMSSPIENANDLAKQTDIAYGAKDGGSTKRFFQNSNN

TVYKRMWAYMESQKPSVFPKTNEEGIQRVLKGDYAYLMEVTSIDYLVERNCNLTKIGGLL

DNKGYGIATPQGSQVRGILEHHLIIMQEKGVLQELKDLWWKIPGEPCDRVKDDSAEMSME

SLGGVFYTLYGGVLIGVFVALIEFWWEKSQAPYGERDHILVEFLRELKLVLTCTGSRPNP

ESADNSIADDAASRRSGHSRQASQVFATLNT

>Vd20855

MHITIETSSKTGQSCTLNGSGHIHRLTTYQNHRGPAWGKCRSFTLQRPLKANDAAFKIGT

SPRRQIEEHPLRSNSSVEAAYVTRTNHGQWPSVRHKWSSPSSSSCLPNLINKTWPRWHSR

WHLAVAVFVIDCSLLMLPNHVGAAKGLPDRIPVGAIFLKEGETELRAAFEHAIEVHNYRD

SHFSVAPHVELVESDDPFQVAKKICSILKNGTFAMVATTTASSYETLVSYSNTFQIPIVS

PSFPYHTRLRPARYGISMRPDYLKAILDLIQHYRWKQIVYIYDSDAGLLKLQNLYKLTTD

EYKLEIIKVRRVTSASEANKFLVNYDNETRDQLKHVVIDCEANLTREIIIKHVKHPIVGR

RTFHFLLGGLIMDEFANNPAIEFGAVNITGFRLVQNNSRAYEEVFTEKSRHQWPDPKTKT

ITSEAALMYDGTRVMLGAFKQLLNEYPAIFAKNFRRGEVYNNNTRGIDCKREKVIPWEHG

NEIFHRLRNIKGIRGLSGNISFDPYGLRSNYTVDIVEVTVNSELTTIAEWNSQMGFVFAA

PKYRRIFVDSGFKSNKTYIVTSILEEPYLMRRKMEPGVNLTGNSQFEGYCKDLADLIAEK

LNFTYELRLVKDGKYGGQSADSESGWNGMVGELIRNEADMAIAPLTITSARERVIDFTKP

FMSLGISIMIKKPMKKKPGVFSFMNPLSREIWMCIIFAYVGVSVVLFLVSRFSPHEWRYE

ETFVGPTVSNDFSLYNSLWFSLGAFMQQGCDICPRSVSGRIVGGVWWFFTLIIISSYTAN

LAAFLTVERMVTPINSADDLAKQTEVEYGTLESSSTQDFFRKSKIAVYARMWEFMHTRKN

VFAATYEQGIQRVRESKGKYAFLMESTKNDYINERRPCDTMKVGGNLDAKGYGVGTPLGS

SLRDQLNLAVLSFKENGDLARLQNKWWYDRSECKTGDANKESTQNELTLSNVAGCFYILI

GGLVLAMLVALLEFCYKSRLEATRSKTTMYNAMKAKVRMSITGTQNGTGPSGGGNVAGGV

GVQGLAEDRIRKSLAEAALYGDSPSSPG

>Vd21106

MQLHWVLVGALGALAYSAHEVLAKEKLRIPIGTILLPNQEVYENILRYAVKNFDSSLFDT

RLVAMRLSNADFVQINMKVCAMMREGVLAFVVAPDSAPALREMLTSYSSHFHIPLATSLA

SRSFSRSEYAISTQVSLADATASLLRRYRWSQVAYIFDDSASPDILEAIQNSGVKISIAK

KVNNEAEASDAVLELSSLGRLKPEKIEKIVLNVNRPSLAKTVLSGMIREVKSFKNLNTQF

ILANPVSDDFWQYIRFRDAGSSLNVTAFRLVDSELPHVTRFHKQMMQLSSHRSLFHNQGT

QSYLFLDAVTALLTGFDRVLKDNPRLLDRNLRHHPGSVYFNGTKGLDCMDQSRFFEYGDI

LAAFIKQIKIPQGQSGPVEFHDNGERVQPTITVIQGSHKGHVRLGTWTAKKGLMLADKPE

ALPQIKAPGAITPAKELSVGSVLNSPYLIQRQTDGEFSGFVPDFLEALSNVVPFAYKIQV

VRQRDYGRRSENGTWSGLMAEVINKKVEIAVSDMALTAERQEAVECTQPFFVDDLAVVMG

RTTPAGRPSLSFFFVRVFSWQLWACVAAILAVFIIFSYILNHLAQSRQRNSSEKNRLLCL

VWFTLASVFARSQGPHVNSKAYRILLASWWLFLAVMTITYLSACIALLQAVLFGSGPDLF

NKDKFCRDAANGAVDVGYMRHGAVEKMIKNQAETQLSFYAQLLNNSRIRIVSGIAEGMRH

VGPNYAFLGLKSQVDSYIGRDCGIKSYTLIPQFAQYALVMPKMSPYRDMFNQGIEKLRES
GVLADLKEKWIDSLVRCPREVEVLEVPAPIRYTALCGMFIIVLAGFILALLAGVIEFCWS
AKRDSSKSEKPIQKVMWDKAHDSLKPFTQKDPALGNDEEMTLNPSQANGTPKQVRVMV
>Vd21675
MRWIAFVLVLCTTVLVQAGDLPVMKIGALLDEDEQVASGALMDTLVARINADHKILPRFR
LHVVRVKVTNSFAAAKATCALIDDGCVALIGPTQHESHRMAQEIAARLQVPFFARGRPGA
SVGMTGASPAGGDNSGPSSSCSTCFDLLPPSRALSRAFADILKAKKWKNYALIYEKNQDL
VELAELIKNKNILLYQYQRGERGTKRLIREIGSDPQYNYIVQLPADRANDFFRLAGIVGL
LSEYFSYFVTAQDFHTWQLPAEALSNITALQLLHQEFVSLPSDSNGKRQRGGPKQQQFIH
QHQASQQQQFQQQQQAQQQLQTPPAPLKYHTALLHDYIFVFARAVGTLSRTQNMAPVSDA
SCHKPRNGWPMGLTIATQIKATSMQGLFGNIQFDTFGARSNFSAFVTEHKHGTIVKVGTW
APSSGFSMTRNVSWDRVHAEHSLRNRPLRVVTLLSPPFVMWRQQQNSAKSERNGDLEGFC
IDLLNELSRMLQLKFDIRLVNDSQHGSRDRQGNWNGLIKDILDMEADVALADLTVNVERA
RVVSFTTPFLPSQLELLYRPPSTAGDLVSLVKELFSPLSQEVWLSGVACVFMATFAMSYA
SKFSPRDRVRRSSSKQANIQTWTKPRFSFGDIFHLVACCALRQPPISKRPRGVATRFLTS
VLCFTSFVFWSVYTARLTAQFVQRRMHFDELHDAGDLLRVKGIRFGYVANTSSQLFLQGA
NYEPFSTIWSLIMGAGDISTLPSLEEGLRLVQAGGYAMFTETPAAEYHQCSISDCSVLRL
ATGIETPGYALALPRGKACMKNTVP
>Vd21743
LNPNRPNVTYVMIVPSTRFESVRRKYRSTINEALNQIKNGKVPGNHLNKHYSLQFQLVYL
SLAPTPREILDTLCTEILNASVLSVGYFTNSETFGSNAASVEYVHQLLGYLGIPVISWNP
DNIALDERVTDAHILGLSPSVDHQVNSIFDFLDRYQWTQFAIITTQLAGHENFIRAIREK
VFHKQHKYSLIAAHTLPRGGDADKYFNLLQELADGEVRVIVLFCGTEDAREIFLASSRHG
ITGKNYAWIVTQAVIGSAERSPAEFPTGLIGMYYNFTLPVLLDEMEKSMYIFASALERLV
NHTTEENRNQLSTGLTCNASEYSYWRKGEEFYKYLKETEWTSKKFNSVAFNFDGTRKRVD
LDILNLDARNVWEKIGDWGESGVDIKDIIWPGEERKPPKGVPEKFNLKVTFMEERPFVIV
GLPDPETGECESSRAVKCRIAPESALIGLNDTMARRHPNYYRCCMGFCIDLLEKFAQDLG
FTYDLSKVEDGMWGVKDKNGKWNGLIAALLNRHTDIVVTSIKINSDRQEAVDFTVPFLET
GIAIVVAKRTGIISPKAFLEPFDTVSWLLILLVGIQVAAFSIFIFEFLSPDGYDMKIAPP
RNYKFSLFRTYWLVWAVLFGAAVNVDCPRGYTARFMSNVWAMFAVVFLAIYTANLAAFMI
TREEYYDLSGIEDSRLRYPHLMEPPFRFGTIPYGNTEQVLQRNKPTMYEYMRPYNRSNVN
EGIKAVKKGTLDAFIYDATVLDYFVGQDDECRLLTVGSWYAMTGYGFALPKKSKYLQMFN
RQMIEYREHGDLERLQRFWLQGACKPDKRKRNAGNPLDINQFMSAFLLLGCGVLLTVVLL
ILEHVYFRYCRKQLAKANFGSCFSLISLSMGKSLSFRDAVYSAQGMLLRRATENRFLQAA

EHERLQADVARLARELDVLRQKIKTLESDLTTTKAGSNGGSLDELRGTGIDTTGSIPGSQ

RVARPQAPKRTPFGGPLAISGSHPNVADKIVGLRQASIHSNTSVGAESPTRSLARFTGSH

EDLLRHRDLTRTKIHSNEPLVISQASILPAVPPHSVLFQQQQAMVKAHIMEKETVL

>Vd21758

MLITDDISMLSIRSIILFLVVTLFAGCVEAGQPFSVNIGGIFASNSPLVRVFDFAIRRAP

ESSRLPIVRDAFIGMKKVIPDIHSSFDASKAVCQLLSRAPSIVVGPTVVPVSHILASTCR

AFHVPFLSIGPLLSRTSAASPRKLPQMLNLHPPSDILGRAYFDVLNMTEWRSLTIVYDRE

DAFLRLAPILRAAPSLTTLRPLDKSVSPGTLFKEVLRSKDTHIVLDVDTQLIPELLRSAK

DVGLMTEYHNFVITSLDLHTLDLSPFFPSRANITFLQLLDLKSKENLKMIRDFRRFQLMD

GIRDNLNLTTEQALLIDSVSFVVRTLYELSASSKLRSPPPFKCQSPRPWQTGDLVIKKML

RSSFRGVTGIITLNRHGLRSRFQVNVMRIMWNGVAKIATWNQDSGIRTQASYPATFRKEL

ENLMASKVFNVTTIVNPPYTMLKSDYRNRTGNDRFEGFCMELMETLAANIGFRYSVHLVK

DNSYGSRQPDGTFDGMIKELINMEADMAIVDLSITAERMQAVDFTQPFLKTGISILFRKP

AQGGLTLFLFMKPFSIDVWICMLTAFTGVTVLYYLIASISPTENRDLGEDDLSQRDPQLL

DQEMKIMSRFWFTIGSIMQQGCDLNPVSLSCRTIASIWWFFTLIVVSSYTASLAATLTAE

RLVSSIDSADDLARQSTIEYGCLASGSSRKFFEVVLLPLQDNSHSLKTAVLFF

>Vd21835

MRLQAKNVEMHRPGFGFIVLLVLLGPALAAVKIGGIIAAGQPRTKELLEEILKERSSGLP

TKNRMHPELAYVEPNATFGISNTACAQVGQGVSSIVSPCTGIVHATLQSLASRLAIPYIN

TAPDLVDTTFNTKQLGESEKSITIVMAPSTAKATADLIRKNQWKDIYFIYDKPEGLEELE

LILSHLLEEDEVQVKGIHQVTSNTSAMTYLQEKENAAWDVEKHIILAIDVKMAKRIIQHS

YSKRRAYYHFVVPRFTVRHFWSEVRAFDQSTVATFQIVEETEFKTMKGMRAGVSTPKNSE

DEHIHEEEALLMDAGRLLSDTMVALSGKQPLAAEDIFKELSQITAAVTCYRSPLEEYPQG

QTIADVMRKIHFKGSTGQVRFNDHGRRTGYTLDVLELTSDADMERVGKWNEQEGLAFSNE

HERNSKRARIKQELSKSIIISTIIERPFLFFKNTNETNPHIDNVDGYIKDLIVRLMKDIP

GRPQFNIHFVKDGKFGYKDSQQASGWNGFIGEILRKDAHMAIAPLMITSERSKVVHFSQP

FMATAIGAVARNDADDLLNIRTPFTFLRPLDWKIWLLFLGAYGTVAIVLLLINLINLAVA

TPPVDPPTPCRHFYNSLWYAFGMFMSEECNNCRPKSLGSRVIACSWWFFILCVLSIYTAQ

LWINDPFAPTGEKLPRCLKELLYQDRIEYGLIRGSATEDFFQNTDDSEYKAMYRRILSFN

DSVIESYPKAIDRVRNNSKFIVFADSNFIRYTSAHTPCDTIQVGTDLRRGQYAVAMSKHF

ALSKKIDDIISSLRDEGFLAELEDRWFGAHALDCPADNLVIAPSLAYGGEKPLDIMKPLE

VRRVLGWFIVLGLGLIIALFITICEVYCKKPKKQRTPEEIVERQTAREAKKSRSKGKKDK

AEEVETQQLTAHEAPIGNSQMDDATRMGVGIANAADDATLKKGGCLPREMKIDEEYLDGD

DGGFRRSELPPIADVEPFTDTDSRDAIQSPVSGQSTIKMHSGNTTEAVNAGLIGIKPSSQ

SPPTLILKSPQQPCQPIQPILTQSQPKMPITTQIQPTFQTQPLTTKSTIPQMSKAPQVAQ

MGRSMSQPVASLPPLSQRVPPVQLPPTMAPIKNTAPAQATPTSAPRTPSPAPAGPPIPKS

TLPSMSPVGPMSPLSPASPASPASPNALPPLSGLSKLGGDPPPPAVVNQGPLSRMELEGQ

PELKSIDRSPADTPGTPHDDLERIDPILGNLGSLAGLPPPPANIRSTIQNQDSIGYESSE

V

>Vd22269i3

MSGVVAPVLALALLGVAVADIKIGGLFETGEDIVEMAFNSAVERINSHGLAEPGERYTPT

SDRLLTRVEHIERADSFQASRKVCTLLEEGVAAVFGPQSTDASFAVRSACETLDVPHIET

HWDYRSRTTNHSLNLYPHPSALGKAYLDFIKYKDWKNFAILYEENDALVRLQEILKDATL

MKENRVTVRQFEIGTEYRKTLKDIGKTGIKNIVLDVPIRSLYTVLKHAQQVDMMSEYHNY

FITSLDAHTIDTEDFQYGGTNISAFRLIDFSSKEIDEVARDWIQRRHRFSSKKSSSVQSN

FNTGAQDFYKNLTTKVALMYDAVRLFATALKDLHPDSGPKIQVAPLSCEREHKWSQGNAF

VKYMRMINIKGLTGNIRFNKDGHRTDMRLTVLDMAHSGWREAGEWTMHGGISITANYTRE

LEEARLSLLNKTLVVTTILQPPYVMYKENWRDNGLTGNDKYEGYCIDLLDAIASSEHFDN

NMKYVIREVADNSYGRKDADGRWNGMIGELLSGKADLAVADLTITFVREEAVDFTMPFMT

LGISILYKKTAKPPPGLFSFLDPLSLEVWIYMMTAFLGVSLFLFVLARFSPYEWVNPHPC

EGNPEELDNQFTIWNTLWFTIGCLMQQGCDVTPRALSTRVAAGMWWFFTLIMVSSYTANL

AAFLTVERLVSPIESVEDLAKQTTIQYGCLRSGSTQSFFKDSQFPTYAKMWHVMQSQRPT

VFAESNQKGIDRVLRGKYAYLMESTSIEYNIERNCDLTQIGSLLDNKGYGIATPPGSPYR

TMLSQAILRLQENGDLHVLKERWWKKKRISKKCPKDIINKGTSAMTVASVGGVFIVLLVG

SCVAIISAIVEFVWRAKKIVPEERDPVCVELCRELKFTLTCGSKKTPDTPSLG

>Vd22292

MHNIMTVVQKALKALEQIEAMRRRVKTSVHYPTIFECRSGRAGANLLLSLLVVPGVFASS

VPTVTADLHSNGNSASVLDPEIISKAVLDADLSVNAINLAAEELPRVYNVGAVLSHPEGI

AYFEEAILDAMATYNIDLVAKSVHMSLNPIRMAENVCEQLISQQVYAVVISSPLKGELSP

AAVSYTCGFYSIPVIGISSRHSSLSDKNLHRTFLRTVPPYSHQADVWMKLLRHLGYQSVV

FVHSSDNDGRATLGRFHSVAAQHKNAVHIEHVIEFESGVSETDLTAALRNASRRHCRVYV

LYADTSEASKVFDVVKKLDMTTSGYVWIVSEQALKAPNCPDGVLGLELVNAVDERAHIRD

SVNLIALALKKLQKDSKVAAPQLNCSNLEHNWDAGLKLVSILKEQVLESGENGHVKFDQR

GDRLNSDYDIFNIQRDKESYLVKVGEYMYSEYDMEMELKINLNAVSWPGKATEKPLGFVI

PKHLRVATLAERPFVWSRKVNSSTECSSVEILCPWYNRTADTGHDEAFCCSGYCMDLLHF

LASKLNFTFDLYLVEDGQYGNLDSNVEGKRVWTGLIGDLVRKKADMVVAPLTITPERSME

VDFTKPFKYQGITILAKKQDKSSTLASFLQPFQKSLWILVVVSVHVVALGLYLLDRFSPF

GNYRVLPSERDEDGLNLSSALWFAWGVLLNSGMAEGTPRSFSGRVLGMVWAGFAMIVVAS

YTANLAAFLVLEKPESSLAGINDPRLRNPSENFTYATVRGSAVDTYFKRQVELQNMYRIM
EGKNFDTVDHGIHALMNGNIDAFIWDSSRLEYEAARHCELLTAGEQFGRSGYGVALQRNS
FWVDKVTLALLEMHESGFMEQLDSLWIHNGGRRCESKLERTPATLGLTNMAGVFILVGAG
IFGGLVLIIIEINYKRYLAKQKRRMQIARKAAAKWRGIVERRRSLRRPSHHRIMLPSNIQ
QLPVLIGSLASGMPSAQTVVPAHSGASKHENLGHQGLSRLKSASTSIKSSHETRSDLADL
PPPPPPPARVQATPGTRMNASTRYLLGAQENKPVSFDINV
>Vd370
MGYRSGSRHLAGVTTLSVLFLCGWMNTTGAVPARVHKDVGTIGIIVEDQDLAKHFLYIAS
KLVDSNLKAVSKVIQNDDILEAIQSMCALMESDVNVVFGPTSPVSSLHVSSMARHFDLPH
IEFRWDPSLIDDEHSLNLFPDPVQIAEAQTALMEYWKWRDIALIYESDDDIIEMRRLLQE
LQEDGITVNYFKKDKGETFRTLLRQIKERDIRNVVVDLEQKAEREKDKEGKIESFLKQAM
QVAMLNENYNYFFLSLDFHVAKLNDFMHSRANITALRLVDMKHHIADDLLRGQDSPQDNK
KGSASGRHNQQKAAMARARRDRNDRRLLKRDEHNGEADEEEEADEDASFPPGLETEVALI
YDAIMLFKLGVKDLVNKKSSYTKALPTAKCNGRKSLDSEHLGTELTKTMLRRTFEGATGR
VNFNEKGIREDFVLFVTELGTDGLEEIGIWSKSSGLNITNDENEFELSKEEKTKIDDIHL
RIVTVMIPPWIMKDEKLSTEDRPRYKGFLIDLLEDMKKRSPRPLDYELYTSPDGSYGVET
RYENGTITWNGMIGELVKEKANVAIADLIVSDERLRAVDFTLPFMTAQLVAVVKAPVPRK
PGGVWSFFLPLTREVWMYTVVAGLLTALIMYMCARFCLSEYVNTGDGVNGPAEEVDNQLG
IVSCLLFVFTTLLHQRMHLDPNATATRVLAGFWYFFTFVILAIIVGNLCESVLYEDEDFS
KLETIQGLLRIRGMKFTCIEHGATCRTLMTSHAPVLWEINKRMFKDETPKVKNMTHALAK
ISQDEHLAFIMEYASAKYLVASSCDLVMTKPFANHASYGIALNKKNPGKTNRLLSEIVLN
LQAKGILQEREERWWKTKRFCDVDNDGDFDDVDDGDDDSINEQKVELRTLSPGDLSGAFI
LLFLGLCFALLINIGEVIVEHLTREKRDTYFNMITVVDHIRFAFKMQTNQVDLSETSLPR
NHWKDQLQKRRENRTRENGQKSNDPTSPRKTDSLSSKIPQYQ
>Vd52944
MTWLCAVLASVTVVSISFSTGVHGGTYKIAGVFSAETGQVESKVFARAVDRVNTDNREFP
EVQMQAIPIMLKEGQTWENYQELCRQFESGVVAIVGAESSVEDSLISSVSSAVRIPHIRT
GFNRELEDPDDLSLGLAPSPSDLAEVVRELILHMGWNHIVYIYDTSTGLLRMNELEEDPE
FKGRRLVPIDLSVDANPWDNIIKLSIRHVVVDIESKSVHQVLKEISMSGLSQDYYSFIMP
NSDIRLPENSTQRTFFIDADVYTFSLVDPDNREMHWIYRMLEMNSSENEPNLPFIYDSVL
QIGHTLNNLTKLQRPIRSSPGNCTLKVAFKDGRKLYDSIRESTKFEGVTGFVEFDDKGAR
KVKHVHVKLLKDGKLTKVADWRPLDGLTFIKELDPKGEPDEALEHDEGLNNRALVVTTIL
LPPFLMRDDSPHLKGNERYSGFIVDLLEKLARLLKFQYLIKEVEDGAYGTYNATTNSYNG
MIGEILLGKADIAVAPFTINAERYQILDFSFPFLEAGLGLLARREVDPLEHNFLSPFSAI

MWFSLFGTMMFATFVITFIARLSPREWSSVTDTVIETHFTVGNCFWYLYSTLMFQRFQHR
PNSLGVIVAFFAWFIFCLAIMVSYGTVLGDFIIKYNERPALSKIDDLRSENNRVKFSAIK
TGSTMRFFEETEYPNYKQLYAKLDKDAAVRNLSEAIQRVKNEDFALFLETPYIEYFVGHD
CSLQQVEGTVGSVSYGIAMSQGSSLQKYLSAGVLSLRREGSLQSMRAKWWNEQHAGRHCP
DHLHIVRPTPFTVRFGRVNMLFVFLLGGIILSVAITIYNYVRKQMTIEDKNEFRYFLPTL
IREVETLFRKRPVPTNPSPEPTAA
>Vd17150
MEILLRVVYELLVTLYFVENGLGQTLNIAVIVEKKFELARQAVEAGLQRAKNDGKTIHWQ
FVVMDADDELKDLCDIIMENQPSLVINTVRTPGSNYYKLTKEIKQTVRNLAIPTLDLSYG
STDEYQMSGWNGKKPEENDYLIHVTPPGDSYTQAVRELSIKMDLATAGILYDKSVIIDHK
YARLLENVPTRHIMRQVSESSADFVNKTKVIQSTDVSNYFVVGTISHLSNALHSAQENDW
KPRHHCWVLITKQEGEPVCEDCMNHEVLFVHPADKEGKRIGIDGAYGSEKLDGLFYEEIT
YYMITRIFADNKYSKQPIRASCREKPATRDDYNLLNALESSKLSTRYGDIYFTDAGNKPG
YQAINMKVAHFNYTASKTRQDNEKGFYVYGGDAKAAMWTKADQTALEQFGSVTFYKVVTL
IQPPFVYKGNGSGPEFYGYCIDLIDEIKKILKFEYNIYAVEDKHFGSKDKETGLWNGLIG
ELVKKKAEIALGPIAVMAERETVVDFTVPYYDLVGLSILMKKPEIKPSLFKFLTVLETNV
WGCILAAYFFTSFLMFVFDRLSPYSYRNNKEKYKDDDEKREFTLKECLWFCMTSLTPQGG
GEAPRNLSGRLVAATWWLFGFIIIASYTANLAAFLTVSRLESPIESLDDLSKQYKVKYAP
QGQTTASTYFHRMANIEEKFYEIWKNMSLDDTLTDDQRAELAVWDYPVSDKYTKIWWTMQ
EASLPLTFEEGVARVKNSQGSDGFAFIADATQIKYATMTNCDLTQIGSEFSRKPLALAVQ
QNSPLRDQLSSAILKLLNQRKLESLKEAWWNGNPEKKNCDDEGKNNDGISIKNIGGVFIV
IFIGVVMACITLVFEYFWYKNKKPRSKVVSVKDHMPQKK
>Vd18850
MAVKSMEYQINRVTTVHHRYEANIPADQQWGVIFPNGSATGMIGMLTRNESDWAANAFGQ
TLDRHRVVTLSSEFMVQDLSILAGRTFGFQDNYFGLFGAFESNVWTCLLMATLVMPVILV
LSRLLERNEGNVNRTLFSRMLVLYGRNLMSVVRTILFESIEDRDLPKGNSGRILIGLWLT
SIYLTMSIFQGTMKASLLISSGSSRIDSMRDLVVRKQVVPIIWRGTAYHKLFQRADMDIY
QTVYSRAMEKNGILPGNQLYTNENFMNVLQGRAVIINEQSSIMYNLGRSCDALRGHPGEF
YFAKEPVYTHGLSMSLRKTLHPMLKRVINKTIRELQETGNIRKWLNIAMADFFTCEIVNG
APTQEEPTVYTLGLSKLKGPLIIYGMGAVATLITLCAELAWESKCFCFIWQNGKLIA
>Vd19098
MTEVTSREGRVVNVNGTLVELLKELARKLNFTYTFIMPPEPVPGLKQSDGRWSGLIGLLV
RQEIDLALYDFTPTPDRQQAVNFTVAFDESPYKFLVPKPQPNYKYLFLDPFTWDTWLAVL
GTVLIIGPILWCVHINSKFYDYYDMRDGKGLFKLANCEWYCFGAIIQQGGIHLPDAISGR

ILVGFWWLFVIVTLTTYSGNLVADLTFPKIRNPYDTVSSLLNSDITWGAFKGHAVIEILK

LQQQGLLTQLSAKLAHIETAHESWALKEVADGKMALIGSEVTLFHYIGKQFIATNLCQYA

VAKKEIIRQVKVLAVRPGFPFLARFNTLLTRIVETGLIIRWKKKYWPKENECTVDSKPQA

GDIRRITIAHMEGSFWILGVGFLVSFTLLALEIFRKRRELRDPNGGRPQRIGPRDFKDNL

FRKTNYSDKDHYNTDYGAQVGGAGPGAGKGGQSGYGFEPSNTPFRGYSGFPNRTDLIPYN

YPARRY

>Vd22240

TDDFWQSIRFRDAGSSLNVTAFRLVDSELPHVMRFHKKMMQNPNYRDSFKNQGTESYLFL

DAVTALLTNYDRALQDKPTLLEAYLPQEDGSKGTVGFNCMDQSRFFEHGKDLASLLKQIK

LPEGQTGTVEFLADGSRLQPTIAVIHSSHVGHVKLGSWSPKKGLMLTDKPEALPQIKAPG

AITPNKEISVGGILSPPYLMLKRGSDADFTGLIPELLEALANVMPFSYKIHAVPRGQAGA

RTDNNASWTGLISEVINKKVEIAIADIPLTADRQQHVEYTQPFLMDDLSVVVSRKSQAGR

PSLSICFITIFSWQLWLCILGVFFTFVLLAYLLNRLVSPQEEADSFGRTLWLSAGSFLCR

SPGPEGRCRASRLLLLFWWFFLAMVTVLYVSAFVAIVQTVVFGYGPDVFTMRKFVQDAAS

GTVDIGYVRDVVPGKVLK

>Isc000549

MAHSFFNKTLLDDFVESVLFWYPYDPHAAFSETPQGIKVTTGLLGDIIRELSHSMKFNETPQGIKVTTGLLGDI
IRELSN

SMKFNYTVSFTPERTLGIRHPNGSWTGLIGSLQRDEGDFALGLMIPTNSRNAIARPTTEVYIDEITIFAGRTHSK
SMNVF

SYVWVCVVIAMFTLAFLSTGFDYFYEHTVRNRKSFSERFFTHLWEYFENLLGKGSSVESDWHATRLLSGFW
WIGVTVLVF

AFAGQMRACLMVKSQEAMIRNVDDLARRSNVKPYTLSGSILTTILRNSKKPSYTKVFQRIVKGRGQTELHQI
YGPKILKE

VVQGKSVVIADRGSFTYKVARTCRNYTDGEFYIAAEPILNFRFVMYLSATMDPRTQWEINRRLVWLRENGIV
GKWIKNML

GDWEHCRQESSRVTLSFEDTYAIFVMWALLIAASLVAFGCEKLHHACAAAKERPSQRPGPILGWGVTTVSRK
RPKVAKPF

QAYNFKV

>Isc001842

VCSLLQQGVIAVMGPPDPTTALLARKACAAHRVPHVYMHREQHGPGPPLDGASFTLTPSPEEIGVAIRDLVT
AQRWNHFT

IVYDKPDALIRLRATVALLAPASGPAVTVSLRTIFRDQDPAPVLRDITKSGESNIILDIDATRLGDVLRQAQKIG
MLTEY

HNYIVTTLDLHTVDLSEFRYSRTNLSGFELLDRDLWQQDLGLTKEAYAKTGIYPLFIRSRPTRRPLSVSCRHID
CSTNVE

RLVALNPFSAETVLYALGGHSKYVCLSMQIHFFGLTGPVHLDRFGRRENITLHVVQLKKAGLASIGTWSLKA
GLKITRTT

SMVQEEILDTLKRKTFQITTLINSPYVMLKKSASQLSGNDRFEGFCVDLVRELSLLLGFRYQLRLVRDGAYGT KDSTGRW

NGMVRELVDREADLALGDLTITYVREEAVDFTMPFMTLGIGILFRKPQGDRTLFFFLSPLSSDVWLCVAVSYL GVSFLLC

LLARFSPAESGLKRRSCCCEGTLSPCGHSKESELKNQFTLLNSLWFTISAIMQQGCDASPRSASGRLLAASWW FFSFVAI

STYTANLASFLTRERLRSPIQSAEDLVKQSDVRYGCVRSGSTEAFFKAINYTTYERMWQAMKHSMVESNSEG VSRVLSEA

YAFLMESTSIEYVAQRHCQLNQVGGLLDSKGYGIATPTGSPYRNLLSSAILRLQESGTLQLLKERWWNVDTR GRCPEDAG

SGVSSLSAASELGLSKVGGVFVVLLAGLGFACIIAVVEFL

>Isc005598

ILRRLCEDLLPDDVVAVLYLTNSAVFGSNAASVQYVLQLLGYLGVPVIAWNADNIGLDQRVSQARVLQLAP SVVHQVAAI

FAILERYAWYQFAVVTTQLGGHEDFVRAKNEHIYSFSVLDVYTLRGHTRQEFRSQLEPVATGEARVLLLFAS KEDSREVF

AAVGQLGMTSKNYVWIVTQSVIGAHPGLAPQEYPAGLLGIHYNTSLAKLHDEIERAVLVLGHGLELFVNEPA NANLSLNP

GLRCYEVGRAHWSQGDHFFRYLRNVSIEAKGSPNIEFLPDGTLKYVELDIVNLNKDGYWDRIGGWTQKGIDI KDIVWPGN

ELVPPRGVPEKFNLKVTFMEERPFLNIGQPDNETGECESGRAVKCRVVPEAMLQGINDTAARKNSTYYKCCL GFCIDLLQ

KFAQDLGFTYDIHRVEDGTWGVKDNGTWNGLIAELLDKKADIVVTSIKINSDRQMAVDFTVPFLETGIAIVV AKRTGIIS

PKAFLEPFDTVSWLLILLVSIQVAAFSIFVFEWMSPSGYDMRITPPREISLFAEHRFSLFRTYWLVWAILFGAAV NVDCP

RGYTARFMSNVWAMFAVVFLAIYTANLAAFMITREEYYNLAGIEDNRLQNPQQTDPPFRFGTIPRGNTEAVL KQNKPQLY

AYMRPFNRSTVTEGIRDVKRGELDAFVYDATVLDYLVGQDDECRLLTVGSWYAMTGYGFALPKKSKYLAM FNRQMVEYRE

RGDLERLQRFWLQGACKPDKPKRNASNPLDVNQFMSAFLLLGCGVLLTLLLLFLEHIYFRYLRARPWLRPKI TLVTHPRH

HHAQSMGKSLSFREAVYEARMRRRCRDPACDNQLWKVRHQLDIARLRVSQLE

>Isc007957

MNNKNMMTFHREFVSVVTQPIDDDFQKLVVGFPDGANVLAAYPEIADNDCPVEPGCQLPLAMETVAKTIGD KLEKGTYRT

TEFFTTKFIFSNTSKSLLLASGKCGQCARFIIRSVAKVQGIQEFLKIGEWTPAVGLKMTHKQFFPGIMGNLGGIR LTIGV

INDPPMSVVEMSPDRKTVKNVTGTMADMVEALAKGLNFTYTWKVPKEEIPGSKENGNWNGLIGMLATGEA DLGAYGFSVT

KERSEVVNFTSAYDESPYKILVPKPRANYKYLFLDPFTWDTWVAVLVSLVLIGPILWGIHCASPFYDYHGLRD NKGLFLL

QNCEWYCFGAIIQQGGIHLPEAISGRILVGFWWLFVIVTLTTYSGNLVADLTFPKIRNPVDSVENLVAHRGYMRWGAFKG

QAVFELLKSQEQGPLKVLSDRMNVFEPNHEMWVLDQVRLGYMALIGSEVNMFHYLGRELNRTGECDFAVARGEVIRDVKS

LAVAPNFAFLERLNNEPDHDGRPPPRRLKRLVESGLVMRWKKKYWPQDNECTVESKPQAGDIRKITLRHMTGSFWVLGVG

FFSSFAALFVEFVRRKRELTAPPTHKPPTVIHTKSPFFTRTEYSGKDTLTTDRFATDYGGRGPRDNAGFAFSPPNSPFRY

NGYPNNRSDLIPYNYPARS

>Isc008225

MQPPFIVRKNASNGENQFEGYCIDLINAIKEILKFEYVITEVKDKMFGSIDKNTKQWNGMIRELVDKNADIALGPISVMA

ERETVVDFTVPYYDLVGISILMKKPEVNFLMYLFDRLSPYSYRNNKEKYKDDEEKRDFNLKECLWFCMTSLTPQGGGEAP

RNLSGRLVAATWWLFGFIIIASYTANLAAFLTVSRLDSPIESLDDLAKQYKIKYAPQKDTSSMTYFERMAYIEEKFYE

>Isc008266

MGTPEVLCLLLLGCLAAHADIKIGGIFETGDDVLEMAFSSAVDRVNTMGLAEPGTQFVAAGSRLLARVEHIERLDSFQAS

RKVCSLLEEGVAAVFGPQSGEASAAVRSACDVLDVPHIETHWDYRARPDNHSVNLFPHPAALGKAYLDFIKHKDWRTFAI

LYEENDALIRLQEILKDPLMRERKVVVRQFESGVEYRKILKDVGKSGIKNIVLDVPTERIHTALKHAQQVDMMSEYHNYF

ITSLDAHTVDMEDFQYGGTNISGFRLVDVNAKEFDEVARDWLTRRLRFSKKGEKEENFYRTKVALMYDAVRLFATALRDL

QPDNGPKIQVRPLSCETEEPWAQGNSFVKYMRMINVQGLTGNIRFNPNGHRTDMRLAILEITHNGLREAGEWTMHGGINI

TTNYSRQLEEARLHLMNKTLIVTTLVQAPYTMLKENHAELEGNAKYEGYCIDLLEEISKLPDISLKYKIREVADKAHGRR

DDKNEWNGMIGELLHGKADLAIADLTITYVREEVVDFTMPFMNLGISILYRKADKKPPWLFSFLAPLSLEVWIYMSTAFL

GVSLFLFVVARFSPYEWVNPHPCDPNPTELENRFTIWNTLWFTIGCLMQQGCDVTPRALSTRVAAGMWWFFTLIMVSSYT

ANLAAFLTVERLVSPIESVEDLAKQTTIQYGSLQSGSTQSFFKESEFPTYKKMWHVMQAARPSVFTESNQKGIERVRRGK

YAYLMESTSIEYAIERNCDLTQIGSLLDNKGYGIATPPGSPYRTMLSQAILQLQESGTLHVLKERWWKKRHIVKKCPKEE

ASASKGTSALGLANVGGVFVVLLTGSCIACITAIFEFIWRSRKAVPEEREPVCMELCRELKFTLTCGGKKPVQNKSEDRQ

PPGENGLPFMPLTGYNAVVSKDTFS

>Isc009282

MAPGSRPRMRASGRRSAPQWARSHRSHGAFRAAPRPVAGAMARLAPPVLVAWLLLVPAVLLVAVDSTPAL LAALPPRSAP

QLARALRRALARWGAGPGDASATSWVVAAFSAVPDTRAALSALCTALEGHAPLLVVMPVLAYTGGYLDR AAQEASPLYLS

LDPGLPELTEALFGLLSQNRWYHFVLVTDESAASSMLAQRLGSLCHGVPWRPMLRLALPGPTQPLLGPLAKI TASQARIV

LLFVEAPMVRAVLDAARTLGLLAGDHLWLFVERDDPNPASPHPCGGDVEGSALKGCPLGALSLQLRQSHVE RTDAPRLLA

GLLHDGLSRWGPIEYPLALRNASGAPTPSCWEEPSARREMFSQRLHGYLRAAAADAVQSQRFPPPVFDILNL VPAEGGAQ

WRSVGNVTKGSAVLDAVLWPSTGEPTLMGPRPFGQPRFRVVTAYAAPFVMAATRLRNGSCLTGVPCLQVST AISDELAAL

FAHYHRTGGHTRHPGYNVTCCAGIAIDLLKSLARDLSFDFDLYLVADGSFGTDRGGRWGGITADLISGAAHV AATAYSVT

SSRSRRVDFSVPFFHSGVSCLAYAVKRDVPLSAFLVPFSVPLWLAIFLSLKVTALAAALYEWFSPFGLNPWGR QRTRNFS

LASALWVMWSLLFSHLVAFKAPKSWPNKVLINLWGCFSVIFLASYTANIAAHFAGLFFQMQVHDFHDTSVP EGYVFAENK

RLWEHIQRFGVASLEQGLESLRLGTLDVLIGDTAVLNYYRANEPSCRLRLLGDSIFDDAYAVGMARGFPLTA AVSELILR

YNAVGYLDQLHGKWYGRAHCLQDGLLQRLDKPMPLGVRAVAGLFIMLLVGLLAGSLVLILEHLVFRYALP GLRARSKQCF

WKSPNLMFFSQKLYRFINTVELVSPHHSAKEIVSNLREGQIASLFQKSVKRKIKEEARRRKSKSQFFEMIQEIR RVVRQQ

QTERDEESPTSPHEGPFSATSSIGPLLVREPSPPSGSSSSSAPTSSWVHGALLSVSLEELPSVAPPACRRRRSHSL GDLS

HMRPAWQPRRAPPSCASDLDDVRLRALSREQLLHRWRDSERRLLNLLREALREKQALERKLAFLHSALRNK PP

>Isc010976

GASFSFRRLLKKQKLLRGETGRVRFDDRGDRLDSDYDILNVVRGNHAPVGQYWSMSMELDLELDRVVWPG NRTETPLGYV

IPKHLKVATIAERPFVWVRPVPDESDCLPSEIFCPWTNSSDGIAYPFCCEGYCMDLLQSLSQKLNFTYTLYQV QDGQYGT

FDFVNGSTHKRWSGMVGDLVRGVADMIIAPLTITPERSMEIEFTKPFKYQGITILAKKANKSSTLASFLQPFQK TLWILV

MVSVHVVALALYLLDRFSPFGRYKLPNSDATEEDALNLSSAIWFAWGVLLNSGIAEGTPRSFSGRVLGMVW AGFAMIVVA

SYTANLAAFLVLEKPESSLSGINDPRVGGVFSEYFTRVSSPGDETTRGRPQREMALASATRILPHTKTFSSGGA PNIVND

LQLDAFIWDSSRLEFEAAQHCELVTAGEQFGRSGYGIGLQRNSFWVDKVTLTLLEMHESESPLSVSFIILSSRQ GEDTVL

PRAWLGPFISAGVFILVGAGIVGGIGLIVIEIVYKKHQTRKQRRLELARHAADKWRGAVEVSQHTPTLFPTMR HRGVQLV

APSAVPLTLSGLPQNGANIHQGLNSHWNDGHRSLLHHK

>Isc012402

GGIFESGSQKAQLSFRYAIDQINKDISILPRTELMPIIHEVKQKDSFTASKALCTFFKQRVSAIIGPTSEAVTSFTKSAC

TNLNVPHLQTSWEPKSNRNPYTLNLFPDPEVFGKAYLDLIINKKWKSFTVLYEHQDSLILLKDVINSSLAQNHSVSLVQV

NPKLSFKKMLKDIGYQKQFNIILDVKTESLPLLFRQAMEVNMMTYYHNYIITSLDLHTLDLREFVGLQANITAFRLVDPS

RPEVMNVLRDIKIGSALWRYGADNHTSPAHIDTDTALTFDAVALLANGLHNADRVLQTLDLQSLNCEHPKPWRHGSSLLD

AMKMITIRGLTGDVRLEEKGVREEFTLDVLELKARGLKQVATWHPRTGLSYFANYTLISEKEFTDELEGFTLKVTTVETK

PYTMVYPDSENKTGNDRFYGYAIDLIAMLAKEIGFKHEFYLTEDGKYGSPQPNKEWNGMIKELLDRKADAAIVDLTITYE

RESAVDFTIPFMNTGISILFKKAEKSDPSLFSFLYPFSVDVWLYMLTAYLGVSLLMFIMGRFTPYEWVSPHPCVPEEGDL

QNQFNLPNSFWFTTGAIMQQGSEIAPIAISTRIASSMWWFFSLIMISSYTANLAAFLTAQRMTAPIENANDLAKQTKIVY

GCKEEGSTCLFFKNSDDPLMKRMWTTMLAARPKAFADSNEKAVERVKKGNYAFLMESSSIEYVVERDCNLTQIGGLLDSK

GYGIATPPGDNL

>ISCW012407-PA conserved hypothetical protein|protein_coding|DS907449:516555:547561:-1|gene:ISCW012407

MGHPPYVIAKSSGRFIGVAAVNGTSELRLTGFAIELLDILARALGFDYNVRVSGEDYGSLSPNGTWTGLVRQVYEREADL

GVGDISITRERLEYVDFTAPFMQNGIGILYRNVDRQFEEIPHFLRPWTSELWLYVLTAVLATSLLFSFLSRLSSAEWVCK

RRAGPSCRKLAGKEHLKNRFTLINSFWFVLASLLQQGTDMFPRYELQLLLFFVELAKLT

>Isc015703

MNSSVLPHLIVAFPDSPPFMTPKYRNGKLYLDGIFGHILQAVAQFVPFSYELVYDRKRTYGVRLPNGTFNGIMGLLQSGE

ADLAAAPLIVRQDRAEAINFTPVIYTTYLSLLAVAGEPTANAFSYILAFDWQVWAILFVSIPILSALLAAIETRRARNKH

KYLREFHDNFWDLLRTISYEGLDAAIRTTPGRLMMATWWLTVLVLTNAFAGHMKACMSIKTEPPRFSSIDDVVLHPKIRP

MIWKDTGYEFYIRTSEKESLRMLSRMAQKHGGFVPLSEMQSPAAVAQVYSGRAVYVNDRDSMLYLLSKQCGRTGSLYVAP

ELIFARFCAMAYSRKLAHHISRRIDLGVQAVIDSGLATKWYADGLVEWERCRMMQEENAGGEMSYKPLLFQDLSAVFILW

GLSVSFALMAFVLESLAFWMERR

>Isc016542

LLKLQQIFHMTSNKKLDVRVVRRFMNVSDANAFLHEMELDDRESRKYVVLDSSAEVAKEIIINHVRDLYTGR
RNFHFLLT

SLYFNNQILELGVINVTGFRMLQSNRDEFKIFHKSWEKLDHSKWPGAGTAYVSADVALMHDATKVILDAYS
RLLKNKSDI

FRNNFRRGEVYNRGTKGIDCQSWPAVTWEHGKVIADYMKQTQIKGLTGNISFDSQGYRVNFSIDVVEMTINS
EMVKIADW

TDTRGLSSHPPKYKRVKQDNEFENKTYIVTSILEEPYLMFKKPEPGQVLVGNDQFEGYCKDLADLVAEHIRIT
YVLKLVN

DSKYGGRDVNSPAGWNGMVGELIRQEADMAIAPLTITSARERVIDFTKPFMSLGISIMIKKPMKMKPGVFSF
MNPLSREI

WMCIIFAYVGVSVVLFLVSRFSPHEWRYEETFVGPSVSNDFSLYNSLWFSLGAFMQQGCDICPRSVAGRIVGS
VWWFFTL

IIISSYTANLAAFLTVERMVTPINSADDLAKQTEVEYGTLSFSSTQEFFRRSKIAVYARMWEFMNTRKHVFTST
YEEGIR

RVRESKGKYAFLMESTKNDYINERHPCDTMKVGRNLDAKGYGVATPLGSNLRDRLNLAVLSMKENGDLAR
LENKWWYDRS

ECRSGDSKESTQNELTLSNVAGCFYILIGGLVLAMVVALLEFCYKSRMEAARSKMTMYEAMKAKVRMSITG
TQNGDRGRV

RQGSL

>Isc017534

MGVEMNGGSSGLMVSFSARMIGLVVCFVALSWHGCQAVRVGVLLRRGHNDVWREYEDAMTNFNNSDVA
SRLGIDIKTHRA

TFDDNLFITAQELCKQLNAGVTTVLLPSRGFSDAALYSLFGSTNVPRITTTVQQRCGSKDPPVLQEDVWNETT
TWTPSST

STLPPWFSDGSTTERTPDDEEDPTTLGVTMMPDLAPAVIDLADVWGFKSLVFVYDSDHALVTLQQFMDSGR
VRVQQARRV

SRSAEAHSMLSSLEKGDQQGRKLVVLDCAYDLAKDIVIRHVRDVYMGRRNYHYVLVKPIVSERYLEGVSEF
AALNITAFR

FQNTEGITQAYNYNYRTTLIINAYRTLKEDPPPKNADLFEDRVSSGYSGGTSCGRVAYLTKDQGQVVDAYLK
NVVFDGKT

GRVAFDSEGCRVNFTVDVMQVNGKNQWIQTGTWSKSSGFVRSGERNDSVPPGHKDYVYRVTTILEDPYLM
VKNKKYETDG

TNQTYEGFCKDLIDAISRLTGIKYQLHLVKDDHYGSVSIDGWNGMIGEIVSNDADIALAGLTITSARKNVVDF
THPFLTS

GIAALIKKPSKLQKGVGVNTFLAPFELHLWIGLGASLGAVLLFLFIFGAAVMKNDHCCYQAGDEDVSKDAVL
KTVCESLE

MVAPLPTGSFMARSISGRIVSSFWWMFVVLVFSTYTTTLTPLLTLRDAEYSSISTLEELSMQNKVKFGTLRTG
GVQEYFE

ESQYRTNKLIWEAMKTERHVLVGTRQEGVGRVRSSDGKYVFFTESLFADYVNGRRPCDTKVIGEVFATQYF
GLAVTPGSP

LREKLNEAIVNMTESGEIDEMKAKWWASECDAPPPDEPMRLELFLHIIFYLACLLIVGIAVALLELLVRGCNK
WRFSRLR

AVSARIDRERALRRSRQGDVTGVFPSCPAGVSDR

>Isc017535

LGVILKPDLNIVQSVYEGVMYNYSNSNVAQEIKFKVKTSFATLEDTDLFSTSRTLCEQLRGNVTTVVLSSTLH
SSLALQS

LFKNTNVPYVATSYQEHCSVNVGGNLNLPSADSLGVSLLPDYLPAVAEVVDHLAWDTFVYVYDSDNGPSKL
QRLLSHQFK

NSVSMRYAKRISNSSDANDFLRLLETTDRESRKYVLLDCRFETAKRIIIDHVRDIYMGRRNYHFLLVNPVVNE
LTYEKVP

EFVAVNITGLRLVGEDLSQQSSLDPSQEPREKKITEEDERRAGPQPRPRLEAEAELLEEGDQLLQADSPSTTTC
GDRPSV

PQELGEIITRNLREVRPKDTEVDVCSCNFVLLVPSIVLQTLLCFYVLLQRSFQGLTGSIRFTSDGCRIDYNVHVV
QLNVN

NEAIKIAEWSDTKGFEPVIKPARVEVNDTGVLDKDKTYVIQSVLKPSSWQRVPDAQDRMGNDRYDGYCKDL
IDALARELD

IKYELRAAEETVYGRRDHKVRGGWTGLIGEVLRKASQNAQSPSTATVINAERKEAVDFSQPFMTTGIAALML
KPSDLPGR

GMFTFLAPFSLELWIFFVSSFGLVFVIMFFVSFFTTRVSSTKPGMEHSACGTIYKSLCYSLEAFTPHYIDSYYAR
PYEVL

RSTGSYRRRYPIFPTLSRSISGRVIGNIWWLFIVFVFSAYTASMVPFLSKESRIRPIRSVEDLPLQSQVDYGFSRQ
SMAK

KYFENPNLNSTAHRRMWEVMNSKPDVFKNSNAEGVDAVRSSKGNYVFFMEANAVAFVNTQRPCDTMQLG
GTFGVRSFAVA

VPKGSSLRKHLDEAIAHLSETGELDKLKKKWWTQKSYCQYPERKKDETVMPLDNFIGVFFILGGGVALGILV
GLIEFI

>Isc022877

MAAKYRNTTLRLSNVGWYPYVFTVNQPDGTFRLKGITGSMIDYILASMGIKYQLVDPSDHKWGNLEPDGSW
SGMIGQINR

NEADLALGPFVLTEREAMVANPSPPYTDEDMIILSGVAKEHKSNVYGYISAFDLHVWMVLLCFLGFVATVA
VVAEYLLSN

PEMSIRQRRQRLTRIFWTFVASVFLESSTFNFTSHSQRLLFGFWFLMIVVLMNTFVSYMESALMLKEETDRVE
TIEQLAL

HPDIQPMVFEAAGFLQVIREAEGQAFQKVLRMMEEYDHEEEVHGEELFTPPYLSMVLKRKAVFLMERISLLA
RVSQHCYD

VELKGQGLFHLGRKRILQIKMVFYVRKTMDPVLQASATPPQEEIFKRVRWIMDSGLNLHWHEDLIPDPGPCW
TKFHPDHL

HDYASLNYQDFAVIFYLLLCSHGLAFCGYLSELVLFHWCCLKSRSRIGPNSYLN

>Isc023268

LYQVVFQAAIDIVNEDSSVNLEPDILPVVDILPEDDSYEAVRDACRLLEQGVAAIFGPMSGVSSVHVSSVCDS
LNVPHLE

TRWDPFMEDSDDSINIFPHPNVLAEAYTALMKYWEWSALAILYDEDDAIVQLKDLLQELREDGIEVSYFFRN
SSDSFRDV

LKKIKEAEYVNIFVAIEDEDLEVFLKQAMQIEMLGEEYNYLFMSLDFHVADLSDFIYAKANITALRIVDVEDP
VADIFQK

KIDENLLRTLSLATLRLTIVKHFVAKKKYLELRQVIGTKSRHSMSSEVLATPAITYGIYNAHFPQVAFDGLTGT VKFDEH

GFRKDFQLYVTELGTDGLEEIGTWSPTSGLNITKDEEEKGDDVVLRVVVISVSFVLHKPFVIVNNDEDGSPKF SGFCVDL

LQELSAMLDFKYELHASYDGRYGSLQEDGQWNGMVGELTNGNADLAMADLTITEQRERVVDFTLPFMSTG LAIVTKKGEP

MRPGGIWSFFLPLTREVWVYYVILATTCTIFVMYIGARLSFREWTLVEDGKGEECMENRFNLFNCFLFVVTTLL HQRITLD

PRAPATRVLAGFWYFFTFIILAIIVSNLCESILWEDDGPDYDTAQELLRARGFTYVVVHHGSSQKFLQDSKVPL HQRISS

LVERNYRNLPTSVGDGLQRVKDEDKVAFVMETASAAYATQEDCNFSRTDPFVFHSAYGFATPRDSPYRSKL SEGILKLQE

NGRLQWIQQRWWNPKR

>Isc023274

CNLISSGVAAIFGPNDPMIGAHIQSLSDSLDIPHIESRLDLEPDAKDCSVNLHPDPHITGKSMRDLVDYLNWTRI AVLYQ

DDISLISLQELVRPFPPKNVQYLFRRAENFTATLMDIKSRGIYSMIIDVRPENLTAFLRAILQIQMNDYKYHYHF ATYDI

EAYNLEDFQYNFVNITAYRMVDSDNQTVRHIMKDMEKFQPFGQNILNRTNVIITEPALMFDAVYAFAHGLN DLQRSTTLR

PANVSCEDEVSWTEGTSLFNLISMVDFYGLTGNIRFNEGNRTSLKLDLLKLRQNFLEKVGEWSTNDGLNITN HNAFHDFG

TSNITLRVTTIESRPYVMMKDNKNLTGNDRFDGFCIDLLRTIADLLGFNYELYLVPDKKFGVENTSTGEWNG MVREIIDK

NADLAVAPMTINYARESVIDFTKPFMNLGIGILFMLPSGMPVKLFSFMSPLDVDIWLYVLAAYILVSFTMFIV ARSDGAK

TSRTKKSRRYTEPQTRVLLYNLNIFFKLSCQKQLQSCDLNPKAMSTRIVGTIWWFFTLILISSYTANLAAFLTV ERMITP

IESVEDLAEQSKIAYGTLDSGSTMTFFRDSKIETYQKMWRYMENRPTVFVSTYEDGVARVLQGNYAFLMEST MLDFMVQR

DCNLTQVGGLLDSKGYGIATPMGSPWRDKISLAILDLQEKGVIQMLYNKWWKSPGLTCNRDDKNKEGKAN ALGLANIGGV

FVVLLCGLAVAIVTAVMEFCINSRKHAQTDRQSLCSEMAEELRFAVRCRASRQRPALRRQCSKCIPGTTYVPS AMDLPQE

NGIMQMLE

Fig. S8. Heat map of the expression profiles for Varroa IGR transcripts

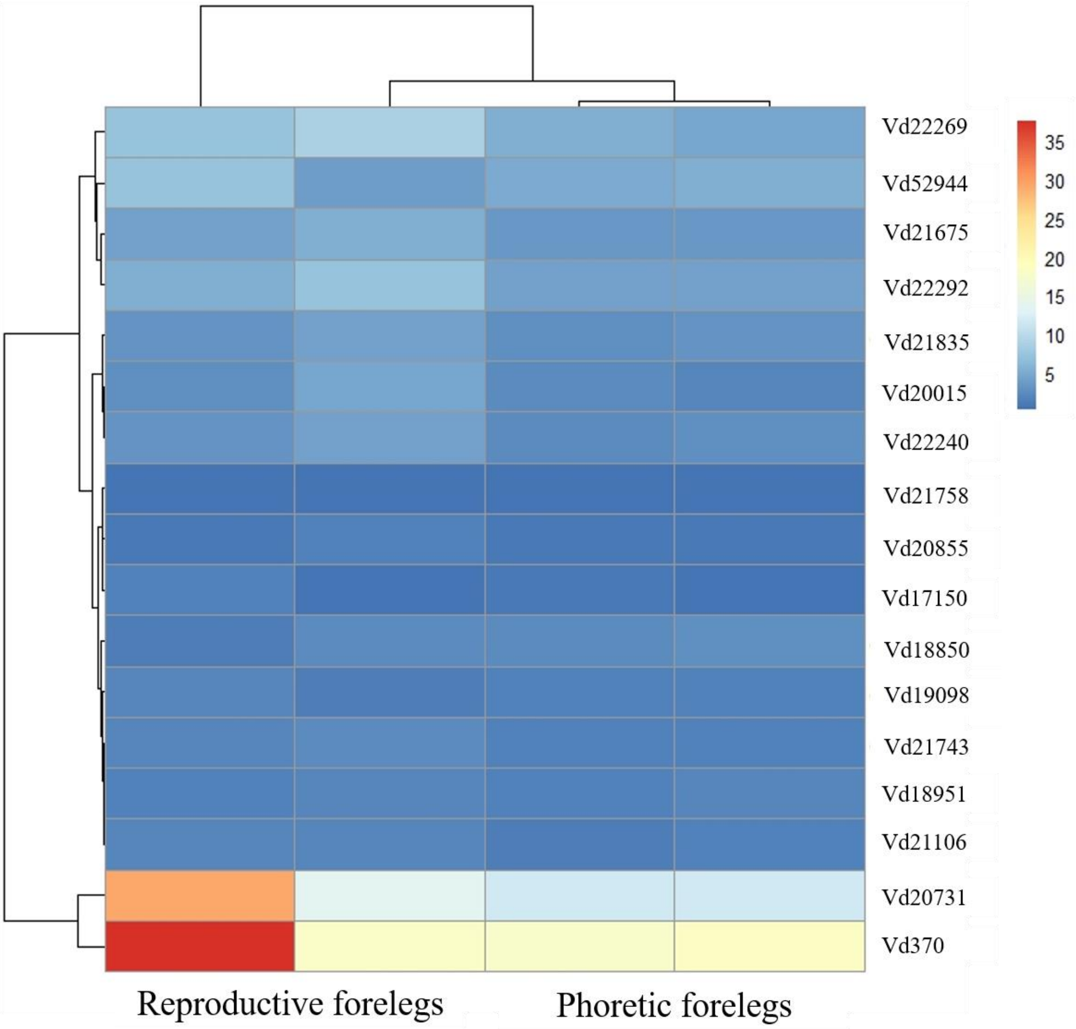

Heat map of the expression profiles for Varroa IGR transcripts, in the mite forelegs from two physiological stages: Phoretic and reproductive mites (two biological replicates of 50 pairs of legs for each replicate). The normalized expression level of the two conditions with two replicates is displayed with the color scale: highly expressed genes in red and low level of expressed genes in blue.

Fig. S9. Heat map of the expression profiles for Varroa GR transcripts

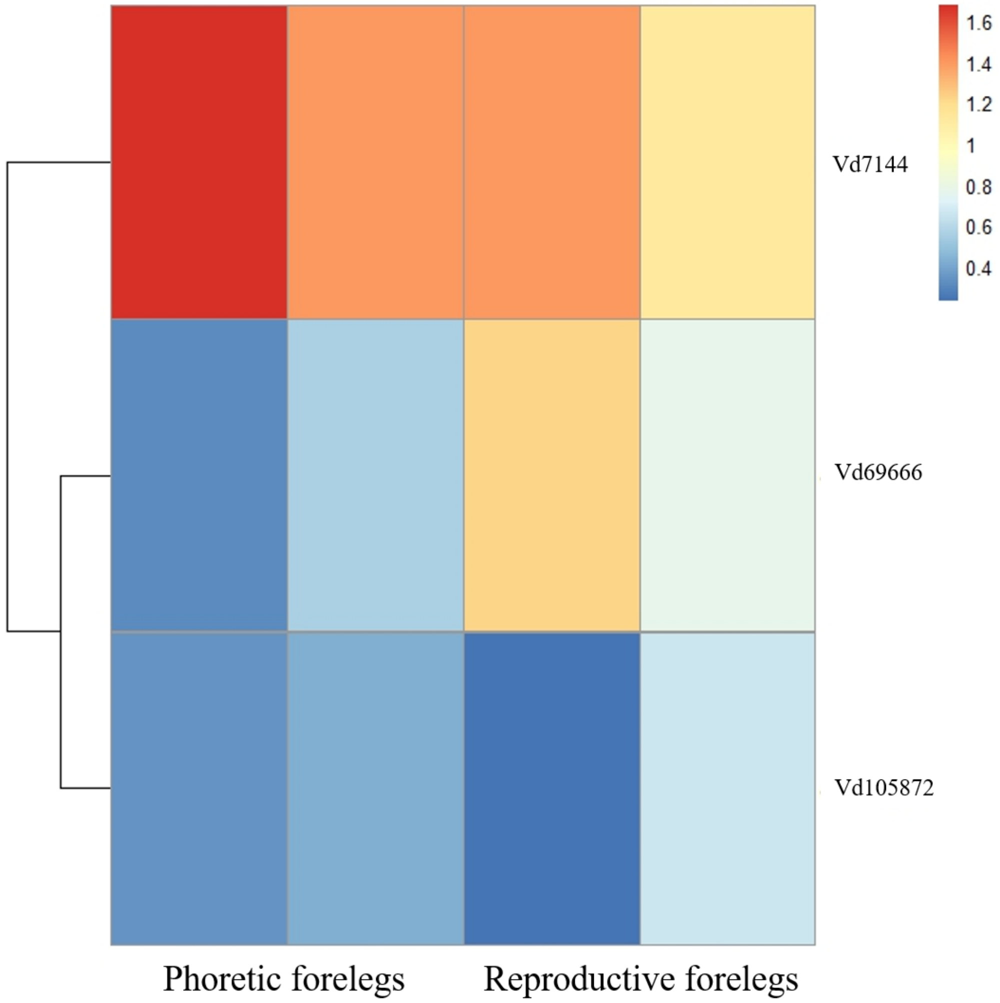

Heat map of the expression profiles for Varroa GRs transcripts, in the mite forelegs from two physiological stages: Phoretic and reproductive mites (two biological replicates of 50 pairs of legs for each replicate). The normalized expression level of the two conditions with two replicates is displayed with the color scale: highly expressed genes in red and low level of expressed genes in blue.

Fig. S10. Heat map of the expression profiles for Varroa NPC2 transcripts

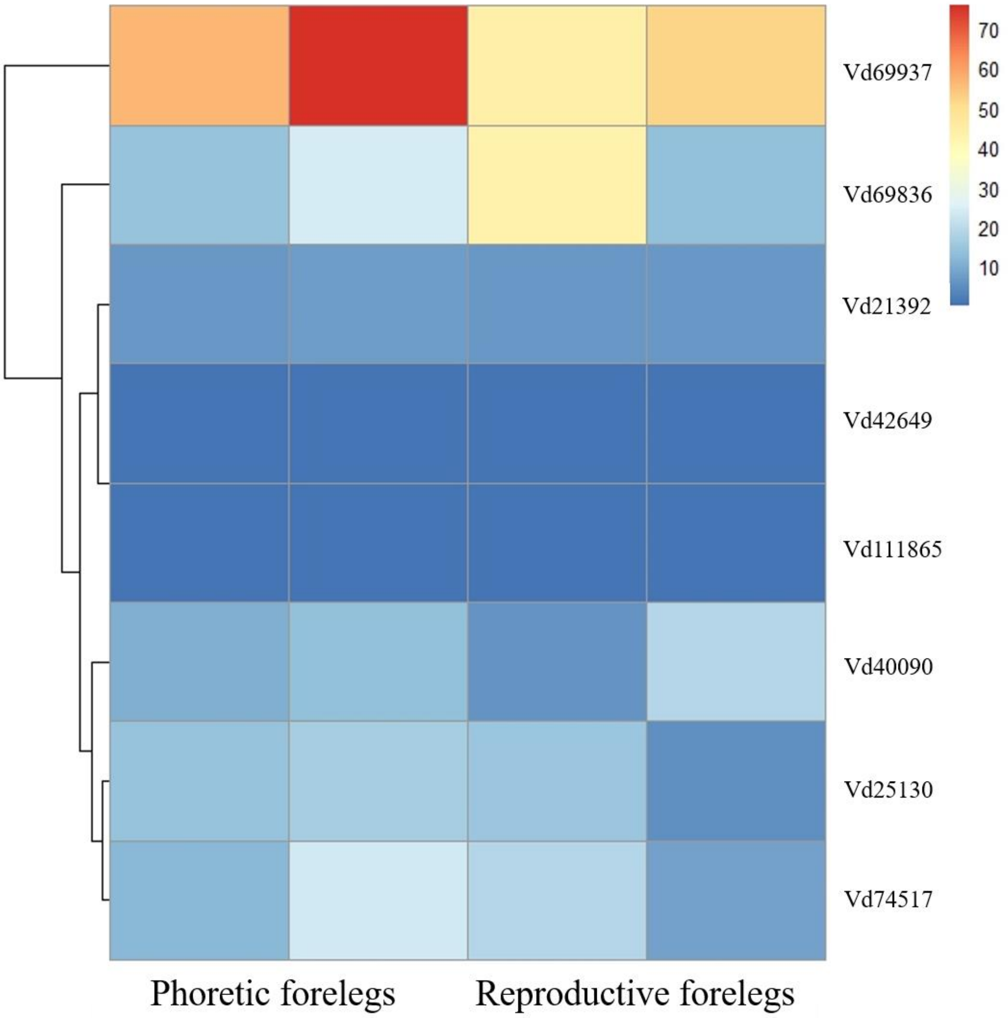

Heat map of the expression profiles for Varroa NPC2 transcripts, in the mite forelegs from two physiological stages: Phoretic and reproductive mites (two biological replicates of 50 pairs of legs for each replicate). The normalized expression level of the two conditions with two replicates is displayed with the color scale: highly expressed genes in red and low level of expressed genes in blue.

Fig. S11. Heat map of the expression profiles for Varroa SNMP transcripts

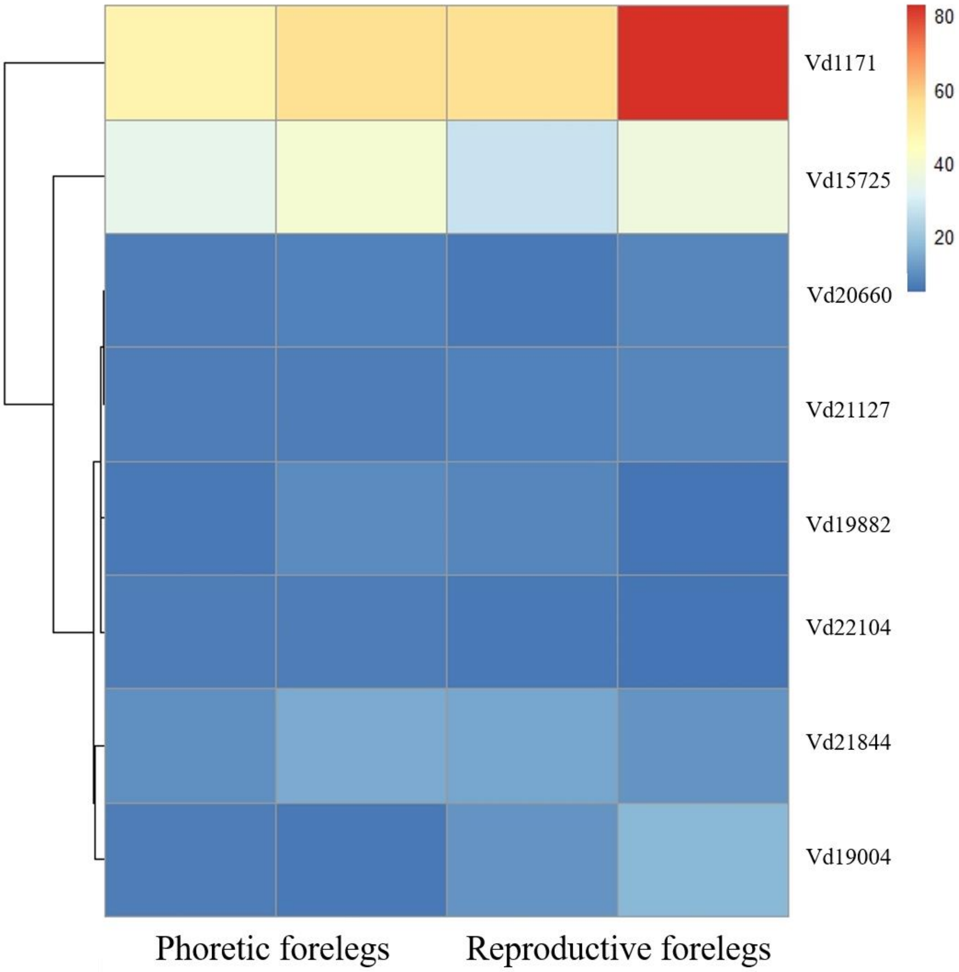

Heat map of the expression profiles for Varroa SNMP transcripts, in the mite forelegs from two physiological stages: Phoretic and reproductive mites (two biological replicates of 50 pairs of legs for each replicate). The normalized expression level of the two conditions with two replicates is displayed with the color scale: highly expressed genes in red and low level of expressed genes in blue.

Fig. S12. Heat map of the expression profiles for Varroa OBP transcripts

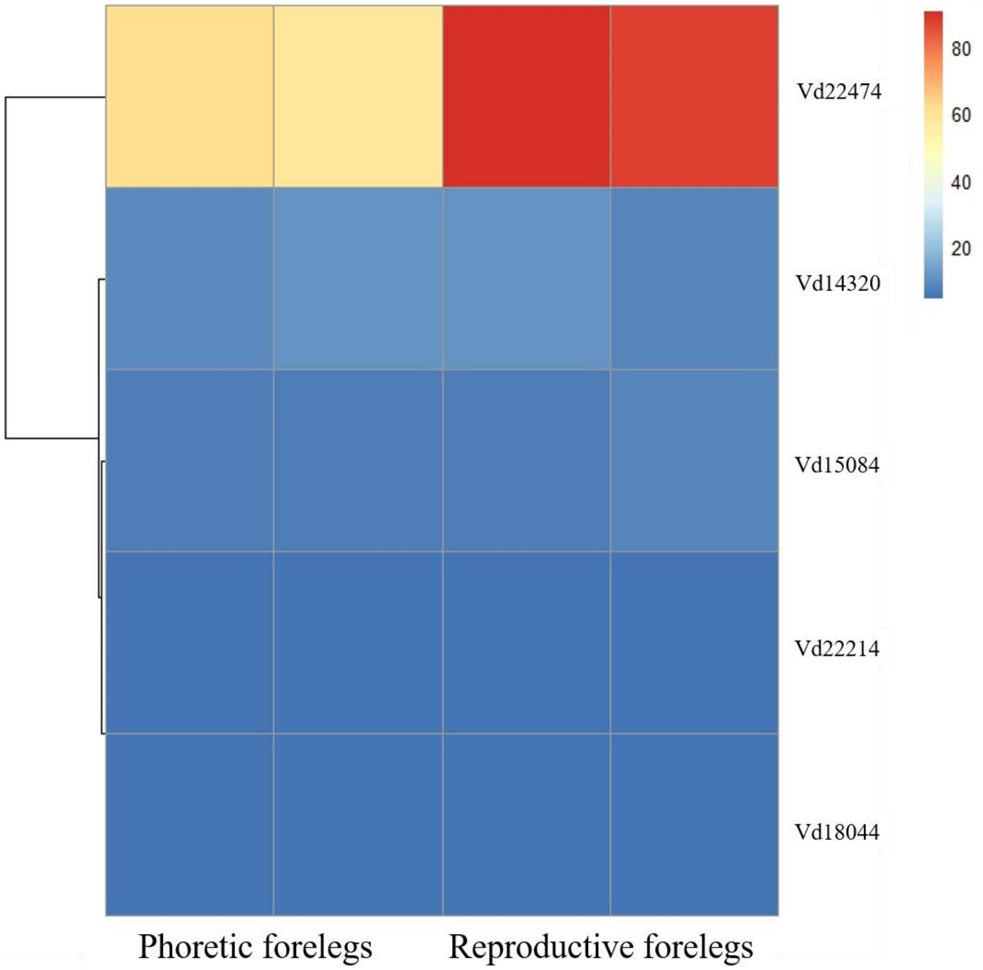

Heat map of the expression profiles for Varroa OBP transcripts, in the mite forelegs from two physiological stages: Phoretic and reproductive mites (two biological replicates of 50 pairs of legs for each replicate). The normalized expression level of the two conditions with two replicates is displayed with the color scale: highly expressed genes in red and low level of expressed genes in blue.

Data S13. Supplementary Table: Details of primer sequences of chemosensory related genes used for polymerase chain reaction (PCR) analysis.

Reference gene

Gene name	Product length (bp)	Primers' sequences	Tm°C
18S	60	Fwd 5'-AATGCCATCATTACCATCCT-3'<br>Rev 5'-CAAAAACCAATCGGCAATCT-3'	54.3

Odorant binding proteins (OBPs)

Gene name	Product length (bp)	Primers' sequences	Tm°C
Vd22474	300	Fwd 5'-CCAGCTAAAGCCTTCCGACA-3'<br>Rev 5'-TCACTCGTGTGCTTCTCACC-3'	60
Vd14320	174	Fwd 5'-CGTGCTGGGACTTGCTAACT-3'<br>Rev 5'-CCGTCCCTGCAGCTATCATC-3'	60.3
Vd15084	202	Fwd 5'-GATCCCGAAAAGGCGACACA-3'<br>Rev 5'-TTGCCTCCTTCTGACATGCG-3'	60.6
Vd22214	293	Fwd 5'-TTTTCACCCGCTGAGGATCG-3'<br>Rev 5'-CTGCAGAGCGTCCTGACATA-3'	60
Vd18044	326	Fwd 5'-GGGTTCGTGCCAATGGAAAC-3'<br>Rev 5'-CTGACCCTCAGTTTCAGCGT-3'	60

Ionotropic glutamate receptors (IGRs)

Gene name	Product length(bp)	Primers' sequences	Tm°C
Vd22269	377	Fwd 5'- CTCGCTCGGATGAAGACCTC-3'<br>Rev 5'- ATAGGTGTCCGGGCTTAGGT-3'	60
Vd52944	228	Fwd 5'- CCAGATGAAGCCCTTGAGCA-3'<br>Rev 5'- GGTCGCATTATACGTCCCGT-3'	60
Vd21675	387	Fwd 5'- GGTCATGAAAATCGGCGCTC-3'<br>Rev 5'- GATGGCGGTAGCAGATCGAA-3'	59.9
Vd22292	845	Fwd 5'- CGATGCGAGAGCAAGCTAGA-3'<br>Rev 5'- CGTTGGCGCACAAACTAACA-3'	60
Vd21835	407	Fwd 5'- TCGCAGATGGACGATGCTAC-3'<br>Rev 5'- ATTTGGGTGGTGATCGGCAT-3'	60
Vd20015	224	Fwd 5'- GTTCGCCGTGCTCTACGATA-3'<br>Rev 5'- TTTGATTCACCTCCTCCGCC-3'	60
Vd22240	698	Fwd 5'- GCCTCCCTGCTGAAACAGAT-3'<br>Rev 5'- CTGTCAGCTTCTTCCTGGGG-3'	60
Vd21758	705	Fwd 5'- ACTGCGATAAGCGGACCAAA-3'<br>Rev 5'- AGCTGTACACATTGGCCTCC-3'	60

Vd20855	350	Fwd 5'- ACGAGTTGCGGCTAGTCAAA-3'<br>Rev 5'- CTCCTCGTAGCGCCATTCAT-3'	59.9
Vd17150	683	Fwd 5'- CGCATTCATTGCTGATGCGA-3'<br>Rev 5'- GCAGGTAAGCCCCATTGACT-3'	60
Vd18850	535	Fwd 5'- GGACTTAGCATGTCCCTCCG-3'<br>Rev 5'- GGACAAATTGCACGGGAACC-3'	60
Vd19098	277	Fwd 5'- TAAAGTTCTCGCGGTCCGTC-3'<br>Rev 5'- GTCTCGAAGTTCGCGTCTCT-3'	60
Vd21743	348	Fwd 5'- GAAAAGTTCCCGGCAACCAC-3'<br>Rev 5'- GATAGCGGTCCAGGAAGTCG-3'	60
Vd18951	366	Fwd 5'- TTTGGCCGCCTTTTTAACCG-3'<br>Rev 5'- AGCACTTGTCTGAATGGCGA-3'	59.9
Vd21106	639	Fwd 5'- GCGGCTATACTCGCAGTCTT-3'<br>Rev 5'- CTCGCGAAGCTTCTCAATGC-3'	60
Vd20731	626	Fwd 5'- TCCCAGGGCTGTCTCTACTC-3'<br>Rev 5'- CGCCTATTAAAACGCCACCG-3'	60
Vd370	178	Fwd 5'- CAAAAAGCCGAGCGGGAAAA-3'<br>Rev 5'- CGAGGCGCAGAGCTGTAATA-3'	59.9

Gustatory receptors (GRs)

Gene name	Product length (bp)	Primers' sequences	Tm°C
Vd7144	320	Fwd 5'- CATGCATGGAAGCCACAACC-3'<br>Rev 5'- GGCACCAAACGTCTGTAGGA-3'	60
Vd69666	91	Fwd 5'-GTCGCGCAGATGAATAAGCC-3'<br>Rev 5'-GAGCTCCGCCGATTGTTAGA-3'	59.9
Vd105872	453	Fwd 5'-CCCAGAGGGTCTGGACCTAA-3'<br>Rev 5'-GCGTAGAGAGCTCGAGTGAC-3'	59.9

Niemann-Pick disease protein, type C2 (NPC2)

Gene name	Product length (bp)	Primers' sequences	Tm°C
Vd21392	294	Fwd 5'- GGCCATGTTTTAAGCCGTCC-3'<br>Rev 5'AATCGCCGGAAGTTAGCCAT-3'	59.8
Vd42649	194	Fwd 5'- TGCGAGATTAAGAAGGGCGG-3'<br>Rev 5'AAACCGCCTATCACGGGTTC-3'	60.3
Vd69937	189	Fwd 5'- AGGTCCAGAACGTACAGCAG-3'<br>Rev 5'ACTCTGCTTCGCCAACGAAT-3'	59.6
Vd111865	194	Fwd 5'-AGTGGGAGATCCGAGCTTTC-3'<br>Rev 5'-TGGGCACATTGGAAGAAACG-3'	59.1
Vd25130	326	Fwd 5'- ATGCAGATTTCGAGGCGGAT-3'<br>Rev 5'ACCATGCCGTCCAAACTCAA-3'	60
Vd69836	120	Fwd 5'- CTTATCCGCTGCCAAACAGC-3'<br>Rev 5'GCGCAATAGAAGGAAAGCCG-3'	59.9

Vd74517	346	Fwd 5'- AGCCGACGCTACGATTTCAT-3'<br>Rev 5'GTCGACGCAGTACAAGCTCT-3'	60
Vd40090	281	Fwd 5'- ATAGCGTTCGCGTACAACCT-3'<br>Rev 5'TGATTCAGCCAGGATGCAGT-3'	59.6

Sensory neuron membrane proteins (SNMPs)

Gene name	Product length (bp)	Primers' sequences	Tm°C
Vd11771	666	Fwd 5'- CTGGCATCGTTGCCTTCTTG-3'<br>Rev 5'- GCATATTTACCCGCGTGCTG-3'	59.9
Vd15725	662	Fwd 5'- TTACAGCTGGCAGTCTCACG-3'<br>Rev 5'- TTTGCTCCCCAGATGCCATT-3'	60
Vd19004	693	Fwd 5'- TGCCTGATACCAAGCTTCCG-3'<br>Rev 5'- CCTCCATACGCTCTTCGACC-3'	60
Vd19882	535	Fwd 5'- CACATCTGGCAGTGACCTGT-3'<br>Rev 5'- AACAGAAGTTCGCGTACCGT-3'	60
Vd20660	141	Fwd 5'- ATGGGGTGGTCGTCATCAAC-3'<br>Rev 5'- TACCCTTTTCTTGCCGTCCC-3'	60
Vd21127	256	Fwd 5'- ATCTCCGCGAGTTTGTTGGT-3'<br>Rev 5'- CCCATTCGGATCGCCATGTA-3'	59.9
Vd21844	295	Fwd 5'- ACCGGAGAGGATAACCGTCA-3'<br>Rev 5'- TCGAACCAGCGAAACATGGA-3'	60
Vd22104	273	Fwd 5'- GTTCCAAAGACACCAACGCC-3'<br>Rev 5'- AGCTATCCAAAGCCGGATCG-3'	59.9

Tm°C: melting temperature

PCR conditions: The PCR amplification program was as follows: 94 °C for 1 min followed by 35 steps of: 94 °C for 30 s, 56 °C for 30 s and 72 °C for 1 min. After 35 cycles, samples were brought to final extension at 72 °C for 5 min.

Data S14. Supplementary Table: Details of primer sequences of ionotropic receptor-like genes used for quantitative polymerase chain reaction (qPCR) analysis.

Gene name	Product length (bp)	Primers' sequences
Vd22240	217	Fwd 5'-ATCGGCAGTCAACGGAATGT-3'<br>Rev 5'-GTTGAAGCGTGGAAGTGACG-3'
Vd17150	157	Fwd 5'-ACGCTTCCTTGAGGCTTTCT-3'<br>Rev 5'-ACAATGACCAACTGCGACCT-3'
Vd18850	169	Fwd 5'-CCAACGTATAGACCGTGGGC-3'<br>Rev 5'-CGGAAAACGCTGCATCCAAT-3'
18S	60	Fwd 5'-AATGCCATCATTACCATCCT-3'<br>Rev 5'-CAAAAACCAATCGGCAATCT-3'

QPCR conditions: The PCR amplification program was as follows: 15 min activation at 95 °C, 40 cycles of 15 s at 95 °C, 30 s at 60 °C and 30 s at 72 °C. A melting ramp from 72 to 95 °C was used with a 1 °C rise at each step and a 5-s interval between steps.
